# Exposure to Phthalates in European Children, Adolescents and Adults since 2005: A Harmonized Approach Based on Existing HBM Data in the HBM4EU Initiative

**DOI:** 10.3390/toxics11030241

**Published:** 2023-03-04

**Authors:** Nina Vogel, Rosa Lange, Phillipp Schmidt, Laura Rodriguez Martin, Sylvie Remy, Andrea Springer, Vladimíra Puklová, Milena Černá, Péter Rudnai, Szilvia Középesy, Beata Janasik, Danuta Ligocka, Lucia Fábelová, Branislav Kolena, Ida Petrovicova, Michal Jajcaj, Milada Eštóková, Marta Esteban-Lopez, Argelia Castaño, Janja Snoj Tratnik, Anja Stajnko, Lisbeth E. Knudsen, Jorma Toppari, Katharina M. Main, Anders Juul, Anna-Maria Andersson, Niels Jørgensen, Hanne Frederiksen, Cathrine Thomsen, Amrit Kaur Sakhi, Agneta Åkesson, Christina Hartmann, Marie Christine Dewolf, Gudrun Koppen, Pierre Biot, Elly Den Hond, Stefan Voorspoels, Liese Gilles, Eva Govarts, Aline Murawski, Antje Gerofke, Till Weber, Maria Rüther, Arno C. Gutleb, Cedric Guignard, Tamar Berman, Holger M. Koch, Marike Kolossa-Gehring

**Affiliations:** 1 German Environment Agency (UBA), Department of Toxicology, Health-Related Environmental Monitoring, 14195 Berlin, Germany; 2 Flemish Institute for Technological Research (VITO), 2400 Mol, Belgium; 3 National Institute of Public Health, Centre for Health and Environment, 10000 Prague, Czech Republic; 4 National Public Health Center, Environmental Health Unit of the Department of Public Health Laboratory, 1097 Budapest, Hungary; 5 Nofer Institute of Occupational Medicine, 91-348 Lodz, Poland; 6 Department of Environmental Medicine, Faculty of Public Health, Slovak Medical University, 83303 Bratislava, Slovakia; 7 Department of Zoology and Anthropology, Faculty of Natural Sciences and Informatics, Constantine the Philosopher University in Nitra, 94901 Nitra, Slovakia; 8 Public Health Authority, Department of Environment and Health, 83105 Bratislava, Slovakia; 9 Instituto de Salud Carlos III, 28220 Majadahonda, Spain; 10 Department of Environmental Sciences, Jožef Stefan Institute, Jamova Cesta 39, 1000 Ljubljana, Slovenia; 11 Department of Public Health, University of Copenhagen, 1165 Copenhagen, Denmark; 12 Research Centre for Integrative Physiology and Pharmacology, Institute of Biomedicine, University of Turku, 20520 Turku, Finland; 13 Department of Pediatrics, Turku University Hospital, 20521 Turku, Finland; 14 Department of Growth and Reproduction, Copenhagen University Hospital—Rigshospitalet, 2100 Copenhagen, Denmark; 15 International Centre for Research and Research Training in Endocrine Disruption of Male Reproduction and Child Health (EDMaRC), Copenhagen University Hospital—Rigshospitalet, 2100 Copenhagen, Denmark; 16 Department of Clinical Medicine, University of Copenhagen, 2200 Copenhagen, Denmark; 17 Department of Food Safety, Norwegian Institute of Public Health, 0456 Oslo, Norway; 18 Institute of Environmental Medicine, Karolinska Institutet, 171 77 Stockholm, Sweden; 19 Environment Agency Austria, 1090 Vienna, Austria; 20 Hainaut Analyse, 7000 Mons, Belgium; 21 Federal Public Service Health, Food Chain Safety and Environment, 1060 Brussels, Belgium; 22 Department of Environment and Health, Provincial Institute of Hygiene (PIH), 2000 Antwerp, Belgium; 23 Environmental Research and Innovation (ERIN) Department, Luxembourg Institute of Science and Technology, L-4422 Belvaux, Luxembourg; 24 Department of Environmental Health, Ministry of Health, Jerusalem 9446724, Israel; 25 Institute for Prevention and Occupational Medicine of the German Social Accident Insurance—Institute of the Ruhr University Bochum (IPA), 44789 Bochum, Germany

**Keywords:** phthalates, pollutants, human biomonitoring, exposure, HBM4EU

## Abstract

Phthalates are mainly used as plasticizers and are associated inter alia with adverse effects on reproductive functions. While more and more national programs in Europe have started monitoring internal exposure to phthalates and its substitute 1,2-Cyclohexanedicarboxylic acid (DINCH), the comparability of results from such existing human biomonitoring (HBM) studies across Europe is challenging. They differ widely in time periods, study samples, degree of geographical coverage, design, analytical methodology, biomarker selection, and analytical quality assurance level. The HBM4EU initiative has gathered existing HBM data of 29 studies from participating countries, covering all European regions and Israel. The data were prepared and aggregated by a harmonized procedure with the aim to describe—as comparably as possible—the EU-wide general population’s internal exposure to phthalates from the years 2005 to 2019. Most data were available from Northern (up to 6 studies and up to 13 time points), Western (11; 19), and Eastern Europe (9; 12), e.g., allowing for the investigation of time patterns. While the bandwidth of exposure was generally similar, we still observed regional differences for Butyl benzyl phthalate (BBzP), Di(2-ethylhexyl) phthalate (DEHP), Di-isononyl phthalate (DiNP), and Di-isobutyl phthalate (DiBP) with pronounced decreases over time in Northern and Western Europe, and to a lesser degree in Eastern Europe. Differences between age groups were visible for Di-n-butyl phthalate (DnBP), where children (3 to 5-year olds and 6 to 11-year olds) had lower urinary concentrations than adolescents (12 to 19-year-olds), who in turn had lower urinary concentrations than adults (20 to 39-year-olds). This study is a step towards making internal exposures to phthalates comparable across countries, although standardized data were not available, targeting European data sets harmonized with respect to data formatting and calculation of aggregated data (such as developed within HBM4EU), and highlights further suggestions for improved harmonization in future studies.

## 1. Introduction

Human biomonitoring (HBM) has become a well-recognized tool to measure chemical burdens in (sub)populations for exposure assessment, risk assessment, and risk management [1,2,3]. Many countries in Europe have conducted HBM programs over the last 20 years, some on a regular basis [4]. However, since these programs had previously worked independently of each other, comparison of HBM data has been challenging. The twin project COPHES/DEMOCOPHES (2009–2012) was the first initiative to close this gap. Here, the EU and 17 European countries conducted a Europe-wide HBM feasibility study, which formed the basis for a systematic approach to establish an HBM network for Europe [5,6,7].

In 2017, the European Human Biomonitoring Initiative (HBM4EU) was launched, a joint effort of 30 European countries and the European Environment Agency, co-funded by the European Commission under Horizon 2020. A major goal of HBM4EU was to create a European network that improves the knowledge and factual basis for the European Union’s environmental and chemical policy by harmonizing the planning and implementation of HBM studies, sample analysis, and data analysis [8]. HBM4EU has prioritized a set of substance groups, for which policy-related research questions have been formulated. In the course of the project, knowledge and data were collected to answer these questions. Thereby, HBM4EU provides tailored results to directly feed into the development of European policies in the areas of health, environment, and chemical safety to eventually protect human health more effectively. From 18 substance groups that had been prioritized, phthalates have been one of the first [9]. 

Phthalates are a large group of phthalic acid esters, comprising many individual substances, but which often are used in combination or in isomeric mixtures. They are widely used as plasticizers with a production volume of millions of tons per year, but also have many niche applications such as solvents and formulation additives [10]. Several phthalates have been identified as substances of very high concern (SVHC) and are included in the candidate list for authorization under the chemical legislation (EC) No. 1907/2006 on Registration, Evaluation, Authorization and Restriction of Chemicals (REACH) due to their toxic effects on reproduction and due to their endocrine-disrupting properties on human health. In addition, many phthalates are restricted under the REACH regulation (see Appendix A Table A1). A common substitute for phthalate exposure is 1,2-Cyclohexanedicarboxylic acid (DINCH), which was introduced into the EU market in 2002 [11]. Unlike many phthalates, DINCH is assumed to be not toxic to reproduction or development and is not expected to be an endocrine disruptor [12]. Thus, as a non-phthalate plasticizer, DINCH is used in toys, food contact materials, and medical devices [12]. DINCH and some of the phthalates (e.g., DnBP, DEHP, BBzP) are allowed in plastic materials and articles intended to come into contact with food, but are subject to a group restriction, as the sum of substances cannot exceed 60 mg/kg of the plasticized material according to Regulation (EU) No 10/2011.

A key aspect in HBM4EU has been building on existing knowledge and capacity. Therefore, an inventory of national, regional, and local HBM studies from the partner countries which had investigated the exposure to several phthalates (among other priority substances) was made. The challenge is, however, that existing HBM studies differ widely in their sample collection method, time periods, study samples, degree of geographical coverage, and study design, but also in analytical methodology, biomarkers used, analytical sensitivities, and analytical quality assurance levels. To overcome, as much as possible, this heterogeneity of data collection, HBM4EU developed standardized protocols (SOPs) for data providers and data users to evaluate already-existing data in a statistically harmonized way. Of course, COPHES/DEMOCOPHES data were included in this approach, but they reflected only one part of the mosaic, as it only included mother–child pairs, with sample collection from 2011–2012. In HBM4EU, several research protocols were developed to investigate and answer policy questions on the group of phthalates: cumulative mixture risk exposure to phthalates in children and adolescents [13], impact and results indicators for European HBM [14], time trends of phthalates and DINCH with repeated cross-sectional data [15], current exposure of European children and adolescents to phthalates and DINCH [16], exposure determinants in the European population (in preparation), and exposure distributions for various age groups of the European population from 2005 to 2019 (this study).

Thus, the aim of the present paper is to describe population exposures to relevant phthalates in Europe after harmonized formatting of the data and calculation of the aggregated data through HBM4EU. We expand on previous reports on the internal exposure to phthalates in the European population ([17]; 6–11-year-old children and their 40–59-year-old mothers) by including additional age groups (3–5, 12–19, 60+), males and females, all four European regions, and a broad sampling period (2005–2019), all with the intention to investigate for potential associations, differences, and/or trends. In addition, we also checked the data availability of DINCH, a major phthalate substitute.

## 2. Materials and Methods

### 2.1. Data Collection and Harmonization

To reduce the degree of heterogeneity between existing HBM data, participating partners in HBM4EU were asked to prepare their data sets in a harmonized way. The data template developed and provided by HBM4EU ensured a standardized format and included, for example, defined variables and variable names, required units of concentration, and respective limits of quantification/detection, but also categorization of sampling types (e.g., first morning urine void (mU), 24-h urine (24hU), etc.) and some basic characteristics (e.g., age, sex, education level). Once individual data of a data collection were in the required format, a standardized procedure using a R-script, which was developed within HBM4EU, was applied. This procedure aggregated individual data with similarly calculated statistical measures (e.g., handling of samples with values below the respective detection/quantification limits) with the option for the data provider to select additional stratification groups if they wanted aggregated data for subgroups (e.g., females and males, smokers and non-smokers, etc.). The resulting harmonized anonymized aggregated data were shared with the data management team of the project which ran basic data quality checks (e.g., values out of expected range). Once all outputs for all data collections were merged into one data file the aggregated data were made accessible for all HBM4EU partners through a project-internal share point with the option to make it available publicly after the end of the project, and the data were integrated in the European HBM dashboard (https://hbm.vito.be/eu-hbm-dashboard, accessed on 20 December 2022) and IPCHEM (https://ipchem.jrc.ec.europa.eu/#showmetadata/HBM4EUAGGREGATED, accessed on 20 December 2022).

The following inclusion criteria and checks were required before data controller provided data from their national study program. All data collections were from national studies, with ethics and data approvals at the time of sampling. Some collections have supplementary approval for further analysis within HBM4EU, dependent on the conditions specified in the initial approvals. In addition, aggregated statistics in the harmonization procedure were per default only displayed for data collections or subgroups of data sets when they had at least 50 analyzed samples (recommended in HBM4EU to fulfil data protection) and can as such be considered anonymous data. Data were requested for the in HBM4EU priority substances, as well as some basic characteristics (sex, age, education, creatinine etc.), which were used to describe the data collection and/or to aggregate data for subgroups. Further limitations were not set to give a variety of study programs the possibility to share data.

For the current endeavor to present internal exposure to phthalates and DINCH in the European population, the following inclusion criteria were defined. First, all data collections with harmonized aggregated data in HBM4EU with available phthalate data measured from the year 2005 on were identified in the project intern share point (status 12/21/2021). Since the focus was on the general population, data collections that have exclusively sampled hospital patients or pregnant women, or that have targeted occupational exposure or hotspots, were excluded. Both regional and national representativeness were allowed within studies and data collections targeting all age groups. Because urine is currently regarded the preferable matrix for the HBM of plasticizers in HBM4EU [18], only analyses in urine were included (applied to all identified data collections). Next, study contact points of each data collection were asked for permission to use the anonymized aggregated data, which were readily available within the HBM4EU project, and were invited to participate in this study. The 29 identified data collections fitting these inclusion criteria are shown in Table 1. They cover all European regions and Israel, with Eastern and Western Europe being represented by four countries each, Southern by two, and Northern by three, respectively. Among the data sets are 11 DEMOCOPHES studies [17], leading to an overrepresentation of women of child-bearing age and 6–11-years-old children.

For some data collections, additional specific information is provided. Israel participated in the HBM4EU project because they were interested in expanding and solidifying their HBM network and capacities, and harmonizing their program and data collections with the European HBM programs [45]. Although the PCB cohort (Slovakia) is situated in the area of environmental polychlorinated biphenyls (PCB) contamination, resulting from the former PCB production in the area, this data collection has been included because there are no known relations to phthalate exposure. For the Danish data collection COPENHAGEN Puberty Study, only the main sample has been included in this publication, excluding smaller additional subsets for specific purposes (e.g., longitudinal design). Since it was not possible to implement population-representative weighting in the harmonization procedure for the German data collections GerES IV and V, unweighted results are displayed here (labelled GerES IV/V (unweighted)). In addition, since GerES IV was carried out in 2003–2006, we used the yearly stratified data from GerES IV to fit this publication’s inclusion criteria, only including data from 2005 onwards. 

Since this approach focused on existing data, the analytical data were not necessarily generated under a common external analytical quality assurance scheme guaranteeing utmost comparability. Such an approach, designed for a common, harmonized chemical analyses, has also been developed within HBM4EU [46,47] and applied to recently collected samples [16]. However, the general data quality of studies included in our current approach was evaluated by the study owners, supported upon request by the analytical experts within HBM4EU. Some continuity and comparability were ensured by the previous participation of laboratories in the external quality assessment schemes of COPHES/DEMOCOPHES [48] or in commercial round-robin tests [49], albeit with a more limited biomarker spectrum.

In our analyses, we included only those phthalates/biomarkers assigned in HBM4EU to Category A and B, namely for which reliable and broad HBM data (A) or at least fragmented HBM data (B) existed [9]. An overview of phthalates and their respective metabolites measured in the available data collections can be found in Table 2. In total, 32 metabolites from 12 phthalates were represented in the selected data, using the following collection methods for sampling: first morning urine void (mU), 24-h urine (24hU), or spot urine (sU) for phthalates. 

### 2.2. Statistical Analysis

An R script/application was developed within HBM4EU to calculate the harmonized aggregated statistics for each data collection individually, including geometric mean and several percentiles (P05, P10, P25, P50, P75, P90, P95) with their 95% CIs [50]. To harmonize the data, first, each individual data set was requested in the same format (e.g., unit of concentration). Then, for each data collection, values below LOD, values between LOD and LOQ, and values below LOQ (when LOD is not known)—whichever applied to the respective data collection—were imputed with the same procedure when at least P50 was above LOD or LOQ, providing estimates for a normal and lognormal distribution via a percentile regression. In the next step, statistics such as the geometric mean were calculated for each individual data collection and/or stratified groups selected by the data providers (e.g., age groups) when the subsample consisted of at least 50 analyzed samples. Among the aggregated data output is a fitting index (R^2^) which indicates whether imputation of values below LOQ/LOD based on the normal or log-normal distribution fit the data better. Since R^2^ was higher for the latter, imputed statistics based on log-normal distribution were used for the current purpose.

## 3. Results

Overall, 29 studies from Europe and Israel were identified. With the exception of one study (Environmental Specimen Bank, Germany), which measured 24hU, all other studies measured phthalates and DINCH in random sU (7 studies) or first mU (21 studies) urine. As expected, due to contribution of samples collected during DEMOCOPHES, the most studied sub samples were 6–11-year old children, and 20–39- and 40–59-year-old mothers. For a given compound, there were at most five data sets (for the age group of 3–5-year-olds, the youngest age group included here) and up to nine data collections (for the age group of 12–19-year olds). We were able to identify one data collection with phthalate data for participants 60 and older (PBAT, Austria). The most data were available for substances Di(2-ethylhexyl) phthalate (DEHP) (metabolites Mono(2-ethylhexyl) phthalate (MEHP), Mono(2-ethyl-5-hydroxy-hexyl) phthalate (5-OH-MEHP), Mono(2-ethyl-5-oxo-hexyl) phthalate (5-oxo-MEHP); 63 data sets across all age groups), Diethyl phthalate (DEP) (60) and Butyl benzyl phthalate (BBzP) (59), and the least data were available for Bis(2-propylheptyl) phthalate (DPHP) (2 German studies) and DINCH (3), respectively.

For easier comparisons of European exposure, we grouped the available studies according to age groups (3–5, 6–11, 12–19, 20–39, 40–59, 60+), chronologically by year of sampling, and the urine collection type (mU, 24hU, sU) in tables, and reported in Supplementary Tables S1–S26 how many samples were collected, the fraction of values below LOD/LOQ, the geometric mean and its CI (imputed as described in the methods section if there were values below LOD/LOQ), P50, P75, P90, P95, and their CIs.

Because it is the intention of HBM4EU to deepen the knowledge on less-investigated phthalates and DINCH (Category B), we will focus on the interpretation of these results, and only highlight results for phthalates from Category A.

### 3.1. BBzP, DEHP, DnBP, DiBP, and DEP (Category A Phthalates)

**Figure 1:**
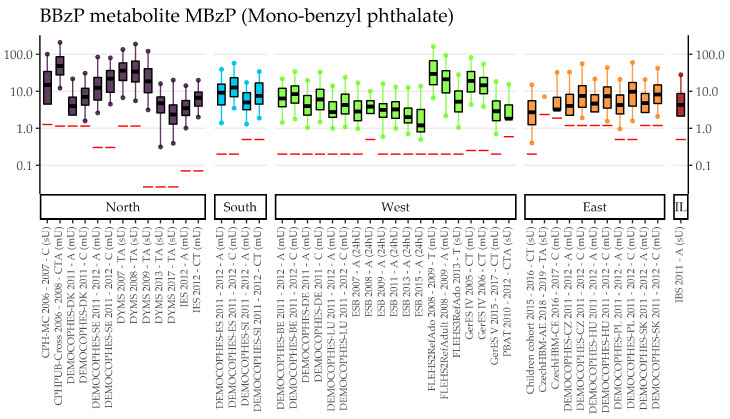
BBzP metabolite MBzP concentration in µg/L in boxplots. The box is based on P25, P50, and P75, and the whiskers are based on P5 and P95. Boxplots are presented separately by regions (North = violet, South = blue, West = green, East = orange, Israel = dark red) within the regions chronologically sorted by year of sampling. Red lines denote LOQ/LOD for the respective study. Since percentiles are only available and displayed when they are above LOQ/LOD, the lower side of boxplots might be truncated. The age categories are shown in capital letters: children of the age groups 3–5 and 6–11 (C), adolescents between 12 and 19 years (T) and adults from the age groups 20–39, 40–59 and 60 years and older (A). The sampling method is indicated in brackets: first morning urine void (mU), spot urine (sU) and 24-hours urine (24hU).

Supplementary Table S1 depicts all studies that measured the exposure biomarker Mono-benzyl phthalate (MBzP) of the parent compound BBzP and give the respective concentrations in µg/L. Overall, MBzP was measured in 27 different studies from 13 countries in urine samples collected between 2005 until 2019. Around half of the available data collections in each of the age groups 6–11 (10 out of 19), 20–39 (9 out of 20), and 40–59-year-olds (5 out of eight) are from DEMOCOPHES. With respect to 3–5-year-olds, a total of five studies from four countries contributed exposure data for MBzP, of which two collected spot urine samples (Children Cohort, CPH-MC) and three morning urine samples (GerES IV and V, CzechHBM-CE). All five studies showed a high prevalence of MBzP in the samples analyzed (9.5% and less < LOD/LOQ for Germany, Slovak, and Denmark, and 25% < LOD/LOQ for Czech Republic). In this age group, German children (GerES IV), sampled in 2005 showed the highest GM concentration with 21.8 µg/L, followed by Danish children sampled in 2006 (GM = 15.6 µg/L). MBzP levels were markedly lower in Slovak (GM = 2.3 µg/L), Czech (GM = 3.3 µg/L), and German (GM = 3.9 µg/L) children sampled in 2015–2016, 2016–2017, and 2014–2017, respectively. Children aged 6–11 years had the highest GM concentration in Denmark, with 50.8 µg/L (CPHPUB-Cross, 2006–2008), followed by Germany (GerES IV, 2005) with 2,5 times lower GM concentration (20.2 µg/L). Another Danish study, which collected spot urine samples between 2006–2007, had considerably lower MBzP levels (GM = 10.7 µg/L) and a higher number of samples below LOD (15.5% < LOD) compared to the CPHPUB-Cross and GerES IV studies (both 0% < LOD/LOQ). Around the year 2011, the highest concentrations were found in Sweden (GM = 19.7 µg/L), followed by Spain (GM = 12.5 µg/L), where both collected morning urine samples and lowest concentrations were observed in spot urine samples from Austrian children (GM = 3.0 µg/L). Likewise, low levels were observed in the most recent data sets, which are from Germany (GerES V, 2014–2017), Czech Republic (CzechHBM-CE, 2016–2017), and Slovakia (Children cohort, 2015–2016), with GMs of 3.3, 3.3, and 2.9 µg/L, respectively. Overall, seven studies from five countries measured MBzP in adolescents (12–19 years), of which three studies collected spot and four studies collected morning urine samples. Only 1% of the samples were below LOD/LOQ in all studies, but one (PBAT, Austria: 23.4% < LOD/LOQ). The highest GM concentrations were observed in Denmark (CPHPUB-Cross, 2006–2008, morning urine) with 45.0 µg/L, followed by Belgium (FLEHS2RefAdo, 2009–2009, morning urine) with 31.9 µg/L, Denmark (DYMS, 2007–2008, spot urine) with 31.8 µg/L, and Germany (GerES IV, 2005, morning urine) 14.3 µg/L. Data collections for young adults (20–39 years) are very heterogeneous in terms of collection methods, with ten data sets collecting morning urine samples, six 24-h-urine, and four spot urine. Thus, comparison of exposure levels across countries is difficult, except for the DEMOCOPHES data sets as protocols, and procedures were harmonized [17]. In regard to middle-aged adults (40–59 years), there were a total of eight data sets, five collected morning urine (all DEMOCOPHES studies) and three spot urine samples (Czech-HBM-AE, PBAT, IBS). In these three studies, a higher percentage of samples were below LOD/LOQ (77%, 52% and 14% < LOD/LOQ) compared to the DEMOCOPHES data sets (all 0% but one 9.6% < LOQ). Thus, no GM concentrations could be calculated for the Czech and the Austrian studies. Only one study for the subgroup of adults 60 years and older was available (Austria, PBAT). Here, 44.9% of the samples analyzed were below LOQ and GM concentrations were rather low (1.5 µg/L) compared to DEMOCOPHES data for the adult samples aged 40–59 years in Belgium, Denmark, Germany, Slovenia, and Spain sampled around the same time (2010–2012). The study from Israel (IBS, 2011) showed higher GM concentration (4.2 µg/L) than the Austrian seniors. Figure 1 shows the differences of internal BBzP exposure across all studies between regions and time patterns within regions. Especially for the regions which covered broad ranges of sampling years (North and West), the data indicate a decrease of MBzP. Interestingly, when sorting all studies by year of sampling (Supplementary Figure S1), boxplots suggest, on average, a rough decreasing time trend across all four European regions across all age groups. For completeness, the age gradient in MBzP can be found in Supplementary Figure S2.

**Figure 2:**
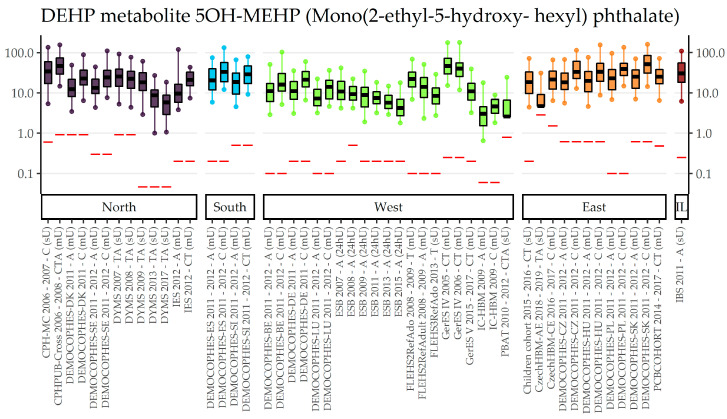
DEHP metabolite 5OH-MEHP concentration in µg/L in boxplots. The box is based on P25, P50, and P75, and the whiskers are based on P5 and P95. Boxplots are presented separately by regions (North = violet, South = blue, West = green, East = orange, Israel = dark red) within regions chronologically sorted by year of sampling. Red lines denote LOQ/LOD for the respective study. Since percentiles are only available and displayed when they are above LOQ/LOD, the lower side of boxplots might be truncated. The age categories are shown in capital letters: children of the age groups 3–5 and 6–11 (C), adolescents between 12 and 19 years (T) and adults from the age groups 20–39, 40–59 and 60 years and older (A). The sampling method is indicated in brackets: first morning urine void (mU), spot urine (sU) and 24-hours urine (24hU).

Supplementary Tables S2–S5 show the concentrations in µg/L for 4 DEHP metabolites: MEHP, 5OH-MEHP, 5oxo-MEHP, and Mono(2-ethyl-5-carboxy-pentyl) phthalate (5cx-MEPP). Generally, detection and quantification rates were very high, with small differences between the metabolites. As an example, concentrations of 5OH-MEHP stratified by region and sorted by year of sampling are shown in Figure 2, which indicates somewhat higher exposures in Eastern Europe and a stronger decreasing trend in Northern and Western Europe. Unfortunately, Southern European studies only cover the sampling years 2011 and 2012. Supplementary Figures S3 and S4 show the 5OH-MEHP results for the year of sampling and age gradient, respectively, showing a tendency to decreasing exposures over time and a tendency to higher exposures in younger groups. Figures for the other DEHP metabolites are shown in Supplementary Figures S5–S13, showing very similar characteristics compared to 5OH-MEHP. 

**Figure 3:**
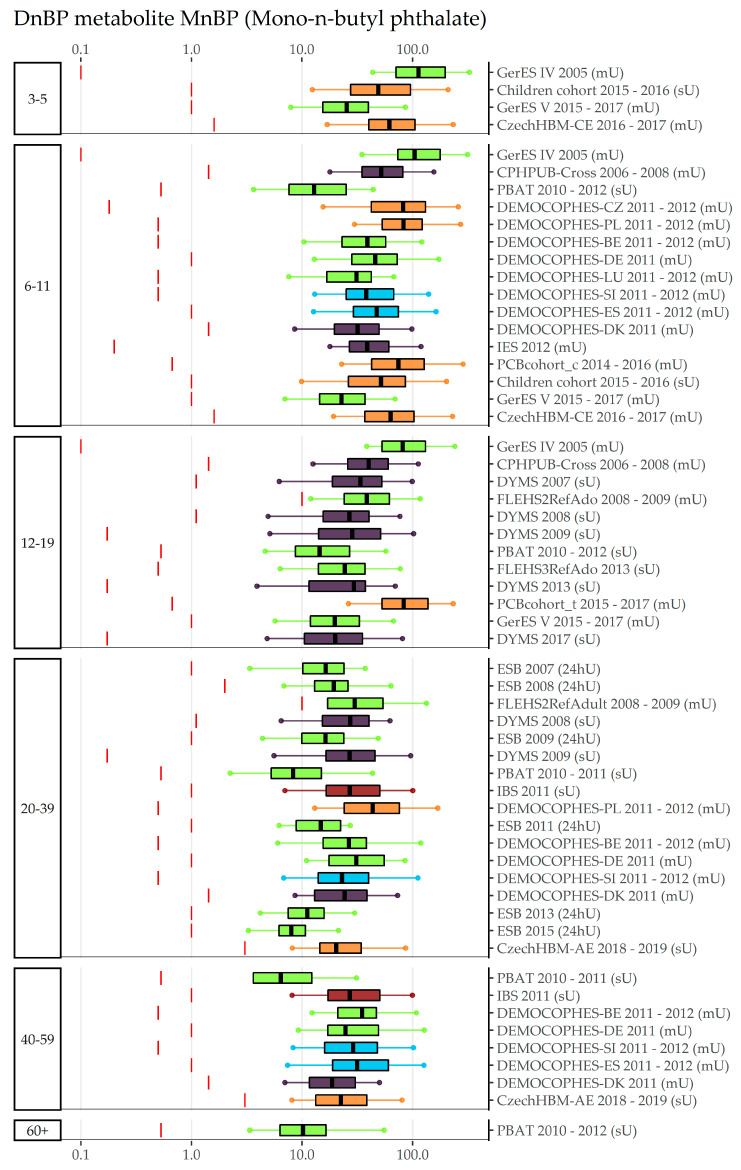
DnBP metabolite MnBP concentration in µg/L in boxplots. The box is based on P25, P50, and P75, and the whiskers are based on P5 and P95. Boxplots are presented separately by age groups; within age groups they are chronologically sorted by year of sampling. Colors of the boxplots refer to the data collection’s region (North = violet, South = blue, West = green, East = orange, Israel = dark red). Red lines denote LOQ/LOD for the respective study. Since percentiles are only available and displayed when they are above LOQ/LOD, the lower side of boxplots might be truncated.

**Figure 4:**
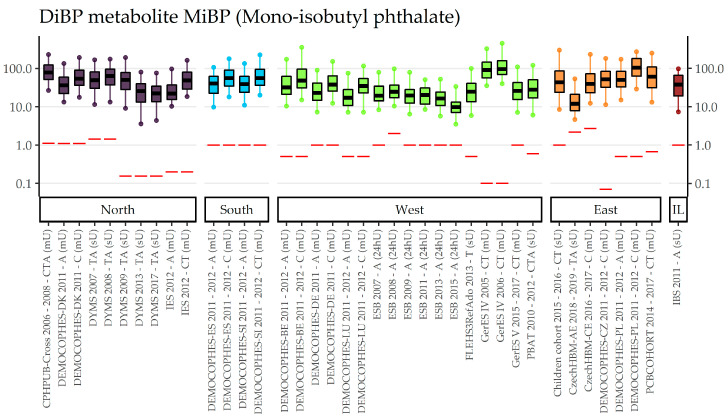
DiBP metabolite MiBP concentration in µg/L in boxplots. The box is based on P25, P50, and P75, and the whiskers are based on P5 and P95. Boxplots are presented separate by regions (North = violet, South = blue, West = green, East = orange, Israel = dark red), within regions chronologically sorted by year of sampling. Red lines denote LOQ/LOD for the respective study. Since percentiles are only available and displayed when they are above LOQ/LOD, the lower side of boxplots might be truncated. The age categories are shown in capital letters: children of the age groups 3–5 and 6–11 (C), adolescents between 12 and 19 years (T) and adults from the age groups 20–39, 40–59 and 60 years and older (A). The sampling method is indicated in brackets: first morning urine void (mU), spot urine (sU) and 24-hours urine (24hU).

**Figure 5:**
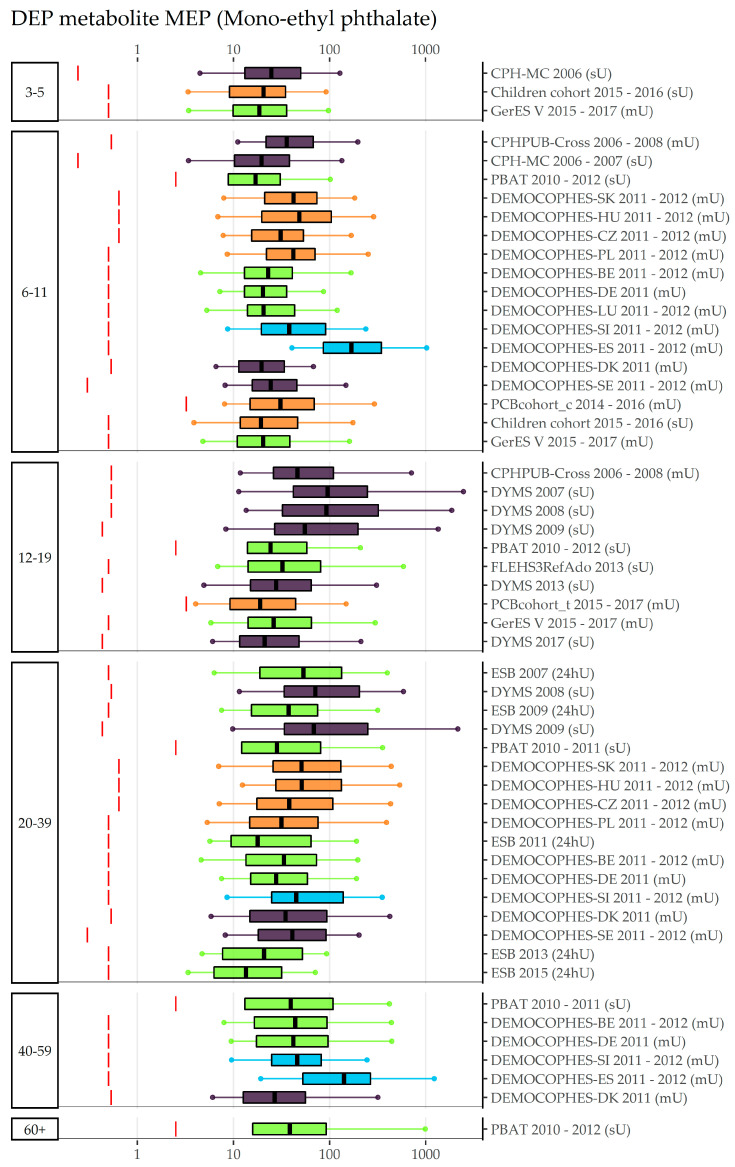
DEP metabolite MEP concentration in µg/L in boxplots. The box is based on P25, P50, and P75, and the whiskers are based on P5 and P95. Boxplots are presented separately by age groups; within age groups they are chronologically sorted by year of sampling. Colors of the boxplots refer to the data collection’s region (North = violet, South = blue, West = green, East = orange, Israel = dark red). Red lines denote LOQ/LOD for the respective study. Since percentiles are only available and displayed when they are above LOQ/LOD, the lower side of the boxplots might be truncated.

An overview of data collections, number of participants, detection rates, and exposure (percentiles, GMs and CIs) to phthalates DnBP, Di-isobutyl phthalate (DiBP), and DEP can be found in Supplementary Tables S6–S10. An age gradient to internal exposure to DnBP is suggested by Figure 3, which shows concentrations in Mono-n-butyl phthalate (MnBP) sorted by the participants’ age group: Children (3–5 and 6–11-year olds) have higher levels than adolescents (12–19-year olds), who in turn have higher levels than adults (20–39 years old). However, the time trend overrules the possible age gradient in these figures, therefore only data obtained in a very limited time period should be compared for the age gradient. To view the time pattern, concentrations sorted by region (and within region chronologically sorted by year of sampling) and year of sampling can be found in Supplementary Figures S14 and S15, respectively, indicating that participants from Eastern Europe are exposed highest to MnBP, but also have lower concentrations in recent years compared to older years. Figures for the DnBP metabolite OH-MnBP (Supplementary Figures S16–S18) indicate similar results to MnBP but with fewer data collections available. Regional differences are highlighted in the exposure to DiBP metabolite Mono-isobutyl phthalate (MiBP) (Figure 4), which shows lower concentrations in recent years compared to older years in all regions which cover more than two sampling years (North, West, East). Visualizations of the sampling year and age gradient for MiBP and Mono-hydroxy-iso-butylphthalate (OH-MiBP) can be found in Supplementary Figures S19–S23. The visualization of internal exposure to Mono-ethyl phthalate (MEP) indicates a less pronounced decreasing time trend (Supplementary Figures S24 and S25) and—in contrast to several other phthalates—no age differences (Figure 5).

### 3.2. DnOP, DnPeP, DCHP, DPHP, DMP, 3cx-MPP, DiNP, DiDP (Category B Phthalates)

Supplementary Tables S11–S26 show studies measuring exposure to Category B phthalates. Category B in HBM4EU denotes those phthalates with limited data availability at the start of the project in 2017. The phthalates Di-n-octyl phthalate (DnOP), Di-n-pentyl phthalate (DnPeP), Dicyclohexyl phthalate (DCHP), Dimethyl phthalate (DMP), DPHP, and the unspecific biomarker 3-Carboxyl-mono-propyl phthalate (3cx-MPP) fall under this category. Due to very low detection or quantification rates (0 to 24% of values at or above LOD/LOQ) for DnOP, DnPeP, and DCHP metabolites across national HBM studies and age groups, GM concentrations were not calculated, higher percentiles (e.g., P75, P95) are only sometimes available, and internal exposure between European data collections cannot be compared (data not shown). For the DMP biomarker mono-methyl phthalate (MMP) there was a considerable variation in detection and quantification rates in the 20 data collections (range: 1.4 to 89% of values below LOD/LOQ). The same was applied to the 11 studies which have analyzed 3cx-MPP (range: 0 to 32.1% below LOD/LOQ. Since there were, at most, quantified concentrations from three data collections per age group (except for the yearly time trend observations for ESB in adults), we refer to Supplementary Tables S11 and S12, and Supplementary Figures S26–S31. Since the secondary metabolites of DPHP (mono-(propyl-6-carboxyhexyl) phthalate (MPHP), mono-(propyl-6-hydroxyheptyl) phthalate (OH-MPHP), and mono-(propyl-6-oxo-heptyl)-phthalate) (oxo-MPHP)) were only measured in German data GerES V, unweighted, and ESB, we refrained from reporting these and refer to national published reports [51,52]. 

**Figure 6:**
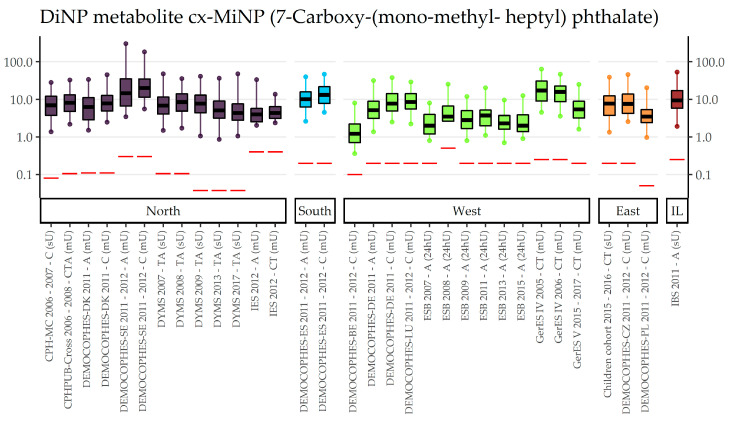
DiNP metabolite cx-MiNP concentration in µg/L in boxplots. The box is based on P25, P50, and P75, and the whiskers are based on P5 and P95. Boxplots are presented separate by regions (North = violet, South = blue, West = green, East = orange, Israel = dark red); within regions they are chronologically sorted by year of sampling. Red lines denote LOQ/LOD for the respective study. Since percentiles are only available and displayed when they are above LOQ/LOD, the lower side of boxplots might be truncated. The age categories are shown in capital letters: children of the age groups 3–5 and 6–11 (C), adolescents between 12 and 19 years (T) and adults from the age groups 20–39, 40–59 and 60 years and older (A). The sampling method is indicated in brackets: first morning urine void (mU), spot urine (sU) and 24-h urine (24hU).

**Figure 7:**
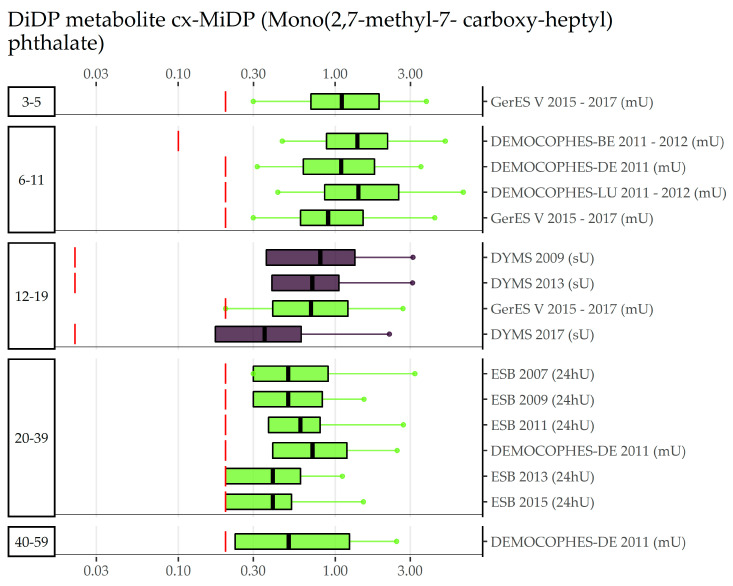
DiDP metabolite cx-MiDP concentration in µg/L in boxplots. The box is based on P25, P50, and P75, and the whiskers are based on P5 and P95. Boxplots are presented separately by age groups; within age groups they are chronologically sorted by year of sampling. Colors of the boxplots refer to the data collection’s region (North = violet, South = blue, West = green, East = orange, Israel = dark red). Red lines denote LOQ/LOD for the respective study. Since percentiles are only available and displayed when they are above LOQ/LOD, the lower side of boxplots might be truncated.

Compiled Di-isononyl phthalate (DiNP) data can be found in Supplementary Tables S13–S16 separately for secondary metabolites 7-Carboxy-(mono-methyl-heptyl) phthalate (cx-MiNP), 7-OH-(Mono-methyl-octyl) phthalate (OH-MiNP), and 7-Oxo-(Mono-methyl-octyl) phthalate (oxo-MiNP). Since metabolite Mono-methyl-octyl phthalate (MiNP) was only measured in Danish data collections (DEMOCOPHES, CPH-MC, and CPHPUB-Cross), no European comparisons can be made. All concentrations were quantified with a fraction of values below LOD/LOQ at most 7% for cx-MiNP and 14% for oxo-MiNP. In OH-MiNP, all but one Polish study ( with 62% of values below LOQ) had rather high detection or quantification rates (range = 0 to 3.5%). In the age group of 3–5-year olds, four data collections were available. Around 2005, German children had considerably higher morning urine concentrations in cx-MiNP than Danish children in spot urine in 2006 (GM 17.7 µg/L vs. 7.2 µg/L). A decade later, German and Slovakian children have very similar levels to each other (7 µg/L in morning and 6 µg/L spot urine, respectively) and levels similar to Danish children in 2006. A similar order of exposure can be found for OH-MiNP and oxo-MiNP which, however, was only measured in the German and Slovakian studies. Details of the age groups in which DEMOCOPHES data were available (6–11, 20–39, 40–59) can be found in the Supplementary Tables S13–S15. To sum up, half or more than half of studies were DEMECHOPHES studies and concentrations of other studies (e.g., Israeli IBS and Norwegian Indoor Environment Study (IES)) in a similar time period fit within the range of DiNP exposures of different DEMOCOPHES studies. For 12–19-year olds, two Danish (2006–2008 and 2007–2009) and two German data collections (around 2005 and 2014–2017) are available. The earliest study (GerES IV, 2005, morning urine) has the highest concentrations for all three secondary metabolites (10.8 µg/L for cx-MiNP, 9.2 µg/L for OH-MiNP and 4.4 µg/L for oxo-MiNP), followed by very similar GM levels of both Danish studies (CPHPUB-Cross, 2006–2008, morning urine; and DYMS, 2007–2009, spot urine) around 7 µg/L in cx-MiNP, 4.5 µg/L in OH-MiNP, and 2.5 µg/L in oxo-MiNP. Sampled approximately eight years later (2014–2017), compared to the Danish studies, the German study GerES V had relatively lower levels in cx-MiNP (4.7 µg/L), higher levels in OH-MiNP (5.6 µg/L), and similar levels in oxo-MiNP (2.3 µg/L); compared to the previous German cycle (GerES IV), all concentrations were lower in the recent cycle. As an example, Figure 6 shows that, compared to Category A substances, fewer data on cx-MiNP, a biomarker of DiNP, are available. No exposure data were available for adults aged 60 and older. The visualization of sampling year and age differences and the other metabolites of DiNP are shown in Supplementary Figures S32–S39. Results for Category B phthalate Di-isodecyl phthalate (DiDP) can be found in Supplementary Tables S17–S19. Although there are fewer data sets for the biomarker Mono(2,7-methyl-7-carboxy-heptyl) phthalate (cx-MiDP), a difference between age groups is visible for the available data from Western and Northern Europe (Figure 7): children (3–5 and 6–11 years old) have lower concentrations than adolescents (12–19 years old), who in turn have smaller concentrations than 20 to 39-year-old adults. Figures for region and sampling year differences and for other DiDP metabolites are shown in Supplementary Figures S40–S47.

Since for the phthalate substitute DINCH (and DEHTP (di-(2-ethylhexyl) terephthalate)) data were only available for one Danish and two German studies, we refrained from reporting results and refer to Supplementary Tables S20–S26 and Supplementary Figures S48–S56, and Vogel, Frederiksen, Lange, Jorgensen, Koch, Weber, Andersson, and Kolossa-Gehring [15].

Overall, the assessment of Category B phthalates strongly suggests that data for these substances has still been rather scarce at the onset of HBM4EU to be used for the investigation of internal exposure and of differences between the exposure by age groups, sampling years, or regions.

## 4. Discussion

HBM4EU provided the possibility to describe and examine HBM-derived internal exposure to phthalates and DINCH on a European scale, in our case by using existing HBM data that was aggregated and evaluated by a harmonized approach within HBM4EU. This approach must not be confused with the forward-oriented establishment of a pan-European HBM study within HBM4EU with a harmonized sampling procedure and utmost comparability of analytical results guaranteed through an accompanying external quality assessment scheme (HBM4EU Aligned Studies) [53,54]. 

We unified existing HBM data with respect to the substitution of values below LOQ, between LOD and LOQ, and below LOD (based on normal and lognormal distribution assumptions), predefined stratification criteria (e.g., age groups), and calculation of statistics (percentiles, means, GM, and their CIs) to describe exposure to 12 phthalates from Category A and B and DINCH in the general population. Overall, we used 29 aggregated data collections from 14 countries from all four European regions (north, east south, west) and Israel, targeting individuals from the age of three to sixty and older. 

With respect to Category A substances (those with an already good database), our observations of generally decreasing time patterns for DEHP, DiBP, DnBP, and BBzP showed higher exposures in the young age groups compared to the elders (except DEP), and some regions had higher exposures than others for some phthalates [17,40,55], which is in good agreement with the results of the individual longitudinal time trend studies [15] and cross-sectional studies that were part of the HBM4EU Aligned Studies in children and teenagers [16,56]. Although exposure to phthalates in some European countries has been described in a harmonized manner in COPHES/DEMOCOPHES [17], this project only focused on the groups of 6–11-year-old children and their mothers, and reflects exposure from a decade ago (2011–2012). While the design could robustly identify differences between the participating countries, and associations between mothers and children, other important aspects remained un-investigated. The rapid change in phthalate exposure due to regulatory measures and market changes could not be investigated. Additionally, hints to pronounced regional differences deserve a more in-depth investigation. The phthalate substitute DINCH was just beginning to being used as a replacement to DEHP and DiNP, and thus was not measured at all. Our analyses revealed that regional differences might be more substantial than previously assumed based on DEMOCOPHES data. Additionally, regional and country to country differences could be substantial. In part, this might be explained by different products or use patterns in different countries/regions. Another reason for these differences might also be found in the different study designs, different sampling strategies, and different quantitative results caused by different analytical methodologies not checked for comparability, so one has to be careful with interpreting these results due to heterogeneity between the studies. Such issues can only be tackled by the harmonized sampling and analytical approach developed in HBM4EU in parallel to our approach. 

For some Category B substances, with available datasets (e.g., DnOP, DnPeP, and DCHP), very low detection or quantification rates (0 to 24% values at or above LOD/LOQ) have been found, and low concentrations suggest either low production and/or low application in consumer products. This is confirmed for DnOP, as it is very likely that no EU market exists [57]. DnPeP and DCHP are produced in very low quantities (10–1000 t/a and 100–1000 t/a) [58,59]. For DINCH, a trend to increased GM concentrations over time in both German and Danish samples was seen [15]. This, together with the increasing number of samples in which DINCH metabolites are being detected, suggests that DINCH is indeed increasingly used as substitute plasticizer in a variety of consumer products. 

Harmonizing data collections such as the HBM4EU Aligned Studies [53,54,56] enable a more straightforward description and interpretation of HBM data. For example, comparable HBM data due to QA/QC program can be generated. Since a harmonization of already collected and analyzed HBM data can only enhance comparison of features affecting data preparation and statistical analyses, we still had to deal with a great deal of heterogeneity due to differences arising from the study design and data acquisition phases. Grouping data collections according to similar features (e.g., age group, year of sampling, urine collection type) helped to overcome this heterogeneity to some degree, only allowing comparisons and conclusions—if any—on strongly selected data portions. 

While using data on 29 data collections for the general population is a great success, several data collections with phthalates or DINCH data were not accessible in a harmonized format. Other fruitful European HBM studies and networks (e.g., HELIX) [60] with phthalate data are available. However, harmonizing data is a resource-intensive and time-consuming process. Therefore, additionally, with the low expected benefit for rather older data, the number of actual HBM programs targeting plasticizers since 2005 is expected to be higher than that collected within HBM4EU. In addition, our data collection did not include phthalates with Repro. 2 “Notified classification and labelling according to CLP criteria” (H361: “Suspected of damaging fertility or the unborn child”) [61]. This category, for example, includes Diheptyl phthalate, Decyl octyl phthalate, and Dipropyl phthalate. 

Additional actions can be applied in the future to enhance comparability between data collections. For example, in the data preparation step, an approximation estimating 24-h excretion for spot and morning urine of the pollutant can reduce level discrepancies caused by different urine collection methods (sU, mU, 24hU; [62]). However, ideally standardized procedures would be already implemented at earlier steps of data collection and acquisition. In addition, where possible, raw and adjusted concentrations should be reported (e.g., standardized by creatinine or normalized specific gravity). In our data, however, whether and which adjustment method was available varied widely, which did not allow for a comprehensive comparison within the EU. Therefore, this study was limited to the analyses of the raw urinary data. Furthermore, future studies could report phthalate concentrations of European samples stratified by or controlled for important factors or exposure determinants (e.g., sex, degree of occupational exposure, degree of urbanization). Within HBM4EU, the HBM4EU Aligned Studies offered a possibility to collect phthalates and DINCH samples via a harmonized manner for selected population groups by suggesting inclusion and exclusion criteria for data acquisition, defining the same number of participants for all studies, providing harmonized questionnaires, selecting specific metabolites, and offering quality assurances of laboratories used to analyze biomarkers among other means [53,54,56]. For the majority of studies, post-harmonization of the questionnaire data was still needed and was sometimes limited, as the studies were already ongoing. Future efforts for the harmonization of European HBM studies (Partnership of the assessment of Risk of Chemicals, PARC) will hopefully initiate the harmonization at earlier steps of HBM data acquisition, both at the pre-analytical and analytical stages. A potential shortcoming that could not be addressed in our study is the analytical comparability of results. While analytical comparability was quality-assured in COPHES/DEMOCOPHES, it cannot be necessarily assumed for all of the other included studies for all reported biomarkers. For this, successful participation in a harmonized external quality assessment scheme during the measurement of the respective study samples is essential. Nevertheless, the comparison with studies outside of HBM4EU (or PARC) still faces the well-known difficulties between HBM programs when examining exposure to chemicals such as phthalates and DINCH.

## 5. Conclusions

The HBM4EU initiative allows for gathering of existing HBM data from participating countries to describe internal exposure to phthalates and DINCH on a European level in a harmonized and comparable way. With this, we provide the best possible overview of exposures to phthalates covering the years 2005 to 2019, which have a high regulatory relevance in the EU (BBzP, DEHP, DnBP, DiBP, DiDP, DiNP), and also summarize data on less investigated phthalates (DnOP, DnPeP, DCHP, DPHP, DMP). This closes a major gap in the reporting of phthalate exposures in Europe. Despite their decreasing concentrations, the SVHC phthalates need continued monitoring since they still have high detection frequencies and can have a cumulative effect. In addition, DINCH data have been very fragmented in this data collection, and the rising concentrations trend also needs further surveillance. The limitations are that, despite all the harmonization performed during data harmonization and aggregation, we still observed a lot of heterogeneity resulting from previous phases of the studies (e.g., data acquisition). Comparisons of exposure to pollutants between countries call for an HBM approach, with harmonization already at the stage of study design and data acquisition at a European and potentially international level. 

## Figures and Tables

**Table 1:** Harmonized, aggregated existing HBM studies collected within HBM4EU with phthalate and or DINCH measured in urine.

EU Region	Country	Data Collection	Age Groups with Phthalates Data	(Range of) Sampling Years	Publications
East	Czech Republic	CzechHBM-CE	Children	2016–2017	[19,20]
	Czech Republic	DEMOCOPHES-CZ	Children, adults	2011–2012	[21]
	Czech Republic	CzechHBM-AE	Adults	2018–2019	[22]
	Hungary	DEMOCOPHES-HU	Children, adults	2011–2012	[21]
	Poland	DEMOCOPHES-PL	Children, adults	2011–2012	[17]
	Slovakia	PCBcohort	Children, teenagers	2014–2017	[23]
	Slovakia	Children cohort	Children	2015–2016	-
	Slovakia	DEMOCOPHES-SK	Children, adults	2011–2012	[21]
South	Spain	DEMOCOPHES-ES	Children, adults	2011–2012	[24]
	Slovenia	DEMOCOPHES-SI	Children, adults	2011–2012	[25]
North	Denmark	DEMOCOPHES-DK	Children, adults	2011	[17,26]
	Denmark	CPH-MC	Children	2006	[27]
	Denmark	CPHPUB-Cross	Children, teenagers	2006–2008	[28]
	Denmark	DYMS	Teenagers, adults	2007–2017	[29,30]
	Norway	IES	Children, adults	2012	[31]
	Sweden	DEMOCOPHES-SE	Children, adults	2011–2012	[32]
West	Austria	PBAT	Children, teenagers, adults, elderly	2010–2012	[33]
	Austria	IC-HBM	Children, adults	2009	[34]
	Belgium	DEMOCOPHES-BE	Children, adults	2011–2012	[35,36]
	Belgium	FLEHS2RefAdo	Teenagers	2008–2009	[37]
	Belgium	FLEHS2RefAdult	Adults	2008–2009	[38]
	Belgium	FLEHS3RefAdo	Teenagers	2013	[39]
	Germany	GerES IV (unweighted)	Children, teenagers	2003–2006	[7]
	Germany	GerES V (unweighted)	Children, teenagers	2015–2017	[40]
	Germany	DEMOCOPHES-DE	Children, adults	2011	[41]
	Germany	ESB	Adults	2007–2019	[42,43]
	Luxemburg	DEMOCOPHES-LU	Children, adults	2011–2012	[35]
Other	Israel	IBS	Adults, elderly	2011	[44]

Notes. Data collections: CPH-MC—COPENHAGEN Mother Child Cohort, CPHPUB-Cross—COPENHAGEN Puberty Study, DYMS—Danish Young Men Study, IES—Indoor Environment Study, PBAT—HBM of phthalates and BPA in the Austrian general population, IC_HBM—HBM of industrial chemicals, FLEHS—FLemish Environment and Health Study, GerES—German Environmental Survey, ESB—Environmental Specimen Bank, IBS—Israel Biomonitoring Study. Metadata can be found in IPCHEM (https://ipchem.jrc.ec.europa.eu/#showmetadata/HBM4EUAGGREGATED, accessed on 20 December 2022).

**Table 2:** Phthalate compounds and their measured metabolites.

Phthalate Diester	Acronym	CAS-No.	Metabolite of Diester	Acronym of Metabolite
HBM4EU Category A ^1^				
**Butyl benzyl phthalate**	BBzP	85-68-7	Mono-benzyl phthalate	**MBzP**
**Di(2-ethylhexyl) phthalate**	DEHP	117-81-7	Mono(2-ethylhexyl) phthalate	**MEHP**
			Mono(2-ethyl-5-carboxy-pentyl) phthalate	**5cx-MEPP**
			Mono(2-ethyl-5-hydroxy-hexyl) phthalate	**5OH-MEHP**
			Mono(2-ethyl-5-oxohexyl) phthalate	**5oxo-MEHP**
**Di-n-butyl phthalate**	DnBP	84-74-2	Mono-n-butyl phthalate	**MnBP**
			Mono-hydroxy-n-butyl phthalate	**OH-MnBP**
**Di-isobutyl phthalate**	DiBP	84-69-5	Mono-isobutyl phthalate	**MiBP**
			Mono-hydroxy-iso-butylphthalate	**OH-MiBP**
**Diethyl phthalate**	DEP	84-66-2	Mono-ethyl phthalate	**MEP**
HBM4EU Category B ^2^				
**Di-isodecyl phthalate (and ^3^ DPHP)**	DiDP	26761-40-0	Mono-isodecyl phthalate	**MiDP**
			Mono(2,7-methyl-7-carboxy-heptyl) phthalate	**cx-MiDP**
			6-OH-Mono-propyl-heptyl phthalate	**OH-MiDP**
			6-Oxo-Mono-propyl-heptyl phthalate	**oxo-MiDP**
**Di-isononyl phthalate**	DiNP	28553-12-0	Mono-methyl-octyl phthalate	**MiNP**
			7-Carboxy-(mono-methyl-heptyl) phthalate	**cx-MiNP**
			7-OH-(Mono-methyl-octyl) phthalate	**OH-MiNP**
			7-Oxo-(Mono-methyl-octyl) phthalate	**oxo-MiNP**
**Bis(2-propylheptyl) phthalate**	DPHP	53306-54-0	mono-(propyl-6-carboxyhexyl) phthalate	**cx-MPHP**
			mono-(propyl-6-hydroxyheptyl) phthalate	**OH-MPHP**
			mono-(propyl-6-oxo-heptyl)-phthalate	**oxo-MPHP**
**Dicyclohexyl phthalate**	DCHP	84-61-7	Mono-cyclo-hexyl phthalate	**MCHP**
**Dimethyl phthalate**	DMP	131-11-3	Mono-methyl phthalate	**MMP**
**Di-n-pentyl phthalate**	DnPeP	131-18-0	Mono-n-pentyl phthalate	**MnPeP**
**Di-n-octyl phthalate**	DnOP	117-84-0	Mono-n-octyl phthalate	**MnOP**
**Various phthalates**			3-Carboxyl-mono-propyl phthalate	**3cx-MPP**

^1^ HBM4EU assigned Category A to phthalates/biomarkers for which reliable and broad HBM data existed [9]. ^2^ HBM4EU assigned Category B to phthalates/biomarkers for which fragmented HBM data existed [9]. ^3^ The analytical method applied cannot distinguish between DiDP and DPHP metabolites.

## Data Availability

Aggregated data used for this study can be found in IPCHEM: https://ipchem.jrc.ec.europa.eu/#showmetadata/HBM4EUAGGREGATED, accessed on 20 December 2022. Meanwhile a newer version of data or additional data collections might be available.

## Supplementary Materials

The supporting information can be downloaded at: https://www.mdpi.com/article/10.3390/toxics11030241/s1. Figure S1. BBzP metabolite MBzP (Mono-benzyl phthalate) concentration in µg/L stratified by sampling year in boxplots; Figure S2. BBzP metabolite MBzP (Mono-benzyl phthalate) concentration in µg/L stratified by age groups in boxplots; Figure S3. DEHP metabolite 5OH-MEHP (Mono(2-ethyl-5-hydroxy- hexyl) phthalate) concentration in µg/L stratified by sampling year in boxplots; Figure S4. DEHP metabolite 5OH-MEHP (Mono(2-ethyl-5-hydroxy- hexyl) phthalate) concentration in µg/L stratified by age groups in boxplots; Figure S5. DEHP metabolite 5oxo-MEHP (Mono(2-ethyl-5-oxo-hexyl) phthalate) concentration in µg/L stratified by region in boxplots; Figure S6. DEHP metabolite 5oxo-MEHP (Mono(2-ethyl-5-oxo-hexyl) phthalate) concentration in µg/L stratified by sampling year in boxplots; Figure S7. DEHP metabolite 5oxo-MEHP (Mono(2-ethyl-5-oxo-hexyl) phthalate) concentration in µg/L stratified by age groups in boxplots; Figure S8. DEHP metabolite 5cx-MEPP (Mono(2-ethyl-5-carboxy- pentyl) phthalate) concentration in µg/L stratified by region in boxplots; Figure S9. DEHP metabolite 5cx-MEPP (Mono(2-ethyl-5-carboxy- pentyl) phthalate) concentration in µg/L stratified by sampling year in boxplots; Figure S10. DEHP metabolite 5cx-MEPP (Mono(2-ethyl-5-carboxy- pentyl) phthalate) concentration in µg/L stratified by age groups in boxplots; Figure S11. DEHP metabolite MEHP (Mono(2-ethylhexyl) phthalate) concentration in µg/L stratified by region in boxplots; Figure S12. DEHP metabolite MEHP (Mono(2-ethylhexyl) phthalate) concentration in µg/L stratified by sampling year in boxplots; Figure S13. DEHP metabolite MEHP (Mono(2-ethylhexyl) phthalate) concentration in µg/L stratified by age groups in boxplots; Figure S14. DnBP metabolite MnBP (Mono-n-butyl phthalate) concentration in µg/L stratified by region in boxplots; Figure S15. DnBP metabolite MnBP (Mono-n-butyl phthalate) concentration in µg/L stratified by sampling year in boxplots; Figure S16. DnBP metabolite OH-MnBP (3-OH-Mono-n-butyl phthalate) concentration in µg/L stratified by region in boxplots; Figure S17. DnBP metabolite OH-MnBP (3-OH-Mono-n-butyl phthalate) concentration in µg/L stratified by sampling year in boxplots; Figure S18. DnBP metabolite OH-MnBP (3-OH-Mono-n-butyl phthalate) concentration in µg/L stratified by age groups in boxplots; Figure S19. DiBP metabolite MiBP (Mono-isobutyl phthalate) concentration in µg/L stratified by sampling year in boxplots; Figure S20. DiBP metabolite MiBP (Mono-isobutyl phthalate) concentration in µg/L stratified by age groups in boxplots; Figure S21. DiBP metabolite OH-MiBP (2-OH-Mono-iso- butylphthalate) concentration in µg/L stratified by region in boxplots; Figure S22. DiBP metabolite OH-MiBP (2-OH-Mono-iso- butylphthalate) concentration in µg/L stratified by sampling year in boxplots; Figure S23. DiBP metabolite OH-MiBP (2-OH-Mono-iso- butylphthalate) concentration in µg/L stratified by age groups in boxplots; Figure S24. DEP metabolite MEP (Mono-ethyl phthalate) concentration in µg/L stratified by region in boxplots; Figure S25. DEP metabolite MEP (Mono-ethyl phthalate) concentration in µg/L stratified by sampling year in boxplots; Figure S26. DMP metabolite MMP (Mono-methyl phthalate) concentration in µg/L stratified by region in boxplots; Figure S27. DMP metabolite MMP (Mono-methyl phthalate) concentration in µg/L stratified by sampling year in boxplots; Figure S28. DMP metabolite MMP (Mono-methyl phthalate) concentration in µg/L stratified by age groups in boxplots; Figure S29. Metabolite 3cx-MPP (3-carboxyl-mono-propyl phthalate) concentration in µg/L stratified by region in boxplots; Figure S30. Metabolite 3cx-MPP (3-carboxyl-mono-propyl phthalate) concentration in µg/L stratified by sampling year in boxplots; Figure S31. Metabolite 3cx-MPP (3-carboxyl-mono-propyl phthalate) concentration in µg/L stratified by age groups in boxplots; Figure S32. DiNP metabolite cx-MiNP (7-Carboxy-(mono-methyl- heptyl) phthalate) concentration in µg/L stratified by sampling year in boxplots; Figure S33. DiNP metabolite cx-MiNP (7-Carboxy-(mono-methyl- heptyl) phthalate) concentration in µg/L stratified by age groups in boxplots; Figure S34. DiNP metabolite OH-MiNP (7-OH-(Mono-methyl-octyl) phthalate) concentration in µg/L stratified by region in boxplots; Figure S35. DiNP metabolite OH-MiNP (7-OH-(Mono-methyl-octyl) phthalate) concentration in µg/L stratified by sampling year in boxplots; Figure S36. DiNP metabolite OH-MiNP (7-OH-(Mono-methyl-octyl) phthalate) concentration in µg/L stratified by age groups in boxplots; Figure S37. DiNP metabolite oxo-MiNP (7-Oxo-(Mono-methyl-octyl) phthalate) concentration in µg/L stratified by region in boxplots; Figure S38. DiNP metabolite oxo-MiNP (7-Oxo-(Mono-methyl-octyl) phthalate) concentration in µg/L stratified by sampling year in boxplots; Figure S39. DiNP metabolite oxo-MiNP (7-Oxo-(Mono-methyl-octyl) phthalate) concentration in µg/L stratified by age groups in boxplots; Figure S40. DiDP metabolite cx-MiDP (Mono(2,7-methyl-7- carboxy-heptyl) phthalate) concentration in µg/L stratified by region in boxplots; Figure S41. DiDP metabolite cx-MiDP (Mono(2,7-methyl-7- carboxy-heptyl) phthalate) concentration in µg/L stratified by sampling year in boxplots; Figure S42. DiDP metabolite OH-MiDP (6-OH-Mono-propyl-heptyl phthalate) concentration in µg/L stratified by region in boxplots; Figure S43. DiDP metabolite OH-MiDP (6-OH-Mono-propyl-heptyl phthalate) concentration in µg/L stratified by sampling year in boxplots; Figure S44. DiDP metabolite OH-MiDP (6-OH-Mono-propyl-heptyl phthalate) concentration in µg/L stratified by age groups in boxplots; Figure S45. DiDP metabolite oxo-MiDP (6-Oxo-Mono-propyl-heptyl phthalate) concentration in µg/L stratified by region in boxplots; Figure S46. DiDP metabolite oxo-MiDP (6-Oxo-Mono-propyl-heptyl phthalate) concentration in µg/L stratified by sampling year in boxplots; Figure S47. DiDP metabolite oxo-MiDP (6-Oxo-Mono-propyl-heptyl phthalate) concentration in µg/L stratified by age groups in boxplots; Figure S48. DINCH metabolite cx-MINCH (cyclohexane-1,2- dicarboxylate-mono-(7- carboxylate-4- methyl)heptyl ester) concentration in µg/L stratified by region in boxplots; Figure S49. DINCH metabolite cx-MINCH (cyclohexane-1,2- dicarboxylate-mono-(7- carboxylate-4- methyl)heptyl ester) concentration in µg/L stratified by sampling year in boxplots; Figure S50. DINCH metabolite cx-MINCH (cyclohexane-1,2- dicarboxylate-mono-(7- carboxylate-4- methyl)heptyl ester) concentration in µg/L stratified by age groups in boxplots; Figure S51. DINCH metabolite OH-MINCH (cyclohexane-1,2- dicarboxylate-mono-(7- hydroxy-4-methyl)octyl ester) concentration in µg/L stratified by region in boxplots; Figure S52. DINCH metabolite OH-MINCH (cyclohexane-1,2- dicarboxylate-mono-(7- hydroxy-4-methyl)octyl ester) concentration in µg/L stratified by sampling year in boxplots; Figure S53. DINCH metabolite OH-MINCH (cyclohexane-1,2- dicarboxylate-mono-(7- hydroxy-4-methyl)octyl ester) concentration in µg/L stratified by age groups in boxplots; Figure S54. DINCH metabolite oxo-MINCH (cyclohexane-1,2- dicarboxylate-mono-(7-oxo- 4-methyl)octyl ester) concentration in µg/L stratified by region in boxplots; Figure S55. DINCH metabolite oxo-MINCH (cyclohexane-1,2- dicarboxylate-mono-(7-oxo- 4-methyl)octyl ester) concentration in µg/L stratified by sampling year in boxplots; Figure S56. DINCH metabolite oxo-MINCH (cyclohexane-1,2- dicarboxylate-mono-(7-oxo- 4-methyl)octyl ester) concentration in µg/L stratified by age groups in boxplots; Table S1: BBzP metabolite MBzP (Mono-benzyl phthalate) in microg/L; Table S2: DEHP metabolite MEHP (Mono(2-ethylhexyl) phthalate) in microg/L; Table S3: DEHP metabolite 5OH-MEHP (Mono(2-ethyl-5-hydroxy- hexyl) phthalate) in microg/L; Table S4: DEHP metabolite 5oxo-MEHP (Mono(2-ethyl-5-oxo-hexyl) phthalate) in microg/L; Table S5: DEHP metabolite 5cx-MEPP (Mono(2-ethyl-5-carboxy- pentyl) phthalate) in microg/L; Table S6: DnBP metabolite MnBP (Mono-n-butyl phthalate) in microg/L; Table S7: DnBP metabolite OH-MnBP (3-OH-Mono-n-butyl phthalate) in microg/L; Table S8: DiBP metabolite MiBP (Mono-isobutyl phthalate) in microg/L; Table S9: DiBP metabolite OH-MiBP (2-OH-Mono-iso- butylphthalate) in microg/L; Table S10: DEP metabolite MEP (Mono-ethyl phthalate) in microg/L; Table S11: DMP metabolite MMP (Mono-methyl phthalate) in microg/L; Table S12: Metabolite 3cx-MPP (3-carboxyl-mono-propyl phthalate) in microg/L; Table S13: DiNP metabolite cx-MiNP (7-Carboxy-(mono-methyl- heptyl) phthalate) in microg/L; Table S14: DiNP metabolite OH-MiNP (7-OH-(Mono-methyl-octyl) phthalate) in microg/L; Table S15: DiNP metabolite oxo-MiNP (7-Oxo-(Mono-methyl-octyl) phthalate) in microg/L; Table S16: DiNP metabolite MiNP (Mono-methyl-octyl phthalate) in microg/L; Table S17: DiDP metabolite cx-MiDP (Mono(2,7-methyl-7- carboxy-heptyl) phthalate) in microg/L; Table S18: DiDP metabolite OH-MiDP (6-OH-Mono-propyl-heptyl phthalate) in microg/L; Table S19: DiDP metabolite oxo-MiDP (6-Oxo-Mono-propyl-heptyl phthalate) in microg/L; Table S20: DINCH metabolite cx-MINCH (cyclohexane-1,2- dicarboxylate-mono-(7- carboxylate-4- methyl)heptyl ester) in microg/L; Table S21: DINCH metabolite OH-MINCH (cyclohexane-1,2- dicarboxylate-mono-(7- hydroxy-4-methyl)octyl ester) in microg/L; Table S22: DINCH metabolite oxo-MINCH (cyclohexane-1,2- dicarboxylate-mono-(7-oxo- 4-methyl)octyl ester) in microg/L; Table S23: DEHTP metabolite 2cx-MMHTP (1-mono-(2-carboxyl-methyl-hexyl) benzene-1,4-dicarboxylate) in microg/L; Table S24: DEHTP metabolite 5cx-MEPTP (1-mono-(2-ethyl-5-carboxyl-pentyl) benzene-1,4-dicarboxylate) in microg/L; Table S25: DEHTP metabolite 5OH-MEHTP (1-mono-(2-ethyl-5-hydroxy-hexyl) benzene-1,4-dicarboxylate) in microg/L; Table S26: DEHTP metabolite 5oxo-MEHTP (1-mono-(2-ethyl-5-oxo-hexyl) benzene-1,4-dicarboxylate) in microg/L.

## Appendix A

**Table A1:** Overview of measured exposure biomarkers of 12 phthalate parent compounds.

Phthalate Diester	Acronym	Classification as Reproductive Toxicant According to Regulation (EC) No 1272/2008	Identification as SVHC under REACH	REACH	CoRAP
Di(2-ethylhexyl) phthalate	DEHP	Repr. 1B (2001)	Toxic for reproduction (2008); Endocrine disrupting properties ENV, HH (2014, 2017)	Subject to Authorization since 2015 (Annex XVI); Restriction in toys, childcare (2005) and any plasticized articles since 2020 (Annex XVII)	
Di-n-butyl phthalate	DnBP	Repr. 1B (2001)	Toxic for reproduction (2008); Endocrine disrupting properties HH (2017)	Restriction in toys, childcare (2005) and any plasticized articles since 2020 (Annex XVII)	
Di-isobutyl phthalate	DiBP	Repr. 1B (2009)	Toxic for reproduction (2008); Endocrine disrupting properties HH (2017)	Subject to Authorization since 2015 (Annex XVI); Restriction in toys, childcare (2005) and any plasticized articles since 2020	
Butyl benzyl phthalate	BBzP	Repr. 1B (2004)	Toxic for reproduction (2008); Endocrine disrupting properties HH (2017)	Subject to Authorization since 2015 (Annex XVI); Restriction in toys, childcare (2005) and any plasticized articles since 2020 (Annex XVII)	
Diethyl phthalate	DEP				No ED properties HH
Di-isodecyl phthalate (and ^1^ DPHP)	DiDP			Restriction in toys, childcare articles since 2005 (Annex XVII) ^2^	
Di-isononyl phthalate	DiNP			Restriction in toys, childcare articles since 2005 (Annex XVII)	
Bis(2-propylheptyl) phthalate	DPHP				Evaluation for ED potential
Dicyclohexyl phthalate	DCHP	Repr. 1B (2016)	Toxic for reproduction (2018); Endocrine disrupting properties HH (2018)		Evaluation for ED properties ENV
Dimethyl phthalate	DMP				
Di-n-pentyl phthalate	DnPeP	Repr. 1B (2004)	Toxic for reproduction (2013)	Subject to Authorization since 2020 (Annex XVI)	
Di-n-octyl phthalate	DnOP			Restriction in toys, childcare articles since 2005 (Annex XVII)	

REACH: Regulation (EC) No 1907/2006; CoRAP: Community Rolling Action Plan; Repr. 1B: Classification as reproductive toxicant, Category 1B (probably reprotoxic to humans, based on chronic animal studies); ENV: for the environment; HH: for human health; ED: endocrine disrupting.^1^ The analytical method applied cannot distinguish between DiDP and DPHP metabolites. ^2^ Only applies to DiDP.
