# Supplementary material for: Exposure to Phthalates in European Children, Adolescents and Adults since 2005: A Harmonized Approach Based on Existing HBM Data in the HBM4EU Initiative
Source: Toxics. 2023 Mar 4;11(3):241. doi: 10.3390/toxics11030241 (PMC10057641; doi:10.3390/toxics11030241)
Supplement: Supplementary file 1 [file toxics-11-00241-s001.zip › toxics-2160558-supplementary.pdf]

Supplementary to

Exposure to Phthalates in European Children, Adolescents and Adults Since 2005: A Harmonized Approach Based on Existing HBM Data in the HBM4EU Initiative

Nina Vogel [1,*], Rosa Lange [1], Phillipp Schmidt [1], Laura Rodriguez Martin [2], Sylvie Remy [2], Andrea Springer [1], Vladimíra Puklová [3], Milena Černá [3], Péter Rudnai [4], Szilvia Középesy [4], Beata Janasik [5], Danuta Ligocka [5], Lucia Fábelová [6], Branislav Kolena [7], Ida Petrovicova [7], Michal Jajcaj [8], Milada Eštóková [8], Marta Esteban-Lopez [9], Argelia Castaño [9], Janja Snoj Tratnik [10], Anja Stajnko [10], Lisbeth E. Knudsen [11], Jorma Toppari [12,13], Katharina M Main [14,15,16], Anders Juul [14,15,16], Anna-Maria Andersson [14,15], Niels Jørgensen [14,15], Hanne Frederiksen [14,15], Cathrine Thomsen [17], Amrit Kaur Sakhi [17], Agneta Åkesson [18], Christina Hartmann [19], Marie Christine Dewolf [20], Gudrun Koppen [2], Pierre Biot [21], Elly Den Hond [22], Stefan Voorspoels [2], Liese Gilles [2], Eva Govarts [2], Aline Murawski [1], Antje Gerofke [1], Till Weber [1], Maria Rüther [1], Arno C Gutleb [23], Cedric Guignard [23], Tamar Berman [24], Holger M Koch [25] and Marike Kolossa-Gehring [1]

1 German Environment Agency (UBA), Department of Toxicology, Health-related Environmental Monitoring, 14195 Berlin, Germany; rosa.lange@uba.de (R.L.); phillipp.schmidt@uba.de (P.S.); springeran67@gmail.com (A.S.); aline.murawski@uba.de (A.M.); antje.gerofke@uba.de (A.G.); till.weber@uba.de (T.W.); maria.ruether@googlemail.com (M.R.); marike.kolossa@uba.de (M.K.-G.)

2 Flemish Institute for Technological Research (VITO), 2400 Mol, Belgium; laura.rodriguezmartin@vito.be (L.R.M.); sylvie.remy@vito.be (S.R.); gudrun.koppen@vito.be (G.K.); stefan.voorspoels@vito.be (S.V.); liese.gilles@vito.be (L.G.); eva.govarts@vito.be (E.G.)

3 National Institute of Public Health, Centre for Health and Environment, 100 00, Prague, Czech Republic; vladimira.puklova@szu.cz (V.P.); milena.cerna@szu.cz (M.Č.); lucia.fabelova@szu.sk (L.F.)

4 National Public Health Center, Environmental Health Unit of the Department of Public Health Laboratory, 1097, Budapest, Hungary; prudnai1@gmail.com (P.R.); kozepesy.szilvia@nnk.gov.hu (S.K.)

5 Nofer Institute of Occupational Medicine, 91-348 Lodz, Poland; beata.janasik@imp.lodz.pl (B.J.); danuta.ligocka@gmail.com (D.L.)

6 Slovak Medical University, Faculty of Public Health, Department of Environmental Medicine, 83303 Bratislava, Slovakia; lucia.fabelova@szu.sk (L.F.)

7 Constantine the Philosopher University in Nitra, Faculty of Natural Sciences and Informatics, Department of Zoology and Anthropology, 949 01 Nitra, Slovakia; bkolena@ukf.sk (B.K.); ipetrovicova@ukf.sk (I.P.)

8 Public Health Authority, Department of Environment and Health, 83105 Bratislava, Slovakia; michal.jajcaj@uvzsr.sk (M.J.); milada.estokova@uvzsr.sk (M.E.)

9 Instituto de Salud Carlos III, 28220 Majadahonda, Spain; m.esteban@isciii.es (M.E.-L.); castano@isciii.es (A.C.)

10 Jožef Stefan Institute, Department of Environmental Sciences, Jamova cesta 39, 1000 Ljubljana, Slovenia; janja.tratnik@ijs.si (J.S.T.); anja.stajnko@ijs.si (A.S.)

11 University of Copenhagen, Department of Public Health, DK1165 Copenhagen, Denmark; liek@sund.ku.dk

12 Research Centre for Integrative Physiology and Pharmacology, Institute of Biomedicine, University of Turku, 20520 Turku, Finland; jorma.toppari@utu.fi

13 Department of Pediatrics, Turku University Hospital, Turku, Finland

14 Department of Growth and Reproduction, Copenhagen University Hospital—Rigshospitalet, 2100 Copenhagen, Denmark; katharina.main@regionh.dk (K.M.M.); anders.juul@regionh.dk (A.J.); anna-maria.andersson@regionh.dk (A.-M.A.); niels.joergensen@regionh.dk (N.J.); hanne.frederiksen@regionh.dk (H.F.)

15 International Centre for Research and Research Training in Endocrine Disruption of Male Reproduction and Child Health (EDMaRC), Copenhagen University Hospital—Rigshospitalet, 2100 Copenhagen, Denmark

16 Department of Clinical Medicine, University of Copenhagen, 2200 Copenhagen, Denmark

17 Department of Food Safety, Norwegian Institute of Public Health, 0456 Oslo, Norway; cathrine.thomsen@fhi.no (C.T.); amritkaur.sakhi@fhi.no (A.K.S.)

18 Institute of Environmental Medicine, Karolinska Institutet, 171 77 Stockholm, Sweden; agneta.akesson@ki.se

19 Environment Agency Austria, 1090 Vienna, Austria; christina.hartmann@umweltbundesamt.at

20 Hainaut Analyse, 7000 Mons, Belgium; marie_christine.dewolf@hainaut.be

21 Federal Public Service Health, Food Chain Safety and Environment, Brussels, Belgium; pierre.biot@health.fgov.be

22 Provincial Institute of Hygiene (PIH), department of Environment and Health, 2000 Antwerp, Belgium; elly.denhond@provincieantwerpen.be

23 Luxembourg Institute of Science and Technology, Environmental Research and Innovation (ERIN) Department, L-4422 Belvaux, Luxembourg; arno.gutleb@list.lu (A.C.G.); cedric.guignard@list.lu (C.G.)

24 Department of Environmental Health, Ministry of Health, 9446724 Jerusalem, Israel; tamar.berman@moh.health.gov.il

25 Institute for Prevention and Occupational Medicine of the German Social Accident Insurance —Institute of the Ruhr University Bochum (IPA), 44789 Bochum, Germany; holger.koch@dguv.de

* Correspondence: nina.vogel@uba.de

Table of contents

Table S1: BBzP metabolite MBzP (Mono-benzyl phthalate) in microg/L 6

Table S2: DEHP metabolite MEHP (Mono(2-ethylhexyl) phthalate) in microg/L........ 9

Table S3: DEHP metabolite 5OH-MEHP (Mono(2-ethyl-5-hydroxy- hexyl) phthalate) in microg/L..... 12

Table S4: DEHP metabolite 5oxo-MEHP (Mono(2-ethyl-5-oxo-hexyl) phthalate) in microg/L........ 15

Table S5: DEHP metabolite 5cx-MEPP (Mono(2-ethyl-5-carboxy- pentyl) phthalate) in microg/L........ 19

Table S6: DnBP metabolite MnBP (Mono-n-butyl phthalate) in microg/L........ 21

Table S7: DnBP metabolite OH-MnBP (3-OH-Mono-n-butyl phthalate) in microg/L........ 23

Table S8: DiBP metabolite MiBP (Mono-isobutyl phthalate) in microg/L........ 25

Table S9: DiBP metabolite OH-MiBP (2-OH-Mono-iso- butylphthalate) in microg/L........ 27

Table S10: DEP metabolite MEP (Mono-ethyl phthalate) in microg/L 29

Table S11: DMP metabolite MMP (Mono-methyl phthalate) in microg/L 31

Table S12: Metabolite 3cx-MPP (3-carboxyl-mono-propyl phthalate) in microg/L 33

Table S13: DiNP metabolite cx-MiNP (7-Carboxy-(mono-methyl- heptyl) phthalate) in microg/L 35

Table S14: DiNP metabolite OH-MiNP (7-OH-(Mono-methyl-octyl) phthalate) in microg/L 37

Table S15: DiNP metabolite oxo-MiNP (7-Oxo-(Mono-methyl-octyl) phthalate) in microg/L........ 39

Table S16: DiNP metabolite MiNP (Mono-methyl-octyl phthalate) in microg/L........ 41

Table S17: DiDP metabolite cx-MiDP (Mono(2,7-methyl-7- carboxy-heptyl) phthalate) in microg/L ... 43

Table S18: DiDP metabolite OH-MiDP (6-OH-Mono-propyl-heptyl phthalate) in microg/L 45

Table S19: DiDP metabolite oxo-MiDP (6-Oxo-Mono-propyl-heptyl phthalate) in microg/L........ 47

Table S20: DINCH metabolite cx-MINCH (cyclohexane-1,2- dicarboxylate-mono-(7- carboxylate-4-methyl)heptyl ester) in microg/L 49

Table S21: DINCH metabolite OH-MINCH (cyclohexane-1,2- dicarboxylate-mono-(7- hydroxy-4-methyl)octyl ester) in microg/L 51

Table S22: DINCH metabolite oxo-MINCH (cyclohexane-1,2- dicarboxylate-mono-(7-oxo- 4-methyl)octyl ester) in microg/L 53

Table S23: DEHTP metabolite 2cx-MMHTP (1-mono-(2-carboxyl-methyl-hexyl) benzene-1,4-dicarboxylate) in microg/L........ 55

Table S24: DEHTP metabolite 5cx-MEPTP (1-mono-(2-ethyl-5-carboxyl-pentyl) benzene-1,4-dicarboxylate) in microg/L........ 55

Table S25: DEHTP metabolite 5OH-MEHTP (1-mono-(2-ethyl-5-hydroxy-hexyl) benzene-1,4-dicarboxylate) in microg/L........ 56

Table S26: DEHTP metabolite 5oxo-MEHTP (1-mono-(2-ethyl-5-oxo-hexyl) benzene-1,4-dicarboxylate) in microg/L........ 56

Figure S1. BBzP metabolite MBzP (Mono-benzyl phthalate) concentration in µg/L stratified by sampling year in boxplots........ 57

Figure S2. BBzP metabolite MBzP (Mono-benzyl phthalate) concentration in µg/L stratified by age groups in boxplots. 58

Figure S3. DEHP metabolite 5OH-MEHP (Mono(2-ethyl-5-hydroxy- hexyl) phthalate) concentration in µg/L stratified by sampling year in boxplots. 60

Figure S4. DEHP metabolite 5OH-MEHP (Mono(2-ethyl-5-hydroxy- hexyl) phthalate) concentration in µg/L stratified by age groups in boxplots. 61

Figure S5. DEHP metabolite 5oxo-MEHP (Mono(2-ethyl-5-oxo-hexyl) phthalate) concentration in µg/L stratified by region in boxplots. 63

Figure S6. DEHP metabolite 5oxo-MEHP (Mono(2-ethyl-5-oxo-hexyl) phthalate) concentration in µg/L stratified by sampling year in boxplots. 64

Figure S7. DEHP metabolite 5oxo-MEHP (Mono(2-ethyl-5-oxo-hexyl) phthalate) concentration in µg/L stratified by age groups in boxplots. 65

Figure S8. DEHP metabolite 5cx-MEPP (Mono(2-ethyl-5-carboxy- pentyl) phthalate) concentration in µg/L stratified by region in boxplots. 67

Figure S9. DEHP metabolite 5cx-MEPP (Mono(2-ethyl-5-carboxy- pentyl) phthalate) concentration in µg/L stratified by sampling year in boxplots. 68

Figure S10. DEHP metabolite 5cx-MEPP (Mono(2-ethyl-5-carboxy- pentyl) phthalate) concentration in µg/L stratified by age groups in boxplots. 69

Figure S11. DEHP metabolite MEHP (Mono(2-ethylhexyl) phthalate) concentration in µg/L stratified by region in boxplots. 71

Figure S12. DEHP metabolite MEHP (Mono(2-ethylhexyl) phthalate) concentration in µg/L stratified by sampling year in boxplots. 72

Figure S13. DEHP metabolite MEHP (Mono(2-ethylhexyl) phthalate) concentration in µg/L stratified by age groups in boxplots. 73

Figure S14. DnBP metabolite MnBP (Mono-n-butyl phthalate) concentration in µg/L stratified by region in boxplots. 75

Figure S15. DnBP metabolite MnBP (Mono-n-butyl phthalate) concentration in µg/L stratified by sampling year in boxplots. 76

Figure S16. DnBP metabolite OH-MnBP (3-OH-Mono-n-butyl phthalate) concentration in µg/L stratified by region in boxplots. 77

Figure S17. DnBP metabolite OH-MnBP (3-OH-Mono-n-butyl phthalate) concentration in µg/L stratified by sampling year in boxplots. 78

Figure S18. DnBP metabolite OH-MnBP (3-OH-Mono-n-butyl phthalate) concentration in µg/L stratified by age groups in boxplots. 79

Figure S19. DiBP metabolite MiBP (Mono-isobutyl phthalate) concentration in µg/L stratified by sampling year in boxplots. 81

Figure S20. DiBP metabolite MiBP (Mono-isobutyl phthalate) concentration in µg/L stratified by age groups in boxplots. 82

Figure S21. DiBP metabolite OH-MiBP (2-OH-Mono-iso- butylphthalate) concentration in µg/L stratified by region in boxplots. 84

Figure S22. DiBP metabolite OH-MiBP (2-OH-Mono-iso- butylphthalate) concentration in µg/L stratified by sampling year in boxplots. 85

Figure S23. DiBP metabolite OH-MiBP (2-OH-Mono-iso- butylphthalate) concentration in µg/L stratified by age groups in boxplots. 86

Figure S24. DEP metabolite MEP (Mono-ethyl phthalate) concentration in µg/L stratified by region in boxplots. 88

Figure S25. DEP metabolite MEP (Mono-ethyl phthalate) concentration in µg/L stratified by sampling year in boxplots. 89

Figure S26. DMP metabolite MMP (Mono-methyl phthalate) concentration in µg/L stratified by region in boxplots. 90

Figure S27. DMP metabolite MMP (Mono-methyl phthalate) concentration in µg/L stratified by sampling year in boxplots. 91

Figure S28. DMP metabolite MMP (Mono-methyl phthalate) concentration in µg/L stratified by age groups in boxplots. 92

Figure S29. Metabolite 3cx-MPP (3-carboxyl-mono-propyl phthalate) concentration in µg/L stratified by region in boxplots. 94

Figure S30. Metabolite 3cx-MPP (3-carboxyl-mono-propyl phthalate) concentration in µg/L stratified by sampling year in boxplots. 95

Figure S31. Metabolite 3cx-MPP (3-carboxyl-mono-propyl phthalate) concentration in µg/L stratified by age groups in boxplots. 96

Figure S32. DiNP metabolite cx-MiNP (7-Carboxy-(mono-methyl- heptyl) phthalate) concentration in µg/L stratified by sampling year in boxplots. 98

Figure S33. DiNP metabolite cx-MiNP (7-Carboxy-(mono-methyl- heptyl) phthalate) concentration in µg/L stratified by age groups in boxplots. 99

Figure S34. DiNP metabolite OH-MiNP (7-OH-(Mono-methyl-octyl) phthalate) concentration in µg/L stratified by region in boxplots. 101

Figure S35. DiNP metabolite OH-MiNP (7-OH-(Mono-methyl-octyl) phthalate) concentration in µg/L stratified by sampling year in boxplots. 102

Figure S36. DiNP metabolite OH-MiNP (7-OH-(Mono-methyl-octyl) phthalate) concentration in µg/L stratified by age groups in boxplots. 103

Figure S37. DiNP metabolite oxo-MiNP (7-Oxo-(Mono-methyl-octyl) phthalate) concentration in µg/L stratified by region in boxplots. 105

Figure S38. DiNP metabolite oxo-MiNP (7-Oxo-(Mono-methyl-octyl) phthalate) concentration in µg/L stratified by sampling year in boxplots. 106

Figure S39. DiNP metabolite oxo-MiNP (7-Oxo-(Mono-methyl-octyl) phthalate) concentration in µg/L stratified by age groups in boxplots. 107

Figure S40. DiDP metabolite cx-MiDP (Mono(2,7-methyl-7- carboxy-heptyl) phthalate) concentration in µg/L stratified by region in boxplots. 109

Figure S41. DiDP metabolite cx-MiDP (Mono(2,7-methyl-7- carboxy-heptyl) phthalate) concentration in µg/L stratified by sampling year in boxplots. 110

Figure S42. DiDP metabolite OH-MiDP (6-OH-Mono-propyl-heptyl phthalate) concentration in µg/L stratified by region in boxplots. 111

Figure S43. DiDP metabolite OH-MiDP (6-OH-Mono-propyl-heptyl phthalate) concentration in µg/L stratified by sampling year in boxplots. 112

Figure S44. DiDP metabolite OH-MiDP (6-OH-Mono-propyl-heptyl phthalate) concentration in µg/L stratified by age groups in boxplots. 113

Figure S45. DiDP metabolite oxo-MiDP (6-Oxo-Mono-propyl-heptyl phthalate) concentration in µg/L stratified by region in boxplots. 115

Figure S46. DiDP metabolite oxo-MiDP (6-Oxo-Mono-propyl-heptyl phthalate) concentration in µg/L stratified by sampling year in boxplots. 116

Figure S47. DiDP metabolite oxo-MiDP (6-Oxo-Mono-propyl-heptyl phthalate) concentration in µg/L stratified by age groups in boxplots. 117

Figure S48. DINCH metabolite cx-MINCH (cyclohexane-1,2- dicarboxylate-mono-(7- carboxylate-4-methyl)heptyl ester) concentration in µg/L stratified by region in boxplots. 119

Figure S49. DINCH metabolite cx-MINCH (cyclohexane-1,2- dicarboxylate-mono-(7- carboxylate-4-methyl)heptyl ester) concentration in µg/L stratified by sampling year in boxplots. 120

Figure S50. DINCH metabolite cx-MINCH (cyclohexane-1,2- dicarboxylate-mono-(7- carboxylate-4-methyl)heptyl ester) concentration in µg/L stratified by age groups in boxplots. 121

Figure S51. DINCH metabolite OH-MINCH (cyclohexane-1,2- dicarboxylate-mono-(7- hydroxy-4-methyl)octyl ester) concentration in µg/L stratified by region in boxplots. 123

Figure S52. DINCH metabolite OH-MINCH (cyclohexane-1,2- dicarboxylate-mono-(7- hydroxy-4-methyl)octyl ester) concentration in µg/L stratified by sampling year in boxplots. 124

Figure S53. DINCH metabolite OH-MINCH (cyclohexane-1,2- dicarboxylate-mono-(7- hydroxy-4-methyl)octyl ester) concentration in µg/L stratified by age groups in boxplots. 125

Figure S54. DINCH metabolite oxo-MINCH (cyclohexane-1,2- dicarboxylate-mono-(7-oxo- 4-methyl)octyl ester) concentration in µg/L stratified by region in boxplots. 127

Figure S55. DINCH metabolite oxo-MINCH (cyclohexane-1,2- dicarboxylate-mono-(7-oxo- 4-methyl)octyl ester) concentration in µg/L stratified by sampling year in boxplots. 128

Figure S56. DINCH metabolite oxo-MINCH (cyclohexane-1,2- dicarboxylate-mono-(7-oxo- 4-methyl)octyl ester) concentration in µg/L stratified by age groups in boxplots. 129

Table S1: BBzP metabolite MBzP (Mono-benzyl phthalate) in microg/L

Age	Matrix	Country	Data collection	Sampling year	Sex	N	LOD	LOQ	% below limit	P50	P50_95% CI	P75	P75_95% CI	P90	P90_95% CI	P95	P95_95% CI	GM	GM_95% CI
3-5	mU	DE	GerES IV 2005	2005	F,M	50		0.25	0.00	24.8	16.7 - 30.8	46.6	30.5 - 52.5	55.0	50.2 - 91.7	66.0		21.8	17.2 - 27.6
3-5	sU	DK	CPH-MC	2006	F,M	189	1.26		9.52	18.5	14.8 - 23.0	42.9	35.2 - 57.0	82.4	71.2 - 111	121	95.2 - 241	15.6	12.6 - 19.4
3-5	mU	DE	GerES V	2015 - 2017	F,M	388		0.20	0.26	3.65	3.20 - 4.30	7.33	6.36 - 8.69	15.7	12.6 - 20.6	30.3	20.6 - 44.4	3.91	3.51 - 4.37
3-5	sU	SK	Children cohort	2015 - 2016	F,M	67		0.20	0.00	2.36	1.92 - 3.70	5.02	3.97 - 8.35	9.35	6.84 - 15.6	13.9		2.31	1.75 - 3.04
3-5	mU	CZ	CzechHBM-CE	2016 - 2017	F,M	148		1.90	25.0	3.16	2.68 - 3.76	6.70	5.25 - 9.05	16.9	11.8 - 43.2	43.5	18.6 - 94.6	3.28	2.66 - 4.05
6-11	mU	DE	GerES IV 2005	2005	F,M	102		0.25	0.00	19.8	16.9 - 24.5	36.7	26.7 - 51.6	66.3	54.4 - 88.6	88.2	69.8 - 112	20.1	16.9 - 23.9
6-11	sU	DK	CPH-MC	2006 - 2007	F,M	659	1.26		15.5	13.7	12.0 - 15.7	31.2	28.1 - 35.9	59.7	52.9 - 67.1	93.3	73.7 - 111	10.7	9.52 - 12.0
6-11	mU	DK	CPHPUB-Cross	2006 - 2008	F,M	913	1.14		0.00	50.3	46.2 - 54.0	87.1	83.2 - 93.8	151	132 - 161	220	187 - 258	50.8	48.0 - 53.8
6-11	sU	AT	PBAT	2010 - 2012	F,M	165	0.59	1.18	23.6	2.69	2.35 - 3.34	7.04	5.35 - 9.30	14.2	11.1 - 23.8	28.7	16.3 - 40.5	2.99	2.49 - 3.59
6-11	mU	SE	DEMOCOPHES-SE	2011 - 2012	F,M	98		0.30	0.00	22.1	17.7 - 24.8	36.2	28.1 - 41.2	56.8	42.8 - 81.8	80.2	58.9 - 118	19.7	16.6 - 23.3
6-11	mU	DK	DEMOCOPHES-DK	2011	F,M	143		1.14	2.80	7.03	6.11 - 8.06	12.1	9.85 - 16.0	24.0	17.4 - 30.9	31.0	26.2 - 51.4	7.24	6.26 - 8.38
6-11	mU	ES	DEMOCOPHES-ES	2011 - 2012	F,M	120		0.20	0.00	12.8	9.74 - 15.6	23.4	19.4 - 27.2	34.8	27.5 - 58.1	57.8	36.1 - 70.4	12.5	10.8 - 14.6
6-11	mU	SL	DEMOCOPHES-SI	2011 - 2012	F,M	119		0.50	0.00	7.60	6.44 - 9.72	17.0	13.3 - 22.0	26.4	23.0 - 34.0	34.0	28.0 - 61.9	8.40	7.11 - 9.92
6-11	mU	LU	DEMOCOPHES-LU	2011 - 2012	F,M	60		0.20	1.67	4.25	3.11 - 5.40	8.43	5.48 - 10.7	13.4	9.42 - 35.8	24.1		4.49	3.46 - 5.82
6-11	mU	DE	DEMOCOPHES-DE	2011	F,M	119		0.20	0.00	6.01	4.76 - 7.18	11.0	8.78 - 13.7	21.7	15.0 - 44.9	34.2	23.8 - 234	6.43	5.27 - 7.85
6-11	mU	BE	DEMOCOPHES-BE	2011 - 2012	F,M	129		0.20	0.00	8.40	6.80 - 10.0	14.0	12.4 - 20.6	26.2	21.6 - 34.7	34.2	27.8 - 100	8.44	7.15 - 9.97
6-11	mU	HU	DEMOCOPHES-HU	2011 - 2012	F,M	120		1.20	2.50	7.37	5.44 - 8.68	13.2	10.3 - 16.5	24.3	17.7 - 44.0	44.0	25.0 - 264	7.33	6.01 - 8.95
6-11	mU	PL	DEMOCOPHES-PL	2011 - 2012	F,M	120		0.50	0.83	9.75	7.26 - 12.0	17.2	15.0 - 21.9	29.1	23.7 - 60.5	60.0	33.3 - 177	8.78	7.14 - 10.8
6-11	mU	CZ	DEMOCOPHES-CZ	2011 - 2012	F,M	120		1.20	0.00	7.55	6.14 - 9.69	14.2	11.2 - 21.6	35.5	22.4 - 60.9	55.7	36.8 - 135	8.32	6.89 - 10.0
6-11	mU	SK	DEMOCOPHES-SK	2011 - 2012	F,M	129		1.20	0.78	8.18	6.24 - 9.50	14.8	12.8 - 17.1	27.9	18.3 - 43.0	41.8	32.5 - 103	8.51	7.25 - 10.00
6-11	mU	NO	IES	2012	F,M	50	0.07	0.20	0.00	6.94	5.40 - 7.80	9.43	7.79 - 12.6	18.4	10.3 - 24.7	20.0		6.41	5.27 - 7.80
6-11	mU	DE	GerES V	2015 - 2017	F,M	937		0.20	0.32	3.10	2.90 - 3.20	5.70	5.20 - 6.10	11.3	9.98 - 14.5	18.7	16.8 - 25.6	3.32	3.11 - 3.54
6-11	sU	SK	Children cohort	2015 - 2016	F,M	112		0.20	0.00	3.00	2.19 - 4.04	6.18	4.88 - 8.19	11.0	8.32 - 21.5	17.8	11.3 - 71.1	2.94	2.34 - 3.68
6-11	mU	CZ	CzechHBM-CE	2016 - 2017	F,M	223		1.90	30.0	3.22	2.69 - 3.90	6.87	6.26 - 9.91	19.9	13.1 - 27.1	29.1	24.5 - 61.8	3.33	2.82 - 3.94
12-19	mU	DE	GerES IV 2005	2005	F,M	51		0.25	0.00	13.0	9.79 - 18.0	31.6	17.9 - 41.4	47.5	33.4 - 84.6	63.2		14.3	11.0 - 18.4
12-19	mU	DK	CPHPUB-Cross	2006 - 2008	F,M	389	1.14		0.00	46.2	41.1 - 49.9	83.2	73.6 - 93.3	138	111 - 161	182	160 - 244	45.0	41.2 - 49.1
12-19	sU	DK	DYMS 2007	2007	M	239	1.14	1.14	0.84	35.1	30.6 - 40.6	60.6	51.4 - 71.7	102	85.8 - 117	128	113 - 164	31.9	28.2 - 36.0
12-19	sU	DK	DYMS 2008	2008	M	261	1.14	1.14	0.38	31.5	29.5 - 37.8	58.8	52.1 - 71.2	116	87.7 - 142	163	129 - 228	32.0	28.2 - 36.4
12-19	mU	BE	FLEHS2RefAdo	2008 - 2009	F,M	210	0.20		0.00	29.5	25.0 - 35.0	67.0	56.5 - 83.0	115	98.6 - 158	163	128 - 237	31.9	27.8 - 36.6
12-19	sU	DK	DYMS 2009	2009	M	210	0.03	0.03	0.48	18.7	16.9 - 22.5	40.8	33.9 - 49.2	80.4	57.8 - 130	145	89.8 - 352	19.7	16.8 - 23.2
12-19	sU	AT	PBAT	2010 - 2012	F,M	94	0.59	1.18	23.4	2.66	2.23 - 3.62	6.98	4.74 - 10.6	12.8	10.7 - 19.0	17.5	13.5 - 39.8	2.99	2.38 - 3.76
12-19	sU	DK	DYMS 2013	2013	M	70	0.03	0.03	2.86	4.97	3.50 - 5.94	8.01	6.57 - 10.8	12.8	9.90 - 23.7	17.1		3.84	2.84 - 5.18
12-19	sU	BE	FLEHS3RefAdo	2013	F,M	207		0.20	0.00	5.25	4.59 - 5.93	10.2	8.93 - 13.8	21.7	18.1 - 27.1	28.4	24.4 - 37.1	5.40	4.69 - 6.23
12-19	mU	DE	GerES V	2015 - 2017	F,M	932		0.20	0.32	2.60	2.40 - 2.70	4.70	4.40 - 5.10	9.39	8.30 - 10.6	14.1	12.5 - 17.5	2.69	2.53 - 2.86

Age	Matrix	Country	Data collection	Sampling year	Sex	N	LOD	LOQ	% below limit	P50	P50_95% CI	P75	P75_95% CI	P90	P90_95% CI	P95	P95_95% CI	GM	GM_95% CI
12-19	sU	DK	DYMS 2017	2017	M	76	0.03	0.03	2.63	2.36	1.95 - 3.08	4.23	3.60 - 5.23	8.36	5.08 - 18.2	15.9	8.38 - 25.6	2.21	1.68 - 2.92
20-39	24hU	DE	ESB 2007	2007	F,M	60		0.20	0.00	2.85	2.40 - 3.70	5.50	3.78 - 8.47	12.7	7.17 - 27.1	16.9		3.28	2.57 - 4.20
20-39	sU	DK	DYMS 2008	2008	M	54	1.14	1.14	0.00	47.5	28.5 - 68.3	80.7	68.3 - 114	168	89.0 - 271	205		40.3	30.6 - 53.2
20-39	mU	BE	FLEHS2RefAdult	2008 - 2009	F,M	189	0.20		0.53	21.0	17.6 - 25.4	36.0	32.0 - 44.0	67.0	54.2 - 89.2	93.0	71.0 - 158	18.5	15.7 - 21.8
20-39	24hU	DE	ESB 2008	2008	F,M	54		0.50	3.70	3.84	3.10 - 4.58	5.58	4.58 - 7.04	8.12	6.32 - 20.1	10.1		3.64	2.98 - 4.45
20-39	sU	DK	DYMS 2009	2009	M	79	0.03	0.03	0.00	19.4	14.3 - 28.5	41.1	35.3 - 55.7	70.0	55.1 - 99.3	92.2	70.3 - 482	19.5	15.3 - 24.7
20-39	24hU	DE	ESB 2009	2009	F,M	60		0.20	0.00	3.15	2.60 - 4.00	4.62	4.00 - 6.60	9.54	5.36 - 27.2	16.0		3.11	2.44 - 3.97
20-39	sU	AT	PBAT	2010 - 2011	F,M	135	0.59	1.18	37.0	1.55	1.32 - 1.85	3.01	2.16 - 4.10	6.08	4.87 - 10.9	10.9	6.64 - 18.0	1.65	1.40 - 1.95
20-39	mU	SE	DEMOCOPHES-SE	2011 - 2012	F	50		0.30	0.00	18.0	10.2 - 21.5	33.1	21.1 - 67.8	85.0	55.0 - 209	108		15.9	11.1 - 22.6
20-39	mU	DK	DEMOCOPHES-DK	2011	F	51		1.14	5.88	4.96	3.66 - 6.68	11.0	6.54 - 17.0	19.2	14.2 - 24.5	22.8		5.08	3.91 - 6.62
20-39	mU	SL	DEMOCOPHES-SI	2011 - 2012	F	63		0.50	0.00	6.50	4.97 - 7.77	10.0	7.89 - 11.0	13.0	11.0 - 30.1	22.9		5.94	4.81 - 7.34
20-39	mU	DE	DEMOCOPHES-DE	2011	F	64		0.20	0.00	5.55	3.51 - 7.05	9.59	7.12 - 16.6	17.7	11.7 - 37.5	30.9		5.44	4.29 - 6.90
20-39	mU	BE	DEMOCOPHES-BE	2011 - 2012	F	62		0.20	0.00	5.95	4.38 - 8.02	11.5	8.25 - 14.0	16.7	13.3 - 39.6	19.9		5.90	4.63 - 7.53
20-39	24hU	DE	ESB 2011	2011	F,M	60		0.20	0.00	3.25	2.30 - 4.30	5.25	4.30 - 8.67	9.93	7.06 - 18.2	13.2		3.36	2.68 - 4.21
20-39	mU	HU	DEMOCOPHES-HU	2011 - 2012	F	89		1.20	4.49	4.77	3.83 - 5.69	9.04	6.57 - 11.6	16.1	11.6 - 24.6	22.3	17.8 - 55.8	5.06	4.20 - 6.09
20-39	mU	PL	DEMOCOPHES-PL	2011 - 2012	F	88		0.50	1.14	3.75	2.80 - 5.49	7.95	6.18 - 17.1	19.3	16.7 - 34.0	26.6	19.7 - 48.7	4.43	3.55 - 5.54
20-39	mU	CZ	DEMOCOPHES-CZ	2011 - 2012	F	88		1.20	10.2	3.65	2.72 - 5.51	8.28	6.53 - 11.4	17.1	11.3 - 124	37.8	17.9 - 507	4.48	3.40 - 5.91
20-39	mU	SK	DEMOCOPHES-SK	2011 - 2012	F	91		1.20	7.69	5.21	3.66 - 5.99	8.97	7.72 - 9.88	11.4	9.87 - 17.5	16.4	12.2 - 39.1	4.56	3.80 - 5.47
20-39	sU	IL	IBS	2011	F,M	144	0.50	1.00	4.86	4.71	3.86 - 5.55	9.35	7.30 - 12.5	21.2	14.0 - 53.3	54.9	22.1 - 70.7	5.01	4.18 - 6.02
20-39	24hU	DE	ESB 2013	2013	F,M	60		0.20	0.00	2.00	1.70 - 2.60	3.50	2.68 - 4.50	5.37	4.40 - 20.7	12.8		2.31	1.88 - 2.85
20-39	24hU	DE	ESB 2015	2015	F,M	60		0.20	1.67	1.20	1.00 - 1.80	3.23	1.88 - 5.97	6.82	5.15 - 33.8	13.7		1.68	1.23 - 2.29
20-39	sU	CZ	CzechHBM-AE	2018 - 2019	F,M	194		2.37	76.8					4.05	3.26 - 5.64	6.37	4.28 - 15.5		
40-59	sU	AT	PBAT	2010 - 2011	F,M	112	0.59	1.18	51.8			2.50	1.77 - 3.46	4.90	3.54 - 7.49	7.26	5.32 - 10.1		
40-59	mU	DK	DEMOCOPHES-DK	2011	F	94		1.14	9.57	3.64	2.74 - 4.26	6.08	5.05 - 7.20	8.29	7.23 - 20.7	17.2	8.60 - 32.5	3.50	2.93 - 4.17
40-59	mU	ES	DEMOCOPHES-ES	2011 - 2012	F	75		0.20	0.00	9.80	7.19 - 11.7	17.1	13.0 - 22.0	27.4	19.8 - 46.6	41.9	27.4 - 82.3	8.66	6.84 - 11.0
40-59	mU	SL	DEMOCOPHES-SI	2011 - 2012	F	57		0.50	0.00	4.00	3.35 - 4.85	7.40	4.94 - 10.2	11.0	9.20 - 17.0	14.6		4.20	3.42 - 5.16
40-59	mU	DE	DEMOCOPHES-DE	2011	F	56		0.20	0.00	3.35	2.40 - 4.24	6.61	4.26 - 7.97	10.8	7.54 - 18.1	14.9		3.45	2.70 - 4.42
40-59	mU	BE	DEMOCOPHES-BE	2011 - 2012	F	67		0.20	0.00	6.50	5.23 - 8.19	12.0	8.46 - 15.0	17.4	15.0 - 26.3	23.7		6.77	5.60 - 8.17
40-59	sU	IL	IBS	2011	F,M	86	0.50	1.00	13.9	3.96	2.77 - 5.16	8.21	6.62 - 11.9	18.4	11.8 - 26.0	25.0	18.9 - 192	4.21	3.30 - 5.38
40-59	sU	CZ	CzechHBM-AE	2018 - 2019	F,M	192		2.37	77.1					4.43	3.25 - 7.29	7.43	5.05 - 9.97		
60+	sU	AT	PBAT	2010 - 2012	F,M	89	0.59	1.18	44.9	1.30		3.09	2.01 - 6.39	10.8	6.16 - 14.9	13.0	10.8 - 30.4	1.47	1.13 - 1.91

Notes. Matrix: mU - first morning urine, sU - spot urine, 24hU - 24-hours urine; country: AT - Austria, BE - Belgium, CZ - Czech Republic, DE - Germany, DK - Denmark, ES - Spain, HU - Hungary, IL - Israel, LU - Luxemburg, NO - Norway, PL - Poland, SE - Sweden, SI - Slovenia, SK - Slovakia; sex: F - female, M - male; n - sample size; LOD - limit of detection; LOQ - limit of quantification; % below limit - percentage of values below limit of detection/quantification, whichever was applied in the data collection; P50, P75, P90, P95 - 50th, 75th, 90th, 95th percentile; 95% CI - 95 % confidence interval; GM - geometric mean; NA - Not available.

Age	Matrix	Country	Data collection	Sampling year	Sex	N	LOD	LOQ	% below limit	P50	P50_95% CI	P75	P75_95% CI	P90	P90_95% CI	P95	P95_95% CI	GM	GM_95% CI

Data only displayed if n was at least 50; percentiles missing when below LOD/LOQ; CIs of the observed percentiles missing when they fall outside the observed range; GM was imputed (fixed imputation) based on a log-normal distribution when at least 50 % of values were above LOD/LOQ.

Table S2: DEHP metabolite MEHP (Mono(2-ethylhexyl) phthalate) in microg/L

Age	Matrix	Country	Data collection	Sampling year	Sex	N	LOD	LOQ	% below limit	P50	P50_95% CI	P75	P75_95% CI	P90	P90_95% CI	P95	P95_95% CI	GM	GM_95% CI
3-5	mU	DE	GerES IV 2005	2005	F,M	50		0.25	0.00	4.87	3.80 - 6.61	7.67	6.49 - 10.7	13.4	8.13 - 39.3	25.9		4.76	3.71 - 6.11
3-5	sU	DK	CPH-MC	2006	F,M	189	0.31		0.53	4.60	3.82 - 5.22	8.12	6.90 - 10.3	15.2	11.7 - 22.0	25.2	17.8 - 38.4	4.16	3.58 - 4.85
3-5	mU	DE	GerES V	2015 - 2017	F,M	388		0.50	13.9	1.30	1.20 - 1.40	2.10	1.90 - 2.49	4.20	3.60 - 4.89	5.79	4.83 - 7.62	1.28	1.17 - 1.40
3-5	sU	SK	Children cohort	2015 - 2016	F,M	67		0.50	0.00	1.63		2.85		5.09		9.04			
3-5	mU	CZ	CzechHBM-CE	2016 - 2017	F,M	148		2.00	43.9	2.24		3.16	2.83 - 3.76	4.62	4.03 - 5.42	5.47	4.75 - 7.33	2.21	2.03 - 2.41
6-11	mU	DE	GerES IV 2005	2005	F,M	102		0.25	0.00	6.72	5.72 - 8.01	9.77	8.66 - 11.4	16.1	11.9 - 23.5	21.6	17.0 - 39.8	6.16	5.24 - 7.23
6-11	sU	DK	CPH-MC	2006 - 2007	F,M	659	0.31		1.06	4.20	3.85 - 4.54	7.46	6.89 - 8.12	12.3	11.3 - 13.7	18.6	15.0 - 22.1	4.00	3.72 - 4.31
6-11	mU	DK	CPHPUB-Cross	2006 - 2008	F,M	913	0.14		0.00	5.03	4.73 - 5.33	8.28	7.84 - 9.08	13.5	12.6 - 14.2	16.9	15.4 - 20.7	4.99	4.74 - 5.26
6-11	sU	AT	PBAT	2010 - 2012	F,M	165	0.69	1.38	61.2			2.41	1.94 - 2.85	4.82	3.34 - 7.02	7.08	5.82 - 10.7		
6-11	mU	SE	DEMOCOPHES-SE	2011 - 2012	F,M	98		0.30	1.02	2.83	1.99 - 3.23	5.06	3.69 - 6.52	7.99	6.53 - 12.9	12.3	8.18 - 19.9	2.71	2.28 - 3.22
6-11	mU	DK	DEMOCOPHES-DK	2011	F,M	143		0.14	4.20	2.01	1.73 - 2.37	3.54	3.08 - 4.67	6.18	5.06 - 9.69	9.88	6.72 - 15.2	1.94	1.62 - 2.34
6-11	mU	ES	DEMOCOPHES-ES	2011 - 2012	F,M	120		0.50	0.00	6.60	5.47 - 7.60	10.1	8.81 - 12.9	15.1	13.6 - 19.8	19.5	15.3 - 25.6	6.22	5.45 - 7.11
6-11	mU	SL	DEMOCOPHES-SI	2011 - 2012	F,M	119		0.50	4.20	2.40	2.04 - 2.90	4.30	3.50 - 5.07	7.52	5.58 - 9.17	9.03	7.79 - 26.1	2.43	2.07 - 2.85
6-11	mU	LU	DEMOCOPHES-LU	2011 - 2012	F,M	60		0.50	8.33	1.70	1.30 - 2.00	2.38	2.00 - 3.47	4.00	2.99 - 4.86	4.71		1.55	1.29 - 1.86
6-11	mU	DE	DEMOCOPHES-DE	2011	F,M	119		0.50	6.72	2.32	1.92 - 2.90	4.59	3.61 - 5.84	7.17	6.00 - 8.97	8.58	7.30 - 31.6	2.41	2.04 - 2.85
6-11	mU	BE	DEMOCOPHES-BE	2011 - 2012	F,M	129		0.50	6.20	2.10	1.80 - 2.60	3.60	3.04 - 4.80	8.38	5.11 - 13.0	13.0	8.94 - 21.5	2.27	1.93 - 2.67
6-11	mU	HU	DEMOCOPHES-HU	2011 - 2012	F,M	120		2.00	25.8	3.59	3.21 - 4.35	6.54	5.41 - 8.37	11.4	8.55 - 23.2	22.2	11.8 - 39.0	3.59	3.02 - 4.28
6-11	mU	PL	DEMOCOPHES-PL	2011 - 2012	F,M	120		0.50	2.50	3.65	3.10 - 4.50	6.00	5.20 - 7.94	8.50	8.10 - 12.5	11.2	8.70 - 15.7	3.45	2.98 - 4.00
6-11	mU	CZ	DEMOCOPHES-CZ	2011 - 2012	F,M	120		2.00	27.5	3.57	2.64 - 3.94	5.56	4.44 - 7.15	8.77	7.52 - 11.0	11.0	9.06 - 19.7	3.35	2.94 - 3.83
6-11	mU	SK	DEMOCOPHES-SK	2011 - 2012	F,M	129		2.00	21.7	4.20	3.59 - 5.15	7.85	6.50 - 9.51	14.0	11.2 - 18.8	18.6	14.4 - 26.6	4.17	3.58 - 4.86
6-11	mU	SK	PCBcohort_c	2014 - 2016	F,M	249	0.33		0.00	4.36	3.73 - 5.09	7.77	6.81 - 9.19	13.4	12.1 - 15.9	18.0	14.8 - 23.8	3.94	3.46 - 4.48
6-11	mU	DE	GerES V	2015 - 2017	F,M	937		0.50	14.3	1.40	1.30 - 1.50	2.50	2.40 - 2.70	4.34	3.80 - 4.94	6.00	5.51 - 7.53	1.39	1.31 - 1.47
6-11	sU	SK	Children cohort	2015 - 2016	F,M	112		0.50	0.00	2.46	1.83 - 3.13	5.17	3.89 - 6.77	10.7	7.92 - 11.9	11.8	10.7 - 21.8	2.53	2.10 - 3.05
6-11	mU	CZ	CzechHBM-CE	2016 - 2017	F,M	223		2.00	33.2	2.55	2.24 - 2.85	4.27	3.81 - 4.76	6.38	5.45 - 7.57	8.03	6.90 - 9.83	2.62	2.40 - 2.85
12-19	mU	DE	GerES IV 2005	2005	F,M	51		0.25	1.96	6.75	5.44 - 7.06	8.21	7.05 - 12.0	12.8	10.0 - 46.7	17.6		6.68	5.46 - 8.18
12-19	mU	DK	CPHPUB-Cross	2006 - 2008	F,M	389	0.14		0.00	4.54	4.17 - 4.96	7.46	6.71 - 8.62	13.6	11.7 - 15.9	17.7	15.9 - 26.3	4.59	4.21 - 5.02
12-19	sU	DK	DYMS 2007	2007	M	239	0.14	0.14	0.84	4.48	3.84 - 4.99	8.25	6.90 - 10.1	13.7	11.6 - 18.1	19.4	16.8 - 27.7	4.22	3.71 - 4.80
12-19	sU	DK	DYMS 2008	2008	M	261	0.14	0.14	0.77	3.91	3.40 - 4.28	6.29	5.65 - 7.47	11.3	9.51 - 15.3	17.6	13.1 - 42.0	3.62	3.17 - 4.14
12-19	mU	BE	FLEHS2RefAdo	2008 - 2009	F,M	210	1.00		9.52	3.75	3.24 - 4.36	5.90	5.30 - 7.60	10.0	9.10 - 14.6	17.0	12.4 - 27.8	3.66	3.23 - 4.15
12-19	sU	DK	DYMS 2009	2009	M	210	0.07	0.07	0.48	2.71	2.34 - 3.25	5.60	4.57 - 6.28	7.90	7.23 - 10.5	12.0	9.43 - 15.9	2.63	2.29 - 3.02
12-19	sU	AT	PBAT	2010 - 2012	F,M	94	0.69	1.38	51.1			3.35	2.23 - 4.49	4.74	4.49 - 10.8	8.97	4.94 - 14.6		
12-19	sU	DK	DYMS 2013	2013	M	70	0.07	0.07	1.43	1.90	1.36 - 2.46	3.18	2.63 - 3.83	4.04	3.66 - 8.78	6.00		1.67	1.31 - 2.12
12-19	sU	BE	FLEHS3RefAdo	2013	F,M	207		0.50	3.86	2.02	1.78 - 2.34	3.80	3.16 - 4.49	6.30	5.51 - 8.06	8.66	7.42 - 11.9	2.09	1.86 - 2.35
12-19	mU	DE	GerES V	2015 - 2017	F,M	932		0.50	13.8	1.50	1.40 - 1.60	2.73	2.54 - 2.90	4.39	4.00 - 4.90	6.34	5.44 - 7.30	1.45	1.37 - 1.54

Age	Matrix	Country	Data collection	Sampling year	Sex	N	LOD	LOQ	% below limit	P50	P50_95% CI	P75	P75_95% CI	P90	P90_95% CI	P95	P95_95% CI	GM	GM_95% CI
12-19	mU	SK	PCBcohort_t	2015 - 2017	F,M	65	0.33		13.8	3.57	1.87 - 4.85	6.16	5.06 - 7.90	9.12	7.37 - 14.8	13.0		2.39	1.75 - 3.27
12-19	sU	DK	DYMS 2017	2017	M	76	0.07	0.07	3.95	0.94	0.79 - 1.21	1.71	1.28 - 2.24	2.99	2.19 - 5.71	4.74	3.01 - 9.79	0.90	0.70 - 1.14
20-39	24hU	DE	ESB 2007	2007	F,M	60		0.50	0.00	3.60	3.10 - 5.49	8.25	5.89 - 10.8	13.3	8.70 - 28.9	23.0		4.34	3.45 - 5.46
20-39	sU	DK	DYMS 2008	2008	M	54	0.14	0.14	0.00	4.79	3.97 - 6.13	8.23	6.15 - 12.3	14.3	10.2 - 17.2	15.9		4.69	3.71 - 5.92
20-39	mU	BE	FLEHS2RefAdult	2008 - 2009	F,M	189	1.00		14.8	2.70	2.30 - 3.40	5.20	4.50 - 6.92	9.30	7.90 - 13.9	14.0	10.8 - 23.3	2.76	2.38 - 3.19
20-39	24hU	DE	ESB 2008	2008	F,M	54		0.50	7.41	3.29	2.85 - 4.08	4.72	4.09 - 6.54	6.95	5.76 - 13.1	12.3		3.13	2.51 - 3.90
20-39	sU	DK	DYMS 2009	2009	M	79	0.07	0.07	1.27	3.28	2.90 - 4.59	6.18	4.91 - 8.05	10.4	7.48 - 15.1	14.2	10.4 - 142	3.24	2.54 - 4.13
20-39	24hU	DE	ESB 2009	2009	F,M	60		0.50	0.00	3.35	2.60 - 4.20	4.72	4.20 - 6.79	8.12	6.09 - 19.1	10.8		3.58	3.02 - 4.23
20-39	sU	AT	PBAT	2010 - 2011	F,M	135	0.69	1.38	79.3					2.54	1.72 - 3.49	3.48	2.65 - 7.94		
20-39	mU	SE	DEMOCOPHES-SE	2011 - 2012	F	50		0.30	4.00	3.10	2.06 - 4.07	5.14	4.05 - 6.47	7.06	6.16 - 38.2	18.6		2.68	1.97 - 3.65
20-39	mU	DK	DEMOCOPHES-DK	2011	F	51		0.14	11.8	1.79	1.27 - 2.43	3.00	2.37 - 4.44	4.83	3.44 - 10.5	6.62		1.42	1.01 - 1.98
20-39	mU	SL	DEMOCOPHES-SI	2011 - 2012	F	63		0.50	3.17	4.80	3.20 - 6.57	8.15	6.60 - 15.1	21.8	12.2 - 33.3	26.0		4.44	3.35 - 5.87
20-39	mU	DE	DEMOCOPHES-DE	2011	F	64		0.50	15.6	3.18	2.44 - 4.12	5.55	4.26 - 7.75	9.16	7.02 - 20.4	17.8		2.69	1.99 - 3.62
20-39	mU	BE	DEMOCOPHES-BE	2011 - 2012	F	62		0.50	9.68	2.05	1.38 - 2.81	4.22	2.99 - 5.23	7.62	4.90 - 24.5	11.9		2.05	1.54 - 2.72
20-39	24hU	DE	ESB 2011	2011	F,M	60		0.50	3.33	1.80	1.40 - 2.10	2.58	2.10 - 3.47	3.85	3.16 - 5.68	4.82		1.69	1.43 - 2.00
20-39	mU	HU	DEMOCOPHES-HU	2011 - 2012	F	89		2.00	22.5	4.61	3.18 - 5.33	7.45	5.69 - 10.6	13.6	10.6 - 19.8	18.6	13.8 - 56.8	4.13	3.40 - 5.02
20-39	mU	PL	DEMOCOPHES-PL	2011 - 2012	F	88		0.50	1.14	4.85	3.44 - 5.98	8.22	6.91 - 12.1	14.0	12.0 - 40.5	25.8	14.0 - 57.1	4.54	3.68 - 5.61
20-39	mU	CZ	DEMOCOPHES-CZ	2011 - 2012	F	88		2.00	23.9	3.48	2.96 - 4.19	6.03	4.33 - 7.18	7.83	7.10 - 13.2	12.4	7.86 - 14.2	3.41	2.95 - 3.95
20-39	mU	SK	DEMOCOPHES-SK	2011 - 2012	F	91		2.00	19.8	4.06	3.16 - 4.94	7.04	5.80 - 9.31	13.5	9.26 - 18.9	17.8	13.6 - 33.6	4.14	3.47 - 4.94
20-39	sU	IL	IBS	2011	F,M	144	0.25	0.50	0.00	12.3	10.4 - 15.7	25.3	19.5 - 37.7	51.0	44.9 - 60.9	61.2	52.4 - 239	13.7	11.4 - 16.4
20-39	24hU	DE	ESB 2013	2013	F,M	60		0.50	5.00	1.35	1.30 - 1.80	2.30	1.88 - 3.30	3.33	2.80 - 4.93	4.42		1.45	1.21 - 1.73
20-39	24hU	DE	ESB 2015	2015	F,M	60		0.50	10.0	1.10	1.00 - 1.20	1.92	1.36 - 2.81	3.21	2.36 - 8.16	4.42		1.22	1.00 - 1.49
20-39	sU	CZ	CzechHBM-AE	2018 - 2019	F,M	194		0.61	3.09	1.89	1.67 - 2.29	3.27	2.99 - 4.01	5.66	4.55 - 9.46	11.5	7.05 - 24.0	2.15	1.90 - 2.44
40-59	sU	AT	PBAT	2010 - 2011	F,M	112	0.69	1.38	80.4					2.19	1.77 - 3.36	3.14	2.22 - 5.72		
40-59	mU	DK	DEMOCOPHES-DK	2011	F	94		0.14	7.45	1.56	1.24 - 2.28	3.48	2.94 - 4.51	5.58	4.53 - 7.58	6.84	5.69 - 153	1.59	1.23 - 2.06
40-59	mU	ES	DEMOCOPHES-ES	2011 - 2012	F	75		0.50	1.33	6.30	5.30 - 7.80	13.9	8.80 - 18.6	21.4	17.7 - 28.0	25.8	21.5 - 35.7	6.97	5.76 - 8.44
40-59	mU	SL	DEMOCOPHES-SI	2011 - 2012	F	57		0.50	3.51	2.80	2.25 - 3.81	5.30	3.98 - 8.24	9.74	6.15 - 13.3	10.2		2.90	2.29 - 3.66
40-59	mU	DE	DEMOCOPHES-DE	2011	F	56		0.50	19.6	1.45	1.01 - 2.25	3.53	2.28 - 5.05	5.62	4.38 - 12.1	9.32		1.48	1.10 - 2.00
40-59	mU	BE	DEMOCOPHES-BE	2011 - 2012	F	67		0.50	5.97	2.40	2.01 - 2.70	4.20	2.93 - 5.13	7.04	4.93 - 8.33	7.88		2.27	1.86 - 2.77
40-59	sU	IL	IBS	2011	F,M	86	0.25	0.50	0.00	10.6	8.24 - 12.5	25.1	15.2 - 33.2	41.5	32.6 - 66.8	62.1	43.5 - 193	10.3	8.02 - 13.2
40-59	sU	CZ	CzechHBM-AE	2018 - 2019	F,M	192		0.61	2.08	1.97	1.78 - 2.39	3.46	2.87 - 4.48	7.46	5.37 - 15.3	20.8	11.8 - 37.9	2.36	2.04 - 2.72
6-59	mU	AT	IC-HBM	2009	F	50	0.22	0.81	36.0	1.20	0.32 - 1.43	2.38	1.40 - 3.58	4.04	3.10 - 10.3	5.38		1.15	0.86 - 1.55
60+	sU	AT	PBAT	2010 - 2012	F,M	89	0.69	1.38	79.8					3.41	1.60 - 6.30	5.86	3.51 - 10.6		

Notes. Matrix: mU - first morning urine, sU - spot urine, 24hU - 24-hours urine; country: AT - Austria, BE - Belgium, CZ - Czech Republic, DE - Germany, DK - Denmark, ES - Spain, HU - Hungary, IL - Israel, LU - Luxemburg, NO - Norway, PL - Poland, SE - Sweden, SI - Slovenia, SK - Slovakia; sex: F - female, M - male; n - sample size; LOD - limit of detection; LOQ - limit of quantification; %

Age	Matrix	Country	Data collection	Sampling year	Sex	N	LOD	LOQ	% below limit	P50	P50_95% CI	P75	P75_95% CI	P90	P90_95% CI	P95	P95_95% CI	GM	GM_95% CI

below limit - percentage of values below limit of detection/quantification, whichever was applied in the data collection; P50, P75, P90, P95 - 50th, 75th, 90th, 95th percentile; 95% CI - 95 % confidence interval; GM - geometric mean; NA - Not available.

Data only displayed if n was at least 50; percentiles missing when below LOD/LOQ; CIs of the observed percentiles missing when they fall outside the observed range; GM was imputed (fixed imputation) based on a log-normal distribution when at least 50 % of values were above LOD/LOQ.

Table S3: DEHP metabolite 5OH-MEHP (Mono(2-ethyl-5-hydroxy- hexyl) phthalate) in microg/L

Age	Matrix	Country	Data collection	Sampling year	Sex	N	LOD	LOQ	% below limit	P50	P50_95% CI	P75	P75_95% CI	P90	P90_95% CI	P95	P95_95% CI	GM	GM_95% CI
3-5	mU	DE	GerES IV 2005	2005	F,M	50		0.25	0.00	57.6	43.8 - 69.1	78.3	67.4 - 103	123	86.6 - 273	181		51.0	40.9 - 63.6
3-5	sU	DK	CPH-MC	2006	F,M	189	0.60		0.00	35.8	28.2 - 43.1	62.8	52.6 - 74.6	105	91.9 - 128	134	116 - 390	31.8	27.3 - 37.1
3-5	mU	DE	GerES V	2015 - 2017	F,M	388		0.20	0.00	13.6	12.4 - 15.0	22.9	21.1 - 25.5	40.2	34.8 - 48.7	55.0	48.5 - 62.7	14.2	13.2 - 15.3
3-5	sU	SK	Children cohort	2015 - 2016	F,M	67		0.20	0.00	18.3	14.4 - 21.0	27.3	21.9 - 34.9	41.5	33.6 - 79.0	63.8		16.0	13.0 - 19.8
3-5	mU	CZ	CzechHBM-CE	2016 - 2017	F,M	148		1.50	0.00	21.0	19.2 - 22.5	35.0	29.4 - 38.0	52.8	41.4 - 66.1	66.4	56.5 - 111	21.0	18.7 - 23.6
6-11	mU	DE	GerES IV 2005	2005	F,M	102		0.25	0.00	48.0	42.2 - 52.4	75.6	61.0 - 95.9	122	99.9 - 143	133	122 - 250	48.2	42.1 - 55.1
6-11	sU	DK	CPH-MC	2006 - 2007	F,M	659	0.60		0.00	33.7	30.7 - 37.2	58.6	54.6 - 65.2	97.6	88.0 - 112	135	117 - 154	30.8	28.5 - 33.3
6-11	mU	DK	CPHPUB-Cross	2006 - 2008	F,M	913	0.91		0.00	49.7	47.3 - 52.0	76.9	73.7 - 81.2	113	106 - 124	165	146 - 194	50.2	48.0 - 52.6
6-11	sU	AT	PBAT	2010 - 2012	F,M	165	0.79	1.58	12.7	5.15	3.37 - 6.64	11.3	8.92 - 16.0	23.2	18.6 - 27.2	27.5	24.7 - 36.5	5.23	4.46 - 6.12
6-11	mU	SE	DEMOCOPHES-SE	2011 - 2012	F,M	98		0.30	0.00	24.2	20.5 - 28.0	36.2	30.6 - 47.0	53.9	47.7 - 125	111	54.7 - 145	24.1	20.9 - 27.9
6-11	mU	DK	DEMOCOPHES-DK	2011	F,M	143		0.91	0.00	22.8	20.5 - 28.6	36.6	31.5 - 41.3	49.8	43.7 - 88.5	88.6	50.5 - 133	23.3	20.6 - 26.4
6-11	mU	ES	DEMOCOPHES-ES	2011 - 2012	F,M	120		0.20	0.00	32.9	28.3 - 40.5	56.5	50.2 - 66.8	93.3	73.7 - 132	132	94.3 - 162	34.8	30.6 - 39.6
6-11	mU	SL	DEMOCOPHES-SI	2011 - 2012	F,M	119		0.50	0.00	29.0	23.0 - 35.2	46.5	41.0 - 53.7	64.0	57.7 - 80.7	79.3	65.0 - 178	28.4	24.9 - 32.4
6-11	mU	LU	DEMOCOPHES-LU	2011 - 2012	F,M	60		0.10	0.00	14.0	12.0 - 17.0	20.5	17.8 - 25.7	26.5	24.6 - 40.9	36.1		12.4	10.2 - 15.1
6-11	mU	DE	DEMOCOPHES-DE	2011	F,M	119		0.20	0.00	21.2	18.3 - 24.5	34.0	29.9 - 40.0	50.7	40.9 - 61.2	60.1	52.1 - 271	21.9	19.1 - 25.0
6-11	mU	BE	DEMOCOPHES-BE	2011 - 2012	F,M	129		0.10	0.00	16.0	14.0 - 21.0	30.0	25.0 - 38.6	65.4	44.9 - 106	103	74.9 - 178	18.8	15.9 - 22.1
6-11	mU	HU	DEMOCOPHES-HU	2011 - 2012	F,M	120		0.61	0.00	33.3	26.5 - 38.7	54.2	47.6 - 70.7	93.3	75.3 - 155	155	98.2 - 286	32.9	28.3 - 38.3
6-11	mU	PL	DEMOCOPHES-PL	2011 - 2012	F,M	120		0.10	0.00	39.0	36.9 - 44.0	54.0	48.0 - 66.9	106	71.4 - 138	135	110 - 190	40.0	35.4 - 45.3
6-11	mU	CZ	DEMOCOPHES-CZ	2011 - 2012	F,M	120		0.61	0.00	33.1	29.4 - 38.8	60.9	46.6 - 75.5	91.9	78.2 - 121	114	97.3 - 246	37.0	32.5 - 42.0
6-11	mU	SK	DEMOCOPHES-SK	2011 - 2012	F,M	129		0.61	0.00	51.2	42.7 - 59.0	85.2	72.5 - 96.9	125	103 - 165	159	129 - 257	50.3	44.2 - 57.2
6-11	mU	NO	IES	2012	F,M	50	0.20	0.50	0.00	21.4	17.8 - 24.7	33.1	24.4 - 37.5	41.0	35.9 - 53.4	44.1		20.9	17.9 - 24.3
6-11	mU	SK	PCBcohort_c	2014 - 2016	F,M	249	0.48		0.00	26.9	25.0 - 29.3	39.7	34.6 - 44.5	58.1	52.8 - 68.5	75.8	61.7 - 102	26.9	24.8 - 29.1
6-11	mU	DE	GerES V	2015 - 2017	F,M	937		0.20	0.11	12.0	11.4 - 12.7	18.1	17.3 - 19.2	28.6	26.3 - 31.7	39.1	35.5 - 44.8	12.0	11.5 - 12.6
6-11	sU	SK	Children cohort	2015 - 2016	F,M	112		0.20	0.00	20.0	15.6 - 22.9	35.1	29.2 - 40.7	51.3	42.6 - 82.8	78.0	51.7 - 123	19.4	16.5 - 22.8
6-11	mU	CZ	CzechHBM-CE	2016 - 2017	F,M	223		1.50	0.00	21.9	18.8 - 23.9	34.7	28.1 - 38.5	52.1	44.0 - 62.8	65.6	57.1 - 81.7	20.5	18.6 - 22.5
12-19	mU	DE	GerES IV 2005	2005	F,M	51		0.25	0.00	37.6	33.7 - 44.9	54.4	43.7 - 86.9	91.1	64.5 - 264	169		41.1	32.8 - 51.6
12-19	mU	DK	CPHPUB-Cross	2006 - 2008	F,M	389	0.91		0.00	38.8	35.5 - 41.8	66.1	61.2 - 71.5	99.4	90.1 - 117	130	117 - 204	39.6	36.4 - 42.9
12-19	sU	DK	DYMS 2007	2007	M	239	0.91	0.91	0.42	25.2	22.0 - 28.6	38.3	35.0 - 44.9	59.7	53.2 - 85.1	90.4	70.5 - 163	23.1	20.5 - 26.0
12-19	sU	DK	DYMS 2008	2008	M	261	0.91	0.91	0.77	20.9	18.4 - 24.1	33.1	29.1 - 36.4	54.6	45.6 - 71.1	81.5	61.0 - 243	20.3	17.9 - 23.1
12-19	mU	BE	FLEHS2RefAdo	2008 - 2009	F,M	210	0.10		0.00	22.0	18.0 - 24.0	32.8	30.5 - 38.0	51.1	43.6 - 61.6	68.8	57.8 - 103	21.6	19.5 - 24.0
12-19	sU	DK	DYMS 2009	2009	M	210	0.05	0.05	0.00	17.9	16.0 - 20.2	30.9	25.1 - 35.0	55.6	40.8 - 59.3	64.8	57.8 - 72.5	16.3	14.3 - 18.6
12-19	sU	AT	PBAT	2010 - 2012	F,M	94	0.79	1.58	14.9	4.38	3.10 - 5.67	11.6	7.24 - 17.8	22.7	18.0 - 26.9	26.4	23.4 - 52.6	4.88	3.94 - 6.05
12-19	sU	DK	DYMS 2013	2013	M	70	0.05	0.05	0.00	9.34	6.99 - 10.5	13.9	10.8 - 17.7	22.7	16.1 - 32.4	28.5		7.82	6.15 - 9.92
12-19	sU	BE	FLEHS3RefAdo	2013	F,M	207		0.10	0.00	8.38	7.42 - 9.54	13.2	11.7 - 15.1	20.6	17.0 - 25.2	28.9	21.6 - 35.6	8.36	7.56 - 9.25

Age	Matrix	Country	Data collection	Sampling year	Sex	N	LOD	LOQ	% below limit	P50	P50_95% CI	P75	P75_95% CI	P90	P90_95% CI	P95	P95_95% CI	GM	GM_95% CI
12-19	mU	DE	GerES V	2015 - 2017	F,M	932		0.20	0.00	8.50	8.16 - 9.00	13.3	12.8 - 14.3	22.7	20.7 - 24.3	28.7	26.6 - 35.3	8.70	8.29 - 9.12
12-19	mU	SK	PCBcohort_t	2015 - 2017	F,M	65	0.48		0.00	16.7	12.5 - 20.0	25.7	21.3 - 38.7	43.6	33.7 - 71.3	60.7		15.2	12.3 - 18.9
12-19	sU	DK	DYMS 2017	2017	M	76	0.05	0.05	0.00	5.67	4.79 - 6.76	8.60	7.36 - 10.1	11.7	9.50 - 33.1	22.9	11.8 - 54.5	5.04	4.09 - 6.21
20-39	24hU	DE	ESB 2007	2007	F,M	60		0.20	0.00	10.8	8.30 - 12.7	19.4	14.1 - 26.8	30.0	26.1 - 121	41.5		11.7	9.29 - 14.6
20-39	sU	DK	DYMS 2008	2008	M	54	0.91	0.91	0.00	26.8	20.3 - 31.3	41.7	31.3 - 57.5	60.4	49.5 - 71.9	68.2		23.8	19.3 - 29.3
20-39	mU	BE	FLEHS2RefAdult	2008 - 2009	F,M	189	0.10		0.00	14.0	12.0 - 16.0	24.0	20.0 - 27.0	42.0	33.2 - 51.0	54.0	44.0 - 105	12.8	11.1 - 14.9
20-39	24hU	DE	ESB 2008	2008	F,M	54		0.50	0.00	9.30	7.76 - 10.7	14.3	10.7 - 19.0	19.9	17.3 - 25.0	22.3		9.49	8.18 - 11.0
20-39	sU	DK	DYMS 2009	2009	M	79	0.05	0.05	0.00	19.9	16.8 - 24.5	34.4	26.5 - 40.4	47.2	38.9 - 69.5	58.3	47.6 - 1065	19.8	16.1 - 24.4
20-39	24hU	DE	ESB 2009	2009	F,M	60		0.20	0.00	8.80	6.22 - 10.9	13.8	11.1 - 19.9	21.9	18.3 - 47.3	34.8		8.52	6.76 - 10.8
20-39	sU	AT	PBAT	2010 - 2011	F,M	135	0.79	1.58	45.9	1.76		2.94	2.52 - 3.82	6.40	4.67 - 7.55	7.54	6.85 - 22.1	2.02	1.76 - 2.32
20-39	mU	SE	DEMOCOPHES-SE	2011 - 2012	F	50		0.30	0.00	12.4	10.6 - 19.5	25.9	19.1 - 33.5	37.0	29.3 - 336	66.8		14.6	11.0 - 19.5
20-39	mU	DK	DEMOCOPHES-DK	2011	F	51		0.91	0.00	12.8	11.1 - 18.5	21.6	18.5 - 30.1	36.4	25.5 - 68.7	58.7		13.6	10.8 - 17.0
20-39	mU	SL	DEMOCOPHES-SI	2011 - 2012	F	63		0.50	0.00	21.0	18.0 - 26.3	37.0	27.0 - 55.3	59.8	54.0 - 138	127		21.3	16.8 - 27.0
20-39	mU	DE	DEMOCOPHES-DE	2011	F	64		0.20	0.00	12.4	10.7 - 15.4	21.6	15.7 - 27.0	33.0	25.0 - 50.4	41.6		13.2	11.0 - 15.8
20-39	mU	BE	DEMOCOPHES-BE	2011 - 2012	F	62		0.10	0.00	11.0	8.33 - 14.2	21.0	16.0 - 41.0	50.9	33.0 - 65.9	57.9		11.6	9.07 - 14.9
20-39	24hU	DE	ESB 2011	2011	F,M	60		0.20	0.00	7.45	6.31 - 9.10	10.6	9.10 - 12.8	14.4	12.1 - 20.3	18.3		7.56	6.62 - 8.64
20-39	mU	HU	DEMOCOPHES-HU	2011 - 2012	F	89		0.61	0.00	20.5	18.0 - 27.2	34.0	28.4 - 48.8	61.3	48.0 - 80.5	78.1	62.0 - 163	20.1	16.6 - 24.2
20-39	mU	PL	DEMOCOPHES-PL	2011 - 2012	F	88		0.10	0.00	22.5	17.4 - 30.0	39.2	33.0 - 50.0	60.4	49.4 - 123	89.0	63.5 - 218	22.4	18.6 - 27.1
20-39	mU	CZ	DEMOCOPHES-CZ	2011 - 2012	F	88		0.61	0.00	17.3	14.6 - 19.7	26.8	21.9 - 42.3	55.5	41.9 - 66.5	62.7	55.8 - 80.5	17.8	15.2 - 20.8
20-39	mU	SK	DEMOCOPHES-SK	2011 - 2012	F	91		0.61	0.00	24.0	17.2 - 27.7	36.9	30.0 - 46.8	61.3	46.7 - 87.7	73.7	62.2 - 122	21.9	18.5 - 26.0
20-39	sU	IL	IBS	2011	F,M	144	0.25	0.50	0.00	29.3	25.7 - 36.5	52.2	44.8 - 61.3	86.7	65.6 - 102	102	88.5 - 251	29.2	25.3 - 33.7
20-39	24hU	DE	ESB 2013	2013	F,M	60		0.20	0.00	5.70	4.70 - 7.39	8.62	7.40 - 11.7	12.3	10.8 - 15.4	14.8		5.97	5.20 - 6.86
20-39	24hU	DE	ESB 2015	2015	F,M	60		0.20	0.00	4.20	3.50 - 5.39	6.95	5.48 - 8.37	11.2	7.93 - 18.5	17.9		4.56	3.77 - 5.51
20-39	sU	CZ	CzechHBM-AE	2018 - 2019	F,M	194		2.84	33.0	4.22	3.48 - 4.90	7.48	6.39 - 9.95	16.2	11.6 - 22.0	23.7	17.8 - 40.2	4.17	3.62 - 4.81
40-59	sU	AT	PBAT	2010 - 2011	F,M	112	0.79	1.58	50.9			2.79	2.42 - 3.97	6.82	4.10 - 10.6	10.5	6.89 - 16.5		
40-59	mU	DK	DEMOCOPHES-DK	2011	F	94		0.91	0.00	11.5	10.1 - 14.9	21.6	17.1 - 26.0	33.4	26.2 - 46.7	42.0	34.9 - 346	12.9	10.8 - 15.4
40-59	mU	ES	DEMOCOPHES-ES	2011 - 2012	F	75		0.20	0.00	19.9	16.0 - 27.9	40.4	30.1 - 52.9	68.8	51.6 - 79.3	74.5	69.1 - 94.3	20.8	16.9 - 25.4
40-59	mU	SL	DEMOCOPHES-SI	2011 - 2012	F	57		0.50	0.00	14.0	10.7 - 19.0	22.0	19.0 - 34.3	36.4	30.1 - 46.5	44.2		14.2	11.7 - 17.3
40-59	mU	DE	DEMOCOPHES-DE	2011	F	56		0.20	0.00	8.79	6.86 - 12.0	16.9	12.2 - 24.6	25.4	19.8 - 37.4	33.5		8.99	7.29 - 11.1
40-59	mU	BE	DEMOCOPHES-BE	2011 - 2012	F	67		0.10	0.00	10.0	9.00 - 13.0	16.0	13.0 - 18.3	20.0	18.0 - 29.4	26.8		10.2	8.88 - 11.8
40-59	sU	IL	IBS	2011	F,M	86	0.25	0.50	0.00	33.6	26.0 - 41.3	55.8	46.3 - 66.3	93.0	66.0 - 116	109	94.6 - 264	29.9	24.5 - 36.5
40-59	sU	CZ	CzechHBM-AE	2018 - 2019	F,M	192		2.84	24.5	5.07	4.33 - 6.05	9.82	7.75 - 10.9	22.1	14.1 - 34.9	39.1	29.0 - 76.3	5.38	4.62 - 6.26
6-59	mU	AT	IC-HBM	2009	F	50	0.06	0.24	2.00	3.00	2.27 - 3.50	4.53	3.49 - 7.49	8.04	5.62 - 43.4	17.9		2.89	2.14 - 3.90
6-59	mU	AT	IC-HBM	2009	F,M	50	0.06	0.24	0.00	4.60	3.77 - 5.63	6.90	5.59 - 7.85	8.00	7.30 - 22.5	8.87		4.60	3.90 - 5.42
60+	sU	AT	PBAT	2010 - 2012	F,M	89	0.79	1.58	24.7	2.80	2.39 - 3.40	4.84	3.90 - 9.33	14.8	9.28 - 28.9	22.0	15.1 - 67.3	3.26	2.64 - 4.04

Age	Matrix	Country	Data collection	Sampling year	Sex	N	LOD	LOQ	% below limit	P50	P50_95% CI	P75	P75_95% CI	P90	P90_95% CI	P95	P95_95% CI	GM	GM_95% CI

Notes. Matrix: mU - first morning urine, sU - spot urine, 24hU - 24-hours urine; country: AT - Austria, BE - Belgium, CZ - Czech Republic, DE - Germany, DK - Denmark, ES - Spain, HU - Hungary, IL - Israel, LU - Luxemburg, NO - Norway, PL - Poland, SE - Sweden, SI - Slovenia, SK - Slovakia; sex: F - female, M - male; n - sample size; LOD - limit of detection; LOQ - limit of quantification; % below limit - percentage of values below limit of detection/quantification, whichever was applied in the data collection; P50, P75, P90, P95 - 50th, 75th, 90th, 95th percentile; 95% CI - 95 % confidence interval; GM - geometric mean; NA - Not available.

Data only displayed if n was at least 50; percentiles missing when below LOD/LOQ; CIs of the observed percentiles missing when they fall outside the observed range; GM was imputed (fixed imputation) based on a log-normal distribution when at least 50 % of values were above LOD/LOQ.

Table S4: DEHP metabolite 5oxo-MEHP (Mono(2-ethyl-5-oxo-hexyl) phthalate) in microg/L

Age	Matrix	Country	Data collection	Sampling year	Sex	N	LOD	LOQ	% below limit	P50	P50_95% CI	P75	P75_95% CI	P90	P90_95% CI	P95	P95_95% CI	GM	GM_95% CI
3-5	mU	DE	GerES IV 2005	2005	F,M	50		0.25	0.00	43.8	32.4 - 55.5	58.6	55.0 - 82.9	104	67.2 - 210	149		39.6	31.7 - 49.4
3-5	sU	DK	CPH-MC	2006	F,M	189	0.14		0.00	20.9	17.3 - 24.5	35.0	31.7 - 43.9	57.7	49.1 - 69.9	76.6	64.1 - 181	18.3	15.8 - 21.2
3-5	mU	DE	GerES V	2015 - 2017	F,M	388		0.20	0.00	9.70	8.80 - 10.8	17.1	15.3 - 19.3	29.0	26.2 - 33.1	35.9	33.0 - 48.5	10.2	9.44 - 11.0
3-5	sU	SK	Children cohort	2015 - 2016	F,M	67		0.20	0.00	12.2	9.53 - 14.5	19.1	15.0 - 23.6	29.9	22.7 - 49.3	43.3		11.1	8.97 - 13.6
3-5	mU	CZ	CzechHBM-CE	2016 - 2017	F,M	148		2.00	1.35	13.7	12.5 - 16.1	19.2	17.8 - 23.3	34.8	27.4 - 40.0	40.6	35.6 - 63.8	13.2	11.8 - 14.9
6-11	mU	DE	GerES IV 2005	2005	F,M	102		0.25	0.00	39.8	34.7 - 43.8	61.1	49.7 - 78.0	94.9	78.1 - 116	109	95.6 - 193	38.8	33.9 - 44.6
6-11	sU	DK	CPH-MC	2006 - 2007	F,M	659	0.14		0.00	16.6	15.4 - 18.7	28.6	26.1 - 30.4	43.5	39.7 - 49.1	59.7	53.6 - 76.9	15.4	14.3 - 16.5
6-11	mU	DK	CPHPUB-Cross	2006 - 2008	F,M	913	0.67		0.00	26.0	24.9 - 27.6	40.2	38.4 - 43.7	66.7	61.1 - 73.9	83.3	78.6 - 96.8	26.7	25.4 - 28.0
6-11	sU	AT	PBAT	2010 - 2012	F,M	165	0.56	1.12	10.9	3.71	2.71 - 4.86	9.14	7.17 - 12.0	17.2	13.6 - 19.3	19.7	17.9 - 25.3	4.03	3.46 - 4.70
6-11	mU	SE	DEMOCOPHES-SE	2011 - 2012	F,M	98		0.30	0.00	16.0	13.6 - 18.0	24.1	19.8 - 29.7	34.2	30.0 - 73.1	59.2	35.4 - 101	15.4	13.3 - 17.8
6-11	mU	DK	DEMOCOPHES-DK	2011	F,M	143		0.67	0.00	11.6	10.2 - 13.1	17.7	15.7 - 19.9	25.1	21.6 - 38.6	39.2	26.2 - 57.6	11.4	10.1 - 12.9
6-11	mU	ES	DEMOCOPHES-ES	2011 - 2012	F,M	120		0.20	0.00	20.6	18.3 - 26.0	35.8	28.6 - 41.4	48.3	43.9 - 76.8	70.1	49.3 - 121	22.0	19.5 - 24.9
6-11	mU	SL	DEMOCOPHES-SI	2011 - 2012	F,M	119		0.50	0.00	17.0	13.4 - 20.0	27.5	23.3 - 32.7	41.0	34.8 - 47.6	47.1	41.0 - 163	17.4	15.3 - 19.8
6-11	mU	LU	DEMOCOPHES-LU	2011 - 2012	F,M	60		0.10	0.00	10.0	8.51 - 12.0	13.0	12.0 - 17.4	20.1	16.0 - 31.6	22.4		8.79	7.29 - 10.6
6-11	mU	DE	DEMOCOPHES-DE	2011	F,M	119		0.20	0.00	14.0	11.8 - 16.2	21.7	18.8 - 25.8	33.4	27.5 - 42.3	41.9	36.0 - 186	14.4	12.6 - 16.5
6-11	mU	BE	DEMOCOPHES-BE	2011 - 2012	F,M	129		0.10	0.00	12.0	10.0 - 15.0	21.0	17.4 - 30.0	43.0	32.6 - 74.1	73.0	46.1 - 125	13.5	11.5 - 15.8
6-11	mU	HU	DEMOCOPHES-HU	2011 - 2012	F,M	120		0.24	0.00	22.7	17.4 - 28.2	38.7	33.9 - 46.5	62.7	50.5 - 102	101	72.7 - 154	22.3	19.2 - 26.0
6-11	mU	PL	DEMOCOPHES-PL	2011 - 2012	F,M	120		0.10	0.00	26.0	23.0 - 29.0	32.5	31.0 - 39.5	62.3	43.7 - 82.5	82.0	65.8 - 140	25.7	22.8 - 28.9
6-11	mU	CZ	DEMOCOPHES-CZ	2011 - 2012	F,M	120		0.24	0.00	21.9	19.3 - 27.7	42.0	33.1 - 48.9	62.1	53.3 - 83.7	81.4	66.1 - 180	24.8	21.7 - 28.2
6-11	mU	SK	DEMOCOPHES-SK	2011 - 2012	F,M	129		0.24	0.00	35.1	28.0 - 41.9	55.9	48.3 - 70.8	87.7	73.7 - 103	102	88.4 - 177	33.9	29.8 - 38.6
6-11	mU	NO	IES	2012	F,M	50	0.20	0.50	0.00	15.2	12.2 - 17.7	22.0	17.2 - 27.1	29.1	24.5 - 38.8	35.7		14.6	12.5 - 17.1
6-11	mU	SK	PCBcohort_c	2014 - 2016	F,M	249	1.08		0.00	21.8	20.4 - 23.3	32.6	29.3 - 36.4	46.4	42.5 - 56.2	61.7	51.1 - 85.8	21.8	20.1 - 23.7
6-11	mU	DE	GerES V	2015 - 2017	F,M	933		0.20	0.11	8.50	8.10 - 8.90	13.2	12.5 - 14.2	21.7	19.7 - 23.3	29.3	26.4 - 33.6	8.51	8.11 - 8.93
6-11	sU	SK	Children cohort	2015 - 2016	F,M	112		0.20	0.00	14.1	11.1 - 16.7	25.9	21.5 - 30.5	39.8	32.6 - 58.0	56.4	41.3 - 87.8	13.9	11.8 - 16.3
6-11	mU	CZ	CzechHBM-CE	2016 - 2017	F,M	223		2.00	1.35	13.5	12.2 - 14.7	20.4	18.5 - 23.9	30.7	28.8 - 39.4	41.2	36.2 - 45.4	12.8	11.6 - 14.1
12-19	mU	DE	GerES IV 2005	2005	F,M	51		0.25	0.00	27.2	25.5 - 32.9	44.6	32.3 - 68.7	72.0	47.1 - 199	106		31.0	24.8 - 38.7
12-19	mU	DK	CPHPUB-Cross	2006 - 2008	F,M	389	0.67		0.26	19.2	17.7 - 21.0	32.2	29.3 - 34.4	49.6	44.0 - 52.2	62.8	51.8 - 81.8	19.4	17.9 - 21.1
12-19	sU	DK	DYMS 2007	2007	M	239	0.67	0.67	0.42	18.1	16.0 - 19.6	27.9	25.4 - 32.5	45.6	38.7 - 60.0	66.9	50.7 - 103	16.7	14.9 - 18.8
12-19	sU	DK	DYMS 2008	2008	M	261	0.67	0.67	0.77	15.6	14.6 - 17.4	24.4	21.5 - 26.9	38.7	33.2 - 50.2	56.6	45.5 - 176	15.0	13.2 - 17.0
12-19	mU	BE	FLEHS2RefAdo	2008 - 2009	F,M	210	0.10		0.00	27.5	24.4 - 31.0	48.0	40.0 - 54.0	85.3	62.2 - 118	126	96.3 - 206	29.0	25.9 - 32.5
12-19	sU	DK	DYMS 2009	2009	M	210	0.06	0.06	0.48	8.54	7.71 - 9.70	14.0	12.5 - 17.1	23.2	20.6 - 27.3	28.0	24.1 - 38.2	7.86	6.93 - 8.93
12-19	sU	AT	PBAT	2010 - 2012	F,M	94	0.56	1.12	11.7	3.08	2.45 - 3.93	8.90	5.28 - 11.1	16.3	11.1 - 18.6	18.0	16.7 - 39.4	3.58	2.91 - 4.42
12-19	sU	DK	DYMS 2013	2013	M	70	0.06	0.06	0.00	5.73	4.76 - 7.22	9.26	7.77 - 12.6	14.9	12.2 - 21.1	19.6		5.45	4.34 - 6.85
12-19	sU	BE	FLEHS3RefAdo	2013	F,M	207		0.10	0.00	6.84	6.12 - 7.78	10.4	9.32 - 11.8	16.0	13.2 - 20.4	22.0	18.8 - 31.7	6.62	5.98 - 7.34

Age	Matrix	Country	Data collection	Sampling year	Sex	N	LOD	LOQ	% below limit	P50	P50_95% CI	P75	P75_95% CI	P90	P90_95% CI	P95	P95_95% CI	GM	GM_95% CI
12-19	mU	DE	GerES V	2015 - 2017	F,M	931		0.20	0.00	5.80	5.50 - 6.20	9.40	8.96 - 10.0	15.3	14.2 - 17.3	20.1	18.4 - 24.1	5.76	5.47 - 6.06
12-19	mU	SK	PCBcohort_t	2015 - 2017	F,M	65	1.08		1.54	18.1	15.4 - 24.0	32.4	24.8 - 42.3	50.9	37.6 - 86.0	63.0		18.6	15.1 - 23.0
12-19	sU	DK	DYMS 2017	2017	M	76	0.06	0.06	0.00	3.97	3.46 - 5.09	5.60	5.23 - 7.00	8.03	6.81 - 20.2	15.4	8.05 - 32.5	3.55	2.90 - 4.35
20-39	24hU	DE	ESB 2007	2007	F,M	60		0.20	0.00	6.25	5.10 - 8.20	10.2	8.36 - 15.9	17.4	14.4 - 64.7	22.0		6.93	5.59 - 8.59
20-39	sU	DK	DYMS 2008	2008	M	54	0.67	0.67	0.00	18.6	13.9 - 23.2	28.3	23.3 - 35.5	38.6	33.7 - 49.4	45.8		17.2	14.1 - 20.8
20-39	mU	BE	FLEHS2RefAdult	2008 - 2009	F,M	189	0.10		0.00	15.0	13.0 - 19.4	31.0	26.0 - 36.6	68.2	43.1 - 99.5	119	81.1 - 224	15.9	13.5 - 18.8
20-39	24hU	DE	ESB 2008	2008	F,M	54		0.50	0.00	5.91	5.10 - 6.83	9.71	6.83 - 12.2	13.0	11.5 - 14.7	14.1		6.27	5.43 - 7.23
20-39	sU	DK	DYMS 2009	2009	M	79	0.06	0.06	0.00	10.0	8.15 - 13.3	16.6	14.0 - 18.8	22.4	18.3 - 28.9	26.7	22.7 - 492	9.48	7.71 - 11.7
20-39	24hU	DE	ESB 2009	2009	F,M	60		0.20	0.00	5.05	3.91 - 6.29	7.12	6.46 - 9.33	12.6	8.52 - 22.9	17.5		4.74	3.80 - 5.91
20-39	sU	AT	PBAT	2010 - 2011	F,M	135	0.56	1.12	43.0	1.23		1.99	1.75 - 2.75	4.49	3.43 - 6.87	6.74	4.66 - 15.9	1.43	1.24 - 1.65
20-39	mU	SE	DEMOCOPHES-SE	2011 - 2012	F	50		0.30	0.00	7.96	6.83 - 10.6	14.0	10.4 - 19.4	22.9	17.1 - 154	45.1		8.55	6.38 - 11.5
20-39	mU	DK	DEMOCOPHES-DK	2011	F	51		0.67	1.96	6.85	5.29 - 8.41	9.82	8.31 - 15.1	16.2	14.0 - 33.6	27.5		6.47	5.12 - 8.17
20-39	mU	SL	DEMOCOPHES-SI	2011 - 2012	F	63		0.50	0.00	11.0	8.77 - 15.0	21.0	15.0 - 33.1	35.6	30.2 - 65.3	57.4		11.2	8.74 - 14.2
20-39	mU	DE	DEMOCOPHES-DE	2011	F	64		0.20	0.00	7.91	6.42 - 10.4	13.4	10.6 - 18.2	20.0	17.6 - 32.2	27.1		8.60	7.19 - 10.3
20-39	mU	BE	DEMOCOPHES-BE	2011 - 2012	F	62		0.10	0.00	7.75	5.48 - 9.82	13.0	10.2 - 24.3	34.9	22.0 - 44.8	42.0		7.98	6.25 - 10.2
20-39	24hU	DE	ESB 2011	2011	F,M	60		0.20	0.00	5.60	4.80 - 6.59	7.35	6.60 - 9.71	11.0	9.36 - 18.1	13.9		5.52	4.77 - 6.39
20-39	mU	HU	DEMOCOPHES-HU	2011 - 2012	F	89		0.24	0.00	14.3	11.3 - 17.0	24.0	19.3 - 31.9	35.9	31.7 - 60.4	53.1	37.9 - 96.8	13.6	11.3 - 16.4
20-39	mU	PL	DEMOCOPHES-PL	2011 - 2012	F	88		0.10	0.00	15.0	10.4 - 18.6	26.0	21.0 - 30.1	34.8	29.8 - 76.3	56.9	37.1 - 128	14.1	11.7 - 16.9
20-39	mU	CZ	DEMOCOPHES-CZ	2011 - 2012	F	88		0.24	0.00	11.1	9.32 - 12.9	17.4	14.5 - 25.4	32.9	25.3 - 44.2	42.3	32.9 - 54.1	11.5	9.81 - 13.4
20-39	mU	SK	DEMOCOPHES-SK	2011 - 2012	F	91		0.24	0.00	14.1	12.4 - 17.2	22.1	18.5 - 31.8	38.8	31.8 - 53.6	52.0	39.3 - 83.3	13.9	11.8 - 16.5
20-39	sU	IL	IBS	2011	F,M	144	0.25	0.50	0.00	16.2	14.5 - 19.8	32.4	24.1 - 39.8	52.6	42.2 - 61.0	61.0	56.4 - 143	16.6	14.3 - 19.2
20-39	24hU	DE	ESB 2013	2013	F,M	60		0.20	0.00	3.90	3.40 - 5.30	6.40	5.38 - 8.84	9.49	7.95 - 12.8	11.4		4.22	3.59 - 4.96
20-39	24hU	DE	ESB 2015	2015	F,M	60		0.20	0.00	3.15	2.30 - 3.70	4.88	3.78 - 5.90	6.63	5.86 - 14.1	10.6		3.13	2.59 - 3.78
20-39	sU	CZ	CzechHBM-AE	2018 - 2019	F,M	194		2.71	54.1			4.67	3.53 - 6.10	9.43	6.94 - 12.9	13.7	10.4 - 24.0		
40-59	sU	AT	PBAT	2010 - 2011	F,M	112	0.56	1.12	56.2			1.96	1.56 - 2.71	4.11	2.77 - 7.00	6.81	4.42 - 8.92		
40-59	mU	DK	DEMOCOPHES-DK	2011	F	94		0.67	0.00	5.90	4.85 - 6.70	9.19	7.67 - 11.7	15.5	11.8 - 20.3	18.5	15.6 - 202	5.87	4.89 - 7.05
40-59	mU	ES	DEMOCOPHES-ES	2011 - 2012	F	75		0.20	0.00	13.5	11.0 - 16.5	23.7	17.4 - 31.8	39.8	30.1 - 56.0	50.7	40.1 - 76.3	13.2	10.9 - 16.1
40-59	mU	SL	DEMOCOPHES-SI	2011 - 2012	F	57		0.50	0.00	7.60	5.74 - 9.20	15.0	9.20 - 17.3	18.0	17.0 - 20.8	19.2		7.47	6.18 - 9.03
40-59	mU	DE	DEMOCOPHES-DE	2011	F	56		0.20	0.00	5.82	4.57 - 7.58	10.3	7.62 - 15.0	17.5	11.6 - 22.3	21.2		5.90	4.78 - 7.28
40-59	mU	BE	DEMOCOPHES-BE	2011 - 2012	F	67		0.10	0.00	7.50	6.30 - 9.80	11.0	10.0 - 15.0	16.4	13.7 - 21.1	19.4		7.19	6.13 - 8.43
40-59	sU	IL	IBS	2011	F,M	86	0.25	0.50	0.00	17.8	15.8 - 23.5	32.6	28.7 - 41.1	54.4	39.6 - 70.5	62.7	54.9 - 162	17.8	14.6 - 21.7
40-59	sU	CZ	CzechHBM-AE	2018 - 2019	F,M	192		2.71	45.3	2.99		5.79	5.07 - 6.66	13.7	8.55 - 19.5	24.0	16.0 - 46.5	2.85	2.43 - 3.35
60+	sU	AT	PBAT	2010 - 2012	F,M	89	0.56	1.12	34.8	1.60	1.24 - 1.90	2.60	2.10 - 6.79	8.10	6.62 - 18.0	14.0	8.36 - 51.6	1.79	1.41 - 2.27

Notes. Matrix: mU - first morning urine, sU - spot urine, 24hU - 24-hours urine; country: AT - Austria, BE - Belgium, CZ - Czech Republic, DE - Germany, DK - Denmark, ES - Spain, HU - Hungary, IL - Israel, LU - Luxemburg, NO - Norway, PL - Poland, SE - Sweden, SI - Slovenia, SK - Slovakia; sex: F - female, M - male; n - sample size; LOD - limit of detection; LOQ - limit of quantification; %

Age	Matrix	Country	Data collection	Sampling year	Sex	N	LOD	LOQ	% below limit	P50	P50_95% CI	P75	P75_95% CI	P90	P90_95% CI	P95	P95_95% CI	GM	GM_95% CI

below limit - percentage of values below limit of detection/quantification, whichever was applied in the data collection; P50, P75, P90, P95 - 50th, 75th, 90th, 95th percentile; 95% CI - 95 % confidence interval; GM - geometric mean; NA - Not available.

Data only displayed if n was at least 50; percentiles missing when below LOD/LOQ; CIs of the observed percentiles missing when they fall outside the observed range; GM was imputed (fixed imputation) based on a log-normal distribution when at least 50 % of values were above LOD/LOQ.

Table S5: DEHP metabolite 5cx-MEPP (Mono(2-ethyl-5-carboxy- pentyl) phthalate) in microg/L

Age	Matrix	Country	Data collection	Sampling year	Sex	N	LOD	LOQ	% below limit	P50	P50_95% CI	P75	P75_95% CI	P90	P90_95% CI	P95	P95_95% CI	GM	GM_95% CI
3-5	mU	DE	GerES IV 2005	2005	F,M	50		0.25	0.00	74.6	62.6 - 94.5	105	93.2 - 158	174	126 - 444	290		73.0	58.6 - 91.0
3-5	sU	DK	CPH-MC	2006	F,M	189	0.43		0.00	29.3	25.1 - 36.0	55.0	48.9 - 66.8	104	78.8 - 134	137	120 - 272	30.6	26.7 - 35.1
3-5	mU	DE	GerES V	2015 - 2017	F,M	388		0.20	0.00	15.7	14.2 - 17.6	27.6	25.0 - 31.2	48.8	39.5 - 57.6	65.3	57.1 - 76.2	16.5	15.2 - 17.8
3-5	sU	SK	Children cohort	2015 - 2016	F,M	67		0.20	0.00	23.8	21.3 - 29.1	39.5	29.6 - 51.1	61.9	48.7 - 81.2	80.5		22.2	18.1 - 27.3
6-11	mU	DE	GerES IV 2005	2005	F,M	102		0.25	0.00	63.4	53.9 - 70.4	98.7	83.7 - 126	153	128 - 207	201	155 - 332	65.6	57.7 - 74.6
6-11	sU	DK	CPH-MC	2006 - 2007	F,M	659	0.43		0.00	28.3	25.9 - 30.9	48.1	44.4 - 52.8	75.9	70.5 - 86.8	108	91.5 - 130	26.8	25.0 - 28.8
6-11	mU	DK	CPHPUB-Cross	2006 - 2008	F,M	913	0.55		0.00	29.3	27.7 - 31.0	44.9	41.9 - 48.3	68.3	61.9 - 75.0	89.4	80.9 - 103	29.7	28.4 - 31.1
6-11	sU	AT	PBAT	2010 - 2012	F,M	165	0.46	0.92	0.00	15.8	13.7 - 17.7	27.5	23.4 - 33.8	42.8	39.5 - 55.5	56.3	45.0 - 80.1	16.1	14.4 - 18.1
6-11	mU	SE	DEMOCOPHES-SE	2011 - 2012	F,M	98		0.30	0.00	22.6	17.7 - 25.0	31.8	27.3 - 38.4	46.3	39.1 - 79.5	75.0	49.8 - 125	21.3	18.6 - 24.4
6-11	mU	DK	DEMOCOPHES-DK	2011	F,M	143		0.55	0.00	14.6	13.3 - 17.2	25.9	21.5 - 29.5	33.9	31.3 - 52.9	53.4	35.1 - 83.3	15.8	14.0 - 17.8
6-11	mU	LU	DEMOCOPHES-LU	2011 - 2012	F,M	59		0.20	0.00	19.5	17.1 - 23.2	28.6	23.3 - 37.6	41.3	30.2 - 62.2	53.2		18.4	15.4 - 21.9
6-11	mU	DE	DEMOCOPHES-DE	2011	F,M	119		0.20	0.00	26.3	23.6 - 33.5	44.3	36.8 - 51.8	65.3	53.3 - 83.9	80.4	65.9 - 381	28.4	24.7 - 32.5
6-11	mU	PL	DEMOCOPHES-PL	2011 - 2012	F,M	94	0.12	0.37	0.00	23.6	19.6 - 28.6	37.3	31.8 - 49.5	54.1	49.6 - 70.6	63.7	54.6 - 109	22.1	18.9 - 25.7
6-11	mU	CZ	DEMOCOPHES-CZ	2011 - 2012	F,M	118	0.07	0.20	0.00	17.9	15.4 - 21.2	29.7	24.7 - 38.0	44.7	39.7 - 71.6	70.5	46.6 - 169	19.0	16.5 - 21.9
6-11	mU	NO	IES	2012	F,M	50	0.70	2.00	0.00	29.1	23.0 - 35.8	42.7	35.4 - 50.8	58.4	45.1 - 101	78.1		28.5	24.1 - 33.7
6-11	mU	SK	PCBcohort_c	2014 - 2016	F,M	249	0.23		0.00	37.2	32.8 - 41.0	54.4	48.9 - 62.7	77.3	72.4 - 91.5	103	87.6 - 147	35.7	32.8 - 38.9
6-11	mU	DE	GerES V	2015 - 2017	F,M	937		0.20	0.11	13.1	12.5 - 14.1	21.7	20.6 - 23.0	32.8	30.2 - 35.7	45.7	39.0 - 54.3	13.4	12.8 - 14.1
6-11	sU	SK	Children cohort	2015 - 2016	F,M	112		0.20	0.00	27.8	22.5 - 32.1	48.0	42.9 - 60.2	73.1	64.2 - 113	106	78.5 - 171	26.7	22.6 - 31.4
12-19	mU	DE	GerES IV 2005	2005	F,M	51		0.25	0.00	53.1	40.3 - 68.1	80.7	67.5 - 107	114	90.9 - 283	156		52.8	42.2 - 66.0
12-19	mU	DK	CPHPUB-Cross	2006 - 2008	F,M	389	0.55		0.00	22.3	21.1 - 24.6	37.7	35.0 - 42.5	57.0	50.7 - 66.0	72.5	65.9 - 81.3	22.9	21.1 - 24.8
12-19	sU	DK	DYMS 2007	2007	M	239	0.55	0.55	0.00	18.2	16.2 - 19.7	29.1	25.4 - 33.3	45.0	40.7 - 62.0	69.4	50.1 - 96.7	17.5	15.7 - 19.6
12-19	sU	DK	DYMS 2008	2008	M	261	0.55	0.55	0.38	14.9	13.3 - 16.9	25.0	22.4 - 27.5	36.2	32.0 - 47.1	61.2	39.7 - 149	15.1	13.4 - 17.1
12-19	sU	DK	DYMS 2009	2009	M	210	0.07	0.07	0.00	13.4	12.0 - 14.8	19.9	18.4 - 22.2	30.4	26.5 - 34.9	39.3	32.0 - 51.1	11.9	10.6 - 13.3
12-19	sU	AT	PBAT	2010 - 2012	F,M	94	0.46	0.92	0.00	16.5	13.3 - 18.7	27.5	21.1 - 36.1	38.6	36.3 - 43.8	42.7	38.8 - 73.6	15.7	13.6 - 18.2
12-19	sU	DK	DYMS 2013	2013	M	70	0.07	0.07	0.00	9.69	8.13 - 11.3	14.7	11.7 - 16.3	19.3	16.0 - 32.5	24.9		9.54	7.93 - 11.5
12-19	mU	DE	GerES V	2015 - 2017	F,M	932		0.20	0.00	8.90	8.30 - 9.54	15.1	14.2 - 15.9	23.7	21.6 - 25.8	33.0	30.2 - 40.3	9.08	8.63 - 9.55
12-19	mU	SK	PCBcohort_t	2015 - 2017	F,M	65	0.23		0.00	28.7	21.8 - 37.1	42.2	38.6 - 69.4	79.3	64.9 - 149	104		26.6	21.5 - 33.1
12-19	sU	DK	DYMS 2017	2017	M	76	0.07	0.07	0.00	6.85	5.63 - 8.44	10.3	8.92 - 11.4	13.4	11.2 - 24.4	19.0	13.5 - 43.4	6.89	5.96 - 7.96
20-39	24hU	DE	ESB 2007	2007	F,M	60		0.20	0.00	12.7	9.12 - 14.5	19.9	14.5 - 28.6	31.1	27.2 - 165	46.6		13.3	10.6 - 16.6
20-39	sU	DK	DYMS 2008	2008	M	54	0.55	0.55	0.00	16.2	14.0 - 21.9	25.4	22.0 - 41.3	44.9	33.4 - 50.3	48.9		17.1	14.1 - 20.7
20-39	24hU	DE	ESB 2008	2008	F,M	54		0.50	0.00	9.66	7.63 - 11.5	13.5	11.5 - 19.0	21.2	18.3 - 25.1	23.6		9.31	7.91 - 10.9
20-39	sU	DK	DYMS 2009	2009	M	79	0.07	0.07	0.00	15.9	12.4 - 19.2	23.9	20.1 - 27.5	32.4	27.2 - 39.1	36.3	32.5 - 740	15.0	12.5 - 18.1
20-39	24hU	DE	ESB 2009	2009	F,M	60		0.20	0.00	8.85	6.20 - 11.4	14.1	11.5 - 21.2	27.3	19.5 - 40.4	38.8		9.13	7.44 - 11.2
20-39	sU	AT	PBAT	2010 - 2011	F,M	135	0.46	0.92	1.48	8.89	7.41 - 9.89	14.0	11.7 - 17.2	21.3	18.2 - 33.1	33.0	24.6 - 74.9	8.07	6.88 - 9.46
20-39	mU	SE	DEMOCOPHES-SE	2011 - 2012	F	50		0.30	0.00	11.1	7.89 - 13.7	19.2	13.4 - 30.0	33.9	28.1 - 133	72.9		11.7	8.97 - 15.3

Age	Matrix	Country	Data collection	Sampling year	Sex	N	LOD	LOQ	% below limit	P50	P50_95% CI	P75	P75_95% CI	P90	P90_95% CI	P95	P95_95% CI	GM	GM_95% CI
20-39	mU	DK	DEMOCOPHES-DK	2011	F	51		0.55	0.00	10.4	7.71 - 11.5	14.5	11.5 - 20.0	20.6	16.6 - 39.2	33.9		9.23	7.50 - 11.4
20-39	mU	DE	DEMOCOPHES-DE	2011	F	64		0.20	0.00	15.6	12.7 - 19.5	30.0	22.3 - 40.4	41.8	34.1 - 63.1	52.9		16.6	13.9 - 19.9
20-39	24hU	DE	ESB 2011	2011	F,M	60		0.20	0.00	7.30	6.70 - 9.30	11.6	9.46 - 15.9	16.9	14.5 - 24.7	20.1		8.09	6.97 - 9.39
20-39	sU	IL	IBS	2011	F,M	144	0.25	0.50	0.00	34.4	29.7 - 37.7	57.1	47.2 - 71.3	106	80.3 - 141	142	119 - 246	32.7	28.2 - 38.0
20-39	24hU	DE	ESB 2013	2013	F,M	60		0.20	0.00	5.90	4.81 - 7.00	9.32	7.08 - 11.8	12.2	10.3 - 19.3	14.8		5.88	5.03 - 6.86
20-39	24hU	DE	ESB 2015	2015	F,M	60		0.20	0.00	3.80	3.30 - 5.09	6.53	5.10 - 8.54	10.6	7.73 - 20.9	14.0		4.37	3.66 - 5.23
40-59	sU	AT	PBAT	2010 - 2011	F,M	112	0.46	0.92	4.46	7.26	5.74 - 8.21	12.1	10.3 - 13.7	16.3	14.2 - 23.1	21.9	17.6 - 31.5	6.15	5.19 - 7.30
40-59	mU	DK	DEMOCOPHES-DK	2011	F	94		0.55	0.00	8.05	7.17 - 9.93	11.9	10.7 - 14.0	18.6	14.1 - 28.5	24.9	18.8 - 302	8.45	7.14 - 10.0
40-59	mU	DE	DEMOCOPHES-DE	2011	F	56		0.20	0.00	9.83	8.03 - 14.2	19.6	14.4 - 24.7	28.9	24.0 - 52.3	39.0		11.3	9.27 - 13.8
40-59	sU	IL	IBS	2011	F,M	86	0.25	0.50	0.00	33.5	26.1 - 41.6	61.6	46.4 - 78.6	103	74.3 - 140	122	104 - 265	33.0	27.3 - 39.8
6-59	mU	AT	IC-HBM	2009	F	50	0.35	1.30	0.00	14.0	11.0 - 22.3	26.0	21.9 - 41.8	76.9	30.2 - 306	146		18.6	13.9 - 24.8
6-59	mU	AT	IC-HBM	2009	F,M	50	0.35	1.30	0.00	32.5	28.1 - 40.6	51.5	39.9 - 70.8	96.0	66.0 - 112	105		33.0	27.3 - 40.0
60+	sU	AT	PBAT	2010 - 2012	F,M	89	0.46	0.92	1.12	9.70	7.87 - 13.9	20.5	16.8 - 25.8	31.5	25.8 - 63.0	51.9	32.9 - 134	10.1	8.11 - 12.6

Notes. Matrix: mU - first morning urine, sU - spot urine, 24hU - 24-hours urine; country: AT - Austria, BE - Belgium, CZ - Czech Republic, DE - Germany, DK - Denmark, ES - Spain, HU - Hungary, IL - Israel, LU - Luxemburg, NO - Norway, PL - Poland, SE - Sweden, SI - Slovenia, SK - Slovakia; sex: F - female, M - male; n - sample size; LOD - limit of detection; LOQ - limit of quantification; % below limit - percentage of values below limit of detection/quantification, whichever was applied in the data collection; P50, P75, P90, P95 - 50th, 75th, 90th, 95th percentile; 95% CI - 95 % confidence interval; GM - geometric mean; NA - Not available.

Data only displayed if n was at least 50; percentiles missing when below LOD/LOQ; CIs of the observed percentiles missing when they fall outside the observed range; GM was imputed (fixed imputation) based on a log-normal distribution when at least 50 % of values were above LOD/LOQ.

Table S6: DnBP metabolite MnBP (Mono-n-butyl phthalate) in microg/L

Age	Matrix	Country	Data collection	Sampling year	Sex	N	LOD	LOQ	% below limit	P50	P50_95% CI	P75	P75_95% CI	P90	P90_95% CI	P95	P95_95% CI	GM	GM_95% CI
3-5	mU	DE	GerES IV 2005	2005	F,M	50		0.10	0.00	113	88.8 - 165	197	163 - 264	280	222 - 360	326		119	99.6 - 143
3-5	mU	DE	GerES V	2015 - 2017	F,M	388		1.00	0.26	25.4	23.1 - 27.4	39.8	37.5 - 41.5	64.2	54.4 - 70.9	85.9	70.5 - 118	25.1	23.3 - 27.1
3-5	sU	SK	Children cohort	2015 - 2016	F,M	67		1.00	0.00	49.0	36.5 - 66.8	95.6	68.3 - 155	171	132 - 258	209		50.0	40.1 - 62.3
3-5	mU	CZ	CzechHBM-CE	2016 - 2017	F,M	148		1.60	0.00	61.6	58.2 - 68.0	105	89.5 - 137	192	148 - 231	233	207 - 337	65.5	57.8 - 74.1
6-11	mU	DE	GerES IV 2005	2005	F,M	102		0.10	0.00	104	93.8 - 134	177	140 - 200	237	202 - 318	314	242 - 450	108	94.7 - 122
6-11	mU	DK	CPHPUB-Cross	2006 - 2008	F,M	913	1.43		0.00	52.0	49.1 - 54.5	81.5	77.5 - 86.0	113	108 - 130	156	144 - 173	52.3	50.1 - 54.6
6-11	sU	AT	PBAT	2010 - 2012	F,M	165	0.53	1.06	0.61	12.8	11.0 - 15.2	25.0	21.0 - 29.5	32.9	30.7 - 43.9	44.0	36.8 - 50.6	13.0	11.6 - 14.7
6-11	mU	DK	DEMOCOPHES-DK	2011	F,M	143		1.43	0.00	31.8	26.7 - 36.1	49.8	43.2 - 60.0	78.4	64.3 - 97.9	98.3	85.5 - 107	30.8	27.5 - 34.5
6-11	mU	ES	DEMOCOPHES-ES	2011 - 2012	F,M	120		1.00	0.00	47.4	41.6 - 56.1	74.2	62.6 - 91.2	116	96.1 - 167	163	126 - 197	46.1	40.4 - 52.7
6-11	mU	SL	DEMOCOPHES-SI	2011 - 2012	F,M	119		0.50	0.00	38.0	31.4 - 48.2	67.5	54.3 - 78.0	106	80.8 - 141	139	109 - 192	40.0	35.0 - 45.7
6-11	mU	LU	DEMOCOPHES-LU	2011 - 2012	F,M	60		0.50	0.00	31.0	21.0 - 35.0	42.2	35.8 - 49.7	54.7	45.6 - 80.6	67.7		25.7	21.6 - 30.7
6-11	mU	DE	DEMOCOPHES-DE	2011	F,M	119		1.00	0.00	45.9	38.0 - 54.1	72.5	59.1 - 88.6	149	92.7 - 178	173	156 - 267	45.9	39.9 - 52.9
6-11	mU	BE	DEMOCOPHES-BE	2011 - 2012	F,M	129		0.50	0.00	39.0	33.0 - 43.0	57.0	51.4 - 68.0	96.4	75.1 - 121	121	99.6 - 212	37.0	32.5 - 42.1
6-11	mU	PL	DEMOCOPHES-PL	2011 - 2012	F,M	120		0.50	0.00	82.5	74.0 - 90.1	122	101 - 160	260	181 - 271	271	262 - 396	83.2	72.7 - 95.1
6-11	mU	CZ	DEMOCOPHES-CZ	2011 - 2012	F,M	118	0.18	0.40	2.54	82.0	67.2 - 91.6	131	118 - 159	206	172 - 286	259	220 - 569	81.0	70.1 - 93.5
6-11	mU	NO	IES	2012	F,M	50	0.20	0.50	0.00	38.8	31.2 - 46.9	60.8	46.3 - 82.9	88.7	66.6 - 133	119		40.8	34.5 - 48.3
6-11	mU	SK	PCBcohort_c	2014 - 2016	F,M	249	0.67		0.00	74.5	66.2 - 83.4	127	110 - 139	222	175 - 253	287	241 - 351	74.7	67.7 - 82.4
6-11	mU	DE	GerES V	2015 - 2017	F,M	937		1.00	0.11	22.7	21.9 - 23.7	37.1	34.4 - 39.0	55.9	53.4 - 58.9	69.5	64.7 - 79.5	22.6	21.6 - 23.7
6-11	sU	SK	Children cohort	2015 - 2016	F,M	112		1.00	0.00	51.8	40.5 - 69.4	86.0	74.7 - 105	148	108 - 203	203	155 - 236	47.4	40.0 - 56.1
6-11	mU	CZ	CzechHBM-CE	2016 - 2017	F,M	223		1.60	0.00	63.2	53.3 - 68.4	103	88.0 - 120	172	135 - 210	230	191 - 275	61.4	55.6 - 67.8
12-19	mU	DE	GerES IV 2005	2005	F,M	51		0.10	0.00	81.4	61.6 - 95.5	131	95.4 - 165	187	136 - 285	242		81.4	67.0 - 98.9
12-19	mU	DK	CPHPUB-Cross	2006 - 2008	F,M	389	1.43		0.00	40.0	37.4 - 43.8	60.1	55.5 - 64.4	86.3	82.4 - 100	113	98.3 - 133	38.7	36.2 - 41.5
12-19	sU	DK	DYMS 2007	2007	M	239	1.10	1.10	1.26	33.7	29.2 - 38.6	52.6	47.4 - 58.3	82.2	67.3 - 93.0	99.4	86.4 - 118	29.6	26.3 - 33.2
12-19	sU	DK	DYMS 2008	2008	M	261	1.10	1.10	0.38	26.8	23.1 - 30.5	40.3	37.6 - 47.4	60.9	55.5 - 71.6	76.8	66.1 - 90.2	23.9	21.6 - 26.4
12-19	mU	BE	FLEHS2RefAdo	2008 - 2009	F,M	210	10.0		2.38	38.5	36.0 - 45.6	61.8	55.5 - 67.0	87.3	74.6 - 109	117	94.8 - 251	39.7	35.8 - 43.9
12-19	sU	DK	DYMS 2009	2009	M	210	0.17	0.17	0.00	28.4	23.8 - 33.9	51.4	44.0 - 58.4	78.1	65.8 - 97.5	102	86.9 - 130	25.7	22.5 - 29.3
12-19	sU	AT	PBAT	2010 - 2012	F,M	94	0.53	1.06	1.06	14.4	11.7 - 16.6	26.9	19.4 - 29.6	39.2	29.6 - 61.0	57.2	39.7 - 82.5	14.4	12.2 - 17.0
12-19	sU	DK	DYMS 2013	2013	M	70	0.17	0.17	0.00	29.5	21.6 - 31.9	37.4	32.6 - 49.1	59.4	45.4 - 86.9	69.6		21.4	17.1 - 26.7
12-19	sU	BE	FLEHS3RefAdo	2013	F,M	207		0.50	0.00	24.3	21.3 - 27.2	37.1	31.5 - 43.2	61.0	50.0 - 69.6	77.6	64.6 - 108	22.8	20.6 - 25.4
12-19	mU	DE	GerES V	2015 - 2017	F,M	932		1.00	0.00	19.8	18.7 - 20.7	33.0	31.1 - 34.7	49.5	45.1 - 56.6	67.3	60.7 - 74.8	19.8	18.8 - 20.7
12-19	mU	SK	PCBcohort_t	2015 - 2017	F,M	65	0.67		0.00	83.1	62.9 - 106	138	115 - 178	200	167 - 360	232		82.2	68.0 - 99.3
12-19	sU	DK	DYMS 2017	2017	M	76	0.17	0.17	0.00	19.9	16.5 - 23.1	35.3	26.2 - 44.1	62.0	41.2 - 87.4	80.9	62.4 - 109	19.2	15.9 - 23.2
20-39	24hU	DE	ESB 2007	2007	F,M	60		1.00	0.00	16.4	11.0 - 20.9	23.9	21.0 - 29.6	34.0	27.2 - 47.8	37.3		15.0	12.5 - 17.9
20-39	sU	DK	DYMS 2008	2008	M	54	1.10	1.10	0.00	27.2	20.1 - 31.8	40.2	31.8 - 55.5	58.8	44.9 - 72.2	62.4		23.9	19.7 - 28.9

Age	Matrix	Country	Data collection	Sampling year	Sex	N	LOD	LOQ	% below limit	P50	P50_95% CI	P75	P75_95% CI	P90	P90_95% CI	P95	P95_95% CI	GM	GM_95% CI
20-39	mU	BE	FLEHS2RefAdult	2008 - 2009	F,M	189	10.0		10.6	30.0	26.0 - 36.4	54.0	47.0 - 68.6	103	86.0 - 122	134	105 - 192	31.1	27.4 - 35.2
20-39	24hU	DE	ESB 2008	2008	F,M	54		2.00	0.00	19.4	16.9 - 23.7	26.1	23.7 - 32.4	37.4	27.7 - 69.2	63.8		18.8	15.9 - 22.1
20-39	sU	DK	DYMS 2009	2009	M	79	0.17	0.17	0.00	26.9	22.3 - 34.8	45.7	38.2 - 60.5	72.4	55.5 - 126	96.0	74.8 - 216	27.0	22.4 - 32.6
20-39	24hU	DE	ESB 2009	2009	F,M	60		1.00	0.00	16.3	11.9 - 20.3	23.9	20.9 - 37.0	42.5	31.4 - 54.1	48.9		15.3	12.5 - 18.6
20-39	sU	AT	PBAT	2010 - 2011	F,M	135	0.53	1.06	0.74	8.31	7.33 - 10.3	15.0	12.6 - 18.5	27.6	21.7 - 43.7	43.5	29.9 - 54.8	8.92	7.72 - 10.3
20-39	mU	DK	DEMOCOPHES-DK	2011	F	51		1.43	0.00	24.3	17.8 - 30.9	38.7	30.7 - 57.6	66.2	48.5 - 105	73.0		23.2	18.5 - 29.0
20-39	mU	SL	DEMOCOPHES-SI	2011 - 2012	F	63		0.50	0.00	23.0	18.0 - 29.7	40.0	30.9 - 56.8	63.0	53.0 - 258	112		25.9	20.1 - 33.6
20-39	mU	DE	DEMOCOPHES-DE	2011	F	64		1.00	0.00	31.0	25.8 - 38.9	55.4	41.5 - 66.7	77.0	64.1 - 111	85.4		31.1	25.8 - 37.4
20-39	mU	BE	DEMOCOPHES-BE	2011 - 2012	F	62		0.50	0.00	26.5	22.9 - 31.0	38.2	31.2 - 62.6	73.3	53.4 - 124	119		26.7	21.6 - 33.0
20-39	24hU	DE	ESB 2011	2011	F,M	60		1.00	0.00	14.8	11.1 - 17.2	22.4	18.4 - 25.3	25.6	24.4 - 28.2	27.3		14.0	12.3 - 16.0
20-39	mU	PL	DEMOCOPHES-PL	2011 - 2012	F	88		0.50	0.00	43.5	38.4 - 54.6	76.0	60.8 - 93.7	126	92.0 - 174	169	138 - 565	45.1	37.7 - 53.8
20-39	sU	IL	IBS	2011	F,M	144	1.00	2.00	0.00	27.1	23.5 - 31.8	50.5	44.0 - 69.9	91.1	75.0 - 99.9	100	92.0 - 170	28.5	24.5 - 33.0
20-39	24hU	DE	ESB 2013	2013	F,M	60		1.00	0.00	11.2	8.64 - 13.2	15.8	13.7 - 22.5	24.3	18.7 - 36.9	29.9		11.4	9.79 - 13.4
20-39	24hU	DE	ESB 2015	2015	F,M	60		1.00	0.00	8.00	6.90 - 8.89	10.7	9.21 - 16.3	20.3	12.9 - 33.5	21.3		8.21	6.99 - 9.66
20-39	sU	CZ	CzechHBM-AE	2018 - 2019	F,M	194		3.04	0.00	20.3	18.6 - 22.4	34.3	30.2 - 46.6	65.2	52.5 - 81.6	86.5	77.0 - 112	22.8	20.6 - 25.3
40-59	sU	AT	PBAT	2010 - 2011	F,M	112	0.53	1.06	4.46	6.43	5.60 - 8.58	12.3	10.00 - 16.1	20.1	17.6 - 33.2	31.0	20.2 - 54.3	6.58	5.51 - 7.86
40-59	mU	DK	DEMOCOPHES-DK	2011	F	94		1.43	0.00	18.7	15.8 - 22.6	30.3	25.3 - 33.3	38.9	33.4 - 54.4	50.1	40.1 - 102	18.7	16.3 - 21.4
40-59	mU	ES	DEMOCOPHES-ES	2011 - 2012	F	75		1.00	0.00	31.4	25.5 - 39.7	60.5	43.2 - 71.3	91.2	68.5 - 187	127	91.5 - 247	31.7	25.9 - 38.8
40-59	mU	SL	DEMOCOPHES-SI	2011 - 2012	F	57		0.50	0.00	29.0	22.0 - 32.0	48.0	32.0 - 61.5	78.0	54.2 - 177	102		28.3	23.1 - 34.7
40-59	mU	DE	DEMOCOPHES-DE	2011	F	56		1.00	0.00	24.7	21.3 - 37.2	49.1	38.4 - 79.1	98.0	60.4 - 169	128		29.6	23.9 - 36.7
40-59	mU	BE	DEMOCOPHES-BE	2011 - 2012	F	67		0.50	0.00	35.0	30.1 - 39.9	47.0	41.7 - 58.0	83.6	56.0 - 154	108		33.8	28.5 - 40.1
40-59	sU	IL	IBS	2011	F,M	86	1.00	2.00	0.00	27.1	22.5 - 35.2	50.5	40.1 - 74.3	85.7	74.1 - 101	99.4	86.2 - 773	28.5	23.2 - 35.0
40-59	sU	CZ	CzechHBM-AE	2018 - 2019	F,M	192		3.04	0.00	22.5	18.9 - 26.8	38.5	33.7 - 49.3	63.0	54.5 - 79.0	80.4	68.4 - 116	23.8	21.4 - 26.4
60+	sU	AT	PBAT	2010 - 2012	F,M	89	0.53	1.06	2.25	10.2	8.39 - 12.2	16.5	13.4 - 25.7	42.3	25.4 - 58.3	55.3	43.2 - 81.7	10.7	8.78 - 13.1

Notes. Matrix: mU - first morning urine, sU - spot urine, 24hU - 24-hours urine; country: AT - Austria, BE - Belgium, CZ - Czech Republic, DE - Germany, DK - Denmark, ES - Spain, HU - Hungary, IL - Israel, LU - Luxemburg, NO - Norway, PL - Poland, SE - Sweden, SI - Slovenia, SK - Slovakia; sex: F - female, M - male; n - sample size; LOD - limit of detection; LOQ - limit of quantification; % below limit - percentage of values below limit of detection/quantification, whichever was applied in the data collection; P50, P75, P90, P95 - 50th, 75th, 90th, 95th percentile; 95% CI - 95 % confidence interval; GM - geometric mean; NA - Not available.

Data only displayed if n was at least 50; percentiles missing when below LOD/LOQ; CIs of the observed percentiles missing when they fall outside the observed range; GM was imputed (fixed imputation) based on a log-normal distribution when at least 50 % of values were above LOD/LOQ.

Table S7: DnBP metabolite OH-MnBP (3-OH-Mono-n-butyl phthalate) in microg/L

Age	Matrix	Country	Data collection	Sampling year	Sex	N	LOD	LOQ	% below limit	P50	P50_95% CI	P75	P75_95% CI	P90	P90_95% CI	P95	P95_95% CI	GM	GM_95% CI
3-5	mU	DE	GerES V	2015 - 2017	F,M	388		0.25	0.00	3.30	3.10 - 3.70	5.50	5.06 - 5.90	8.90	7.55 - 10.5	11.6	10.1 - 13.4	3.30	3.05 - 3.56
3-5	sU	SK	Children cohort	2015 - 2016	F,M	67		0.25	0.00	8.31	5.36 - 9.20	13.2	9.34 - 21.1	21.5	20.2 - 38.5	34.2		7.35	5.90 - 9.16
6-11	mU	DE	DEMOCOPHES-DE	2011	F,M	119		0.25	0.84	3.30	2.90 - 4.06	5.85	4.90 - 9.77	12.9	10.1 - 19.6	19.3	13.1 - 34.4	3.51	2.95 - 4.19
6-11	mU	DE	GerES V	2015 - 2017	F,M	937		0.25	0.53	2.70	2.60 - 2.80	4.50	4.20 - 4.80	6.70	6.30 - 7.34	9.20	8.11 - 9.90	2.67	2.54 - 2.81
6-11	sU	SK	Children cohort	2015 - 2016	F,M	112		0.25	0.00	6.54	4.96 - 7.60	11.3	9.37 - 15.1	21.0	16.6 - 25.8	25.6	22.2 - 38.1	5.92	4.95 - 7.08
12-19	sU	DK	DYMS 2009	2009	M	69	0.25	0.25	13.0	2.88	2.09 - 3.65	5.16	4.05 - 6.75	8.12	6.29 - 12.3	9.52		2.19	1.65 - 2.91
12-19	sU	DK	DYMS 2013	2013	M	70	0.25	0.25	17.1	1.82	1.37 - 2.30	3.19	2.42 - 4.20	4.97	3.61 - 5.96	5.42		1.39	1.08 - 1.81
12-19	mU	DE	GerES V	2015 - 2017	F,M	932		0.25	0.75	2.00	1.90 - 2.20	3.50	3.20 - 3.70	5.59	5.00 - 6.18	7.64	6.84 - 8.35	2.04	1.94 - 2.15
12-19	sU	DK	DYMS 2017	2017	M	76	0.25	0.25	17.1	1.37	1.02 - 1.90	2.37	2.06 - 3.37	4.77	2.90 - 7.26	6.67	4.91 - 10.6	1.13	0.87 - 1.47
20-39	24hU	DE	ESB 2007	2007	F,M	60		0.25	1.67	1.40	1.20 - 1.80	2.33	1.80 - 3.37	3.52	3.22 - 5.02	4.12		1.46	1.22 - 1.74
20-39	24hU	DE	ESB 2009	2009	F,M	60		0.25	0.00	1.40	1.10 - 1.80	2.42	1.80 - 3.14	3.41	2.96 - 5.45	3.67		1.34	1.10 - 1.63
20-39	mU	DE	DEMOCOPHES-DE	2011	F	64		0.25	3.12	1.85	1.58 - 2.74	3.67	2.90 - 4.89	5.80	4.71 - 12.4	9.54		2.03	1.62 - 2.54
20-39	24hU	DE	ESB 2011	2011	F,M	60		0.25	0.00	1.45	1.10 - 1.80	2.10	1.80 - 2.97	3.20	2.70 - 5.07	4.04		1.42	1.21 - 1.67
20-39	24hU	DE	ESB 2013	2013	F,M	60		0.25	0.00	1.10	0.80 - 1.40	1.65	1.40 - 2.20	2.41	2.06 - 4.57	3.04		1.08	0.91 - 1.28
20-39	24hU	DE	ESB 2015	2015	F,M	60		0.25	8.33	0.80	0.70 - 0.90	1.20	0.90 - 1.60	1.62	1.46 - 3.40	2.92		0.79	0.66 - 0.95
40-59	mU	DE	DEMOCOPHES-DE	2011	F	56		0.25	3.57	1.85	1.26 - 2.27	2.90	2.30 - 4.29	5.25	3.51 - 11.8	10.1		1.83	1.44 - 2.32

Notes. Matrix: mU - first morning urine, sU - spot urine, 24hU - 24-hours urine; country: AT - Austria, BE - Belgium, CZ - Czech Republic, DE - Germany, DK - Denmark, ES - Spain, HU - Hungary, IL - Israel, LU - Luxemburg, NO - Norway, PL - Poland, SE - Sweden, SI - Slovenia, SK - Slovakia; sex: F - female, M - male; n - sample size; LOD - limit of detection; LOQ - limit of quantification; % below limit - percentage of values below limit of detection/quantification, whichever was applied in the data collection; P50, P75, P90, P95 - 50th, 75th, 90th, 95th percentile; 95% CI - 95 % confidence interval; GM - geometric mean; NA - Not available.

Data only displayed if n was at least 50; percentiles missing when below LOD/LOQ; CIs of the observed percentiles missing when they fall outside the observed range; GM was imputed (fixed imputation) based on a log-normal distribution when at least 50 % of values were above LOD/LOQ.

Table S8: DiBP metabolite MiBP (Mono-isobutyl phthalate) in microg/L

Age	Matrix	Country	Data collection	Sampling year	Sex	N	LOD	LOQ	% below limit	P50	P50_95% CI	P75	P75_95% CI	P90	P90_95% CI	P95	P95_95% CI	GM	GM_95% CI
3-5	mU	DE	GerES IV 2005	2005	F,M	50		0.10	0.00	97.2	76.3 - 120	146	118 - 255	277	175 - 511	322		103	84.1 - 127
3-5	mU	DE	GerES V	2015 - 2017	F,M	388		1.00	0.00	29.7	27.7 - 31.6	53.7	48.8 - 59.4	85.1	74.7 - 101	114	100 - 151	30.2	27.8 - 32.9
3-5	sU	SK	Children cohort	2015 - 2016	F,M	67		1.00	0.00	40.3	29.2 - 54.4	76.3	61.0 - 97.6	109	93.7 - 130	121		37.2	29.7 - 46.5
3-5	mU	CZ	CzechHBM-CE	2016 - 2017	F,M	148		2.70	0.00	41.1	36.7 - 48.3	72.4	60.4 - 97.0	165	112 - 238	238	174 - 395	45.0	38.8 - 52.2
6-11	mU	DE	GerES IV 2005	2005	F,M	102		0.10	0.00	93.0	81.9 - 113	154	136 - 180	214	181 - 301	284	221 - 436	97.1	85.5 - 110
6-11	mU	DK	CPHPUB-Cross	2006 - 2008	F,M	913	1.10		0.00	79.3	76.4 - 83.0	126	118 - 137	180	171 - 200	241	220 - 276	81.4	77.9 - 85.1
6-11	sU	AT	PBAT	2010 - 2012	F,M	165	0.59	1.18	1.21	35.2	28.9 - 39.1	57.3	51.7 - 67.9	91.3	78.1 - 122	125	104 - 145	32.4	28.4 - 37.1
6-11	mU	DK	DEMOCOPHES-DK	2011	F,M	143		1.10	0.00	53.8	48.2 - 63.7	89.8	79.5 - 106	140	112 - 192	192	152 - 217	56.7	50.4 - 63.7
6-11	mU	ES	DEMOCOPHES-ES	2011 - 2012	F,M	120		1.00	0.00	55.6	47.8 - 64.9	91.0	77.7 - 107	133	114 - 180	178	140 - 229	55.7	49.1 - 63.2
6-11	mU	SL	DEMOCOPHES-SI	2011 - 2012	F,M	119		1.00	0.00	55.0	50.0 - 66.6	93.5	78.6 - 125	149	128 - 227	225	164 - 237	57.8	50.6 - 66.0
6-11	mU	LU	DEMOCOPHES-LU	2011 - 2012	F,M	60		0.50	0.00	34.5	27.0 - 45.0	54.8	45.0 - 65.7	79.7	59.6 - 232	115		32.6	25.7 - 41.4
6-11	mU	DE	DEMOCOPHES-DE	2011	F,M	119		1.00	0.00	37.7	33.8 - 46.0	64.1	55.3 - 79.5	109	96.1 - 164	154	116 - 238	40.9	35.6 - 47.0
6-11	mU	BE	DEMOCOPHES-BE	2011 - 2012	F,M	129		0.50	0.00	48.0	41.0 - 63.1	96.0	81.4 - 116	178	125 - 359	356	196 - 777	55.7	47.1 - 65.8
6-11	mU	PL	DEMOCOPHES-PL	2011 - 2012	F,M	120		0.50	0.00	104	89.0 - 111	180	131 - 206	261	212 - 302	273	265 - 572	101	88.0 - 116
6-11	mU	CZ	DEMOCOPHES-CZ	2011 - 2012	F,M	118	0.07	0.20	0.00	52.0	41.1 - 65.2	84.3	71.8 - 105	135	110 - 184	182	147 - 250	47.2	39.5 - 56.4
6-11	mU	NO	IES	2012	F,M	50	0.20	0.50	0.00	48.5	33.3 - 56.8	75.5	54.4 - 128	143	91.0 - 227	160		48.3	39.5 - 59.0
6-11	mU	SK	PCBcohort_c	2014 - 2016	F,M	249	0.67		0.40	61.5	51.7 - 70.8	108	94.3 - 129	176	143 - 215	246	202 - 487	59.8	53.1 - 67.3
6-11	mU	DE	GerES V	2015 - 2017	F,M	937		1.00	0.11	27.6	26.3 - 28.8	43.1	40.3 - 46.6	75.2	68.2 - 86.4	113	101 - 140	27.8	26.4 - 29.3
6-11	sU	SK	Children cohort	2015 - 2016	F,M	112		1.00	0.00	45.9	40.6 - 63.4	104	74.5 - 146	172	153 - 329	322	212 - 460	52.9	43.6 - 64.2
6-11	mU	CZ	CzechHBM-CE	2016 - 2017	F,M	223		2.70	0.00	37.4	34.4 - 45.7	72.5	57.6 - 85.7	138	104 - 180	214	148 - 407	43.4	38.8 - 48.6
12-19	mU	DE	GerES IV 2005	2005	F,M	51		0.10	0.00	81.5	55.9 - 99.8	127	99.5 - 200	327	159 - 1338	457		90.4	70.3 - 116
12-19	mU	DK	CPHPUB-Cross	2006 - 2008	F,M	389	1.10		0.00	74.0	67.0 - 79.6	111	102 - 128	154	148 - 177	201	177 - 241	70.1	65.5 - 75.1
12-19	sU	DK	DYMS 2007	2007	M	239	1.43	1.43	0.00	48.3	43.7 - 53.6	79.1	70.5 - 90.4	125	106 - 145	167	135 - 229	47.3	42.2 - 53.1
12-19	sU	DK	DYMS 2008	2008	M	261	1.43	1.43	0.00	63.0	55.0 - 67.3	90.8	84.0 - 99.8	140	116 - 165	172	155 - 202	55.5	50.3 - 61.1
12-19	sU	DK	DYMS 2009	2009	M	210	0.16	0.16	0.00	47.4	39.1 - 54.8	73.2	64.2 - 89.8	121	104 - 156	174	139 - 344	43.8	38.8 - 49.6
12-19	sU	AT	PBAT	2010 - 2012	F,M	94	0.59	1.18	0.00	39.6	33.7 - 48.1	71.7	55.9 - 104	125	105 - 148	137	127 - 203	41.1	34.9 - 48.3
12-19	sU	DK	DYMS 2013	2013	M	70	0.16	0.16	0.00	29.5	21.0 - 35.5	43.0	36.1 - 54.1	59.2	52.5 - 104	87.2		24.7	20.2 - 30.2
12-19	sU	BE	FLEHS3RefAdo	2013	F,M	207		0.50	0.00	24.8	21.3 - 27.8	40.8	36.7 - 49.5	66.6	56.9 - 93.1	99.5	81.7 - 170	24.3	21.6 - 27.4
12-19	mU	DE	GerES V	2015 - 2017	F,M	932		1.00	0.00	22.0	20.4 - 23.5	39.0	36.2 - 42.7	67.2	60.2 - 80.6	104	89.9 - 117	23.6	22.4 - 25.0
12-19	mU	SK	PCBcohort_t	2015 - 2017	F,M	65	0.67		0.00	56.9	41.2 - 74.2	124	80.8 - 161	207	135 - 323	258		59.2	46.8 - 74.8
12-19	sU	DK	DYMS 2017	2017	M	76	0.16	0.16	0.00	20.9	16.4 - 23.7	32.9	27.3 - 41.0	45.3	40.4 - 75.8	61.5	45.3 - 129	19.8	16.6 - 23.5
20-39	24hU	DE	ESB 2007	2007	F,M	60		1.00	0.00	19.3	16.3 - 25.3	34.5	25.7 - 44.2	47.5	41.4 - 97.2	79.3		21.8	18.3 - 25.9
20-39	sU	DK	DYMS 2008	2008	M	54	1.43	1.43	0.00	64.0	55.2 - 91.8	106	91.8 - 135	147	122 - 181	158		62.6	52.1 - 75.3
20-39	24hU	DE	ESB 2008	2008	F,M	54		2.00	0.00	24.5	19.7 - 31.5	36.7	31.6 - 49.8	58.2	40.0 - 194	98.1		26.8	22.2 - 32.2

Age	Matrix	Country	Data collection	Sampling year	Sex	N	LOD	LOQ	% below limit	P50	P50_95% CI	P75	P75_95% CI	P90	P90_95% CI	P95	P95_95% CI	GM	GM_95% CI
20-39	sU	DK	DYMS 2009	2009	M	79	0.16	0.16	0.00	55.6	45.3 - 68.3	96.9	74.2 - 110	151	108 - 281	202	151 - 522	53.3	43.8 - 64.9
20-39	24hU	DE	ESB 2009	2009	F,M	60		1.00	0.00	19.6	15.8 - 24.4	28.1	24.6 - 38.7	58.1	36.9 - 105	79.4		20.4	17.0 - 24.5
20-39	sU	AT	PBAT	2010 - 2011	F,M	135	0.59	1.18	2.22	24.7	21.5 - 27.7	42.1	33.4 - 57.5	79.2	66.9 - 108	108	81.7 - 118	23.4	19.7 - 27.7
20-39	mU	DK	DEMOCOPHES-DK	2011	F	51		1.10	0.00	41.0	33.0 - 56.5	77.8	55.8 - 103	114	84.2 - 190	151		43.7	35.0 - 54.6
20-39	mU	SL	DEMOCOPHES-SI	2011 - 2012	F	63		1.00	0.00	39.0	35.0 - 50.0	62.0	51.7 - 80.1	86.6	69.4 - 142	112		38.9	32.4 - 46.6
20-39	mU	DE	DEMOCOPHES-DE	2011	F	64		1.00	0.00	29.0	22.2 - 36.9	41.6	37.3 - 45.3	53.6	44.1 - 97.5	86.4		27.3	23.2 - 32.1
20-39	mU	BE	DEMOCOPHES-BE	2011 - 2012	F	62		0.50	0.00	32.5	27.0 - 47.2	61.0	49.5 - 87.8	112	71.1 - 209	126		35.4	28.3 - 44.4
20-39	24hU	DE	ESB 2011	2011	F,M	60		1.00	0.00	20.2	16.9 - 23.9	30.9	25.3 - 37.5	40.3	32.1 - 54.5	50.8		19.6	16.8 - 22.9
20-39	mU	PL	DEMOCOPHES-PL	2011 - 2012	F	88		0.50	0.00	50.0	44.4 - 58.8	84.2	68.8 - 106	147	105 - 234	190	153 - 538	51.4	43.0 - 61.4
20-39	sU	IL	IBS	2011	F,M	144	1.00	2.00	0.00	38.1	34.0 - 46.2	67.7	61.2 - 78.7	89.8	82.2 - 97.8	97.9	92.9 - 211	36.3	31.7 - 41.6
20-39	24hU	DE	ESB 2013	2013	F,M	60		1.00	0.00	16.4	13.3 - 17.9	24.2	19.0 - 33.0	43.5	27.2 - 52.7	52.5		16.3	13.9 - 19.1
20-39	24hU	DE	ESB 2015	2015	F,M	60		1.00	0.00	9.80	8.61 - 11.6	13.3	11.7 - 19.2	23.7	17.2 - 71.7	33.4		10.9	8.82 - 13.5
20-39	sU	CZ	CzechHBM-AE	2018 - 2019	F,M	194		2.17	0.00	11.8	10.8 - 13.7	21.6	17.4 - 25.8	41.2	30.2 - 51.3	52.9	42.9 - 76.4	13.4	12.1 - 15.0
40-59	sU	AT	PBAT	2010 - 2011	F,M	112	0.59	1.18	6.25	22.9	19.8 - 27.2	34.9	29.6 - 41.4	50.5	43.5 - 101	94.4	53.1 - 130	18.2	14.5 - 22.7
40-59	mU	DK	DEMOCOPHES-DK	2011	F	94		1.10	0.00	35.9	30.1 - 42.0	49.4	45.0 - 61.7	73.7	61.9 - 98.7	85.9	74.5 - 166	33.8	29.6 - 38.5
40-59	mU	ES	DEMOCOPHES-ES	2011 - 2012	F	75		1.00	0.00	39.5	28.0 - 49.5	61.5	50.8 - 70.3	86.8	67.8 - 135	106	86.9 - 199	35.5	29.9 - 42.2
40-59	mU	SL	DEMOCOPHES-SI	2011 - 2012	F	57		1.00	0.00	39.0	27.7 - 48.5	63.0	49.4 - 74.3	84.0	68.1 - 156	144		37.8	31.4 - 45.6
40-59	mU	DE	DEMOCOPHES-DE	2011	F	56		1.00	0.00	17.5	14.8 - 26.8	38.0	28.3 - 51.0	57.4	44.9 - 92.8	89.3		21.2	17.4 - 25.9
40-59	mU	BE	DEMOCOPHES-BE	2011 - 2012	F	67		0.50	0.00	31.0	27.1 - 46.9	64.0	50.7 - 102	116	96.3 - 260	176		38.6	30.9 - 48.3
40-59	sU	IL	IBS	2011	F,M	86	1.00	2.00	1.16	37.2	29.4 - 44.9	60.5	49.4 - 75.6	81.9	75.5 - 116	94.5	82.7 - 308	31.7	25.9 - 39.0
40-59	sU	CZ	CzechHBM-AE	2018 - 2019	F,M	192		2.17	0.00	11.9	10.5 - 13.9	20.7	16.8 - 24.7	31.5	26.6 - 49.9	55.7	36.9 - 117	13.1	11.7 - 14.5
60+	sU	AT	PBAT	2010 - 2012	F,M	89	0.59	1.18	1.12	24.8	22.0 - 30.0	45.1	35.1 - 63.2	87.5	63.2 - 119	113	87.9 - 208	26.2	21.5 - 31.8

Notes. Matrix: mU - first morning urine, sU - spot urine, 24hU - 24-hours urine; country: AT - Austria, BE - Belgium, CZ - Czech Republic, DE - Germany, DK - Denmark, ES - Spain, HU - Hungary, IL - Israel, LU - Luxemburg, NO - Norway, PL - Poland, SE - Sweden, SI - Slovenia, SK - Slovakia; sex: F - female, M - male; n - sample size; LOD - limit of detection; LOQ - limit of quantification; % below limit - percentage of values below limit of detection/quantification, whichever was applied in the data collection; P50, P75, P90, P95 - 50th, 75th, 90th, 95th percentile; 95% CI - 95 % confidence interval; GM - geometric mean; NA - Not available.

Data only displayed if n was at least 50; percentiles missing when below LOD/LOQ; CIs of the observed percentiles missing when they fall outside the observed range; GM was imputed (fixed imputation) based on a log-normal distribution when at least 50 % of values were above LOD/LOQ.

Table S9: DiBP metabolite OH-MiBP (2-OH-Mono-iso- butylphthalate) in microg/L

Age	Matrix	Country	Data collection	Sampling year	Sex	N	LOD	LOQ	% below limit	P50	P50_95% CI	P75	P75_95% CI	P90	P90_95% CI	P95	P95_95% CI	GM	GM_95% CI
3-5	mU	DE	GerES V	2015 - 2017	F,M	388		0.25	0.00	11.1	9.92 - 12.3	19.4	18.0 - 21.5	32.9	28.8 - 36.9	42.4	36.8 - 60.4	11.3	10.4 - 12.3
3-5	sU	SK	Children cohort	2015 - 2016	F,M	67		0.25	0.00	14.8	9.41 - 19.1	26.6	21.3 - 33.0	37.1	31.6 - 52.4	47.3		12.6	9.98 - 15.9
6-11	mU	DE	DEMOCOPHES-DE	2011	F,M	119		0.25	0.00	16.1	13.5 - 19.7	25.8	22.2 - 34.1	45.2	37.4 - 75.9	75.9	57.6 - 86.3	16.2	14.0 - 18.8
6-11	mU	DE	GerES V	2015 - 2017	F,M	937		0.25	0.11	9.50	9.00 - 10.0	15.3	14.5 - 16.4	26.9	23.7 - 30.1	36.8	32.6 - 46.7	9.68	9.19 - 10.2
6-11	sU	SK	Children cohort	2015 - 2016	F,M	112		0.25	0.00	16.8	13.5 - 20.2	35.8	24.4 - 40.7	56.1	45.4 - 81.7	80.3	63.4 - 136	16.8	14.0 - 20.2
12-19	sU	DK	DYMS 2009	2009	M	69	0.25	0.25	0.00	7.21	6.34 - 8.99	12.2	9.95 - 16.2	17.6	15.6 - 30.9	22.8		6.84	5.51 - 8.49
12-19	sU	DK	DYMS 2013	2013	M	70	0.25	0.25	2.86	4.97	3.88 - 6.46	8.21	6.66 - 11.5	13.5	10.1 - 18.6	16.9		4.41	3.51 - 5.55
12-19	mU	DE	GerES V	2015 - 2017	F,M	932		0.25	0.00	7.25	6.90 - 7.80	12.9	12.0 - 13.8	23.6	21.0 - 25.6	33.1	29.0 - 39.8	7.69	7.27 - 8.12
12-19	sU	DK	DYMS 2017	2017	M	76	0.25	0.25	1.32	3.46	2.70 - 4.10	5.68	4.48 - 7.92	9.12	7.04 - 15.8	11.9	9.15 - 34.2	3.32	2.73 - 4.04
20-39	24hU	DE	ESB 2007	2007	F,M	60		0.25	0.00	8.15	5.72 - 9.10	11.2	9.34 - 14.0	17.4	12.8 - 27.5	24.0		7.66	6.51 - 9.00
20-39	24hU	DE	ESB 2009	2009	F,M	60		0.25	0.00	5.90	4.70 - 7.69	10.7	7.70 - 14.9	20.5	12.8 - 36.5	27.3		6.58	5.38 - 8.05
20-39	mU	DE	DEMOCOPHES-DE	2011	F	64		0.25	0.00	10.4	7.56 - 13.0	16.2	14.3 - 18.2	19.1	17.4 - 29.6	27.7		9.68	8.21 - 11.4
20-39	24hU	DE	ESB 2011	2011	F,M	60		0.25	0.00	5.75	4.50 - 7.50	9.07	7.66 - 12.3	13.2	11.2 - 26.2	22.0		6.01	5.04 - 7.16
20-39	24hU	DE	ESB 2013	2013	F,M	60		0.25	0.00	4.60	3.90 - 5.79	7.25	5.80 - 9.14	10.5	8.46 - 13.2	12.2		4.64	3.95 - 5.46
20-39	24hU	DE	ESB 2015	2015	F,M	60		0.25	0.00	2.80	2.50 - 3.00	3.80	3.08 - 5.07	5.96	4.92 - 18.4	11.9		2.90	2.29 - 3.67
40-59	mU	DE	DEMOCOPHES-DE	2011	F	54		0.25	0.00	6.30	5.34 - 8.16	11.9	8.16 - 25.6	26.3	19.8 - 58.3	30.9		7.70	6.09 - 9.74

Notes. Matrix: mU - first morning urine, sU - spot urine, 24hU - 24-hours urine; country: AT - Austria, BE - Belgium, CZ - Czech Republic, DE - Germany, DK - Denmark, ES - Spain, HU - Hungary, IL - Israel, LU - Luxemburg, NO - Norway, PL - Poland, SE - Sweden, SI - Slovenia, SK - Slovakia; sex: F - female, M - male; n - sample size; LOD - limit of detection; LOQ - limit of quantification; % below limit - percentage of values below limit of detection/quantification, whichever was applied in the data collection; P50, P75, P90, P95 - 50th, 75th, 90th, 95th percentile; 95% CI - 95 % confidence interval; GM - geometric mean; NA - Not available.

Data only displayed if n was at least 50; percentiles missing when below LOD/LOQ; CIs of the observed percentiles missing when they fall outside the observed range; GM was imputed (fixed imputation) based on a log-normal distribution when at least 50 % of values were above LOD/LOQ.

Table S10: DEP metabolite MEP (Mono-ethyl phthalate) in microg/L

Age	Matrix	Country	Data collection	Sampling year	Sex	N	LOD	LOQ	% below limit	P50	P50_95% CI	P75	P75_95% CI	P90	P90_95% CI	P95	P95_95% CI	GM	GM_95% CI
3-5	sU	DK	CPH-MC	2006	F,M	189	0.24		0.00	24.7	20.8 - 28.8	50.1	39.6 - 61.6	98.5	75.7 - 120	128	107 - 235	24.9	21.4 - 28.9
3-5	mU	DE	GerES V	2015 - 2017	F,M	388		0.50	0.00	18.6	16.6 - 20.8	35.7	32.2 - 40.8	64.7	57.4 - 84.4	97.2	82.5 - 175	19.3	17.4 - 21.3
3-5	sU	SK	Children cohort	2015 - 2016	F,M	67		0.50	0.00	20.5	12.2 - 25.3	34.8	28.6 - 50.0	84.8	45.0 - 113	91.9		17.8	13.8 - 23.0
6-11	sU	DK	CPH-MC	2006 - 2007	F,M	659	0.24		0.00	19.6	17.9 - 21.5	38.3	34.4 - 44.0	74.1	66.0 - 91.4	134	102 - 154	19.8	18.3 - 21.6
6-11	mU	DK	CPHPUB-Cross	2006 - 2008	F,M	913	0.53		0.00	35.9	33.0 - 38.6	67.6	60.7 - 72.3	114	105 - 135	197	167 - 222	39.3	37.1 - 41.7
6-11	sU	AT	PBAT	2010 - 2012	F,M	165	2.50	5.00	12.7	16.9	13.5 - 20.1	30.6	25.6 - 39.2	53.7	46.2 - 98.3	102	59.5 - 177	16.9	14.4 - 19.7
6-11	mU	SE	DEMOCOPHES-SE	2011 - 2012	F,M	98		0.30	0.00	24.4	20.8 - 32.5	45.6	36.0 - 57.2	100	57.6 - 207	149	114 - 687	28.7	23.6 - 34.8
6-11	mU	DK	DEMOCOPHES-DK	2011	F,M	143		0.53	0.00	19.6	16.1 - 21.9	33.8	27.6 - 40.9	60.6	47.8 - 67.8	67.8	62.1 - 125	20.1	17.6 - 22.8
6-11	mU	ES	DEMOCOPHES-ES	2011 - 2012	F,M	120		0.50	0.00	169	136 - 203	347	275 - 489	708	522 - 1062	1027	751 - 2628	180	149 - 219
6-11	mU	SL	DEMOCOPHES-SI	2011 - 2012	F,M	119		0.50	0.00	38.0	33.0 - 45.8	91.0	67.0 - 122	159	125 - 240	239	165 - 342	42.5	35.1 - 51.5
6-11	mU	LU	DEMOCOPHES-LU	2011 - 2012	F,M	60		0.50	0.00	20.5	17.0 - 31.9	43.2	32.8 - 54.1	74.0	51.6 - 174	120		23.6	18.7 - 29.9
6-11	mU	DE	DEMOCOPHES-DE	2011	F,M	119		0.50	0.00	20.2	17.1 - 24.8	35.8	30.3 - 43.0	54.7	45.5 - 87.1	86.6	59.4 - 441	22.8	19.6 - 26.6
6-11	mU	BE	DEMOCOPHES-BE	2011 - 2012	F,M	129		0.50	0.00	23.0	19.0 - 29.0	41.0	33.4 - 63.0	101	70.6 - 168	167	105 - 638	25.7	21.2 - 31.2
6-11	mU	HU	DEMOCOPHES-HU	2011 - 2012	F,M	120		0.64	0.00	48.5	34.5 - 56.3	104	82.4 - 139	195	149 - 312	288	207 - 459	45.4	36.6 - 56.1
6-11	mU	PL	DEMOCOPHES-PL	2011 - 2012	F,M	120		0.50	0.00	42.0	29.9 - 48.3	70.5	57.0 - 109	213	145 - 252	252	224 - 429	42.2	35.0 - 50.9
6-11	mU	CZ	DEMOCOPHES-CZ	2011 - 2012	F,M	120		0.64	0.00	31.0	21.0 - 40.4	53.5	44.8 - 79.5	103	84.0 - 178	168	118 - 420	31.6	26.3 - 38.0
6-11	mU	SK	DEMOCOPHES-SK	2011 - 2012	F,M	129		0.64	0.00	42.2	35.5 - 51.0	73.7	60.6 - 94.1	122	97.9 - 183	183	147 - 290	39.8	33.6 - 47.1
6-11	mU	SK	PCBcohort_c	2014 - 2016	F,M	249	3.23		0.00	30.8	27.0 - 34.6	69.1	58.8 - 92.8	171	138 - 269	294	210 - 547	37.4	32.4 - 43.1
6-11	mU	DE	GerES V	2015 - 2017	F,M	937		0.50	0.11	20.3	18.5 - 21.7	38.5	36.0 - 43.6	89.4	77.6 - 105	161	120 - 185	22.4	20.9 - 24.0
6-11	sU	SK	Children cohort	2015 - 2016	F,M	112		0.50	0.00	19.3	17.5 - 27.2	46.4	34.5 - 76.3	113	80.1 - 186	175	125 - 408	23.3	18.6 - 29.1
12-19	mU	DK	CPHPUB-Cross	2006 - 2008	F,M	389	0.53		0.00	46.3	40.2 - 52.4	110	93.4 - 128	290	239 - 422	713	420 - 1151	58.3	51.4 - 66.1
12-19	sU	DK	DYMS 2007	2007	M	239	0.53	0.53	0.00	95.6	74.7 - 109	248	192 - 404	1119	729 - 1771	2480	1582 - 2801	114	93.7 - 139
12-19	sU	DK	DYMS 2008	2008	M	261	0.53	0.53	0.00	92.9	69.5 - 123	321	251 - 488	1214	778 - 1643	1872	1593 - 2836	114	93.3 - 138
12-19	sU	DK	DYMS 2009	2009	M	210	0.43	0.43	0.00	55.3	43.2 - 69.3	198	133 - 300	767	490 - 1022	1352	892 - 2965	75.7	61.4 - 93.2
12-19	sU	AT	PBAT	2010 - 2012	F,M	94	2.50	5.00	5.32	24.3	19.5 - 31.6	57.9	38.6 - 75.0	90.7	75.5 - 258	209	97.2 - 460	27.3	22.0 - 33.9
12-19	sU	DK	DYMS 2013	2013	M	70	0.43	0.43	0.00	27.8	19.9 - 37.4	64.5	39.7 - 81.4	152	80.9 - 534	309		30.8	23.1 - 41.1
12-19	sU	BE	FLEHS3RefAdo	2013	F,M	207		0.50	0.00	32.3	25.7 - 40.2	80.8	61.3 - 102	371	147 - 562	589	441 - 958	39.3	32.6 - 47.4
12-19	mU	DE	GerES V	2015 - 2017	F,M	932		0.50	0.00	26.2	23.7 - 28.4	64.8	55.9 - 71.1	148	129 - 193	299	227 - 410	31.2	28.9 - 33.6
12-19	mU	SK	PCBcohort_t	2015 - 2017	F,M	65	3.23		3.08	18.9	14.1 - 27.4	44.4	31.6 - 67.0	90.8	63.3 - 211	149		21.6	16.4 - 28.5
12-19	sU	DK	DYMS 2017	2017	M	76	0.43	0.43	0.00	21.1	16.4 - 29.7	48.0	30.0 - 62.7	122	62.3 - 336	212	123 - 727	25.1	19.6 - 32.2
20-39	24hU	DE	ESB 2007	2007	F,M	60		0.50	0.00	53.5	30.7 - 91.5	134	91.5 - 256	294	187 - 851	400		54.3	38.5 - 76.7
20-39	sU	DK	DYMS 2008	2008	M	54	0.53	0.53	0.00	71.1	46.6 - 121	205	121 - 394	470	245 - 1596	589		80.4	56.2 - 115
20-39	sU	DK	DYMS 2009	2009	M	79	0.43	0.43	0.00	68.6	55.1 - 116	250	137 - 511	1049	432 - 3676	2172	1074 - 7458	99.1	69.0 - 142
20-39	24hU	DE	ESB 2009	2009	F,M	60		0.50	0.00	37.5	26.7 - 52.5	75.3	54.2 - 125	150	107 - 542	318		38.6	28.6 - 52.1

Age	Matrix	Country	Data collection	Sampling year	Sex	N	LOD	LOQ	% below limit	P50	P50_95% CI	P75	P75_95% CI	P90	P90_95% CI	P95	P95_95% CI	GM	GM_95% CI
20-39	sU	AT	PBAT	2010 - 2011	F,M	135	2.50	5.00	5.19	28.3	21.8 - 38.9	80.8	59.5 - 104	171	125 - 358	357	202 - 472	32.2	26.0 - 39.9
20-39	mU	SE	DEMOCOPHES-SE	2011 - 2012	F	50		0.30	0.00	40.9	24.7 - 60.0	91.8	56.6 - 170	188	127 - 303	203		41.3	30.7 - 55.6
20-39	mU	DK	DEMOCOPHES-DK	2011	F	51		0.53	0.00	34.7	16.8 - 63.6	93.8	61.4 - 192	207	128 - 539	424		38.0	26.5 - 54.4
20-39	mU	SL	DEMOCOPHES-SI	2011 - 2012	F	63		0.50	0.00	45.0	38.0 - 83.2	139	95.7 - 241	255	213 - 627	353		56.8	41.6 - 77.6
20-39	mU	DE	DEMOCOPHES-DE	2011	F	64		0.50	0.00	27.8	22.9 - 41.7	58.9	44.6 - 102	133	83.5 - 249	190		31.8	24.6 - 41.1
20-39	mU	BE	DEMOCOPHES-BE	2011 - 2012	F	62		0.50	0.00	33.5	19.9 - 51.1	73.2	52.4 - 98.9	166	91.1 - 200	197		30.4	22.7 - 40.8
20-39	24hU	DE	ESB 2011	2011	F,M	60		0.50	0.00	17.9	13.7 - 38.4	64.2	38.8 - 115	149	85.4 - 599	190		26.7	18.7 - 38.3
20-39	mU	HU	DEMOCOPHES-HU	2011 - 2012	F	89		0.64	0.00	51.3	39.1 - 71.6	133	91.6 - 206	326	200 - 603	537	349 - 3941	62.7	47.9 - 82.0
20-39	mU	PL	DEMOCOPHES-PL	2011 - 2012	F	88		0.50	0.00	31.5	18.0 - 42.0	76.0	47.4 - 186	227	184 - 508	392	228 - 1813	37.0	27.8 - 49.1
20-39	mU	CZ	DEMOCOPHES-CZ	2011 - 2012	F	88		0.64	0.00	38.0	29.8 - 64.7	108	80.4 - 154	196	153 - 539	432	221 - 2433	44.7	33.7 - 59.3
20-39	mU	SK	DEMOCOPHES-SK	2011 - 2012	F	91		0.64	0.00	51.0	40.8 - 83.5	131	99.4 - 190	219	189 - 733	438	221 - 1291	55.9	43.0 - 72.6
20-39	24hU	DE	ESB 2013	2013	F,M	60		0.50	0.00	20.9	13.6 - 25.2	52.3	26.8 - 69.3	75.2	60.2 - 114	93.3		20.3	15.4 - 26.7
20-39	24hU	DE	ESB 2015	2015	F,M	60		0.50	0.00	13.5	8.32 - 21.2	31.7	21.3 - 52.0	55.5	43.8 - 91.5	71.2		14.1	10.6 - 18.8
40-59	sU	AT	PBAT	2010 - 2011	F,M	112	2.50	5.00	8.04	39.4	22.0 - 54.3	109	73.0 - 174	239	179 - 422	419	248 - 626	36.9	28.1 - 48.3
40-59	mU	DK	DEMOCOPHES-DK	2011	F	94		0.53	0.00	26.7	21.8 - 39.5	55.9	46.3 - 88.1	143	89.3 - 371	319	147 - 538	31.0	24.6 - 39.1
40-59	mU	ES	DEMOCOPHES-ES	2011 - 2012	F	75		0.50	0.00	141	102 - 207	267	218 - 437	638	423 - 1385	1233	639 - 1665	136	103 - 180
40-59	mU	SL	DEMOCOPHES-SI	2011 - 2012	F	57		0.50	0.00	46.0	33.7 - 67.8	82.0	69.1 - 176	186	131 - 360	245		47.8	36.5 - 62.5
40-59	mU	DE	DEMOCOPHES-DE	2011	F	56		0.50	0.00	42.0	26.0 - 65.9	96.3	66.3 - 166	181	137 - 726	445		46.8	34.3 - 63.9
40-59	mU	BE	DEMOCOPHES-BE	2011 - 2012	F	67		0.50	0.00	44.0	29.0 - 67.0	94.0	70.0 - 136	190	123 - 535	441		43.9	32.7 - 58.9
60+	sU	AT	PBAT	2010 - 2012	F,M	89	2.50	5.00	6.74	38.6	29.2 - 52.1	92.4	71.5 - 170	343	169 - 1014	994	443 - 1879	45.4	33.0 - 62.6

Notes. Matrix: mU - first morning urine, sU - spot urine, 24hU - 24-hours urine; country: AT - Austria, BE - Belgium, CZ - Czech Republic, DE - Germany, DK - Denmark, ES - Spain, HU - Hungary, IL - Israel, LU - Luxemburg, NO - Norway, PL - Poland, SE - Sweden, SI - Slovenia, SK - Slovakia; sex: F - female, M - male; n - sample size; LOD - limit of detection; LOQ - limit of quantification; % below limit - percentage of values below limit of detection/quantification, whichever was applied in the data collection; P50, P75, P90, P95 - 50th, 75th, 90th, 95th percentile; 95% CI - 95 % confidence interval; GM - geometric mean; NA - Not available.

Data only displayed if n was at least 50; percentiles missing when below LOD/LOQ; CIs of the observed percentiles missing when they fall outside the observed range; GM was imputed (fixed imputation) based on a log-normal distribution when at least 50 % of values were above LOD/LOQ.

Table S11: DMP metabolite MMP (Mono-methyl phthalate) in microg/L

Age	Matrix	Country	Data collection	Sampling year	Sex	N	LOD	LOQ	% below limit	P50	P50_95% CI	P75	P75_95% CI	P90	P90_95% CI	P95	P95_95% CI	GM	GM_95% CI
3-5	mU	DE	GerES V	2015 - 2017	F,M	388		1.00	2.32	6.70	5.60 - 7.30	12.2	10.6 - 14.7	30.8	23.2 - 43.7	66.0	39.7 - 115	7.04	6.28 - 7.89
3-5	sU	SK	Children cohort	2015 - 2016	F,M	67		1.00	0.00	1.88		3.71		6.62		8.92			
6-11	mU	DE	DEMOCOPHES-DE	2011	F,M	119		1.00	15.1	5.89	4.29 - 7.34	12.1	9.14 - 17.2	29.6	19.5 - 45.5	43.8	31.8 - 149	5.01	3.86 - 6.49
6-11	mU	HU	DEMOCOPHES-HU	2011 - 2012	F,M	120		4.73	53.3			11.6	8.56 - 17.4	27.7	19.4 - 63.3	56.2	38.1 - 307		
6-11	mU	CZ	DEMOCOPHES-CZ	2011 - 2012	F,M	120		4.73	58.3			8.55	6.31 - 10.6	18.6	11.0 - 30.3	28.6	19.3 - 237		
6-11	mU	SK	DEMOCOPHES-SK	2011 - 2012	F,M	129		4.73	69.8			6.47		13.1	9.83 - 38.6	38.3	15.8 - 97.2		
6-11	mU	SK	PCBcohort_c	2014 - 2016	F,M	249	3.40		37.4	3.60	3.60 - 3.82	3.82	3.82 - 3.82	11.4	8.78 - 15.2	18.1	14.1 - 30.5	3.34	2.98 - 3.74
6-11	mU	DE	GerES V	2015 - 2017	F,M	937		1.00	1.39	6.80	6.20 - 7.40	13.7	12.5 - 15.4	28.0	24.7 - 34.4	47.0	38.9 - 60.9	7.39	6.91 - 7.90
6-11	sU	SK	Children cohort	2015 - 2016	F,M	112		1.00	0.00	3.27		6.75		10.7		19.7			
12-19	sU	DK	DYMS 2009	2009	M	69	0.27	0.27	7.25	2.52	2.05 - 3.03	3.61	3.21 - 5.06	6.46	4.99 - 17.6	9.24		2.33	1.80 - 3.03
12-19	sU	DK	DYMS 2013	2013	M	70	0.27	0.27	8.57	2.32	1.96 - 3.06	3.82	3.18 - 4.91	5.28	4.50 - 7.97	6.64		2.08	1.68 - 2.59
12-19	mU	DE	GerES V	2015 - 2017	F,M	932		1.00	2.68	5.80	5.40 - 6.18	11.0	10.1 - 12.2	19.8	18.6 - 21.9	30.7	25.3 - 37.2	6.13	5.77 - 6.52
12-19	mU	SK	PCBcohort_t	2015 - 2017	F,M	65	3.40		70.8			3.81		9.78	5.10 - 21.0	13.4			
12-19	sU	DK	DYMS 2017	2017	M	76	0.27	0.27	17.1	1.84	1.68 - 2.30	2.74	2.39 - 3.74	4.24	3.52 - 5.94	5.34	4.26 - 8.69	1.49	1.19 - 1.87
20-39	24hU	DE	ESB 2007	2007	F,M	59		1.00	5.08	8.00	4.70 - 10.3	22.8	10.8 - 43.0	62.5	35.7 - 122	102		7.98	5.52 - 11.6
20-39	24hU	DE	ESB 2009	2009	F,M	59		1.00	11.9	7.10	4.10 - 12.2	15.2	12.4 - 26.1	29.6	16.4 - 114	54.6		6.12	4.27 - 8.78
20-39	mU	DE	DEMOCOPHES-DE	2011	F	64		1.00	34.4	1.76	1.26 - 3.24	8.24	3.52 - 10.7	11.3	9.24 - 15.4	12.6		2.15	1.56 - 2.96
20-39	24hU	DE	ESB 2011	2011	F,M	60		1.00	1.67	4.35	3.70 - 6.99	8.22	7.08 - 11.6	13.3	9.83 - 40.1	35.1		5.16	4.14 - 6.44
20-39	mU	HU	DEMOCOPHES-HU	2011 - 2012	F	89		4.73	69.7			8.26		45.7	15.2 - 144	124	46.4 - 619		
20-39	mU	CZ	DEMOCOPHES-CZ	2011 - 2012	F	88		4.73	80.7					6.70	5.41 - 15.4	13.5	7.25 - 273		
20-39	mU	SK	DEMOCOPHES-SK	2011 - 2012	F	91		4.73	89.0							9.10	5.93 - 44.2		
20-39	24hU	DE	ESB 2013	2013	F,M	60		1.00	1.67	3.50	2.70 - 4.49	6.12	4.50 - 10.9	16.8	9.10 - 31.1	29.2		4.19	3.32 - 5.30
20-39	24hU	DE	ESB 2015	2015	F,M	60		1.00	5.00	2.75	2.20 - 3.99	5.12	4.00 - 7.04	9.11	6.65 - 17.9	14.3		3.16	2.58 - 3.86
40-59	mU	DE	DEMOCOPHES-DE	2011	F	56		1.00	32.1	2.49	1.11 - 5.64	8.57	5.66 - 22.1	36.2	13.0 - 71.3	59.2		2.44	1.50 - 3.98

Notes. Matrix: mU - first morning urine, sU - spot urine, 24hU - 24-hours urine; country: AT - Austria, BE - Belgium, CZ - Czech Republic, DE - Germany, DK - Denmark, ES - Spain, HU - Hungary, IL - Israel, LU - Luxemburg, NO - Norway, PL - Poland, SE - Sweden, SI - Slovenia, SK - Slovakia; sex: F - female, M - male; n - sample size; LOD - limit of detection; LOQ - limit of quantification; % below limit - percentage of values below limit of detection/quantification, whichever was applied in the data collection; P50, P75, P90, P95 - 50th, 75th, 90th, 95th percentile; 95% CI - 95 % confidence interval; GM - geometric mean; NA - Not available.

Data only displayed if n was at least 50; percentiles missing when below LOD/LOQ; CIs of the observed percentiles missing when they fall outside the observed range; GM was imputed (fixed imputation) based on a log-normal distribution when at least 50 % of values were above LOD/LOQ.

Table S12: Metabolite 3cx-MPP (3-carboxyl-mono-propyl phthalate) in microg/L

Age	Matrix	Country	Data collection	Sampling year	Sex	N	LOD	LOQ	% below limit	P50	P50_95% CI	P75	P75_95% CI	P90	P90_95% CI	P95	P95_95% CI	GM	GM_95% CI
3-5	mU	DE	GerES V	2015 - 2017	F,M	388		0.50	2.06	2.10	2.00 - 2.30	3.50	3.30 - 3.90	5.20	4.60 - 6.40	7.10	6.40 - 9.45	2.14	1.98 - 2.31
6-11	mU	DK	CPHPUB-Cross	2006 - 2008	F,M	779	0.36		0.00	9.97	9.29 - 10.5	15.6	14.4 - 16.5	23.0	21.5 - 26.0	31.3	27.8 - 38.1	9.89	9.40 - 10.4
6-11	mU	DK	DEMOCOPHES-DK	2011	F,M	143		0.36	0.00	7.44	6.51 - 8.63	12.8	10.8 - 15.5	29.2	18.5 - 62.1	64.7	36.0 - 120	8.67	7.46 - 10.1
6-11	mU	DE	DEMOCOPHES-DE	2011	F,M	119		0.50	4.20	1.50	1.38 - 1.67	2.38	2.15 - 3.00	3.81	3.32 - 6.25	6.21	4.07 - 11.9	1.64	1.43 - 1.88
6-11	mU	DE	GerES V	2015 - 2017	F,M	937		0.50	4.80	1.70	1.60 - 1.80	2.60	2.50 - 2.80	4.30	3.94 - 4.90	6.42	5.90 - 7.40	1.71	1.63 - 1.79
12-19	mU	DK	CPHPUB-Cross	2006 - 2008	F,M	389	0.36		0.26	6.90	6.41 - 7.58	11.1	9.89 - 12.3	16.8	15.0 - 19.2	22.0	18.8 - 32.3	6.86	6.31 - 7.46
12-19	mU	DE	GerES V	2015 - 2017	F,M	932		0.50	13.4	1.10	1.00 - 1.10	1.80	1.70 - 2.00	3.00	2.80 - 3.40	4.20	3.70 - 4.90	1.14	1.09 - 1.20
20-39	mU	DK	DEMOCOPHES-DK	2011	F	51		0.36	1.96	5.82	4.34 - 6.63	7.71	6.60 - 9.01	12.2	8.03 - 25.9	15.6		4.68	3.65 - 6.01
20-39	mU	DE	DEMOCOPHES-DE	2011	F	64		0.50	17.2	0.83	0.73 - 1.09	1.39	1.15 - 1.95	2.66	1.86 - 4.02	3.35		0.94	0.78 - 1.12
40-59	mU	DK	DEMOCOPHES-DK	2011	F	94		0.36	0.00	3.12	2.61 - 4.27	5.76	4.68 - 6.96	8.52	7.03 - 11.4	10.6	8.54 - 24.7	3.26	2.78 - 3.82
40-59	mU	DE	DEMOCOPHES-DE	2011	F	56		0.50	32.1	0.64	0.51 - 0.76	1.23	0.77 - 1.64	1.95	1.42 - 4.00	2.83		0.67	0.54 - 0.83

Notes. Matrix: mU - first morning urine, sU - spot urine, 24hU - 24-hours urine; country: AT - Austria, BE - Belgium, CZ - Czech Republic, DE - Germany, DK - Denmark, ES - Spain, HU - Hungary, IL - Israel, LU - Luxemburg, NO - Norway, PL - Poland, SE - Sweden, SI - Slovenia, SK - Slovakia; sex: F - female, M - male; n - sample size; LOD - limit of detection; LOQ - limit of quantification; % below limit - percentage of values below limit of detection/quantification, whichever was applied in the data collection; P50, P75, P90, P95 - 50th, 75th, 90th, 95th percentile; 95% CI - 95 % confidence interval; GM - geometric mean; NA - Not available.

Data only displayed if n was at least 50; percentiles missing when below LOD/LOQ; CIs of the observed percentiles missing when they fall outside the observed range; GM was imputed (fixed imputation) based on a log-normal distribution when at least 50 % of values were above LOD/LOQ.

Table S13: DiNP metabolite cx-MiNP (7-Carboxy-(mono-methyl- heptyl) phthalate) in microg/L

Age	Matrix	Country	Data collection	Sampling year	Sex	N	LOD	LOQ	% below limit	P50	P50_95% CI	P75	P75_95% CI	P90	P90_95% CI	P95	P95_95% CI	GM	GM_95% CI
3-5	mU	DE	GerES IV 2005	2005	F,M	50		0.25	0.00	21.5	18.1 - 32.8	39.5	32.1 - 49.3	53.1	45.6 - 139	57.5		21.4	16.9 - 27.2
3-5	sU	DK	CPH-MC	2006	F,M	189	0.08		0.00	6.75	5.97 - 8.66	12.8	11.9 - 14.6	21.6	16.9 - 28.9	30.3	22.8 - 60.5	7.22	6.33 - 8.24
3-5	mU	DE	GerES V	2015 - 2017	F,M	388		0.20	0.00	6.70	6.12 - 7.38	12.4	10.9 - 13.6	19.4	17.9 - 23.3	29.3	22.7 - 43.6	7.01	6.44 - 7.62
3-5	sU	SK	Children cohort	2015 - 2016	F,M	67		0.20	0.00	6.35	4.57 - 8.67	12.2	10.6 - 15.2	21.5	14.3 - 64.1	45.0		6.05	4.55 - 8.04
6-11	mU	DE	GerES IV 2005	2005	F,M	102		0.25	0.00	18.3	14.6 - 22.1	31.3	28.0 - 37.3	46.4	39.1 - 81.4	78.7	51.2 - 165	18.0	15.3 - 21.3
6-11	sU	DK	CPH-MC	2006 - 2007	F,M	659	0.08		0.00	6.94	6.51 - 7.56	11.7	10.9 - 12.5	19.1	16.8 - 22.1	27.0	24.1 - 36.0	6.74	6.24 - 7.29
6-11	mU	DK	CPHPUB-Cross	2006 - 2008	F,M	913	0.11		0.00	8.91	8.30 - 9.42	14.0	13.4 - 15.2	24.2	21.9 - 26.2	34.4	30.6 - 41.6	9.08	8.62 - 9.56
6-11	mU	SE	DEMOCOPHES-SE	2011 - 2012	F,M	98		0.30	0.00	19.9	16.1 - 23.4	35.0	26.7 - 54.6	70.7	55.8 - 219	183	82.8 - 819	22.0	17.8 - 27.1
6-11	mU	DK	DEMOCOPHES-DK	2011	F,M	143		0.11	0.00	7.84	6.80 - 8.98	12.7	10.5 - 16.2	22.8	20.2 - 44.8	45.4	28.0 - 107	8.59	7.36 - 10.0
6-11	mU	ES	DEMOCOPHES-ES	2011 - 2012	F,M	120		0.20	0.00	13.2	11.2 - 15.6	21.7	17.9 - 27.0	41.4	30.0 - 53.0	46.8	43.7 - 91.9	13.7	12.0 - 15.8
6-11	mU	LU	DEMOCOPHES-LU	2011 - 2012	F,M	59		0.20	0.00	8.52	7.37 - 10.2	14.1	10.5 - 20.5	22.1	18.0 - 48.9	29.0		8.74	6.99 - 10.9
6-11	mU	DE	DEMOCOPHES-DE	2011	F,M	119		0.20	0.00	7.90	6.55 - 8.84	14.2	11.0 - 19.5	23.2	21.4 - 39.9	38.1	24.2 - 425	8.83	7.31 - 10.7
6-11	mU	BE	DEMOCOPHES-BE	2011 - 2012	F,M	117		0.10	0.85	1.21	1.07 - 1.43	2.22	1.69 - 2.61	4.47	2.73 - 8.17	8.00	5.11 - 30.5	1.34	1.12 - 1.60
6-11	mU	PL	DEMOCOPHES-PL	2011 - 2012	F,M	94	0.05	0.15	0.00	3.48	3.11 - 4.44	5.35	4.81 - 7.78	11.1	7.86 - 23.8	20.4	11.9 - 143	3.90	3.23 - 4.71
6-11	mU	CZ	DEMOCOPHES-CZ	2011 - 2012	F,M	118	0.20	0.40	0.00	7.57	6.53 - 9.04	13.8	10.3 - 17.8	27.3	18.2 - 45.9	45.8	31.0 - 75.9	8.17	6.99 - 9.56
6-11	mU	NO	IES	2012	F,M	50	0.40	1.00	0.00	4.34	3.48 - 5.32	6.62	5.20 - 8.68	10.6	7.06 - 22.0	14.1		4.80	4.06 - 5.68
6-11	mU	DE	GerES V	2015 - 2017	F,M	935		0.20	0.11	5.70	5.40 - 6.10	9.75	9.00 - 10.7	17.4	16.3 - 20.8	28.3	23.6 - 33.0	6.18	5.85 - 6.54
6-11	sU	SK	Children cohort	2015 - 2016	F,M	112		0.20	0.00	8.34	7.05 - 9.75	12.5	11.6 - 17.3	22.3	18.4 - 35.4	31.6	22.5 - 76.9	7.80	6.54 - 9.31
12-19	mU	DE	GerES IV 2005	2005	F,M	51		0.25	0.00	10.7	8.91 - 16.3	21.9	16.1 - 27.7	30.3	24.7 - 63.9	42.5		12.4	10.0 - 15.3
12-19	mU	DK	CPHPUB-Cross	2006 - 2008	F,M	389	0.11		0.00	6.01	5.60 - 7.04	10.9	9.59 - 12.3	19.2	16.5 - 22.1	26.6	21.9 - 38.3	6.34	5.81 - 6.92
12-19	sU	DK	DYMS 2007	2007	M	239	0.11	0.11	0.84	6.69	5.78 - 7.63	11.5	10.3 - 13.0	19.5	17.6 - 26.3	28.8	22.6 - 65.0	6.77	5.97 - 7.68
12-19	sU	DK	DYMS 2008	2008	M	261	0.11	0.11	1.53	8.08	7.35 - 9.35	14.0	11.9 - 15.5	24.7	19.6 - 29.9	35.4	28.4 - 61.9	7.84	6.93 - 8.87
12-19	sU	DK	DYMS 2009	2009	M	210	0.04	0.04	1.43	7.61	6.76 - 8.69	12.6	11.5 - 14.6	21.9	17.9 - 29.2	33.0	24.0 - 44.0	6.64	5.70 - 7.73
12-19	sU	DK	DYMS 2013	2013	M	70	0.04	0.04	0.00	5.63	4.69 - 7.01	11.8	7.47 - 17.0	24.6	16.8 - 71.0	55.6		6.10	4.63 - 8.04
12-19	mU	DE	GerES V	2015 - 2017	F,M	931		0.20	0.11	4.60	4.31 - 4.90	7.50	7.10 - 8.00	12.9	11.3 - 14.7	20.1	18.0 - 24.8	4.73	4.46 - 5.01
12-19	sU	DK	DYMS 2017	2017	M	76	0.04	0.04	0.00	3.75	3.35 - 4.38	6.41	4.63 - 7.87	12.2	7.63 - 59.9	49.7	12.4 - 335	4.48	3.49 - 5.76
20-39	24hU	DE	ESB 2007	2007	F,M	60		0.20	0.00	2.00	1.50 - 2.88	3.95	2.90 - 5.30	6.41	4.92 - 9.60	8.01		2.23	1.83 - 2.72
20-39	sU	DK	DYMS 2008	2008	M	54	0.11	0.11	0.00	9.48	8.22 - 10.9	13.7	10.9 - 18.5	21.9	16.0 - 53.6	31.5		9.54	7.67 - 11.9
20-39	24hU	DE	ESB 2008	2008	F,M	54		0.50	7.41	3.49	3.28 - 4.55	6.66	4.55 - 10.7	13.3	8.57 - 30.8	25.1		3.93	2.99 - 5.17
20-39	sU	DK	DYMS 2009	2009	M	79	0.04	0.04	1.27	8.04	7.03 - 10.9	14.5	12.1 - 21.0	41.2	21.0 - 77.6	54.3	41.2 - 216	8.95	7.02 - 11.4
20-39	24hU	DE	ESB 2009	2009	F,M	60		0.20	0.00	2.80	2.00 - 3.90	5.05	3.98 - 8.29	9.70	7.13 - 12.7	11.9		2.92	2.28 - 3.74
20-39	mU	SE	DEMOCOPHES-SE	2011 - 2012	F	50		0.30	0.00	17.2	12.9 - 32.7	73.3	30.9 - 164	187	122 - 790	326		26.6	17.8 - 39.9
20-39	mU	DK	DEMOCOPHES-DK	2011	F	51		0.11	0.00	7.33	5.26 - 11.7	14.0	11.6 - 21.1	22.0	16.3 - 72.7	48.1		7.25	5.40 - 9.75
20-39	mU	DE	DEMOCOPHES-DE	2011	F	64		0.20	0.00	6.05	4.84 - 7.46	9.34	7.61 - 14.2	20.9	12.6 - 39.2	32.7		6.59	5.16 - 8.42

Age	Matrix	Country	Data collection	Sampling year	Sex	N	LOD	LOQ	% below limit	P50	P50_95% CI	P75	P75_95% CI	P90	P90_95% CI	P95	P95_95% CI	GM	GM_95% CI
20-39	24hU	DE	ESB 2011	2011	F,M	60		0.20	0.00	3.70	3.00 - 4.30	5.20	4.30 - 9.36	11.3	6.77 - 80.1	20.4		3.95	3.04 - 5.13
20-39	sU	IL	IBS	2011	F,M	144	0.25	0.50	0.69	9.64	8.50 - 13.1	18.6	16.9 - 22.5	29.7	24.5 - 58.4	58.6	31.3 - 69.5	10.1	8.65 - 11.9
20-39	24hU	DE	ESB 2013	2013	F,M	60		0.20	0.00	2.30	1.80 - 2.70	3.73	2.78 - 5.17	5.64	4.76 - 12.0	9.62		2.41	1.97 - 2.95
20-39	24hU	DE	ESB 2015	2015	F,M	60		0.20	0.00	2.00	1.80 - 2.50	3.90	2.58 - 7.59	8.02	6.57 - 14.1	12.5		2.50	2.00 - 3.13
40-59	mU	DK	DEMOCOPHES-DK	2011	F	94		0.11	0.00	5.13	3.67 - 6.60	8.54	7.15 - 11.4	17.6	11.5 - 35.5	30.2	18.2 - 49.1	5.12	4.23 - 6.18
40-59	mU	ES	DEMOCOPHES-ES	2011 - 2012	F	75		0.20	0.00	10.0	8.13 - 11.0	15.6	11.5 - 20.7	28.2	19.3 - 43.5	42.4	28.2 - 46.5	9.55	8.01 - 11.4
40-59	mU	DE	DEMOCOPHES-DE	2011	F	56		0.20	0.00	4.00	3.20 - 5.18	7.41	5.20 - 15.3	18.4	9.50 - 35.6	27.0		4.53	3.55 - 5.78
40-59	sU	IL	IBS	2011	F,M	86	0.25	0.50	1.16	9.51	7.47 - 10.9	15.7	12.4 - 20.3	29.6	20.0 - 55.2	44.6	30.1 - 69.8	9.03	7.36 - 11.1

Notes. Matrix: mU - first morning urine, sU - spot urine, 24hU - 24-hours urine; country: AT - Austria, BE - Belgium, CZ - Czech Republic, DE - Germany, DK - Denmark, ES - Spain, HU - Hungary, IL - Israel, LU - Luxemburg, NO - Norway, PL - Poland, SE - Sweden, SI - Slovenia, SK - Slovakia; sex: F - female, M - male; n - sample size; LOD - limit of detection; LOQ - limit of quantification; % below limit - percentage of values below limit of detection/quantification, whichever was applied in the data collection; P50, P75, P90, P95 - 50th, 75th, 90th, 95th percentile; 95% CI - 95 % confidence interval; GM - geometric mean; NA - Not available.

Data only displayed if n was at least 50; percentiles missing when below LOD/LOQ; CIs of the observed percentiles missing when they fall outside the observed range; GM was imputed (fixed imputation) based on a log-normal distribution when at least 50 % of values were above LOD/LOQ.

Table S14: DiNP metabolite OH-MiNP (7-OH-(Mono-methyl-octyl) phthalate) in microg/L

Age	Matrix	Country	Data collection	Sampling year	Sex	N	LOD	LOQ	% below limit	P50	P50_95% CI	P75	P75_95% CI	P90	P90_95% CI	P95	P95_95% CI	GM	GM_95% CI
3-5	mU	DE	GerES IV 2005	2005	F,M	50		0.25	0.00	15.9	11.4 - 23.6	28.1	23.5 - 42.3	44.9	34.5 - 83.4	51.3		15.2	11.6 - 19.8
3-5	mU	DE	GerES V	2015 - 2017	F,M	384		0.20	0.00	8.55	7.63 - 9.20	15.5	13.4 - 17.3	24.5	21.9 - 27.2	33.1	27.2 - 40.0	8.44	7.75 - 9.20
3-5	sU	SK	Children cohort	2015 - 2016	F,M	67		0.20	0.00	7.76	4.65 - 11.6	13.7	12.3 - 15.1	17.1	14.7 - 35.9	24.8		6.29	4.93 - 8.03
6-11	mU	DE	GerES IV 2005	2005	F,M	102		0.25	0.00	14.4	12.2 - 16.9	26.1	19.3 - 30.3	44.2	31.5 - 62.0	61.1	46.4 - 128	14.1	11.8 - 16.7
6-11	sU	DK	CPH-MC	2006 - 2007	F,M	201	0.31		2.49	6.22	5.00 - 6.97	9.89	8.15 - 11.0	16.0	12.4 - 19.1	21.1	17.2 - 39.4	5.02	4.31 - 5.86
6-11	mU	DK	CPHPUB-Cross	2006 - 2008	F,M	913	0.26		0.11	6.58	6.17 - 6.99	11.0	10.2 - 11.7	18.4	16.6 - 20.2	26.2	22.8 - 32.9	6.46	6.11 - 6.83
6-11	mU	SE	DEMOCOPHES-SE	2011 - 2012	F,M	98		0.30	0.00	8.98	7.94 - 11.4	14.8	12.7 - 20.0	32.4	20.1 - 59.9	52.1	35.4 - 493	9.87	8.01 - 12.2
6-11	mU	DK	DEMOCOPHES-DK	2011	F,M	143		0.26	0.00	4.99	4.19 - 5.60	9.55	7.48 - 12.6	19.3	15.7 - 36.3	37.0	21.7 - 94.3	5.49	4.61 - 6.55
6-11	mU	LU	DEMOCOPHES-LU	2011 - 2012	F,M	59		0.20	0.00	7.48	5.57 - 9.01	12.4	9.07 - 17.9	22.6	16.3 - 54.0	26.4		7.18	5.56 - 9.27
6-11	mU	DE	DEMOCOPHES-DE	2011	F,M	116		0.20	0.00	12.5	10.8 - 14.6	20.9	16.1 - 29.5	35.2	30.4 - 52.8	52.4	40.6 - 594	13.9	11.6 - 16.5
6-11	mU	BE	DEMOCOPHES-BE	2011 - 2012	F,M	117		0.10	0.00	6.69	5.82 - 8.31	12.2	10.4 - 17.5	25.7	18.3 - 29.6	29.3	27.0 - 53.9	7.35	6.27 - 8.62
6-11	mU	PL	DEMOCOPHES-PL	2011 - 2012	F,M	94	0.05	0.15	61.7			0.50	0.34 - 0.93	1.47	0.94 - 3.85	2.14	1.49 - 18.5		
6-11	mU	CZ	DEMOCOPHES-CZ	2011 - 2012	F,M	118	0.30	0.70	0.00	12.3	9.70 - 14.8	22.8	18.8 - 27.5	42.6	28.8 - 57.6	53.5	45.5 - 98.0	13.3	11.5 - 15.4
6-11	mU	NO	IES	2012	F,M	50	0.10	0.25	0.00	4.25	3.86 - 6.82	7.97	6.77 - 12.4	13.5	9.88 - 27.0	17.3		5.21	4.23 - 6.42
6-11	mU	DE	GerES V	2015 - 2017	F,M	936		0.20	0.11	7.50	7.16 - 8.00	12.0	11.3 - 12.7	19.4	18.3 - 21.9	27.1	23.8 - 32.6	7.55	7.18 - 7.95
6-11	sU	SK	Children cohort	2015 - 2016	F,M	112		0.20	0.00	7.49	6.53 - 9.02	12.6	10.6 - 15.5	21.4	15.8 - 27.1	26.6	21.7 - 65.8	6.94	5.84 - 8.26
12-19	mU	DE	GerES IV 2005	2005	F,M	51		0.25	0.00	9.53	7.10 - 12.5	16.6	12.4 - 26.2	33.7	22.0 - 46.4	45.1		9.88	7.73 - 12.6
12-19	mU	DK	CPHPUB-Cross	2006 - 2008	F,M	389	0.26		0.51	4.58	4.16 - 5.06	7.92	7.20 - 9.05	14.7	12.4 - 18.1	20.4	17.9 - 30.6	4.53	4.12 - 4.98
12-19	sU	DK	DYMS 2007	2007	M	239	0.26	0.26	2.51	4.12	3.62 - 4.61	7.37	6.52 - 8.53	13.0	10.2 - 17.7	18.9	15.3 - 32.1	3.81	3.30 - 4.39
12-19	sU	DK	DYMS 2008	2008	M	261	0.26	0.26	2.30	4.46	3.90 - 5.39	8.70	7.64 - 10.1	15.2	12.7 - 18.0	21.7	17.5 - 37.7	4.23	3.69 - 4.85
12-19	sU	DK	DYMS 2009	2009	M	210	0.03	0.03	2.38	4.48	3.81 - 5.37	8.12	7.19 - 10.3	14.6	12.6 - 19.0	20.8	15.9 - 29.6	3.82	3.23 - 4.52
12-19	sU	DK	DYMS 2013	2013	M	70	0.03	0.03	0.00	3.02	2.56 - 4.10	6.12	4.54 - 10.0	14.8	9.42 - 33.5	27.4		3.14	2.33 - 4.23
12-19	mU	DE	GerES V	2015 - 2017	F,M	931		0.20	0.11	5.60	5.30 - 5.90	8.65	8.00 - 9.04	13.7	12.6 - 15.0	19.6	16.9 - 24.1	5.56	5.27 - 5.87
12-19	sU	DK	DYMS 2017	2017	M	76	0.03	0.03	0.00	2.42	1.87 - 3.02	4.25	3.08 - 5.91	6.57	5.61 - 24.2	12.3	6.58 - 180	2.63	2.06 - 3.36
20-39	24hU	DE	ESB 2007	2007	F,M	60		0.20	0.00	2.15	1.50 - 2.90	4.25	3.14 - 6.31	7.21	4.96 - 15.5	10.4		2.22	1.73 - 2.84
20-39	sU	DK	DYMS 2008	2008	M	54	0.26	0.26	0.00	5.75	4.73 - 7.37	9.19	7.37 - 11.7	13.2	10.3 - 20.9	14.5		5.18	3.99 - 6.73
20-39	24hU	DE	ESB 2008	2008	F,M	54		0.50	1.85	3.63	2.76 - 4.05	5.27	4.07 - 10.7	12.2	7.57 - 28.1	17.6		3.63	2.84 - 4.62
20-39	sU	DK	DYMS 2009	2009	M	79	0.03	0.03	0.00	5.08	4.29 - 6.47	9.56	7.08 - 12.2	13.8	10.8 - 38.5	23.6	14.1 - 229	5.08	3.99 - 6.45
20-39	24hU	DE	ESB 2009	2009	F,M	60		0.20	1.67	2.40	1.71 - 4.28	5.85	4.30 - 8.94	11.4	7.90 - 18.6	12.2		2.81	2.16 - 3.65
20-39	mU	SE	DEMOCOPHES-SE	2011 - 2012	F	50		0.30	0.00	7.88	5.85 - 12.1	25.0	11.6 - 36.5	75.5	30.5 - 701	154		10.4	6.78 - 16.0
20-39	mU	DK	DEMOCOPHES-DK	2011	F	51		0.26	1.96	3.48	2.08 - 5.57	8.76	5.31 - 17.1	19.1	13.4 - 37.4	31.1		3.47	2.42 - 4.97
20-39	mU	DE	DEMOCOPHES-DE	2011	F	64		0.20	0.00	8.63	6.97 - 12.0	17.5	12.4 - 23.9	26.3	22.1 - 67.7	58.2		9.86	7.79 - 12.5
20-39	24hU	DE	ESB 2011	2011	F,M	60		0.20	0.00	2.85	2.30 - 3.40	4.05	3.48 - 7.30	10.1	6.29 - 70.8	23.2		3.32	2.54 - 4.33
20-39	sU	IL	IBS	2011	F,M	144	0.25	0.50	0.69	15.1	12.2 - 17.4	29.5	22.7 - 40.2	55.1	48.4 - 71.8	72.1	59.7 - 186	14.5	12.0 - 17.4

Age	Matrix	Country	Data collection	Sampling year	Sex	N	LOD	LOQ	% below limit	P50	P50_95% CI	P75	P75_95% CI	P90	P90_95% CI	P95	P95_95% CI	GM	GM_95% CI
20-39	24hU	DE	ESB 2013	2013	F,M	60		0.20	0.00	2.45	1.90 - 2.90	3.73	2.98 - 5.87	6.85	4.75 - 8.81	8.21		2.52	2.08 - 3.05
20-39	24hU	DE	ESB 2015	2015	F,M	60		0.20	0.00	2.40	1.90 - 2.70	4.03	2.86 - 6.49	8.19	5.25 - 17.7	15.3		2.71	2.21 - 3.32
40-59	mU	DK	DEMOCOPHES-DK	2011	F	94		0.26	3.19	2.35	1.81 - 3.03	5.40	3.34 - 6.77	8.28	6.79 - 16.7	14.5	8.93 - 25.4	2.33	1.86 - 2.92
40-59	mU	DE	DEMOCOPHES-DE	2011	F	53		0.20	0.00	5.69	4.65 - 7.63	10.6	7.62 - 17.8	21.4	14.1 - 39.6	32.6		6.31	4.99 - 7.96
40-59	sU	IL	IBS	2011	F,M	86	0.25	0.50	3.49	13.7	9.61 - 15.4	20.1	16.1 - 25.9	35.2	24.6 - 67.8	62.3	36.4 - 294	11.6	9.14 - 14.7

Notes. Matrix: mU - first morning urine, sU - spot urine, 24hU - 24-hours urine; country: AT - Austria, BE - Belgium, CZ - Czech Republic, DE - Germany, DK - Denmark, ES - Spain, HU - Hungary, IL - Israel, LU - Luxemburg, NO - Norway, PL - Poland, SE - Sweden, SI - Slovenia, SK - Slovakia; sex: F - female, M - male; n - sample size; LOD - limit of detection; LOQ - limit of quantification; % below limit - percentage of values below limit of detection/quantification, whichever was applied in the data collection; P50, P75, P90, P95 - 50th, 75th, 90th, 95th percentile; 95% CI - 95 % confidence interval; GM - geometric mean; NA - Not available.

Data only displayed if n was at least 50; percentiles missing when below LOD/LOQ; CIs of the observed percentiles missing when they fall outside the observed range; GM was imputed (fixed imputation) based on a log-normal distribution when at least 50 % of values were above LOD/LOQ.

Table S15: DiNP metabolite oxo-MiNP (7-Oxo-(Mono-methyl-octyl) phthalate) in microg/L

Age	Matrix	Country	Data collection	Sampling year	Sex	N	LOD	LOQ	% below limit	P50	P50_95% CI	P75	P75_95% CI	P90	P90_95% CI	P95	P95_95% CI	GM	GM_95% CI
3-5	mU	DE	GerES IV 2005	2005	F,M	50		0.25	0.00	7.85	5.56 - 10.5	14.8	10.2 - 19.3	29.4	16.0 - 36.7	31.2		7.33	5.58 - 9.64
3-5	mU	DE	GerES V	2015 - 2017	F,M	388		0.20	0.00	3.10	2.90 - 3.40	5.90	5.26 - 6.49	10.2	8.50 - 11.9	13.4	11.8 - 19.9	3.26	2.98 - 3.57
3-5	sU	SK	Children cohort	2015 - 2016	F,M	67		0.20	0.00	2.45	1.69 - 2.77	4.55	3.22 - 6.29	7.49	5.83 - 11.5	10.5		2.13	1.66 - 2.71
6-11	mU	DE	GerES IV 2005	2005	F,M	102		0.25	0.00	6.96	5.79 - 8.78	13.2	10.6 - 16.3	24.4	17.3 - 37.7	35.2	24.6 - 56.2	7.18	6.02 - 8.55
6-11	sU	DK	CPH-MC	2006 - 2007	F,M	201	0.16		1.49	3.13	2.60 - 3.51	4.78	4.10 - 5.46	7.41	6.24 - 8.72	8.95	8.29 - 20.3	2.62	2.27 - 3.02
6-11	mU	DK	CPHPUB-Cross	2006 - 2008	F,M	913	0.25		0.33	3.83	3.55 - 4.17	6.42	5.98 - 6.92	11.2	10.4 - 12.8	15.8	14.0 - 19.7	3.76	3.55 - 3.98
6-11	mU	SE	DEMOCOPHES-SE	2011 - 2012	F,M	98		0.30	0.00	5.25	4.32 - 6.66	9.82	8.04 - 12.1	17.1	12.2 - 41.8	40.8	17.3 - 192	5.71	4.68 - 6.98
6-11	mU	DK	DEMOCOPHES-DK	2011	F,M	143		0.25	1.40	2.58	1.97 - 3.16	5.08	4.00 - 5.95	12.3	8.09 - 16.6	16.7	13.8 - 39.0	2.64	2.18 - 3.20
6-11	mU	DE	DEMOCOPHES-DE	2011	F,M	119		0.20	0.00	4.44	3.70 - 5.13	8.65	6.50 - 11.2	15.8	12.4 - 22.4	20.9	16.5 - 416	4.91	4.02 - 6.02
6-11	mU	NO	IES	2012	F,M	50	0.10	0.25	0.00	2.44	1.91 - 3.16	4.64	3.09 - 6.10	7.36	5.64 - 12.6	10.4		2.64	2.13 - 3.26
6-11	mU	DE	GerES V	2015 - 2017	F,M	937		0.20	0.21	2.90	2.70 - 3.00	4.80	4.40 - 5.10	8.50	7.74 - 9.54	12.3	10.5 - 14.5	2.94	2.78 - 3.11
6-11	sU	SK	Children cohort	2015 - 2016	F,M	112		0.20	0.00	2.77	2.43 - 3.70	4.42	3.97 - 5.88	7.42	6.11 - 11.9	10.9	8.55 - 53.1	2.60	2.15 - 3.15
12-19	mU	DE	GerES IV 2005	2005	F,M	51		0.25	0.00	4.86	3.53 - 6.67	8.11	6.66 - 13.3	17.3	9.95 - 25.7	20.9		4.93	3.87 - 6.28
12-19	mU	DK	CPHPUB-Cross	2006 - 2008	F,M	389	0.25		1.29	2.62	2.34 - 2.93	4.46	4.00 - 5.27	7.87	6.97 - 10.2	12.9	10.0 - 17.4	2.55	2.32 - 2.81
12-19	sU	DK	DYMS 2007	2007	M	239	0.25	0.25	4.60	1.94	1.71 - 2.31	3.70	3.19 - 4.02	6.37	5.29 - 9.87	11.1	8.81 - 14.5	1.86	1.61 - 2.14
12-19	sU	DK	DYMS 2008	2008	M	261	0.25	0.25	4.21	2.39	2.02 - 2.73	5.15	4.06 - 5.95	8.15	6.86 - 10.4	13.0	9.16 - 18.7	2.23	1.95 - 2.56
12-19	sU	DK	DYMS 2009	2009	M	210	0.02	0.02	6.67	2.28	1.99 - 2.65	4.40	3.78 - 5.47	7.44	6.59 - 9.30	9.67	8.66 - 16.4	1.93	1.62 - 2.29
12-19	sU	DK	DYMS 2013	2013	M	70	0.02	0.02	1.43	1.56	1.19 - 2.32	3.16	2.47 - 5.31	9.92	4.98 - 21.2	17.9		1.53	1.09 - 2.15
12-19	mU	DE	GerES V	2015 - 2017	F,M	932		0.20	0.54	2.20	2.10 - 2.40	3.83	3.60 - 4.10	6.40	5.80 - 7.17	9.04	8.10 - 12.3	2.31	2.18 - 2.45
12-19	sU	DK	DYMS 2017	2017	M	76	0.02	0.02	0.00	1.16	0.93 - 1.44	2.45	1.71 - 3.05	3.90	3.02 - 14.9	7.37	3.92 - 87.3	1.27	0.97 - 1.66
20-39	24hU	DE	ESB 2007	2007	F,M	60		0.20	5.00	0.95	0.80 - 1.30	1.80	1.46 - 2.67	3.10	2.16 - 8.16	4.43		1.07	0.85 - 1.34
20-39	sU	DK	DYMS 2008	2008	M	54	0.25	0.25	3.70	3.31	2.33 - 3.67	4.27	3.68 - 6.90	7.17	5.73 - 9.72	8.91		2.66	2.06 - 3.45
20-39	24hU	DE	ESB 2008	2008	F,M	54		0.50	9.26	2.20	1.98 - 2.84	3.39	2.84 - 5.89	8.27	5.17 - 21.4	12.6		2.44	1.89 - 3.15
20-39	sU	DK	DYMS 2009	2009	M	79	0.02	0.02	1.27	2.47	1.80 - 2.99	4.71	3.44 - 6.10	8.30	6.03 - 15.9	12.4	8.36 - 148	2.38	1.85 - 3.06
20-39	24hU	DE	ESB 2009	2009	F,M	60		0.20	5.00	1.15	0.71 - 1.90	3.33	2.14 - 4.60	5.44	4.36 - 8.56	7.91		1.22	0.90 - 1.65
20-39	mU	SE	DEMOCOPHES-SE	2011 - 2012	F	50		0.30	0.00	4.51	3.35 - 6.67	9.83	6.45 - 24.1	55.4	16.2 - 510	63.8		5.84	3.85 - 8.88
20-39	mU	DK	DEMOCOPHES-DK	2011	F	51		0.25	5.88	2.06	1.45 - 2.49	5.61	2.45 - 12.1	13.7	8.07 - 21.7	18.4		1.91	1.30 - 2.79
20-39	mU	DE	DEMOCOPHES-DE	2011	F	64		0.20	0.00	2.90	2.01 - 3.68	5.42	3.95 - 8.09	11.6	7.78 - 36.1	27.6		3.25	2.46 - 4.29
20-39	24hU	DE	ESB 2011	2011	F,M	60		0.20	0.00	1.65	1.20 - 2.00	2.62	2.08 - 4.64	5.50	3.95 - 39.0	14.3		1.85	1.39 - 2.46
20-39	sU	IL	IBS	2011	F,M	144	0.25	0.50	1.39	7.75	6.51 - 9.66	16.9	13.7 - 21.7	30.1	23.0 - 36.5	36.5	31.7 - 109	7.83	6.52 - 9.40
20-39	24hU	DE	ESB 2013	2013	F,M	60		0.20	5.00	1.10	0.90 - 1.40	1.62	1.40 - 2.49	2.71	2.06 - 7.76	3.96		1.09	0.87 - 1.38
20-39	24hU	DE	ESB 2015	2015	F,M	60		0.20	0.00	0.85	0.70 - 1.50	1.90	1.50 - 2.97	3.48	2.29 - 6.42	4.71		1.08	0.86 - 1.36
40-59	mU	DK	DEMOCOPHES-DK	2011	F	94		0.25	13.8	1.12	0.86 - 1.57	2.33	2.03 - 3.50	5.23	3.54 - 8.10	7.66	5.46 - 14.3	1.10	0.86 - 1.40
40-59	mU	DE	DEMOCOPHES-DE	2011	F	56		0.20	0.00	1.96	1.40 - 2.90	3.97	2.90 - 7.63	8.19	5.61 - 16.0	10.4		2.10	1.61 - 2.74

Age	Matrix	Country	Data collection	Sampling year	Sex	N	LOD	LOQ	% below limit	P50	P50_95% CI	P75	P75_95% CI	P90	P90_95% CI	P95	P95_95% CI	GM	GM_95% CI
40-59	sU	IL	IBS	2011	F,M	86	0.25	0.50	3.49	6.14	4.46 - 8.56	11.7	9.82 - 15.6	23.5	15.6 - 39.7	31.2	23.6 - 114	6.19	4.93 - 7.77

Notes. Matrix: mU - first morning urine, sU - spot urine, 24hU - 24-hours urine; country: AT - Austria, BE - Belgium, CZ - Czech Republic, DE - Germany, DK - Denmark, ES - Spain, HU - Hungary, IL - Israel, LU - Luxemburg, NO - Norway, PL - Poland, SE - Sweden, SI - Slovenia, SK - Slovakia; sex: F - female, M - male; n - sample size; LOD - limit of detection; LOQ - limit of quantification; % below limit - percentage of values below limit of detection/quantification, whichever was applied in the data collection; P50, P75, P90, P95 - 50th, 75th, 90th, 95th percentile; 95% CI - 95 % confidence interval; GM - geometric mean; NA - Not available.

Data only displayed if n was at least 50; percentiles missing when below LOD/LOQ; CIs of the observed percentiles missing when they fall outside the observed range; GM was imputed (fixed imputation) based on a log-normal distribution when at least 50 % of values were above LOD/LOQ.

Table S16: DiNP metabolite MiNP (Mono-methyl-octyl phthalate) in microg/L

Age	Matrix	Country	Data collection	Sampling year	Sex	N	LOD	LOQ	% below limit	P50	P50_95% CI	P75	P75_95% CI	P90	P90_95% CI	P95	P95_95% CI	GM	GM_95% CI
3-5	sU	DK	CPH-MC	2006	F,M	189	0.62		49.7			2.13	1.57 - 2.87	4.33	3.51 - 5.81	6.26	5.34 - 8.41		
6-11	sU	DK	CPH-MC	2006 - 2007	F,M	659	0.62		51.4			1.67	1.44 - 1.84	3.06	2.77 - 3.54	4.41	3.99 - 5.03		
6-11	mU	DK	CPHPUB-Cross	2006 - 2008	F,M	913	0.61		44.6	0.71	0.66 - 0.78	1.53	1.33 - 1.71	2.96	2.63 - 3.36	4.41	3.90 - 5.27	0.72	0.67 - 0.77
6-11	mU	DK	DEMOCOPHES-DK	2011	F,M	143	0.61		83.9					1.21	0.67 - 3.14	3.17	2.01 - 9.24		
12-19	mU	DK	CPHPUB-Cross	2006 - 2008	F,M	389	0.61		56.0			1.03	0.90 - 1.28	2.26	1.85 - 2.77	3.46	2.73 - 6.04		
20-39	mU	DK	DEMOCOPHES-DK	2011	F	51	0.61		76.5					1.93	1.07 - 5.41	3.27			
40-59	mU	DK	DEMOCOPHES-DK	2011	F	94	0.61		89.4							1.12	0.81 - 2.93		

Notes. Matrix: mU - first morning urine, sU - spot urine, 24hU - 24-hours urine; country: AT - Austria, BE - Belgium, CZ - Czech Republic, DE - Germany, DK - Denmark, ES - Spain, HU - Hungary, IL - Israel, LU - Luxemburg, NO - Norway, PL - Poland, SE - Sweden, SI - Slovenia, SK - Slovakia; sex: F - female, M - male; n - sample size; LOD - limit of detection; LOQ - limit of quantification; % below limit - percentage of values below limit of detection/quantification, whichever was applied in the data collection; P50, P75, P90, P95 - 50th, 75th, 90th, 95th percentile; 95% CI - 95 % confidence interval; GM - geometric mean; NA - Not available.

Data only displayed if n was at least 50; percentiles missing when below LOD/LOQ; CIs of the observed percentiles missing when they fall outside the observed range; GM was imputed (fixed imputation) based on a log-normal distribution when at least 50 % of values were above LOD/LOQ.

Table S17: DiDP metabolite cx-MiDP (Mono(2,7-methyl-7- carboxy-heptyl) phthalate) in microg/L

Age	Matrix	Country	Data collection	Sampling year	Sex	N	LOD	LOQ	% below limit	P50	P50_95% CI	P75	P75_95% CI	P90	P90_95% CI	P95	P95_95% CI	GM	GM_95% CI
3-5	mU	DE	GerES V	2015 - 2017	F,M	387		0.20	0.78	1.10	1.00 - 1.20	1.90	1.78 - 2.20	2.90	2.60 - 3.50	3.80	3.50 - 4.80	1.12	1.03 - 1.21
6-11	mU	LU	DEMOCOPHES-LU	2011 - 2012	F,M	59		0.20	0.00	1.40	1.20 - 1.78	2.54	1.83 - 3.67	4.49	2.93 - 7.16	6.53		1.48	1.20 - 1.83
6-11	mU	DE	DEMOCOPHES-DE	2011	F,M	119		0.20	2.52	1.09	0.86 - 1.24	1.77	1.43 - 2.19	2.68	2.28 - 4.02	3.51	2.85 - 12.5	1.06	0.91 - 1.23
6-11	mU	BE	DEMOCOPHES-BE	2011 - 2012	F,M	117		0.10	0.00	1.38	1.17 - 1.59	2.15	1.83 - 2.58	3.15	2.75 - 5.11	5.02	3.98 - 10.8	1.43	1.24 - 1.64
6-11	mU	DE	GerES V	2015 - 2017	F,M	937		0.20	1.60	0.90	0.90 - 1.00	1.50	1.40 - 1.60	2.60	2.30 - 3.10	4.30	3.80 - 5.43	0.97	0.92 - 1.02
12-19	sU	DK	DYMS 2009	2009	M	69	0.02	0.02	10.1	0.80	0.66 - 1.01	1.33	1.12 - 1.76	2.54	1.60 - 4.24	3.12		0.54	0.38 - 0.77
12-19	sU	DK	DYMS 2013	2013	M	70	0.02	0.02	5.71	0.71	0.55 - 0.88	1.05	0.89 - 1.57	2.19	1.39 - 3.52	3.10		0.59	0.41 - 0.84
12-19	mU	DE	GerES V	2015 - 2017	F,M	932		0.20	3.97	0.70	0.70 - 0.70	1.20	1.10 - 1.20	1.80	1.70 - 2.10	2.70	2.40 - 3.15	0.73	0.70 - 0.77
12-19	sU	DK	DYMS 2017	2017	M	76	0.02	0.02	6.58	0.35	0.23 - 0.44	0.61	0.48 - 0.79	1.24	0.72 - 2.28	2.21	1.26 - 4.57	0.28	0.21 - 0.38
20-39	24hU	DE	ESB 2007	2007	F,M	60		0.20	0.00	0.50	0.40 - 0.60	0.90	0.60 - 1.40	1.83	1.20 - 3.92	3.21		0.63	0.51 - 0.77
20-39	24hU	DE	ESB 2009	2009	F,M	60		0.20	5.00	0.50	0.40 - 0.60	0.82	0.60 - 0.97	1.21	0.90 - 4.13	1.52		0.52	0.42 - 0.64
20-39	mU	DE	DEMOCOPHES-DE	2011	F	64		0.20	6.25	0.71	0.53 - 0.93	1.18	0.95 - 1.59	1.99	1.46 - 2.69	2.48		0.69	0.57 - 0.83
20-39	24hU	DE	ESB 2011	2011	F,M	60		0.20	13.3	0.60	0.50 - 0.60	0.80	0.60 - 1.00	1.61	1.00 - 3.06	2.71		0.56	0.44 - 0.70
20-39	24hU	DE	ESB 2013	2013	F,M	60		0.20	23.3	0.40	0.30 - 0.50	0.60	0.50 - 0.87	1.00	0.80 - 2.05	1.11		0.37	0.30 - 0.46
20-39	24hU	DE	ESB 2015	2015	F,M	60		0.20	18.3	0.40	0.30 - 0.40	0.52	0.40 - 0.80	1.01	0.76 - 2.05	1.51		0.37	0.30 - 0.46
40-59	mU	DE	DEMOCOPHES-DE	2011	F	56		0.20	19.6	0.50	0.39 - 0.78	1.23	0.80 - 1.53	1.75	1.48 - 2.93	2.45		0.52	0.40 - 0.68

Notes. Matrix: mU - first morning urine, sU - spot urine, 24hU - 24-hours urine; country: AT - Austria, BE - Belgium, CZ - Czech Republic, DE - Germany, DK - Denmark, ES - Spain, HU - Hungary, IL - Israel, LU - Luxemburg, NO - Norway, PL - Poland, SE - Sweden, SI - Slovenia, SK - Slovakia; sex: F - female, M - male; n - sample size; LOD - limit of detection; LOQ - limit of quantification; % below limit - percentage of values below limit of detection/quantification, whichever was applied in the data collection; P50, P75, P90, P95 - 50th, 75th, 90th, 95th percentile; 95% CI - 95 % confidence interval; GM - geometric mean; NA - Not available.

Data only displayed if n was at least 50; percentiles missing when below LOD/LOQ; CIs of the observed percentiles missing when they fall outside the observed range; GM was imputed (fixed imputation) based on a log-normal distribution when at least 50 % of values were above LOD/LOQ.

Table S18: DiDP metabolite OH-MiDP (6-OH-Mono-propyl-heptyl phthalate) in microg/L

Age	Matrix	Country	Data collection	Sampling year	Sex	N	LOD	LOQ	% below limit	P50	P50_95% CI	P75	P75_95% CI	P90	P90_95% CI	P95	P95_95% CI	GM	GM_95% CI
3-5	mU	DE	GerES V	2015 - 2017	F,M	388		0.20	0.77	1.70	1.60 - 2.00	3.42	3.00 - 3.80	5.43	4.80 - 6.89	8.43	6.83 - 11.1	1.81	1.65 - 1.98
3-5	sU	SK	Children cohort	2015 - 2016	F,M	67		0.20	0.00	1.42		2.97		4.40		6.14			
6-11	mU	LU	DEMOCOPHES-LU	2011 - 2012	F,M	59		0.20	1.69	2.02	1.61 - 2.87	4.09	3.18 - 5.38	6.84	5.05 - 19.1	9.89		2.14	1.64 - 2.79
6-11	mU	DE	DEMOCOPHES-DE	2011	F,M	119		0.20	0.00	2.30	2.00 - 2.69	3.79	3.15 - 5.05	5.70	5.13 - 11.3	10.6	5.95 - 17.7	2.34	2.00 - 2.73
6-11	mU	BE	DEMOCOPHES-BE	2011 - 2012	F,M	117		0.10	19.7	0.61	0.52 - 0.77	1.54	1.27 - 2.22	3.67	2.47 - 5.57	5.44	3.80 - 11.7	0.60	0.46 - 0.78
6-11	mU	CZ	DEMOCOPHES-CZ	2011 - 2012	F,M	118	0.20	0.60	91.5							0.71			
6-11	mU	DE	GerES V	2015 - 2017	F,M	937		0.20	0.85	1.60	1.50 - 1.70	2.80	2.60 - 3.00	4.90	4.34 - 5.80	8.06	6.71 - 10.1	1.65	1.56 - 1.75
6-11	sU	SK	Children cohort	2015 - 2016	F,M	112		0.20	0.00	1.54	1.25 - 2.01	2.84	2.29 - 3.26	4.13	3.33 - 6.64	6.39	4.15 - 7.36	1.46	1.23 - 1.73
12-19	sU	DK	DYMS 2009	2009	M	69	0.02	0.02	11.6	0.45	0.32 - 0.97	1.43	1.03 - 1.84	2.07	1.73 - 2.66	2.61		0.35	0.23 - 0.52
12-19	sU	DK	DYMS 2013	2013	M	70	0.02	0.02	7.14	0.50	0.44 - 0.83	1.03	0.89 - 1.60	2.25	1.41 - 8.23	3.57		0.51	0.35 - 0.76
12-19	mU	DE	GerES V	2015 - 2017	F,M	932		0.20	2.90	1.30	1.20 - 1.34	2.20	2.00 - 2.30	3.60	3.30 - 4.00	5.30	4.80 - 6.40	1.27	1.20 - 1.35
12-19	sU	DK	DYMS 2017	2017	M	76	0.02	0.02	2.63	0.40	0.31 - 0.55	0.74	0.56 - 1.15	1.44	0.92 - 6.86	4.24	1.45 - 17.8	0.41	0.30 - 0.55
20-39	24hU	DE	ESB 2007	2007	F,M	60		0.20	3.33	0.70	0.50 - 1.00	1.23	1.00 - 1.77	2.37	1.66 - 5.39	3.94		0.80	0.63 - 1.01
20-39	24hU	DE	ESB 2009	2009	F,M	60		0.20	10.0	0.70	0.50 - 0.90	1.20	0.90 - 1.77	1.92	1.56 - 4.83	2.42		0.69	0.54 - 0.88
20-39	mU	DE	DEMOCOPHES-DE	2011	F	64		0.20	0.00	1.66	1.31 - 1.92	2.62	1.96 - 3.35	4.55	3.01 - 7.14	5.44		1.69	1.41 - 2.02
20-39	24hU	DE	ESB 2011	2011	F,M	60		0.20	5.00	0.95	0.70 - 1.10	1.20	1.10 - 1.80	3.71	1.70 - 13.0	5.88		0.93	0.70 - 1.22
20-39	24hU	DE	ESB 2013	2013	F,M	60		0.20	10.0	0.80	0.60 - 1.00	1.30	1.00 - 1.70	1.92	1.46 - 5.65	3.00		0.76	0.61 - 0.96
20-39	24hU	DE	ESB 2015	2015	F,M	60		0.20	3.33	0.80	0.50 - 0.90	1.20	0.90 - 1.47	1.60	1.40 - 3.95	3.41		0.70	0.57 - 0.86
40-59	mU	DE	DEMOCOPHES-DE	2011	F	56		0.20	0.00	1.08	0.75 - 1.71	2.13	1.71 - 3.35	3.66	2.63 - 4.88	4.28		1.10	0.87 - 1.39

Notes. Matrix: mU - first morning urine, sU - spot urine, 24hU - 24-hours urine; country: AT - Austria, BE - Belgium, CZ - Czech Republic, DE - Germany, DK - Denmark, ES - Spain, HU - Hungary, IL - Israel, LU - Luxemburg, NO - Norway, PL - Poland, SE - Sweden, SI - Slovenia, SK - Slovakia; sex: F - female, M - male; n - sample size; LOD - limit of detection; LOQ - limit of quantification; % below limit - percentage of values below limit of detection/quantification, whichever was applied in the data collection; P50, P75, P90, P95 - 50th, 75th, 90th, 95th percentile; 95% CI - 95 % confidence interval; GM - geometric mean; NA - Not available.

Data only displayed if n was at least 50; percentiles missing when below LOD/LOQ; CIs of the observed percentiles missing when they fall outside the observed range; GM was imputed (fixed imputation) based on a log-normal distribution when at least 50 % of values were above LOD/LOQ.

Table S19: DiDP metabolite oxo-MiDP (6-Oxo-Mono-propyl-heptyl phthalate) in microg/L

Age	Matrix	Country	Data collection	Sampling year	Sex	N	LOD	LOQ	% below limit	P50	P50_95% CI	P75	P75_95% CI	P90	P90_95% CI	P95	P95_95% CI	GM	GM_95% CI
3-5	mU	DE	GerES V	2015 - 2017	F,M	388		0.20	10.1	0.70	0.60 - 0.80	1.32	1.16 - 1.60	2.23	2.00 - 2.89	3.63	2.83 - 6.12	0.71	0.64 - 0.78
3-5	sU	SK	Children cohort	2015 - 2016	F,M	67		0.20	0.00	0.60		0.93		1.62		2.54			
6-11	mU	DE	DEMOCOPHES-DE	2011	F,M	119		0.20	19.3	0.45	0.40 - 0.61	1.01	0.79 - 1.29	1.79	1.36 - 3.18	2.98	1.95 - 5.87	0.51	0.42 - 0.61
6-11	mU	DE	GerES V	2015 - 2017	F,M	937		0.20	9.39	0.60	0.60 - 0.70	1.20	1.10 - 1.30	2.20	2.00 - 2.50	3.40	2.91 - 4.15	0.68	0.64 - 0.72
6-11	sU	SK	Children cohort	2015 - 2016	F,M	112		0.20	0.00	0.48		0.85		1.31		2.05			
12-19	sU	DK	DYMS 2009	2009	M	69	0.02	0.02	4.35	0.58	0.48 - 0.72	1.02	0.83 - 1.32	1.55	1.26 - 2.15	2.08		0.44	0.32 - 0.59
12-19	sU	DK	DYMS 2013	2013	M	70	0.02	0.02	5.71	0.75	0.55 - 0.96	1.13	1.03 - 1.33	1.51	1.27 - 3.37	2.79		0.62	0.45 - 0.87
12-19	mU	DE	GerES V	2015 - 2017	F,M	932		0.20	14.9	0.50	0.50 - 0.60	1.00	0.90 - 1.10	1.79	1.60 - 1.90	2.60	2.20 - 3.05	0.54	0.51 - 0.57
12-19	sU	DK	DYMS 2017	2017	M	76	0.02	0.02	0.00	0.95	0.68 - 1.10	1.54	1.15 - 2.01	2.62	1.88 - 8.09	4.47	2.62 - 33.5	0.93	0.75 - 1.17
20-39	24hU	DE	ESB 2007	2007	F,M	60		0.20	33.3	0.30	0.20 - 0.40	0.50	0.40 - 0.87	1.04	0.76 - 2.43	1.73		0.30	0.23 - 0.39
20-39	24hU	DE	ESB 2009	2009	F,M	60		0.20	35.0	0.30	0.20 - 0.40	0.40	0.40 - 0.60	0.60	0.50 - 1.46	1.11		0.28	0.23 - 0.34
20-39	mU	DE	DEMOCOPHES-DE	2011	F	64		0.20	28.1	0.36	0.24 - 0.53	0.72	0.55 - 0.88	1.08	0.83 - 4.88	1.56		0.38	0.30 - 0.49
20-39	24hU	DE	ESB 2011	2011	F,M	60		0.20	46.7	0.20		0.50	0.40 - 0.60	0.81	0.60 - 3.61	1.49		0.22	0.17 - 0.30
20-39	24hU	DE	ESB 2013	2013	F,M	60		0.20	43.3	0.20		0.42	0.30 - 0.70	0.80	0.60 - 2.30	1.64		0.21	0.16 - 0.28
20-39	24hU	DE	ESB 2015	2015	F,M	60		0.20	43.3	0.30		0.50	0.40 - 0.60	0.70	0.60 - 0.92	0.80		0.28	0.24 - 0.34
40-59	mU	DE	DEMOCOPHES-DE	2011	F	56		0.20	48.2	0.20		0.63	0.44 - 0.91	0.97	0.77 - 2.10	1.66		0.23	0.17 - 0.31

Notes. Matrix: mU - first morning urine, sU - spot urine, 24hU - 24-hours urine; country: AT - Austria, BE - Belgium, CZ - Czech Republic, DE - Germany, DK - Denmark, ES - Spain, HU - Hungary, IL - Israel, LU - Luxemburg, NO - Norway, PL - Poland, SE - Sweden, SI - Slovenia, SK - Slovakia; sex: F - female, M - male; n - sample size; LOD - limit of detection; LOQ - limit of quantification; % below limit - percentage of values below limit of detection/quantification, whichever was applied in the data collection; P50, P75, P90, P95 - 50th, 75th, 90th, 95th percentile; 95% CI - 95 % confidence interval; GM - geometric mean; NA - Not available.

Data only displayed if n was at least 50; percentiles missing when below LOD/LOQ; CIs of the observed percentiles missing when they fall outside the observed range; GM was imputed (fixed imputation) based on a log-normal distribution when at least 50 % of values were above LOD/LOQ.

Table S20: DINCH metabolite cx-MINCH (cyclohexane-1,2- dicarboxylate-mono-(7- carboxylate-4- methyl)heptyl ester) in microg/L

Age	Matrix	Country	Data collection	Sampling year	Sex	N	LOD	LOQ	% below limit	P50	P50_95% CI	P75	P75_95% CI	P90	P90_95% CI	P95	P95_95% CI	GM	GM_95% CI
3-5	mU	DE	GerES V	2015 - 2017	F,M	378		0.05	0.53	2.06	1.82 - 2.25	4.05	3.41 - 4.49	7.54	6.78 - 9.24	11.1	9.11 - 14.9	2.16	1.95 - 2.40
6-11	mU	DE	GerES V	2015 - 2017	F,M	927		0.05	0.76	1.14	1.07 - 1.23	2.23	2.10 - 2.38	4.37	3.80 - 5.04	7.50	5.99 - 9.24	1.23	1.15 - 1.31
12-19	sU	DK	DYMS 2009	2009	M	69	0.02	0.02	23.2	0.36	0.13 - 0.80	2.31	1.20 - 4.55	8.28	4.22 - 29.4	13.6		0.28	0.15 - 0.55
12-19	sU	DK	DYMS 2013	2013	M	70	0.02	0.02	5.71	0.54	0.35 - 0.81	1.38	0.91 - 3.28	5.80	2.40 - 13.5	8.44		0.51	0.33 - 0.77
12-19	mU	DE	GerES V	2015 - 2017	F,M	919		0.05	1.20	0.81	0.75 - 0.87	1.59	1.45 - 1.80	3.19	2.84 - 3.72	4.64	4.26 - 5.89	0.83	0.77 - 0.89
12-19	sU	DK	DYMS 2017	2017	M	76	0.02	0.02	3.95	0.49	0.31 - 0.80	1.80	0.91 - 5.62	8.26	3.90 - 14.1	13.4	8.27 - 70.8	0.63	0.42 - 0.94
20-39	24hU	DE	ESB 2009	2009	F,M	60		0.05	71.7			0.06		0.15	0.11 - 1.45	0.88			
20-39	24hU	DE	ESB 2010	2010	F,M	60		0.05	71.7			0.11		0.38	0.22 - 1.47	1.11			
20-39	24hU	DE	ESB 2011	2011	F,M	60		0.05	41.7	0.09		0.34	0.19 - 0.60	1.16	0.45 - 8.62	3.36		0.08	0.05 - 0.14
20-39	24hU	DE	ESB 2012	2012	F,M	56		0.05	12.5	0.17	0.12 - 0.26	0.34	0.27 - 0.58	0.64	0.48 - 2.00	0.85		0.19	0.13 - 0.26
20-39	24hU	DE	ESB 2013	2013	F,M	60		0.05	21.7	0.13	0.10 - 0.19	0.29	0.19 - 0.36	0.54	0.34 - 0.92	0.87		0.13	0.10 - 0.18
20-39	24hU	DE	ESB 2015	2015	F,M	60		0.05	20.0	0.21	0.17 - 0.26	0.35	0.26 - 0.42	0.60	0.41 - 3.60	1.33		0.18	0.13 - 0.25
20-39	24hU	DE	ESB 2017	2017	F,M	60		0.05	13.3	0.20	0.16 - 0.27	0.38	0.29 - 0.61	0.86	0.47 - 2.82	1.02		0.21	0.16 - 0.28

Notes. Matrix: mU - first morning urine, sU - spot urine, 24hU - 24-hours urine; country: AT - Austria, BE - Belgium, CZ - Czech Republic, DE - Germany, DK - Denmark, ES - Spain, HU - Hungary, IL - Israel, LU - Luxemburg, NO - Norway, PL - Poland, SE - Sweden, SI - Slovenia, SK - Slovakia; sex: F - female, M - male; n - sample size; LOD - limit of detection; LOQ - limit of quantification; % below limit - percentage of values below limit of detection/quantification, whichever was applied in the data collection; P50, P75, P90, P95 - 50th, 75th, 90th, 95th percentile; 95% CI - 95 % confidence interval; GM - geometric mean; NA - Not available.

Data only displayed if n was at least 50; percentiles missing when below LOD/LOQ; CIs of the observed percentiles missing when they fall outside the observed range; GM was imputed (fixed imputation) based on a log-normal distribution when at least 50 % of values were above LOD/LOQ.

Table S21: DINCH metabolite OH-MINCH (cyclohexane-1,2- dicarboxylate-mono-(7- hydroxy-4-methyl)octyl ester) in microg/L

Age	Matrix	Country	Data collection	Sampling year	Sex	N	LOD	LOQ	% below limit	P50	P50_95% CI	P75	P75_95% CI	P90	P90_95% CI	P95	P95_95% CI	GM	GM_95% CI
3-5	mU	DE	GerES V	2015 - 2017	F,M	378		0.05	0.00	4.14	3.60 - 4.53	7.86	7.09 - 9.85	16.2	12.4 - 19.5	22.6	19.4 - 31.0	4.39	3.97 - 4.86
6-11	mU	DE	GerES V	2015 - 2017	F,M	929		0.05	0.22	2.27	2.08 - 2.46	4.50	4.08 - 4.91	9.42	8.45 - 10.6	15.6	12.6 - 22.5	2.48	2.31 - 2.65
12-19	sU	DK	DYMS 2009	2009	M	69	0.02	0.02	21.7	0.74	0.45 - 1.49	2.76	1.92 - 6.81	13.8	4.20 - 31.7	24.4		0.50	0.26 - 0.95
12-19	sU	DK	DYMS 2013	2013	M	70	0.02	0.02	8.57	1.01	0.70 - 1.70	3.43	1.92 - 7.19	13.3	6.29 - 25.8	23.2		0.93	0.57 - 1.51
12-19	mU	DE	GerES V	2015 - 2017	F,M	921		0.05	0.33	1.51	1.39 - 1.61	3.15	2.76 - 3.53	7.05	6.21 - 8.11	10.7	9.45 - 14.3	1.62	1.50 - 1.74
12-19	sU	DK	DYMS 2017	2017	M	76	0.02	0.02	2.63	1.18	0.91 - 1.85	3.75	1.99 - 6.64	15.2	5.93 - 34.1	28.5	15.4 - 142	1.61	1.12 - 2.31
20-39	24hU	DE	ESB 2009	2009	F,M	60		0.05	56.7			0.13	0.07 - 0.22	0.37	0.18 - 3.44	1.01			
20-39	24hU	DE	ESB 2010	2010	F,M	60		0.05	20.0	0.14	0.10 - 0.24	0.52	0.26 - 1.02	1.11	0.90 - 4.62	3.23		0.17	0.12 - 0.26
20-39	24hU	DE	ESB 2011	2011	F,M	60		0.05	1.67	0.31	0.20 - 0.53	0.93	0.55 - 2.19	4.20	1.20 - 39.4	8.23		0.43	0.28 - 0.64
20-39	24hU	DE	ESB 2012	2012	F,M	56		0.05	1.79	0.39	0.25 - 0.50	0.70	0.52 - 1.32	1.55	1.08 - 4.28	2.04		0.41	0.28 - 0.58
20-39	24hU	DE	ESB 2013	2013	F,M	60		0.05	0.00	0.37	0.31 - 0.43	0.67	0.43 - 1.20	1.38	1.05 - 4.06	2.81		0.45	0.35 - 0.58
20-39	24hU	DE	ESB 2015	2015	F,M	60		0.05	0.00	0.58	0.54 - 0.77	1.10	0.80 - 1.45	1.74	1.25 - 11.1	3.53		0.64	0.48 - 0.84
20-39	24hU	DE	ESB 2017	2017	F,M	60		0.05	0.00	0.70	0.48 - 0.89	1.24	0.91 - 1.85	2.42	1.52 - 8.01	3.96		0.70	0.54 - 0.90

Notes. Matrix: mU - first morning urine, sU - spot urine, 24hU - 24-hours urine; country: AT - Austria, BE - Belgium, CZ - Czech Republic, DE - Germany, DK - Denmark, ES - Spain, HU - Hungary, IL - Israel, LU - Luxemburg, NO - Norway, PL - Poland, SE - Sweden, SI - Slovenia, SK - Slovakia; sex: F - female, M - male; n - sample size; LOD - limit of detection; LOQ - limit of quantification; % below limit - percentage of values below limit of detection/quantification, whichever was applied in the data collection; P50, P75, P90, P95 - 50th, 75th, 90th, 95th percentile; 95% CI - 95 % confidence interval; GM - geometric mean; NA - Not available.

Data only displayed if n was at least 50; percentiles missing when below LOD/LOQ; CIs of the observed percentiles missing when they fall outside the observed range; GM was imputed (fixed imputation) based on a log-normal distribution when at least 50 % of values were above LOD/LOQ.

Table S22: DINCH metabolite oxo-MINCH (cyclohexane-1,2- dicarboxylate-mono-(7-oxo- 4-methyl)octyl ester) in microg/L

Age	Matrix	Country	Data collection	Sampling year	Sex	N	LOD	LOQ	% below limit	P50	P50_95% CI	P75	P75_95% CI	P90	P90_95% CI	P95	P95_95% CI	GM	GM_95% CI
3-5	mU	DE	GerES V	2015 - 2017	F,M	378		0.05	1.06	1.88	1.61 - 2.07	3.56	3.06 - 4.27	7.47	6.51 - 8.65	10.3	8.62 - 14.1	1.82	1.62 - 2.05
6-11	mU	DE	GerES V	2015 - 2017	F,M	930		0.05	1.51	0.94	0.87 - 1.02	2.11	1.86 - 2.40	4.59	4.18 - 5.57	7.70	6.60 - 9.96	1.01	0.93 - 1.09
12-19	mU	DE	GerES V	2015 - 2017	F,M	918		0.05	4.25	0.70	0.63 - 0.77	1.47	1.34 - 1.68	3.60	3.02 - 4.25	6.37	5.14 - 7.59	0.69	0.63 - 0.75
20-39	24hU	DE	ESB 2009	2009	F,M	60		0.05	80.0					0.22	0.11 - 2.83	0.72			
20-39	24hU	DE	ESB 2010	2010	F,M	60		0.05	45.0	0.06		0.19	0.11 - 0.43	0.51	0.37 - 2.09	1.45		0.06	0.04 - 0.10
20-39	24hU	DE	ESB 2011	2011	F,M	60		0.05	20.0	0.17	0.11 - 0.33	0.52	0.33 - 1.43	2.74	0.75 - 20.9	4.98		0.21	0.13 - 0.33
20-39	24hU	DE	ESB 2012	2012	F,M	56		0.05	14.3	0.27	0.20 - 0.34	0.49	0.34 - 1.13	1.22	0.76 - 3.01	1.55		0.27	0.18 - 0.40
20-39	24hU	DE	ESB 2013	2013	F,M	60		0.05	0.00	0.21	0.18 - 0.30	0.38	0.31 - 0.64	0.70	0.57 - 3.34	2.17		0.25	0.19 - 0.33
20-39	24hU	DE	ESB 2015	2015	F,M	60		0.05	3.33	0.28	0.23 - 0.37	0.57	0.40 - 0.84	1.19	0.74 - 6.88	1.81		0.31	0.23 - 0.42
20-39	24hU	DE	ESB 2017	2017	F,M	60		0.05	5.00	0.30	0.25 - 0.41	0.65	0.41 - 1.16	1.31	0.93 - 5.10	1.88		0.34	0.25 - 0.45

Notes. Matrix: mU - first morning urine, sU - spot urine, 24hU - 24-hours urine; country: AT - Austria, BE - Belgium, CZ - Czech Republic, DE - Germany, DK - Denmark, ES - Spain, HU - Hungary, IL - Israel, LU - Luxemburg, NO - Norway, PL - Poland, SE - Sweden, SI - Slovenia, SK - Slovakia; sex: F - female, M - male; n - sample size; LOD - limit of detection; LOQ - limit of quantification; % below limit - percentage of values below limit of detection/quantification, whichever was applied in the data collection; P50, P75, P90, P95 - 50th, 75th, 90th, 95th percentile; 95% CI - 95 % confidence interval; GM - geometric mean; NA - Not available.

Data only displayed if n was at least 50; percentiles missing when below LOD/LOQ; CIs of the observed percentiles missing when they fall outside the observed range; GM was imputed (fixed imputation) based on a log-normal distribution when at least 50 % of values were above LOD/LOQ.

Table S23: DEHTP metabolite 2cx-MMHTP (1-mono-(2-carboxyl-methyl-hexyl) benzene-1,4-dicarboxylate) in microg/L

Age	Matrix	Country	Data collection	Sampling year	Sex	N	LOD	LOQ	% below limit	P50	P50_95% CI	P75	P75_95% CI	P90	P90_95% CI	P95	P95_95% CI	GM	GM_95% CI
3-5	mU	DE	GerES V	2015 - 2017	F,M	333		0.40	61.3			0.58	0.51 - 0.70	1.22	0.98 - 1.58	1.73	1.53 - 2.83		
6-11	mU	DE	GerES V	2015 - 2017	F,M	890		0.40	77.1					0.70	0.61 - 0.79	1.03	0.91 - 1.40		
12-19	mU	DE	GerES V	2015 - 2017	F,M	895		0.40	90.8							0.58	0.51 - 0.81		

Notes. Matrix: mU - first morning urine, sU - spot urine, 24hU - 24-hours urine; country: AT - Austria, BE - Belgium, CZ - Czech Republic, DE - Germany, DK - Denmark, ES - Spain, HU - Hungary, IL - Israel, LU - Luxemburg, NO - Norway, PL - Poland, SE - Sweden, SI - Slovenia, SK - Slovakia; sex: F - female, M - male; n - sample size; LOD - limit of detection; LOQ - limit of quantification; % below limit - percentage of values below limit of detection/quantification, whichever was applied in the data collection; P50, P75, P90, P95 - 50th, 75th, 90th, 95th percentile; 95% CI - 95 % confidence interval; GM - geometric mean; NA - Not available.

Data only displayed if n was at least 50; percentiles missing when below LOD/LOQ; CIs of the observed percentiles missing when they fall outside the observed range; GM was imputed (fixed imputation) based on a log-normal distribution when at least 50 % of values were above LOD/LOQ.

Table S24: DEHTP metabolite 5cx-MEPTP (1-mono-(2-ethyl-5-carboxyl-pentyl) benzene-1,4-dicarboxylate) in microg/L

Age	Matrix	Country	Data collection	Sampling year	Sex	N	LOD	LOQ	% below limit	P50	P50_95% CI	P75	P75_95% CI	P90	P90_95% CI	P95	P95_95% CI	GM	GM_95% CI
3-5	mU	DE	GerES V	2015 - 2017	F,M	333		0.20	0.00	13.5	11.9 - 15.7	28.2	23.1 - 35.2	53.3	46.9 - 73.6	87.1	71.4 - 141	14.5	12.9 - 16.3
6-11	mU	DE	GerES V	2015 - 2017	F,M	890		0.20	0.00	8.51	8.00 - 9.30	17.5	16.0 - 18.8	33.0	28.8 - 39.0	53.0	46.9 - 65.9	8.95	8.33 - 9.61
12-19	mU	DE	GerES V	2015 - 2017	F,M	895		0.20	0.22	4.85	4.49 - 5.18	9.44	8.41 - 10.3	18.5	15.8 - 21.4	30.3	25.2 - 44.0	5.11	4.75 - 5.49

Notes. Matrix: mU - first morning urine, sU - spot urine, 24hU - 24-hours urine; country: AT - Austria, BE - Belgium, CZ - Czech Republic, DE - Germany, DK - Denmark, ES - Spain, HU - Hungary, IL - Israel, LU - Luxemburg, NO - Norway, PL - Poland, SE - Sweden, SI - Slovenia, SK - Slovakia; sex: F - female, M - male; n - sample size; LOD - limit of detection; LOQ - limit of quantification; % below limit - percentage of values below limit of detection/quantification, whichever was applied in the data collection; P50, P75, P90, P95 - 50th, 75th, 90th, 95th percentile; 95% CI - 95 % confidence interval; GM - geometric mean; NA - Not available.

Data only displayed if n was at least 50; percentiles missing when below LOD/LOQ; CIs of the observed percentiles missing when they fall outside the observed range; GM was imputed (fixed imputation) based on a log-normal distribution when at least 50 % of values were above LOD/LOQ.

Table S25: DEHTP metabolite 5OH-MEHTP (1-mono-(2-ethyl-5-hydroxy-hexyl) benzene-1,4-dicarboxylate) in microg/L

Age	Matrix	Country	Data collection	Sampling year	Sex	N	LOD	LOQ	% below limit	P50	P50_95% CI	P75	P75_95% CI	P90	P90_95% CI	P95	P95_95% CI	GM	GM_95% CI
3-5	mU	DE	GerES V	2015 - 2017	F,M	333		0.30	19.5	0.97	0.78 - 1.18	2.25	1.94 - 2.69	4.57	3.65 - 5.90	8.36	5.82 - 12.3	0.94	0.82 - 1.08
6-11	mU	DE	GerES V	2015 - 2017	F,M	890		0.30	28.0	0.61	0.54 - 0.67	1.42	1.29 - 1.58	2.91	2.65 - 3.46	4.40	3.94 - 6.30	0.62	0.57 - 0.67
12-19	mU	DE	GerES V	2015 - 2017	F,M	895		0.30	44.4	0.34	0.31 - 0.37	0.76	0.70 - 0.89	1.83	1.68 - 2.07	2.79	2.40 - 4.07	0.35	0.32 - 0.38

Notes. Matrix: mU - first morning urine, sU - spot urine, 24hU - 24-hours urine; country: AT - Austria, BE - Belgium, CZ - Czech Republic, DE - Germany, DK - Denmark, ES - Spain, HU - Hungary, IL - Israel, LU - Luxemburg, NO - Norway, PL - Poland, SE - Sweden, SI - Slovenia, SK - Slovakia; sex: F - female, M - male; n - sample size; LOD - limit of detection; LOQ - limit of quantification; % below limit - percentage of values below limit of detection/quantification, whichever was applied in the data collection; P50, P75, P90, P95 - 50th, 75th, 90th, 95th percentile; 95% CI - 95 % confidence interval; GM - geometric mean; NA - Not available.

Data only displayed if n was at least 50; percentiles missing when below LOD/LOQ; CIs of the observed percentiles missing when they fall outside the observed range; GM was imputed (fixed imputation) based on a log-normal distribution when at least 50 % of values were above LOD/LOQ.

Table S26: DEHTP metabolite 5oxo-MEHTP (1-mono-(2-ethyl-5-oxo-hexyl) benzene-1,4-dicarboxylate) in microg/L

Age	Matrix	Country	Data collection	Sampling year	Sex	N	LOD	LOQ	% below limit	P50	P50_95% CI	P75	P75_95% CI	P90	P90_95% CI	P95	P95_95% CI	GM	GM_95% CI
3-5	mU	DE	GerES V	2015 - 2017	F,M	333		0.20	9.31	1.01	0.80 - 1.21	2.13	1.91 - 2.42	4.21	3.24 - 5.81	7.26	5.42 - 11.5	0.97	0.86 - 1.11
6-11	mU	DE	GerES V	2015 - 2017	F,M	890		0.20	16.6	0.66	0.61 - 0.72	1.39	1.25 - 1.53	2.74	2.36 - 3.14	4.08	3.53 - 5.26	0.64	0.59 - 0.69
12-19	mU	DE	GerES V	2015 - 2017	F,M	895		0.20	30.3	0.34	0.32 - 0.37	0.80	0.71 - 0.89	1.58	1.38 - 1.79	2.44	2.09 - 3.75	0.36	0.34 - 0.39

Notes. Matrix: mU - first morning urine, sU - spot urine, 24hU - 24-hours urine; country: AT - Austria, BE - Belgium, CZ - Czech Republic, DE - Germany, DK - Denmark, ES - Spain, HU - Hungary, IL - Israel, LU - Luxemburg, NO - Norway, PL - Poland, SE - Sweden, SI - Slovenia, SK - Slovakia; sex: F - female, M - male; n - sample size; LOD - limit of detection; LOQ - limit of quantification; % below limit - percentage of values below limit of detection/quantification, whichever was applied in the data collection; P50, P75, P90, P95 - 50th, 75th, 90th, 95th percentile; 95% CI - 95 % confidence interval; GM - geometric mean; NA - Not available.

Data only displayed if n was at least 50; percentiles missing when below LOD/LOQ; CIs of the observed percentiles missing when they fall outside the observed range; GM was imputed (fixed imputation) based on a log-normal distribution when at least 50 % of values were above LOD/LOQ.

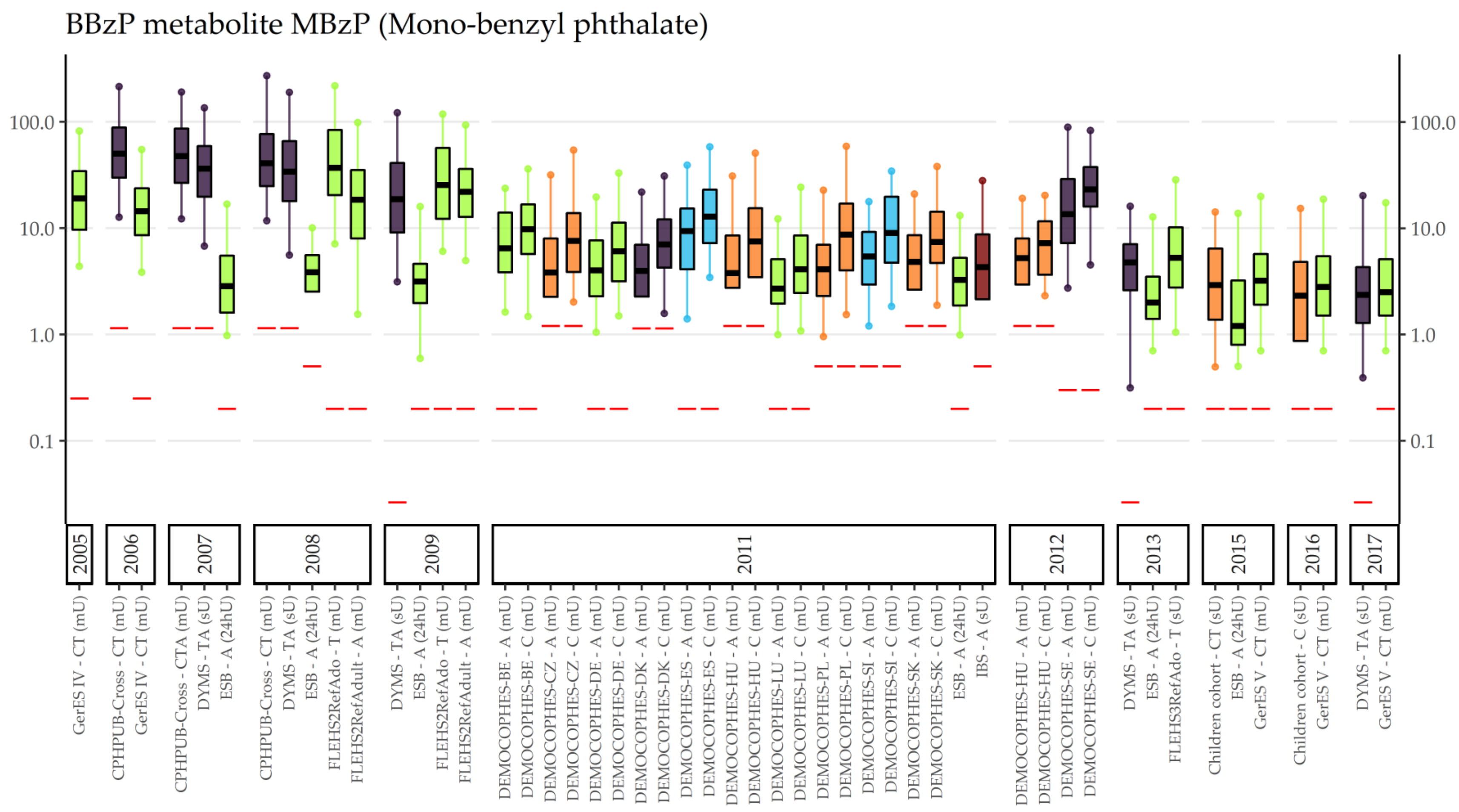

Figure S1. BBzP metabolite MBzP (Mono-benzyl phthalate) concentration in µg/L stratified by sampling year in boxplots.

The box is based on P25, P50, and P75, and the whiskers are based on P5 and P95. Within sampling years plots are sorted by country. Colors of the boxplots refer to the data collection's region (North = violet, South = blue, West = green, East = orange, Israel = dark red). Red lines denote LOQ/LOD for the respective study. Since percentiles are only available and displayed when they are above LOQ/LOD the lower side of boxplots might be truncated. The age categories are shown in capital letters: children of the age groups 3-5 and 6-11 (C), adolescents between 12 and 19 years (T) and adults from the age groups 20-39, 40-59 and 60 years and older (A). The sampling method is indicated in brackets: first morning urine void (mU), spot urine (sU) and 24-hours urine (24hU).

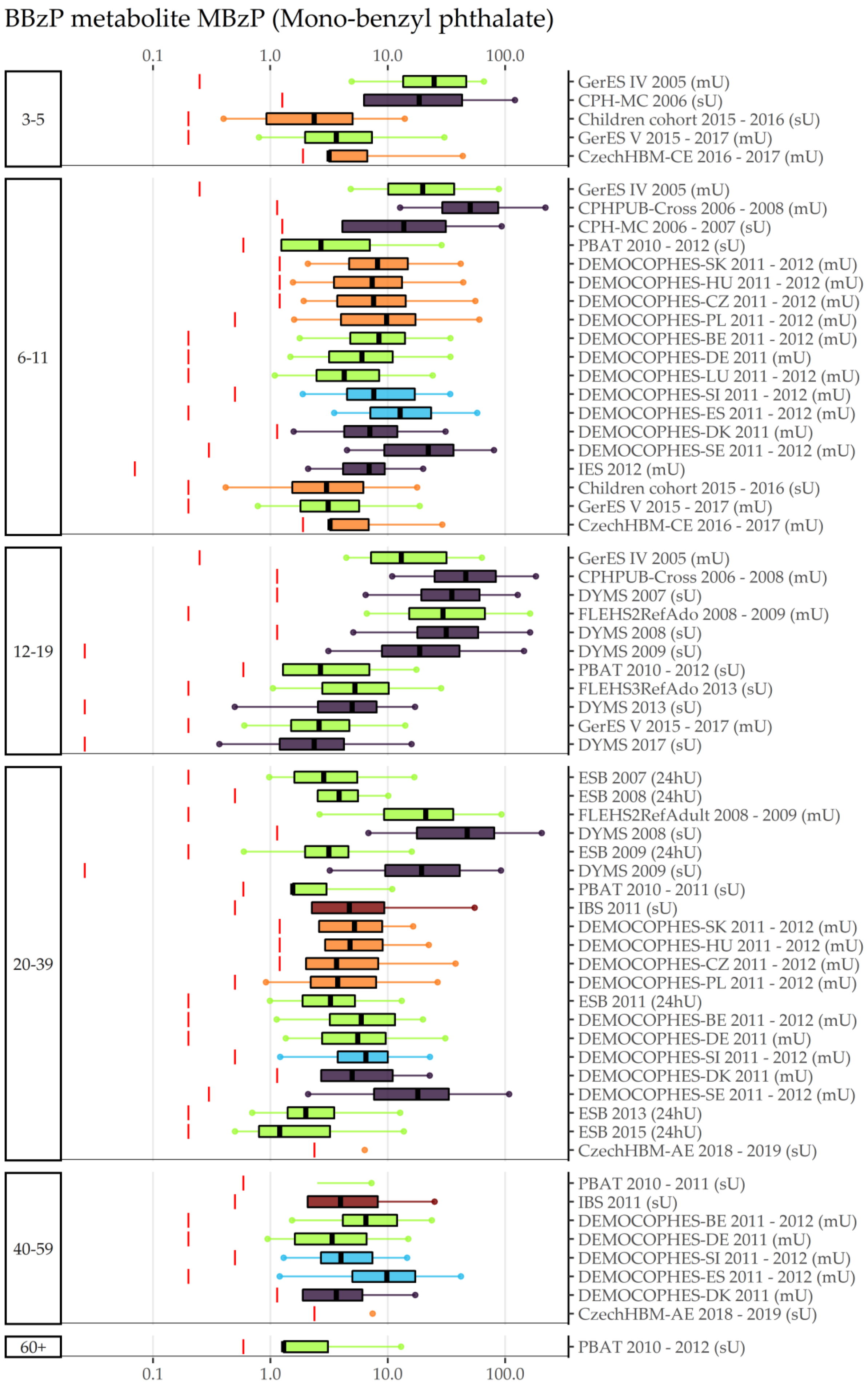

Figure S2. BBzP metabolite MBzP (Mono-benzyl phthalate) concentration in µg/L stratified by age groups in boxplots.

The box is based on P25, P50, and P75, and the whiskers are based on P5 and P95. Within age groups plots are chronologically sorted by year of sampling. Colors of the boxplots refer to the data collection's region (North = violet, South = blue, West = green, East = orange, Israel = dark red). Red lines denote LOQ/LOD for the respective study. Since percentiles are only available and displayed when they are above LOQ/LOD the lower side of boxplots might be truncated. The sampling method is indicated in brackets: first morning urine void (mU), spot urine (sU) and 24-hours urine (24hU).

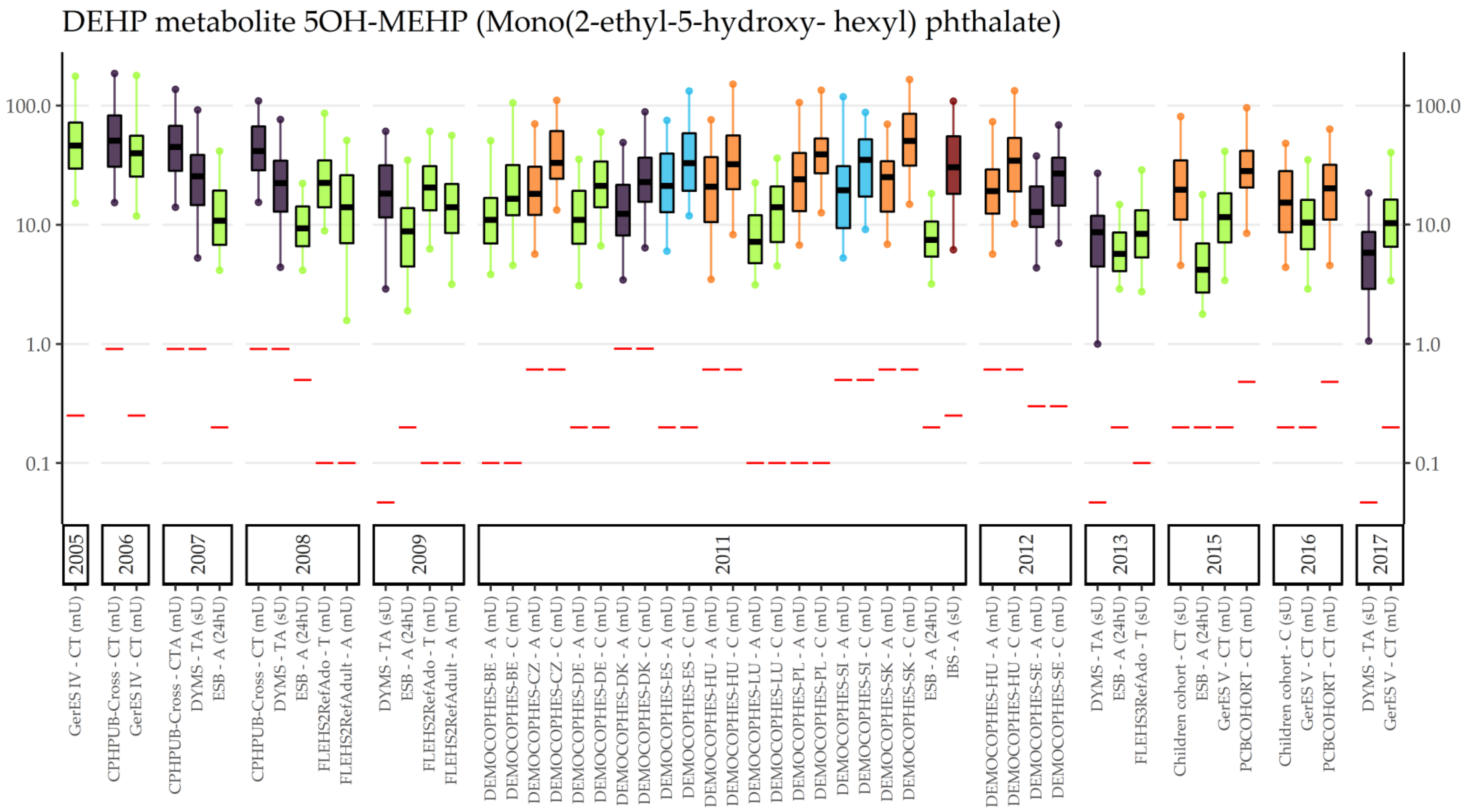

Figure S3. DEHP metabolite 5OH-MEHP (Mono(2-ethyl-5-hydroxy- hexyl) phthalate) concentration in µg/L stratified by sampling year in boxplots.

The box is based on P25, P50, and P75, and the whiskers are based on P5 and P95. Within sampling years plots are sorted by country. Colors of the boxplots refer to the data collection's region (North = violet, South = blue, West = green, East = orange, Israel = dark red). Red lines denote LOQ/LOD for the respective study. Since percentiles are only available and displayed when they are above LOQ/LOD the lower side of boxplots might be truncated. The age categories are shown in capital letters: children of the age groups 3-5 and 6-11 (C), adolescents between 12 and 19 years (T) and adults from the age groups 20-39, 40-59 and 60 years and older (A). The sampling method is indicated in brackets: first morning urine void (mU), spot urine (sU) and 24-hours urine (24hU).

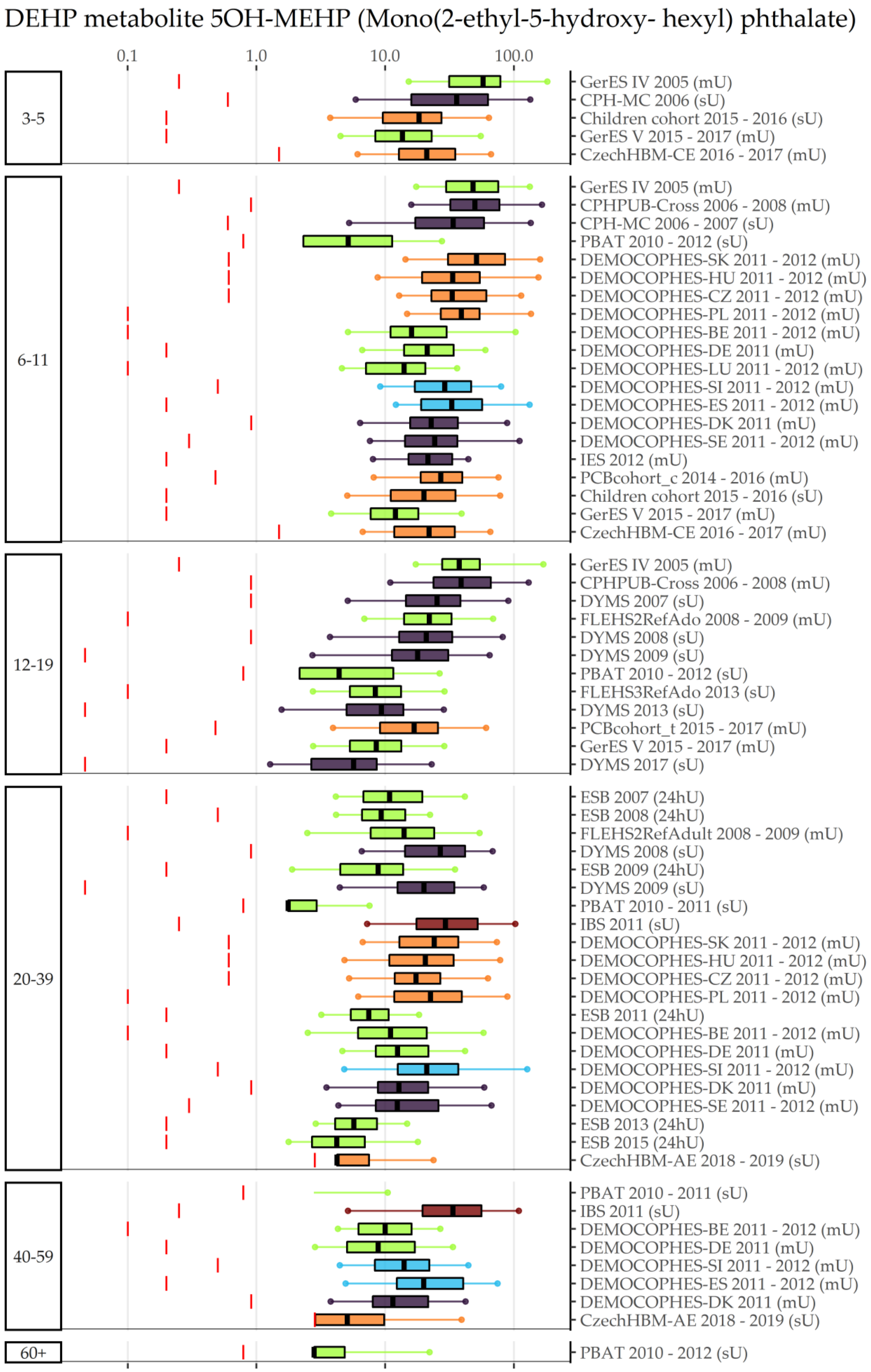

Figure S4. DEHP metabolite 5OH-MEHP (Mono(2-ethyl-5-hydroxy- hexyl) phthalate) concentration in μg/L stratified by age groups in boxplots.

The box is based on P25, P50, and P75, and the whiskers are based on P5 and P95. Within age groups plots are chronologically sorted by year of sampling. Colors of the boxplots refer to the data collection's region (North = violet, South = blue, West = green, East = orange, Israel = dark red). Red lines denote LOQ/LOD for the respective study. Since percentiles are only available and displayed when they are above LOQ/LOD the lower side of boxplots might be truncated. The sampling method is indicated in brackets: first morning urine void (mU), spot urine (sU) and 24-hours urine (24hU).

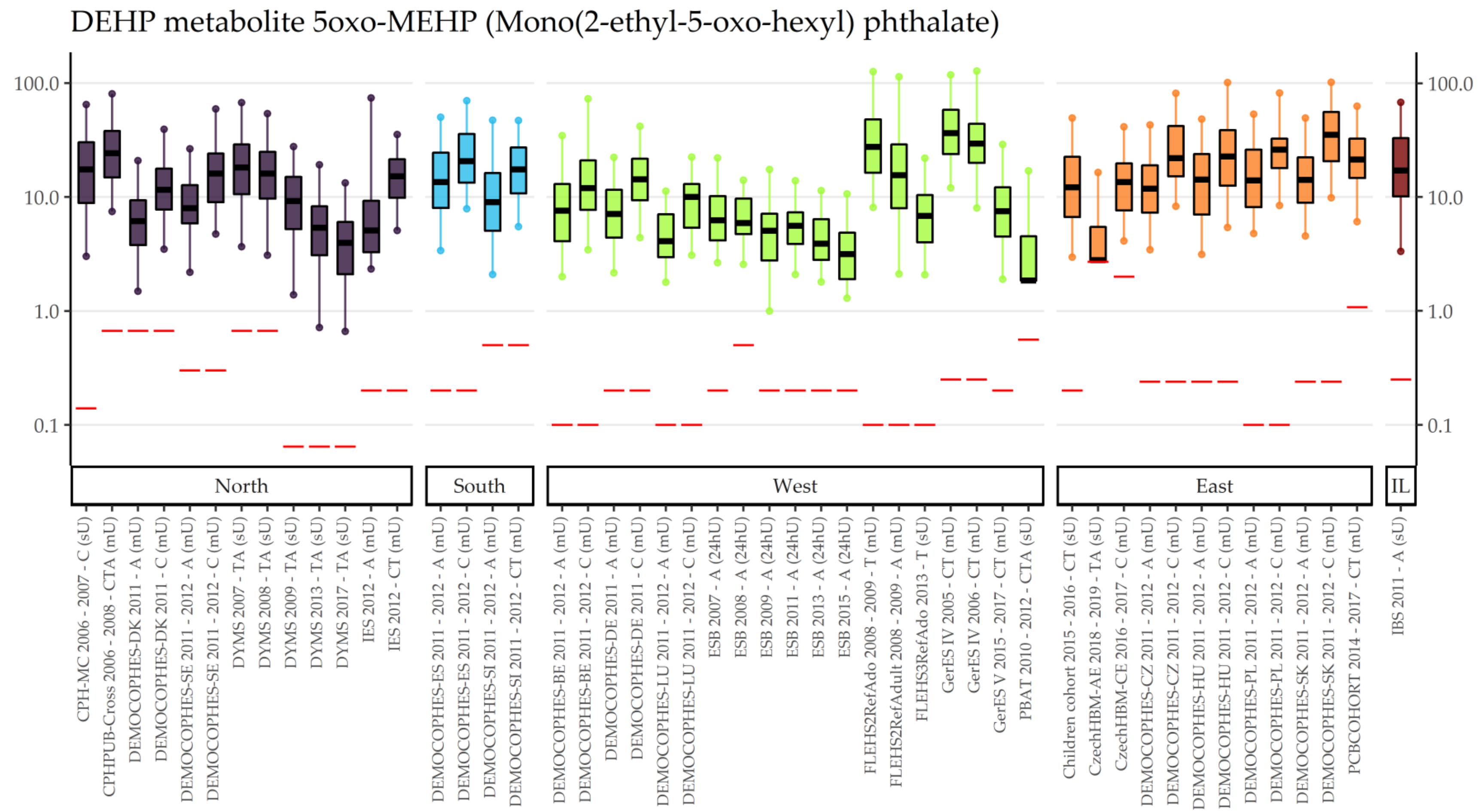

Figure S5. DEHP metabolite 5oxo-MEHP (Mono(2-ethyl-5-oxo-hexyl) phthalate) concentration in µg/L stratified by region in boxplots.

The box is based on P25, P50, and P75, and the whiskers are based on P5 and P95. Within regions plots are sorted by country and year of sampling. Colors of the boxplots refer to the data collection's region (North = violet, South = blue, West = green, East = orange, Israel = dark red). Red lines denote LOQ/LOD for the respective study. Since percentiles are only available and displayed when they are above LOQ/LOD the lower side of boxplots might be truncated. The age categories are shown in capital letters: children of the age groups 3-5 and 6-11 (C), adolescents between 12 and 19 years (T) and adults from the age groups 20-39, 40-59 and 60 years and older (A). The sampling method is indicated in brackets: first morning urine void (mU), spot urine (sU) and 24-hours urine (24hU).

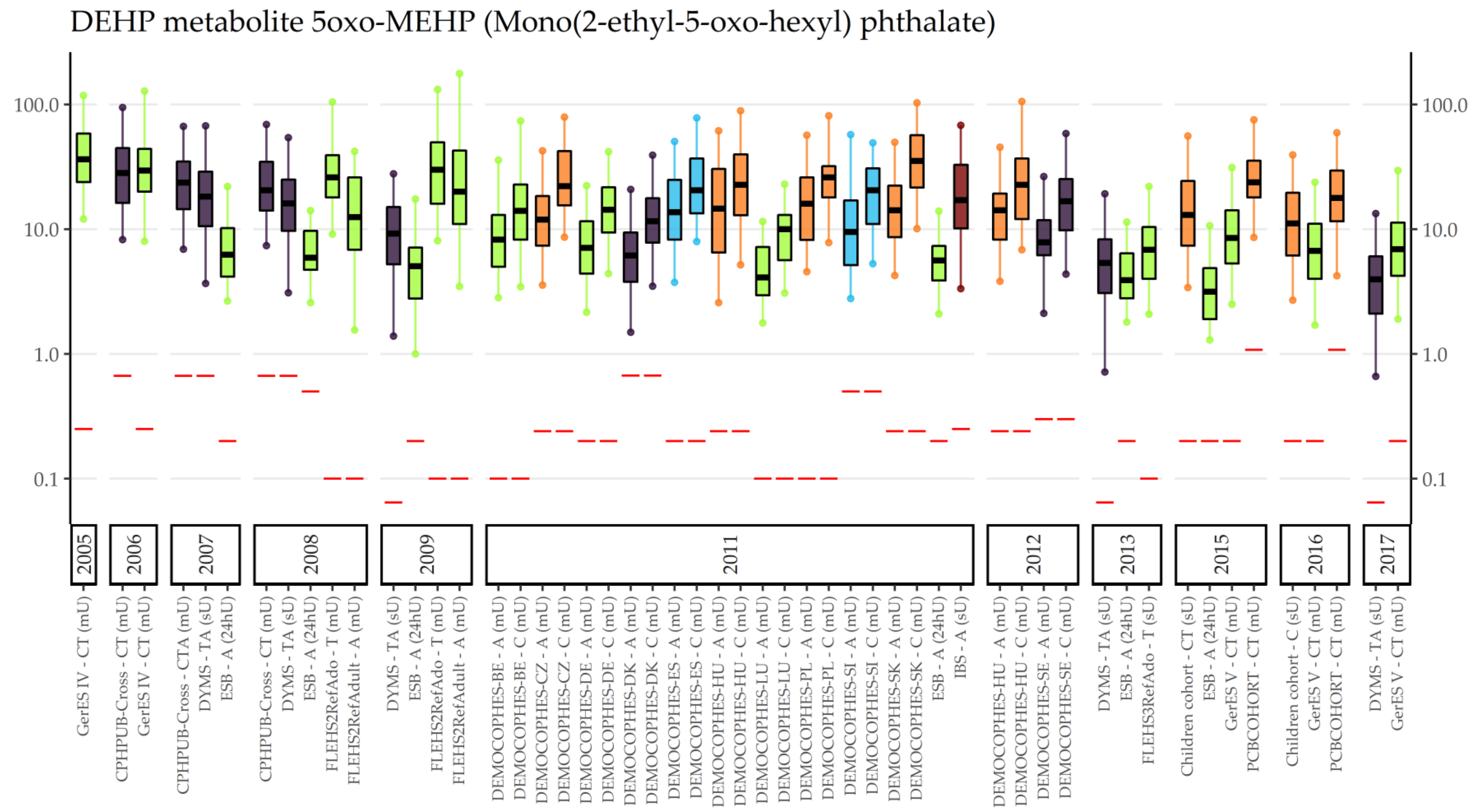

Figure S6. DEHP metabolite 5oxo-MEHP (Mono(2-ethyl-5-oxo-hexyl) phthalate) concentration in µg/L stratified by sampling year in boxplots.

The box is based on P25, P50, and P75, and the whiskers are based on P5 and P95. Within sampling years plots are sorted by country. Colors of the boxplots refer to the data collection's region (North = violet, South = blue, West = green, East = orange, Israel = dark red). Red lines denote LOQ/LOD for the respective study. Since percentiles are only available and displayed when they are above LOQ/LOD the lower side of boxplots might be truncated. The age categories are shown in capital letters: children of the age groups 3-5 and 6-11 (C), adolescents between 12 and 19 years (T) and adults from the age groups 20-39, 40-59 and 60 years and older (A). The sampling method is indicated in brackets: first morning urine void (mU), spot urine (sU) and 24-hours urine (24hU).

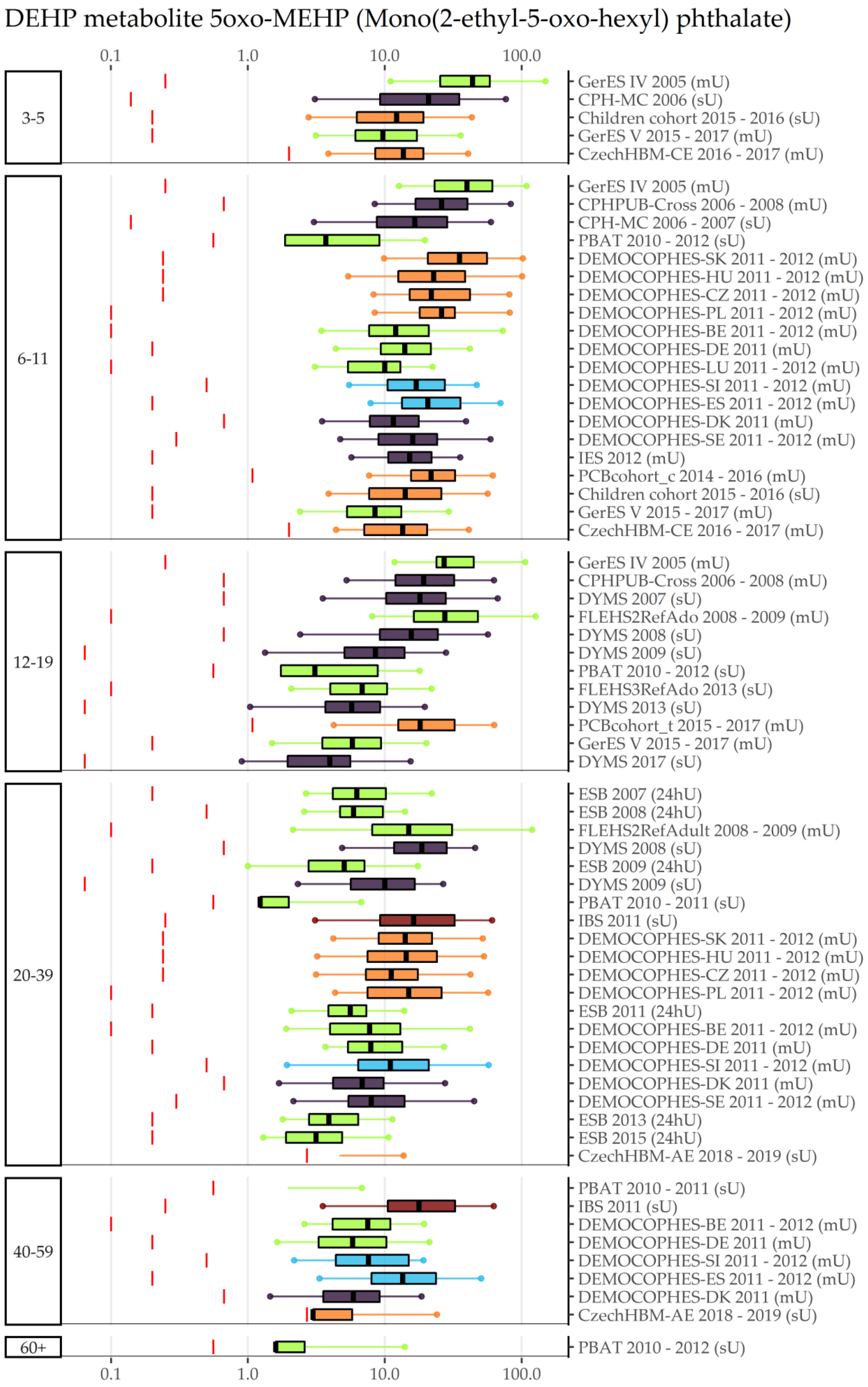

Figure S7. DEHP metabolite 5oxo-MEHP (Mono(2-ethyl-5-oxo-hexyl) phthalate) concentration in µg/L stratified by age groups in boxplots.

The box is based on P25, P50, and P75, and the whiskers are based on P5 and P95. Within age groups plots are chronologically sorted by year of sampling. Colors of the boxplots refer to the data collection's region (North = violet, South = blue, West = green, East = orange, Israel = dark red). Red lines denote LOQ/LOD for the respective study. Since percentiles are only available and displayed when they are above LOQ/LOD the lower side of boxplots might be truncated. The sampling method is indicated in brackets: first morning urine void (mU), spot urine (sU) and 24-hours urine (24hU).

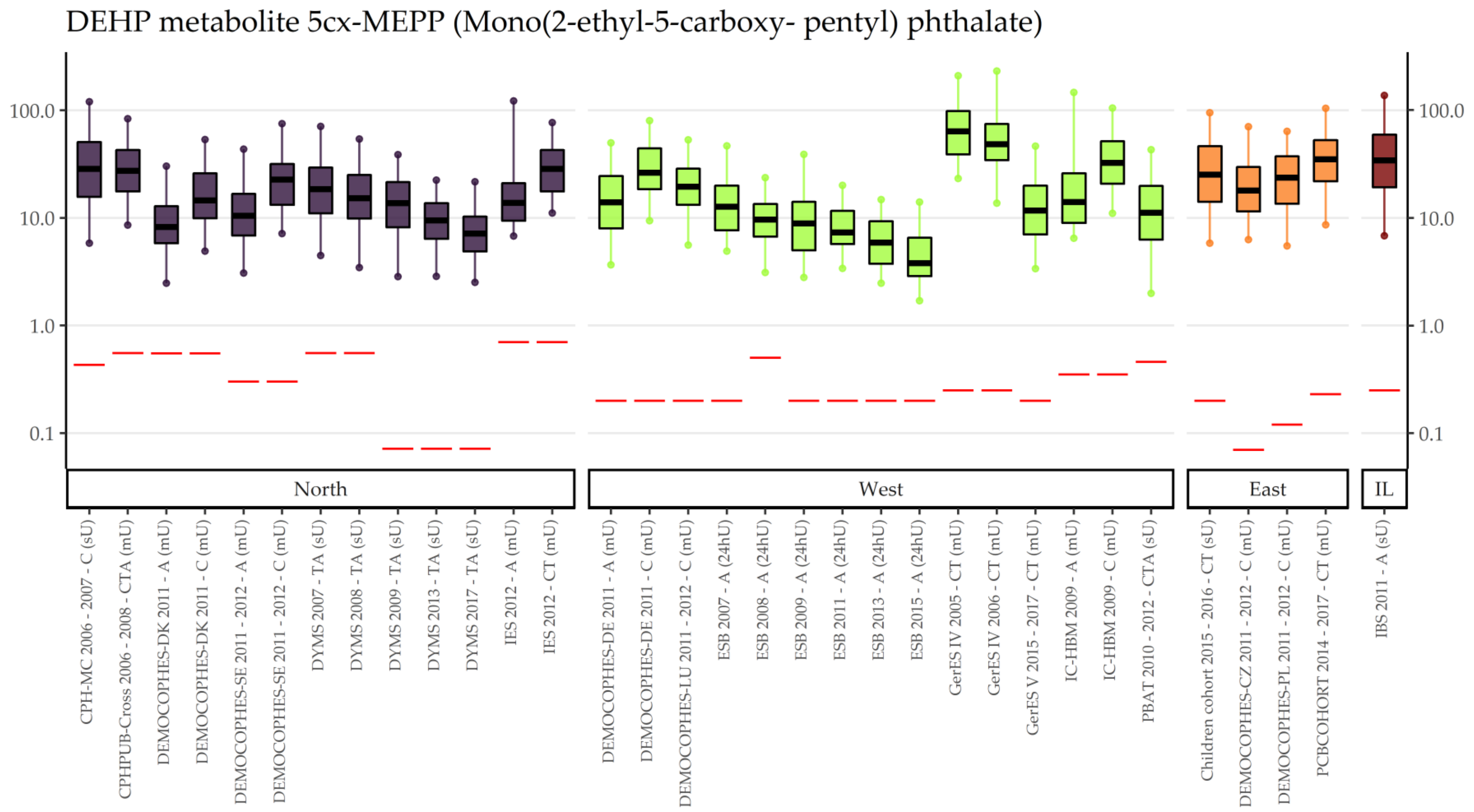

Figure S8. DEHP metabolite 5cx-MEPP (Mono(2-ethyl-5-carboxy- pentyl) phthalate) concentration in µg/L stratified by region in boxplots.

The box is based on P25, P50, and P75, and the whiskers are based on P5 and P95. Within regions plots are sorted by country and year of sampling. Colors of the boxplots refer to the data collection's region (North = violet, South = blue, West = green, East = orange, Israel = dark red). Red lines denote LOQ/LOD for the respective study. Since percentiles are only available and displayed when they are above LOQ/LOD the lower side of boxplots might be truncated. The age categories are shown in capital letters: children of the age groups 3-5 and 6-11 (C), adolescents between 12 and 19 years (T) and adults from the age groups 20-39, 40-59 and 60 years and older (A). The sampling method is indicated in brackets: first morning urine void (mU), spot urine (sU) and 24-hours urine (24hU).

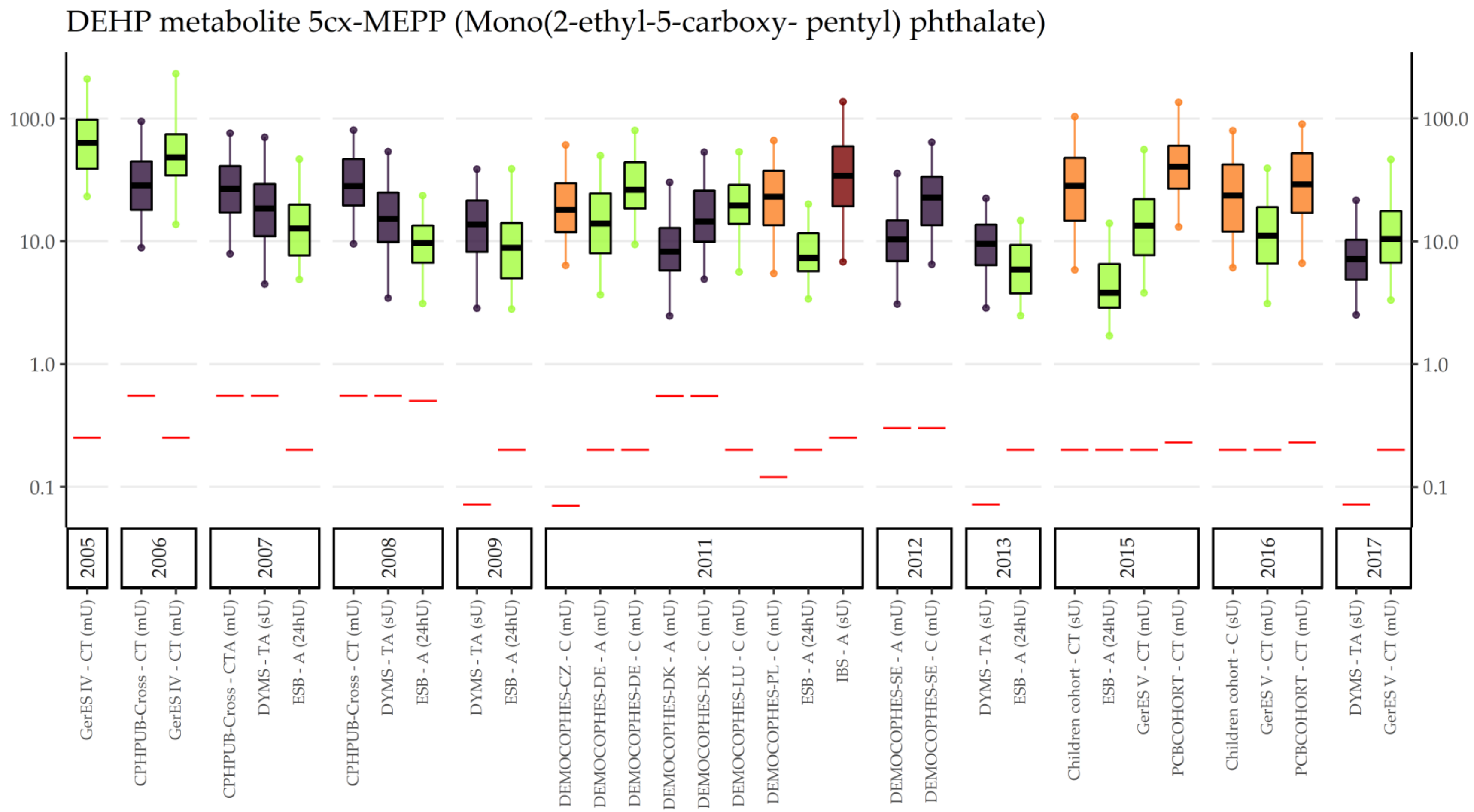

Figure S9. DEHP metabolite 5cx-MEPP (Mono(2-ethyl-5-carboxy- pentyl) phthalate) concentration in µg/L stratified by sampling year in boxplots.

The box is based on P25, P50, and P75, and the whiskers are based on P5 and P95. Within sampling years plots are sorted by country. Colors of the boxplots refer to the data collection’s region (North = violet, South = blue, West = green, East = orange, Israel = dark red). Red lines denote LOQ/LOD for the respective study. Since percentiles are only available and displayed when they are above LOQ/LOD the lower side of boxplots might be truncated. The age categories are shown in capital letters: children of the age groups 3-5 and 6-11 (C), adolescents between 12 and 19 years (T) and adults from the age groups 20-39, 40-59 and 60 years and older (A). The sampling method is indicated in brackets: first morning urine void (mU), spot urine (sU) and 24-hours urine (24hU).

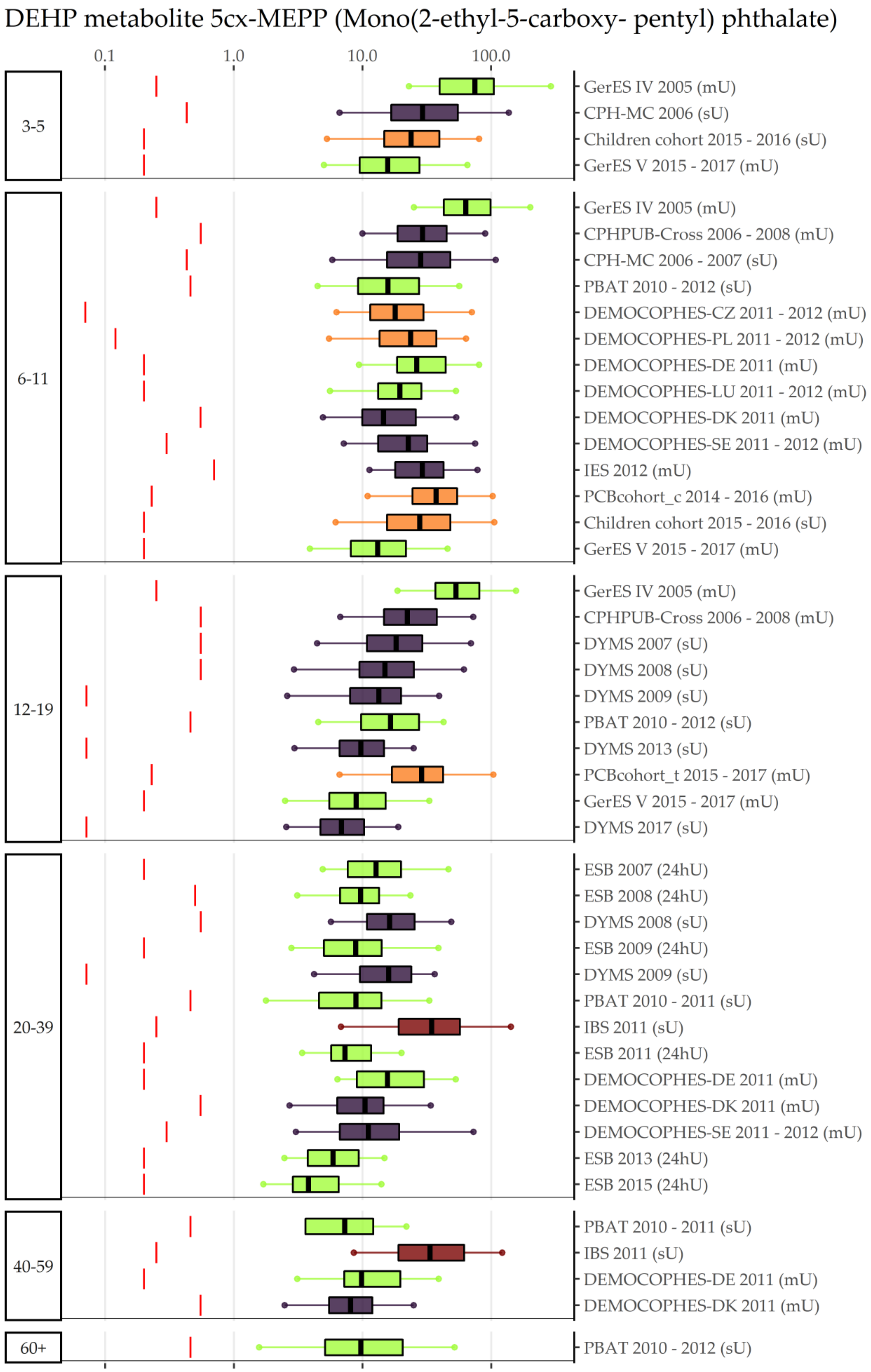

Figure S10. DEHP metabolite 5cx-MEPP (Mono(2-ethyl-5-carboxy- pentyl) phthalate) concentration in µg/L stratified by age groups in boxplots.

The box is based on P25, P50, and P75, and the whiskers are based on P5 and P95. Within age groups plots are chronologically sorted by year of sampling. Colors of the boxplots refer to the data collection's region (North = violet, South = blue, West = green, East = orange, Israel = dark red). Red lines denote LOQ/LOD for the respective study. Since percentiles are only available and displayed when they are above LOQ/LOD the lower side of boxplots might be truncated. The sampling method is indicated in brackets: first morning urine void (mU), spot urine (sU) and 24-hours urine (24hU).

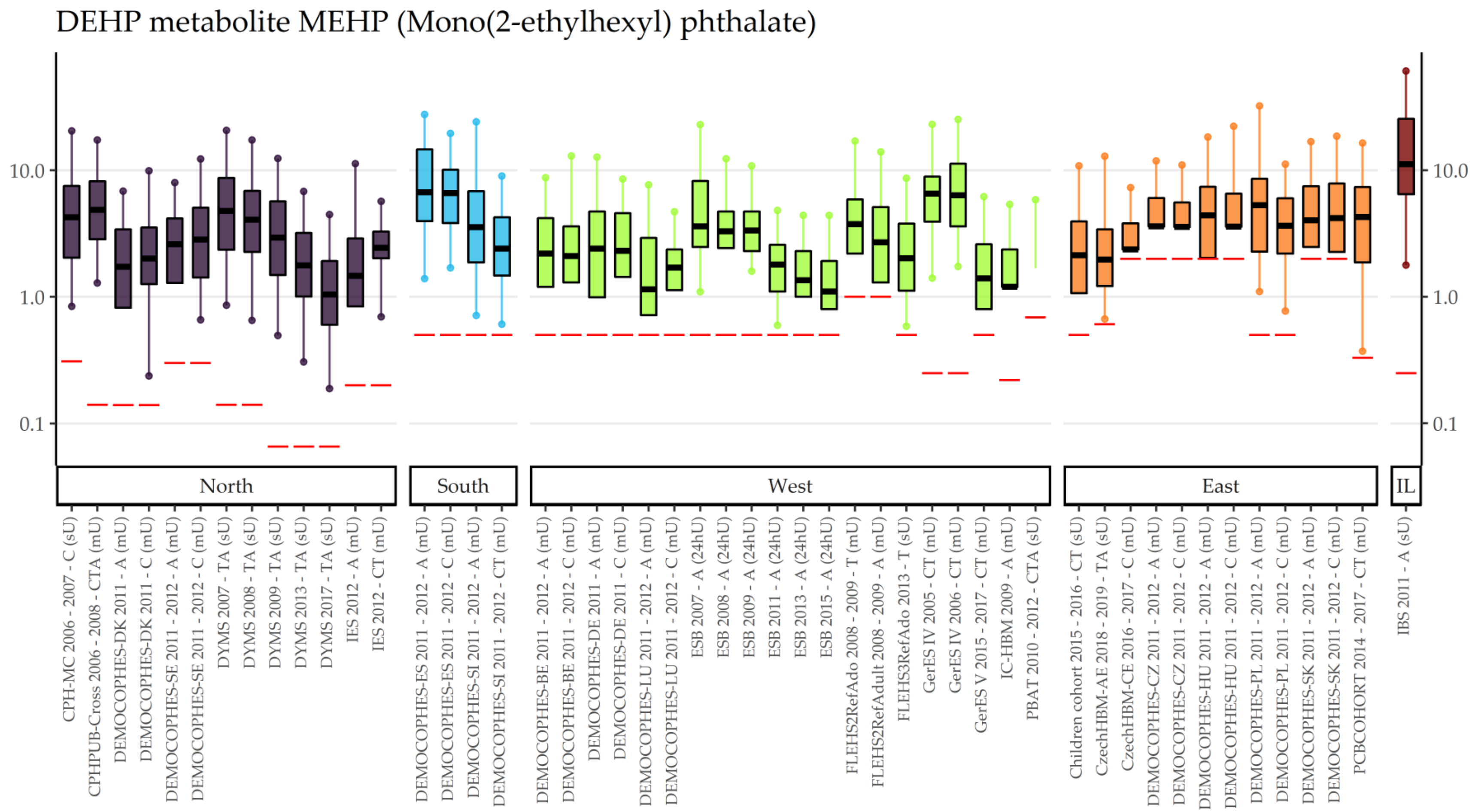

Figure S11. DEHP metabolite MEHP (Mono(2-ethylhexyl) phthalate) concentration in µg/L stratified by region in boxplots.

The box is based on P25, P50, and P75, and the whiskers are based on P5 and P95. Within regions plots are sorted by country and year of sampling. Colors of the boxplots refer to the data collection's region (North = violet, South = blue, West = green, East = orange, Israel = dark red). Red lines denote LOQ/LOD for the respective study. Since percentiles are only available and displayed when they are above LOQ/LOD the lower side of boxplots might be truncated. The age categories are shown in capital letters: children of the age groups 3-5 and 6-11 (C), adolescents between 12 and 19 years (T) and adults from the age groups 20-39, 40-59 and 60 years and older (A). The sampling method is indicated in brackets: first morning urine void (mU), spot urine (sU) and 24-hours urine (24hU).

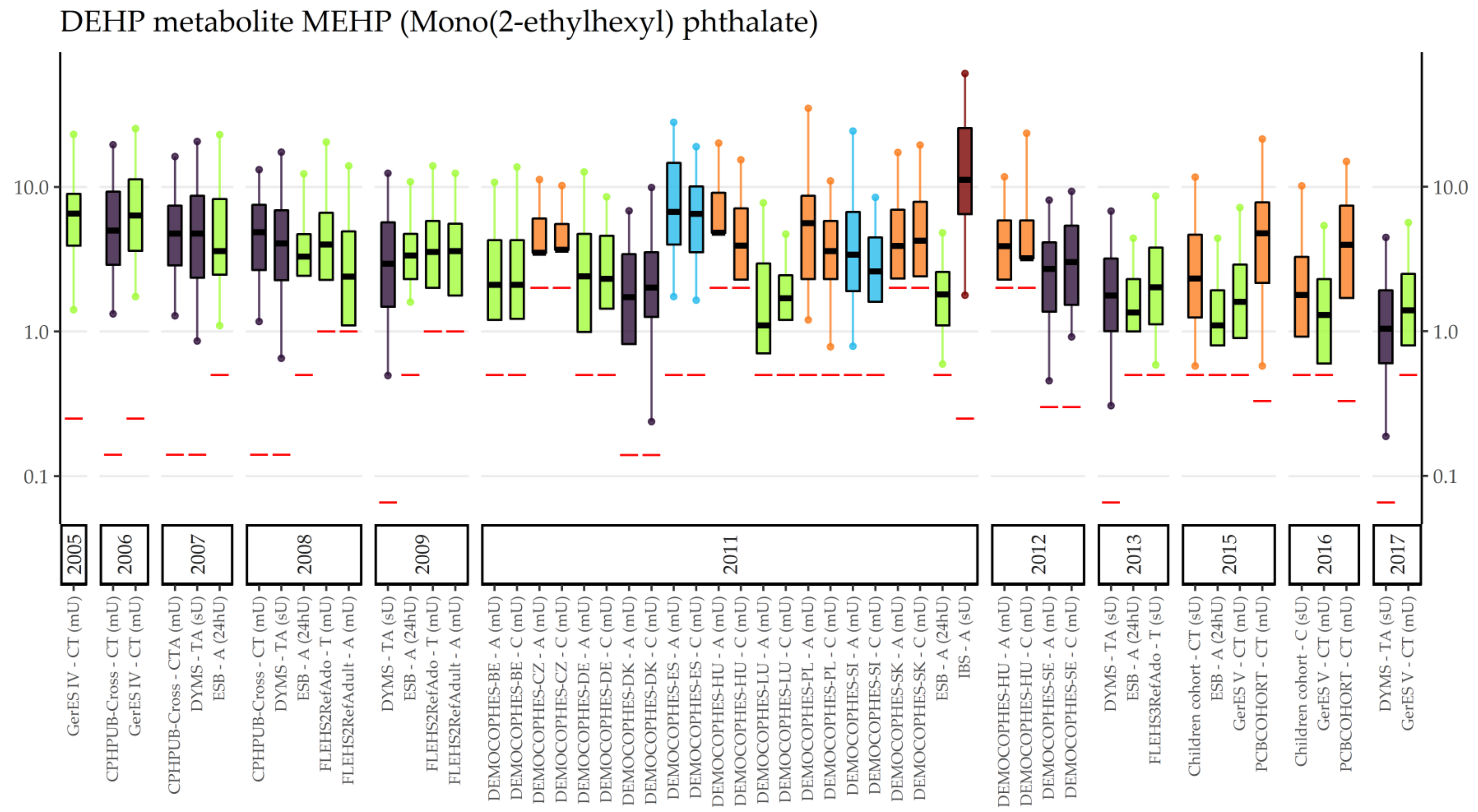

Figure S12. DEHP metabolite MEHP (Mono(2-ethylhexyl) phthalate) concentration in µg/L stratified by sampling year in boxplots.

The box is based on P25, P50, and P75, and the whiskers are based on P5 and P95. Within sampling years plots are sorted by country. Colors of the boxplots refer to the data collection's region (North = violet, South = blue, West = green, East = orange, Israel = dark red). Red lines denote LOQ/LOD for the respective study. Since percentiles are only available and displayed when they are above LOQ/LOD the lower side of boxplots might be truncated. The age categories are shown in capital letters: children of the age groups 3-5 and 6-11 (C), adolescents between 12 and 19 years (T) and adults from the age groups 20-39, 40-59 and 60 years and older (A). The sampling method is indicated in brackets: first morning urine void (mU), spot urine (sU) and 24-hours urine (24hU).

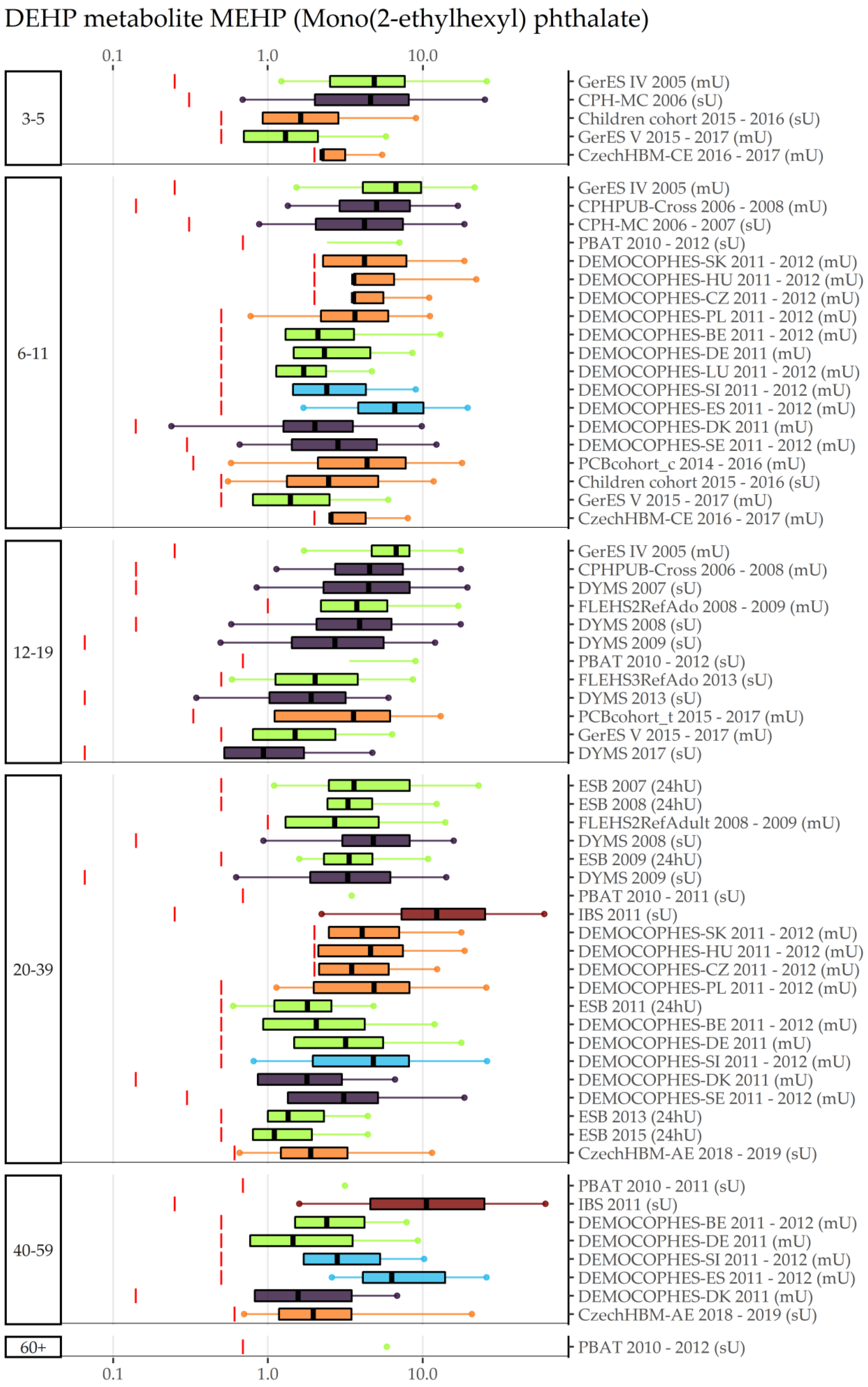

Figure S13. DEHP metabolite MEHP (Mono(2-ethylhexyl) phthalate) concentration in µg/L stratified by age groups in boxplots.

The box is based on P25, P50, and P75, and the whiskers are based on P5 and P95. Within age groups plots are chronologically sorted by year of sampling. Colors of the boxplots refer to the data collection's region (North = violet, South = blue, West = green, East = orange, Israel = dark red). Red lines denote LOQ/LOD for the respective study. Since percentiles are only available and displayed when they are above LOQ/LOD the lower side of boxplots might be truncated. The sampling method is indicated in brackets: first morning urine void (mU), spot urine (sU) and 24-hours urine (24hU).

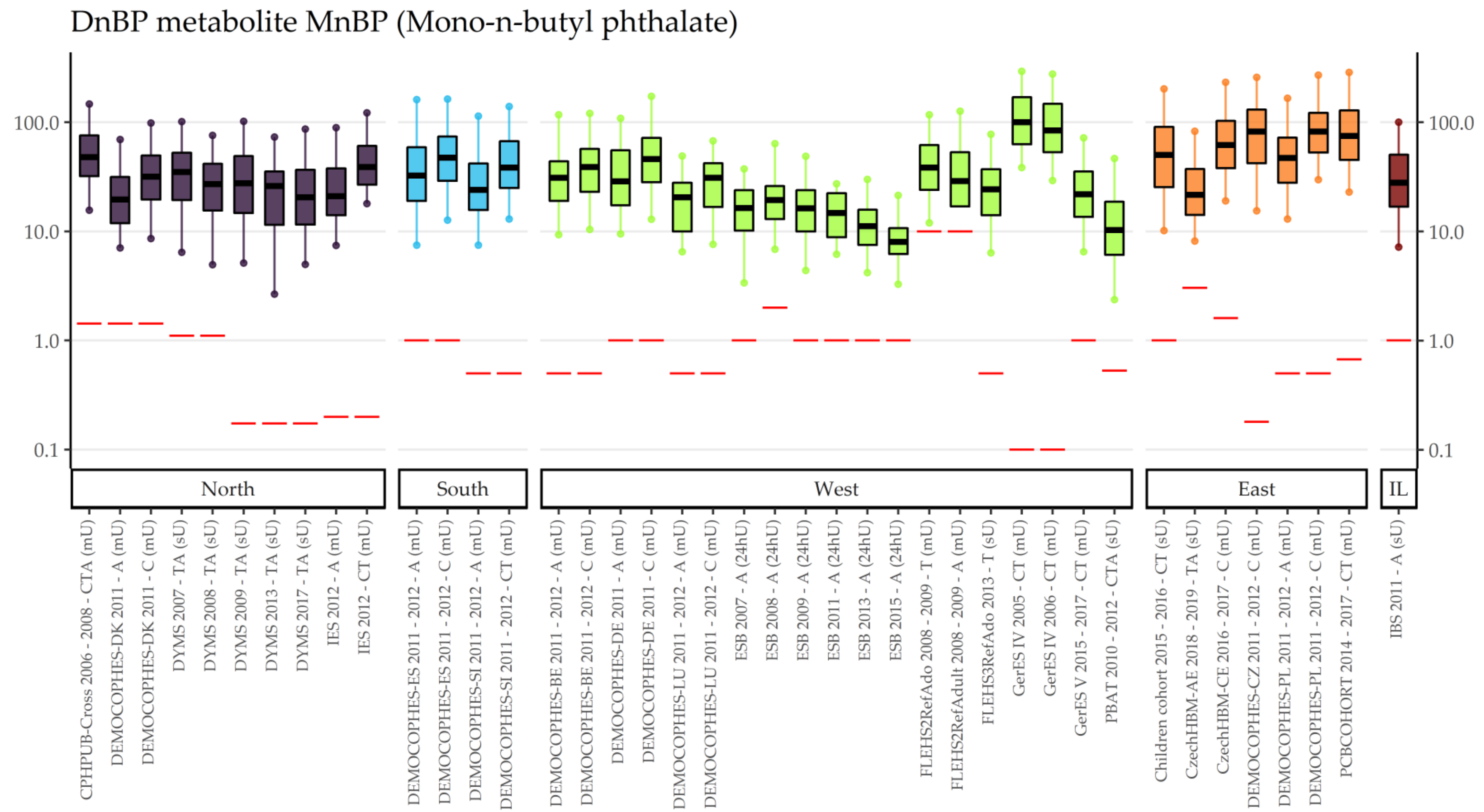

Figure S14. DnBP metabolite MnBP (Mono-n-butyl phthalate) concentration in µg/L stratified by region in boxplots.

The box is based on P25, P50, and P75, and the whiskers are based on P5 and P95. Within regions plots are sorted by country and year of sampling. Colors of the boxplots refer to the data collection's region (North = violet, South = blue, West = green, East = orange, Israel = dark red). Red lines denote LOQ/LOD for the respective study. Since percentiles are only available and displayed when they are above LOQ/LOD the lower side of boxplots might be truncated. The age categories are shown in capital letters: children of the age groups 3-5 and 6-11 (C), adolescents between 12 and 19 years (T) and adults from the age groups 20-39, 40-59 and 60 years and older (A). The sampling method is indicated in brackets: first morning urine void (mU), spot urine (sU) and 24-hours urine (24hU).

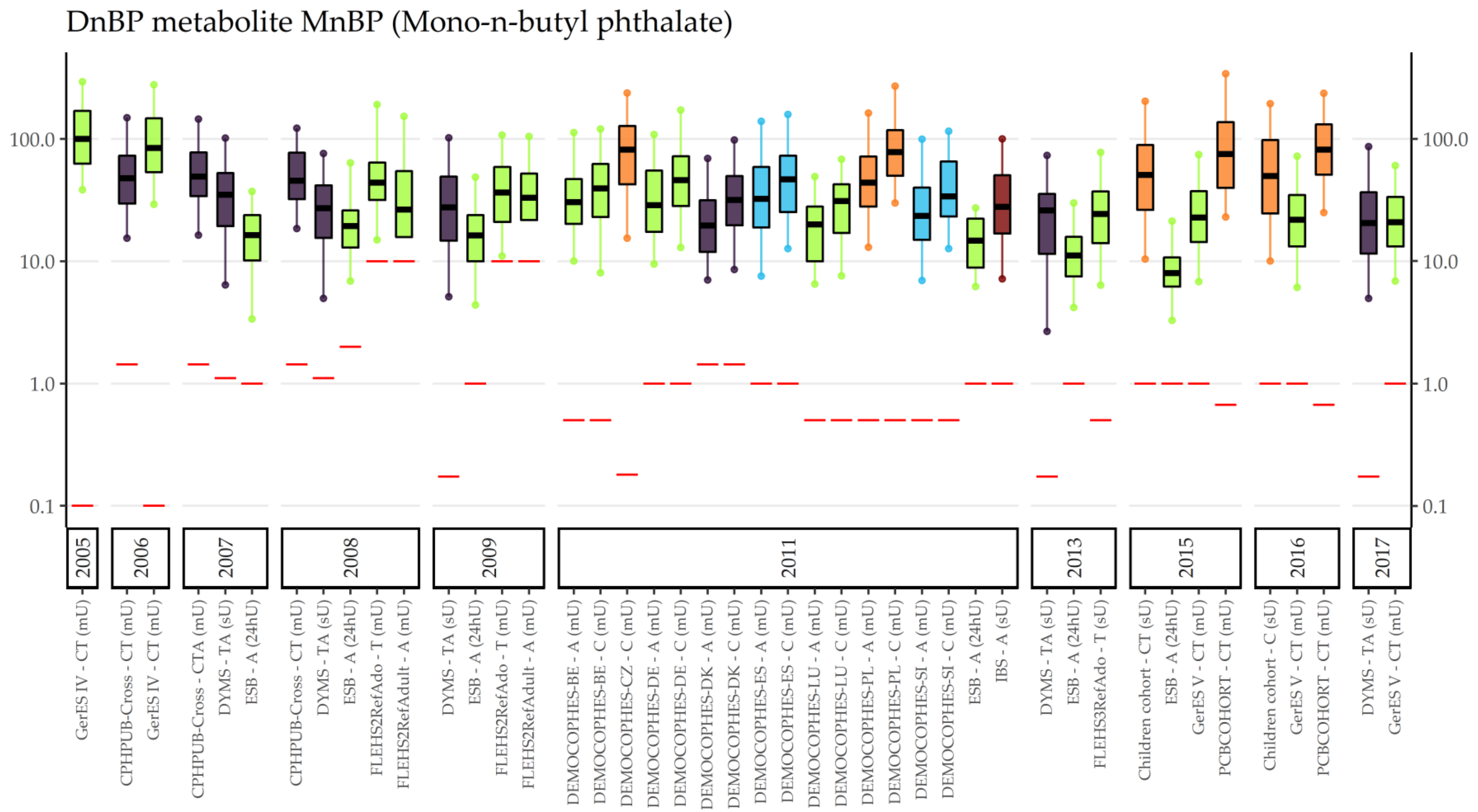

Figure S15. DnBP metabolite MnBP (Mono-n-butyl phthalate) concentration in µg/L stratified by sampling year in boxplots.

The box is based on P25, P50, and P75, and the whiskers are based on P5 and P95. Within sampling years plots are sorted by country. Colors of the boxplots refer to the data collection's region (North = violet, South = blue, West = green, East = orange, Israel = dark red). Red lines denote LOQ/LOD for the respective study. Since percentiles are only available and displayed when they are above LOQ/LOD the lower side of boxplots might be truncated. The age categories are shown in capital letters: children of the age groups 3-5 and 6-11 (C), adolescents between 12 and 19 years (T) and adults from the age groups 20-39, 40-59 and 60 years and older (A). The sampling method is indicated in brackets: first morning urine void (mU), spot urine (sU) and 24-hours urine (24hU).

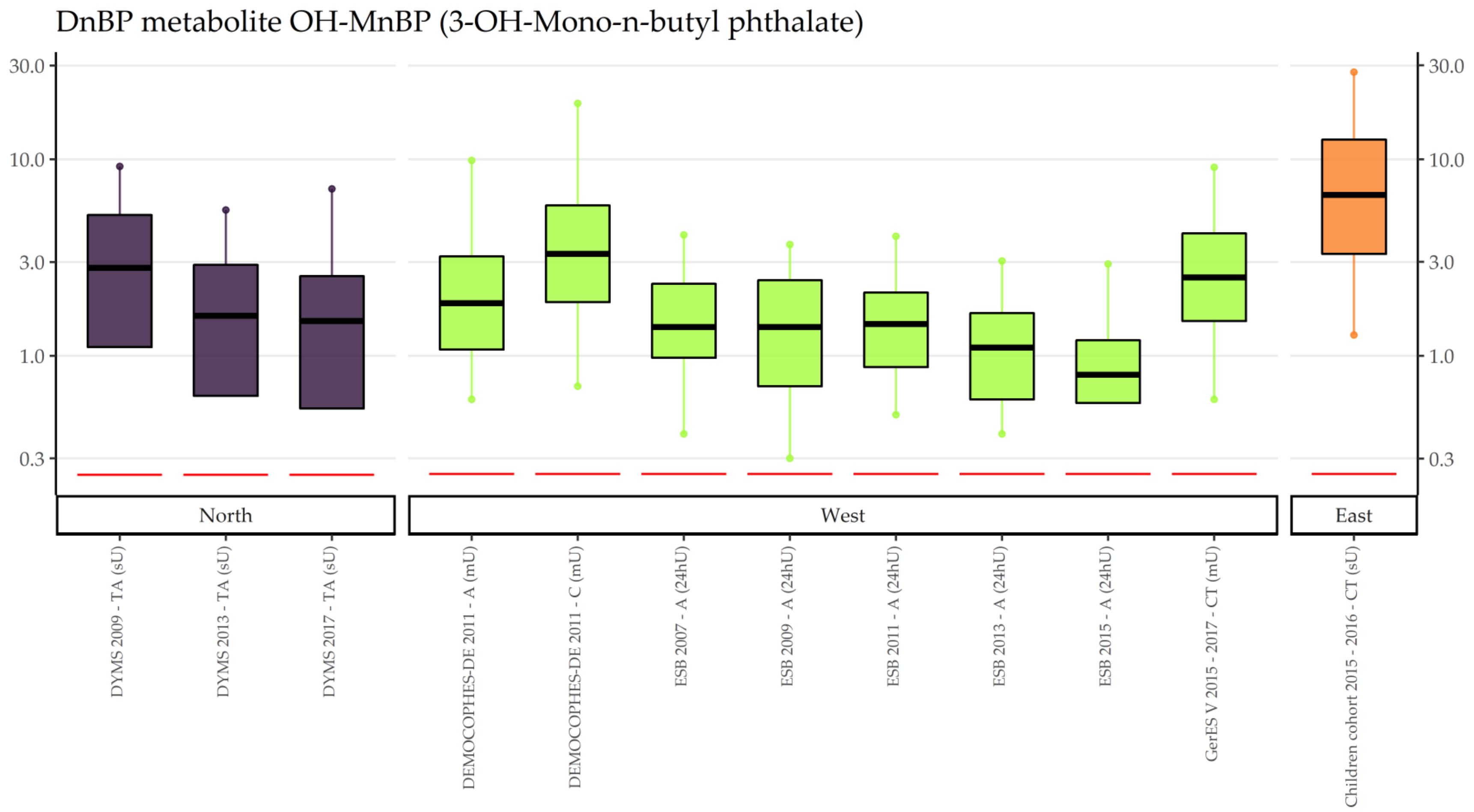

Figure S16. DnBP metabolite OH-MnBP (3-OH-Mono-n-butyl phthalate) concentration in µg/L stratified by region in boxplots.

The box is based on P25, P50, and P75, and the whiskers are based on P5 and P95. Within regions plots are sorted by country and year of sampling. Colors of the boxplots refer to the data collection's region (North = violet, South = blue, West = green, East = orange, Israel = dark red). Red lines denote LOQ/LOD for the respective study. Since percentiles are only available and displayed when they are above LOQ/LOD the lower side of boxplots might be truncated. The age categories are shown in capital letters: children of the age groups 3-5 and 6-11 (C), adolescents between 12 and 19 years (T) and adults from the age groups 20-39, 40-59 and 60 years and older (A). The sampling method is indicated in brackets: first morning urine void (mU), spot urine (sU) and 24-hours urine (24hU).

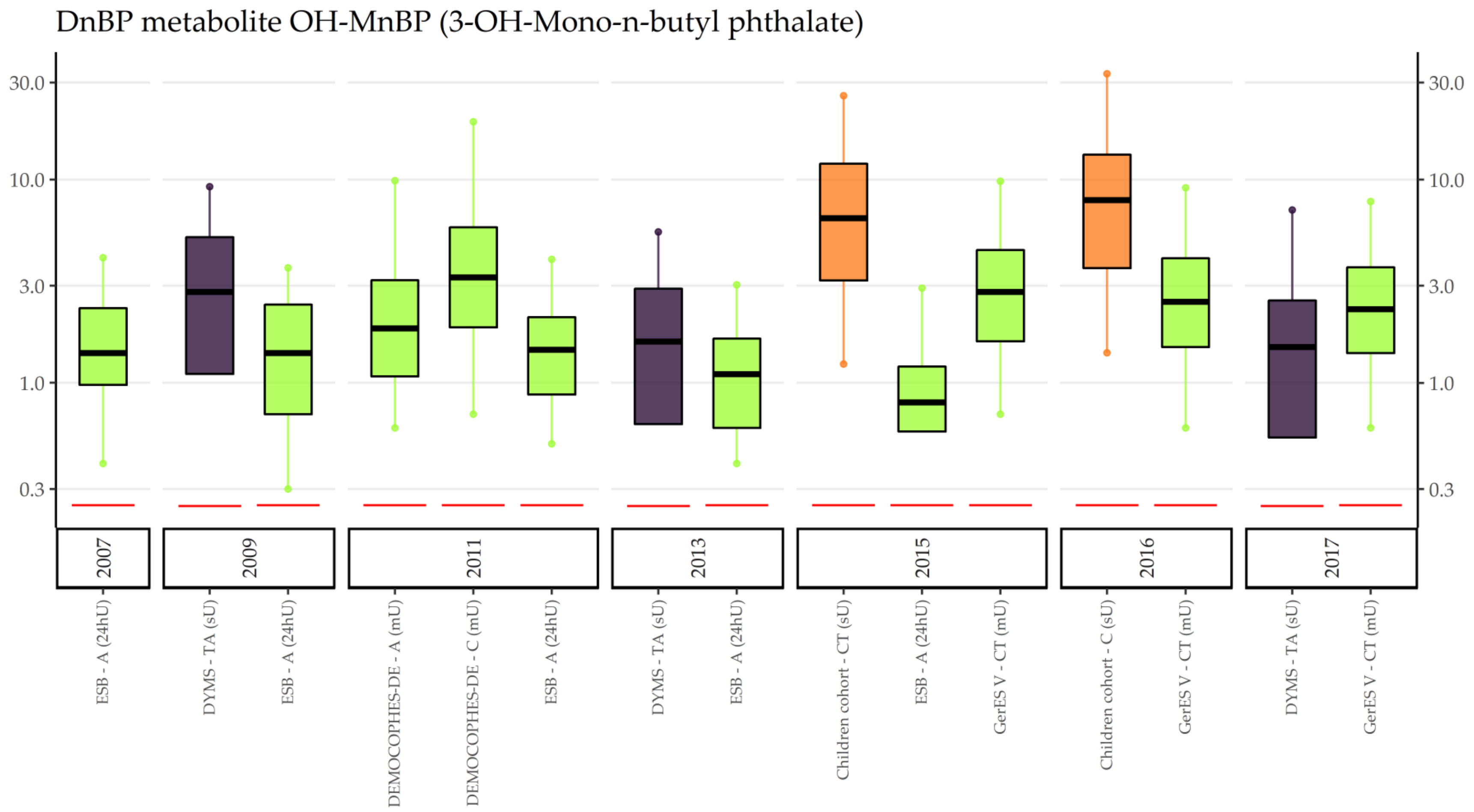

Figure S17. DnBP metabolite OH-MnBP (3-OH-Mono-n-butyl phthalate) concentration in µg/L stratified by sampling year in boxplots.

The box is based on P25, P50, and P75, and the whiskers are based on P5 and P95. Within sampling years plots are sorted by country. Colors of the boxplots refer to the data collection's region (North = violet, South = blue, West = green, East = orange, Israel = dark red). Red lines denote LOQ/LOD for the respective study. Since percentiles are only available and displayed when they are above LOQ/LOD the lower side of boxplots might be truncated. The age categories are shown in capital letters: children of the age groups 3-5 and 6-11 (C), adolescents between 12 and 19 years (T) and adults from the age groups 20-39, 40-59 and 60 years and older (A). The sampling method is indicated in brackets: first morning urine void (mU), spot urine (sU) and 24-hours urine (24hU).

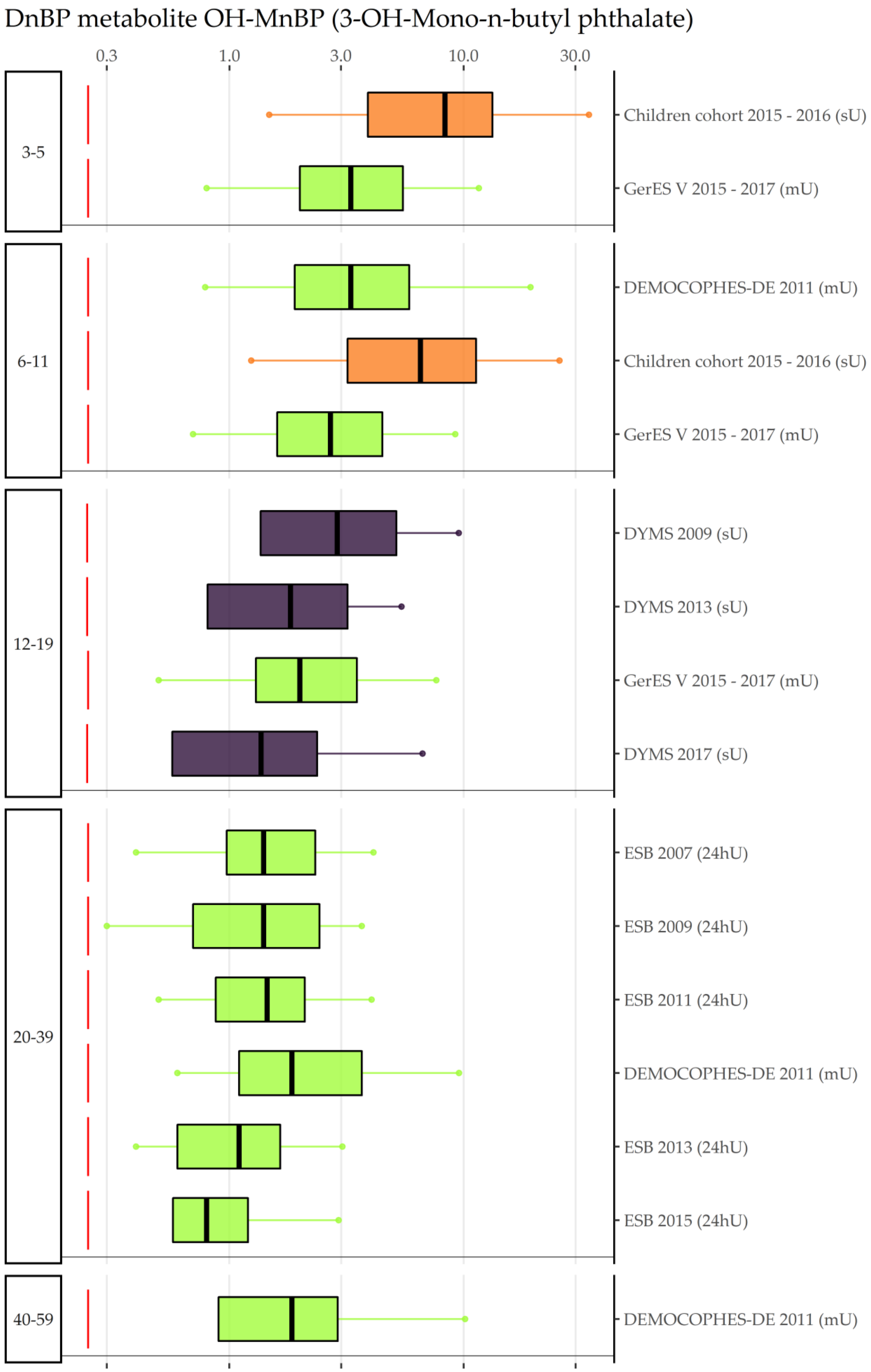

Figure S18. DnBP metabolite OH-MnBP (3-OH-Mono-n-butyl phthalate) concentration in µg/L stratified by age groups in boxplots.

The box is based on P25, P50, and P75, and the whiskers are based on P5 and P95. Within age groups plots are chronologically sorted by year of sampling. Colors of the boxplots refer to the data collection's region (North = violet, South = blue, West = green, East = orange, Israel = dark red). Red lines denote LOQ/LOD for the respective study. Since percentiles are only available and displayed when they are above LOQ/LOD the lower side of boxplots might be truncated. The sampling method is indicated in brackets: first morning urine void (mU), spot urine (sU) and 24-hours urine (24hU).

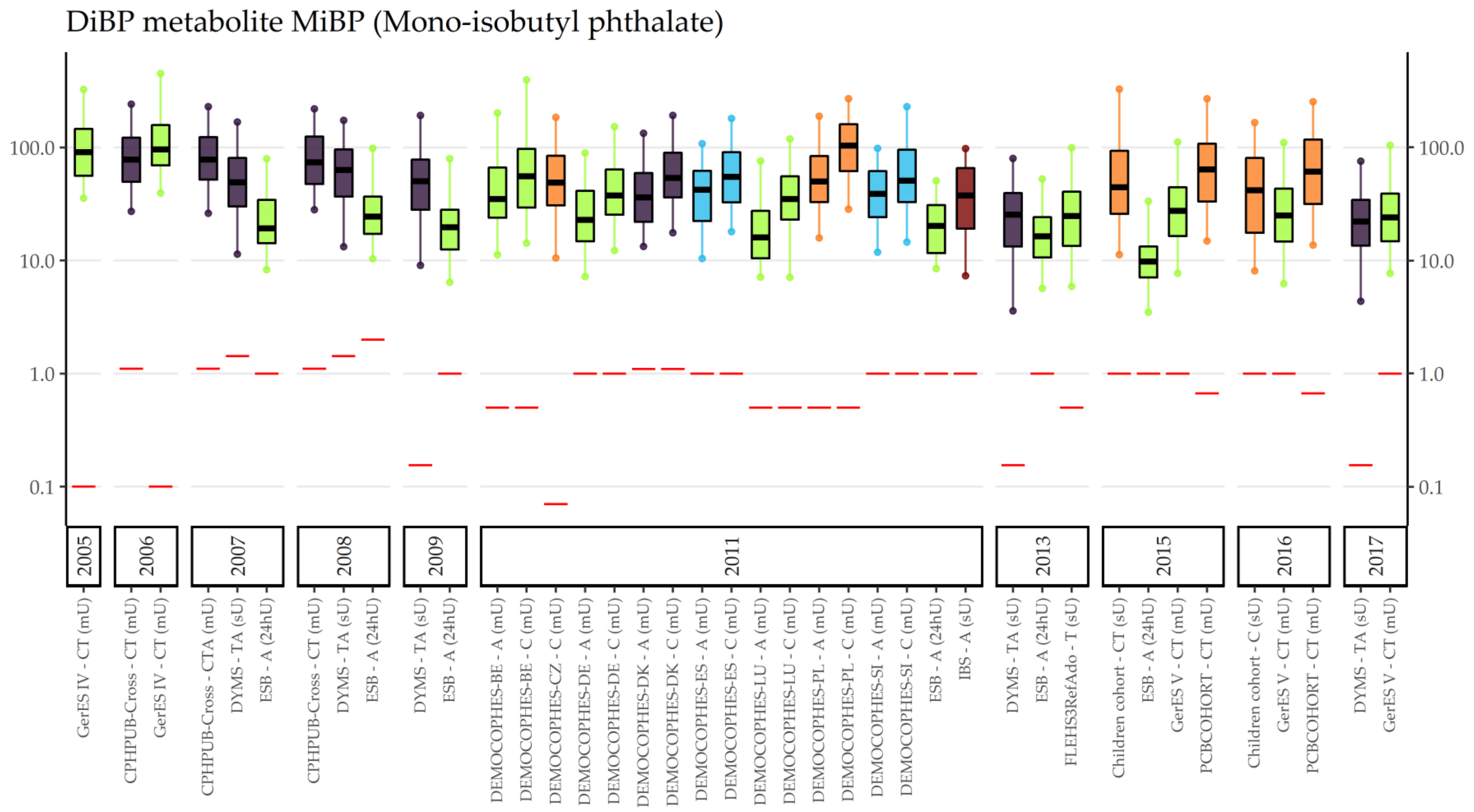

Figure S19. DiBP metabolite MiBP (Mono-isobutyl phthalate) concentration in µg/L stratified by sampling year in boxplots.

The box is based on P25, P50, and P75, and the whiskers are based on P5 and P95. Within sampling years plots are sorted by country. Colors of the boxplots refer to the data collection's region (North = violet, South = blue, West = green, East = orange, Israel = dark red). Red lines denote LOQ/LOD for the respective study. Since percentiles are only available and displayed when they are above LOQ/LOD the lower side of boxplots might be truncated. The age categories are shown in capital letters: children of the age groups 3-5 and 6-11 (C), adolescents between 12 and 19 years (T) and adults from the age groups 20-39, 40-59 and 60 years and older (A). The sampling method is indicated in brackets: first morning urine void (mU), spot urine (sU) and 24-hours urine (24hU).

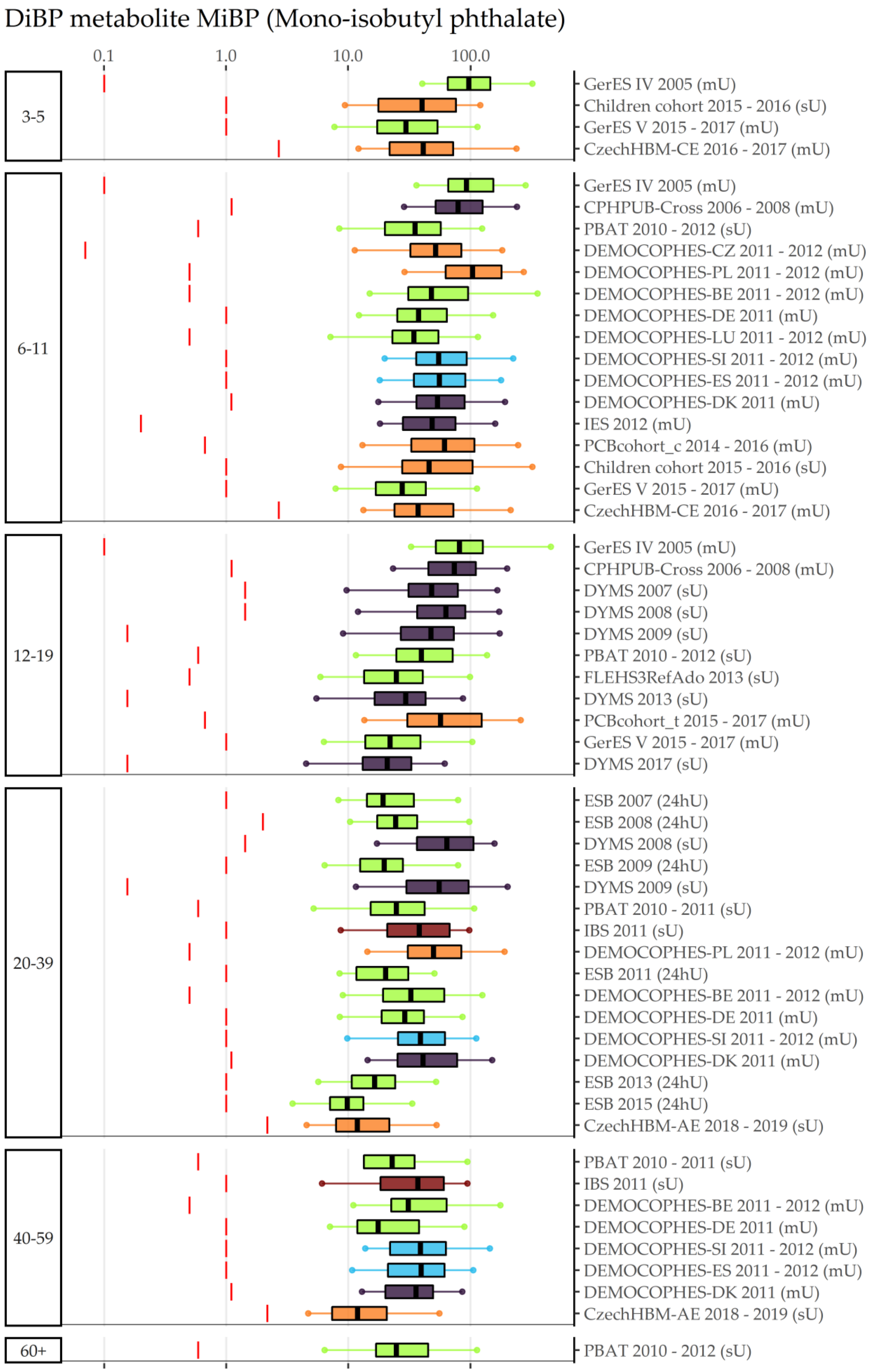

Figure S20. DiBP metabolite MiBP (Mono-isobutyl phthalate) concentration in µg/L stratified by age groups in boxplots.

The box is based on P25, P50, and P75, and the whiskers are based on P5 and P95. Within age groups plots are chronologically sorted by year of sampling. Colors of the boxplots refer to the data collection's region (North = violet, South = blue, West = green, East = orange, Israel = dark red). Red lines denote LOQ/LOD for the respective study. Since percentiles are only available and displayed when they are above LOQ/LOD the lower side of boxplots might be truncated. The sampling method is indicated in brackets: first morning urine void (mU), spot urine (sU) and 24-hours urine (24hU).

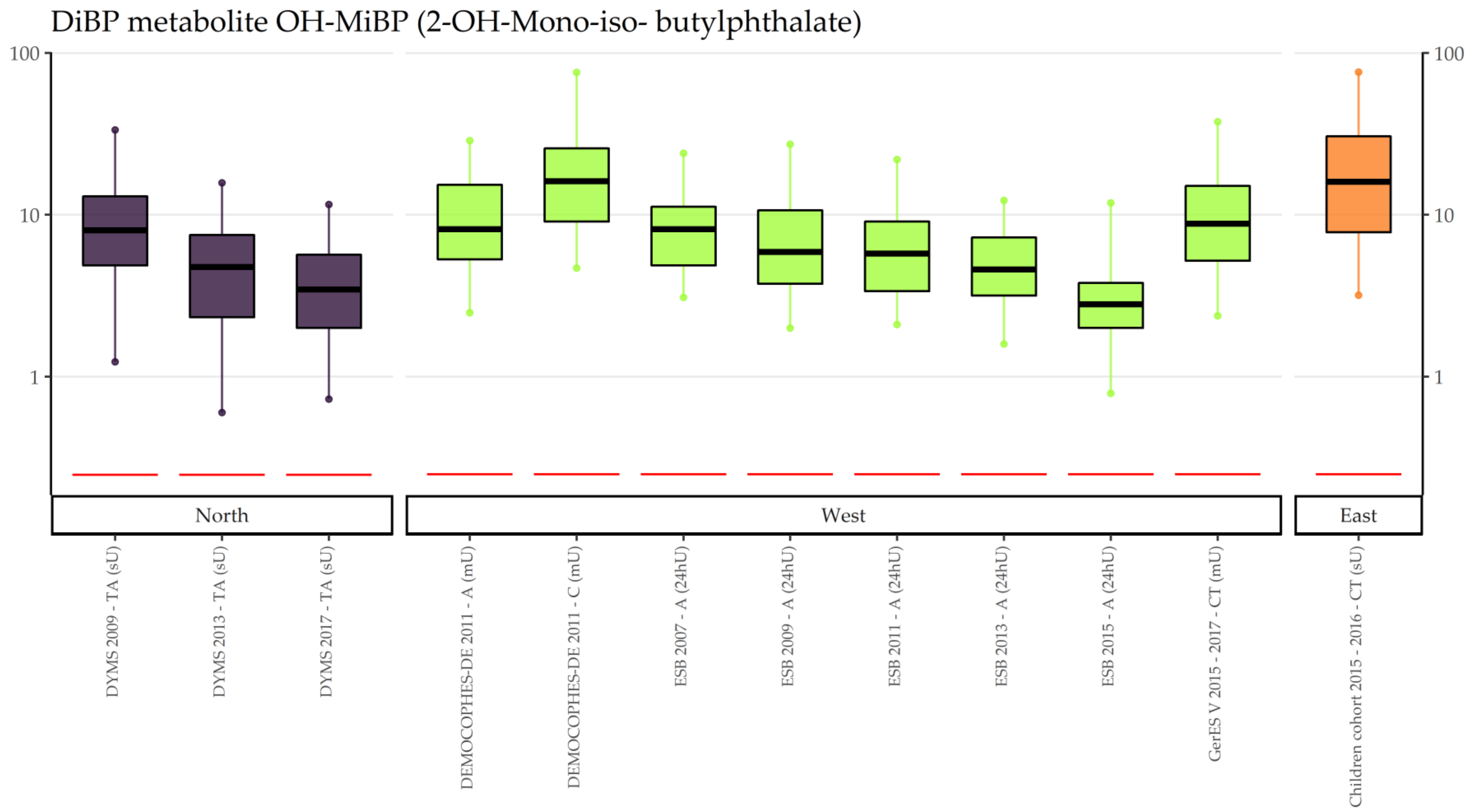

Figure S21. DiBP metabolite OH-MiBP (2-OH-Mono-iso- butylphthalate) concentration in µg/L stratified by region in boxplots.

The box is based on P25, P50, and P75, and the whiskers are based on P5 and P95. Within regions plots are sorted by country and year of sampling. Colors of the boxplots refer to the data collection's region (North = violet, South = blue, West = green, East = orange, Israel = dark red). Red lines denote LOQ/LOD for the respective study. Since percentiles are only available and displayed when they are above LOQ/LOD the lower side of boxplots might be truncated. The age categories are shown in capital letters: children of the age groups 3-5 and 6-11 (C), adolescents between 12 and 19 years (T) and adults from the age groups 20-39, 40-59 and 60 years and older (A). The sampling method is indicated in brackets: first morning urine void (mU), spot urine (sU) and 24-hours urine (24hU).

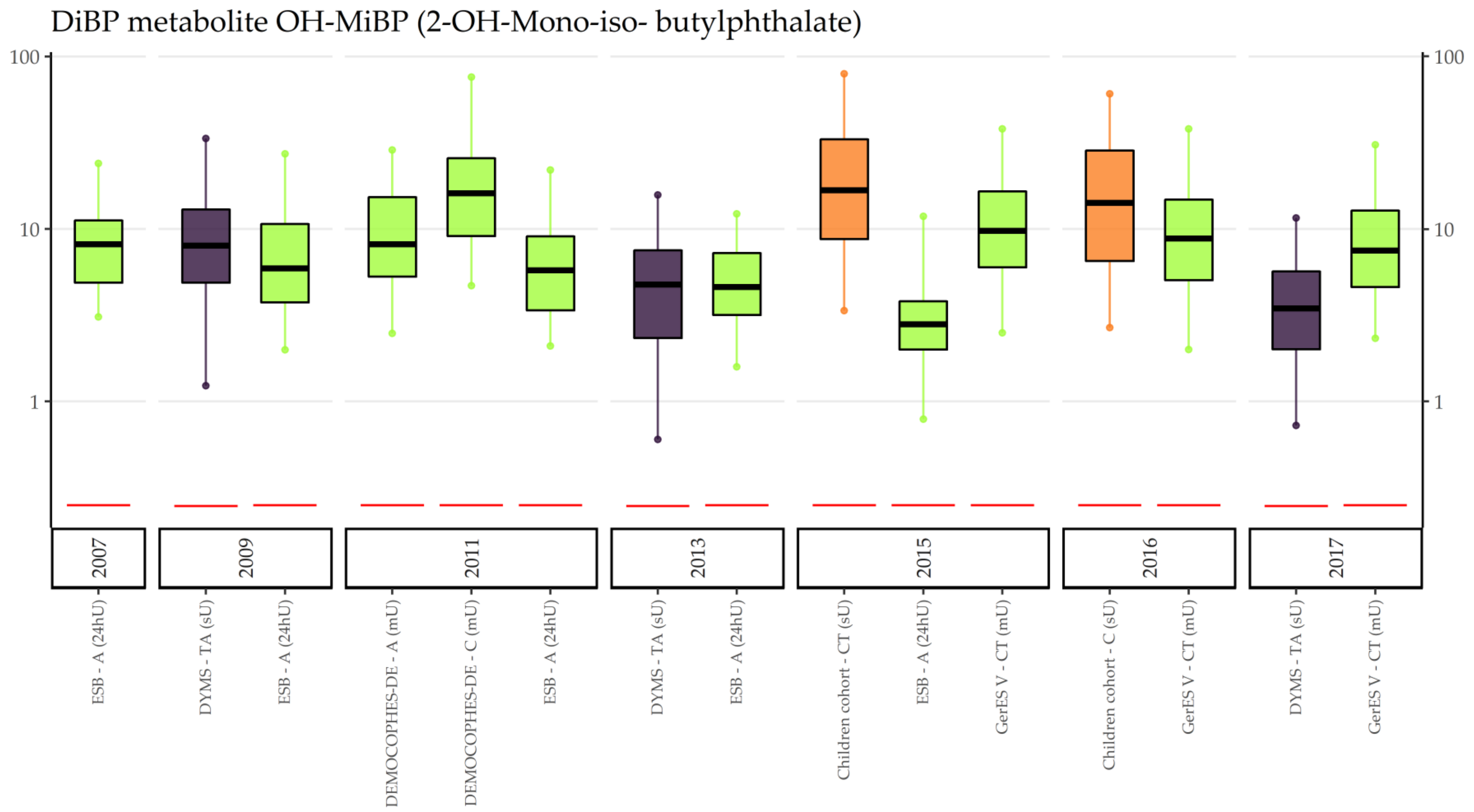

Figure S22. DiBP metabolite OH-MiBP (2-OH-Mono-iso- butylphthalate) concentration in µg/L stratified by sampling year in boxplots.

The box is based on P25, P50, and P75, and the whiskers are based on P5 and P95. Within sampling years plots are sorted by country. Colors of the boxplots refer to the data collection's region (North = violet, South = blue, West = green, East = orange, Israel = dark red). Red lines denote LOQ/LOD for the respective study. Since percentiles are only available and displayed when they are above LOQ/LOD the lower side of boxplots might be truncated. The age categories are shown in capital letters: children of the age groups 3-5 and 6-11 (C), adolescents between 12 and 19 years (T) and adults from the age groups 20-39, 40-59 and 60 years and older (A). The sampling method is indicated in brackets: first morning urine void (mU), spot urine (sU) and 24-hours urine (24hU).

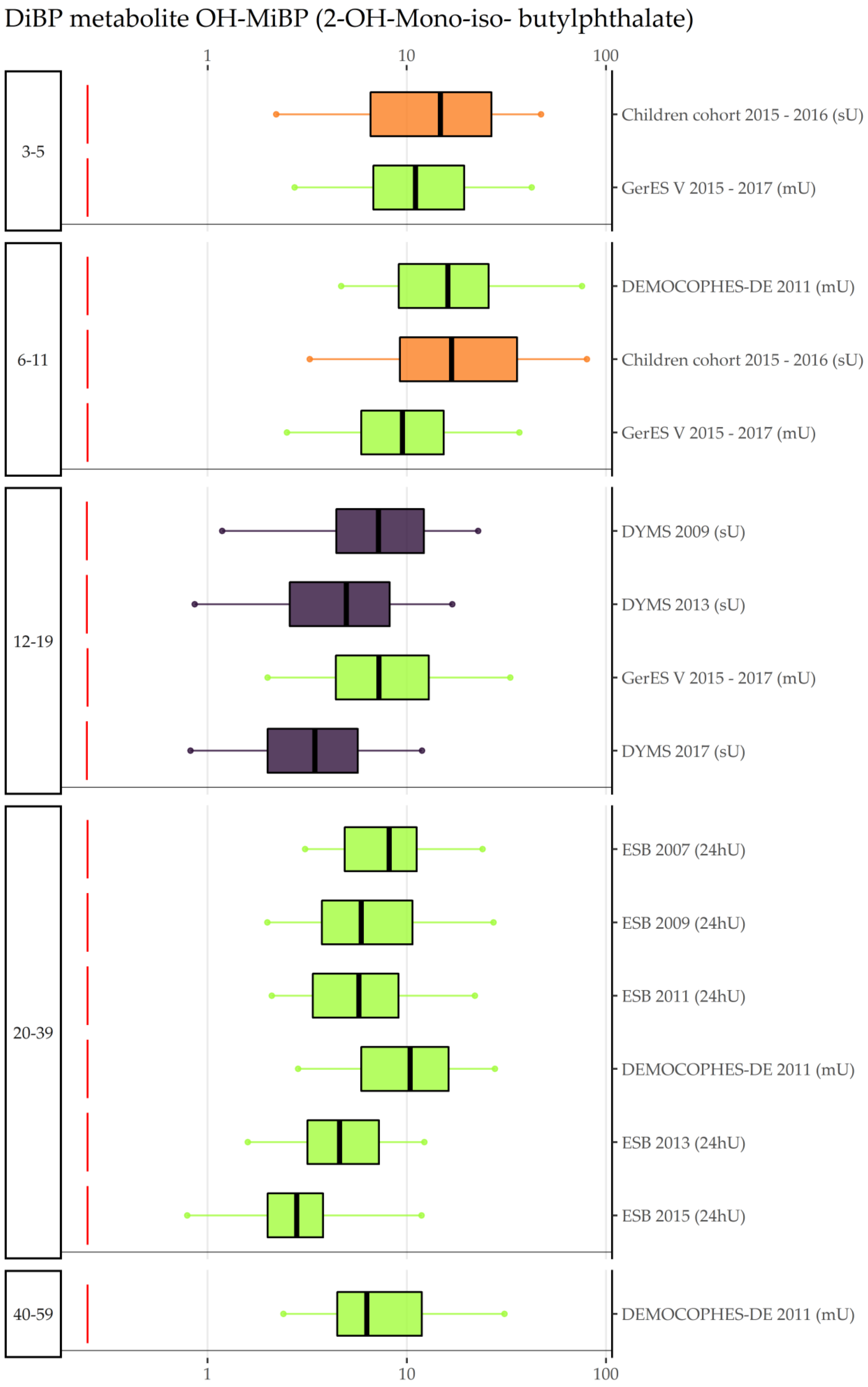

Figure S23. DiBP metabolite OH-MiBP (2-OH-Mono-iso- butylphthalate) concentration in µg/L stratified by age groups in boxplots.

The box is based on P25, P50, and P75, and the whiskers are based on P5 and P95. Within age groups plots are chronologically sorted by year of sampling. Colors of the boxplots refer to the data collection's region (North = violet, South = blue, West = green, East = orange, Israel = dark red). Red lines denote LOQ/LOD for the respective study. Since percentiles are only available and displayed when they are above LOQ/LOD the lower side of boxplots might be truncated. The sampling method is indicated in brackets: first morning urine void (mU), spot urine (sU) and 24-hours urine (24hU).

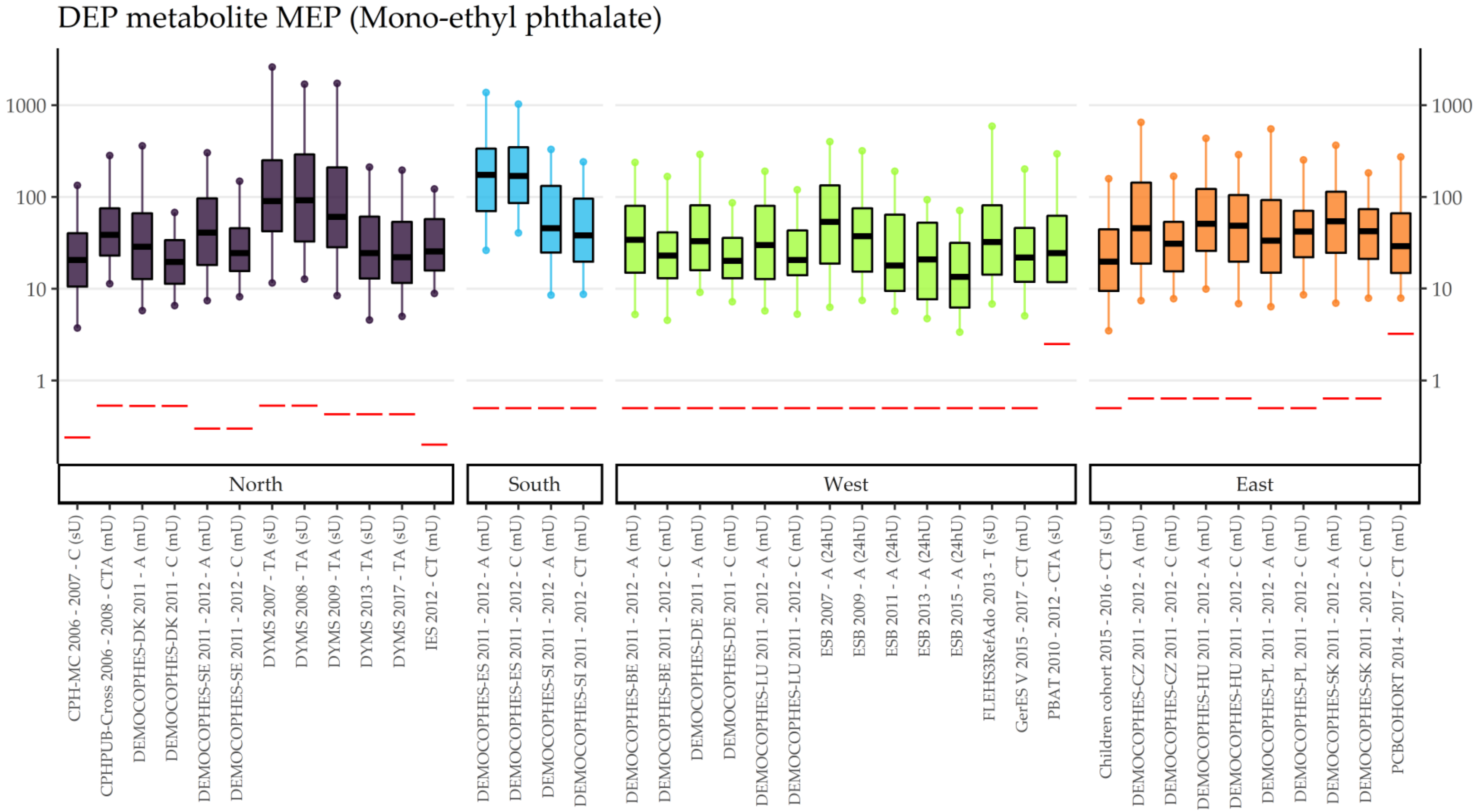

Figure S24. DEP metabolite MEP (Mono-ethyl phthalate) concentration in µg/L stratified by region in boxplots.

The box is based on P25, P50, and P75, and the whiskers are based on P5 and P95. Within regions plots are sorted by country and year of sampling. Colors of the boxplots refer to the data collection's region (North = violet, South = blue, West = green, East = orange, Israel = dark red). Red lines denote LOQ/LOD for the respective study. Since percentiles are only available and displayed when they are above LOQ/LOD the lower side of boxplots might be truncated. The age categories are shown in capital letters: children of the age groups 3-5 and 6-11 (C), adolescents between 12 and 19 years (T) and adults from the age groups 20-39, 40-59 and 60 years and older (A). The sampling method is indicated in brackets: first morning urine void (mU), spot urine (sU) and 24-hours urine (24hU).

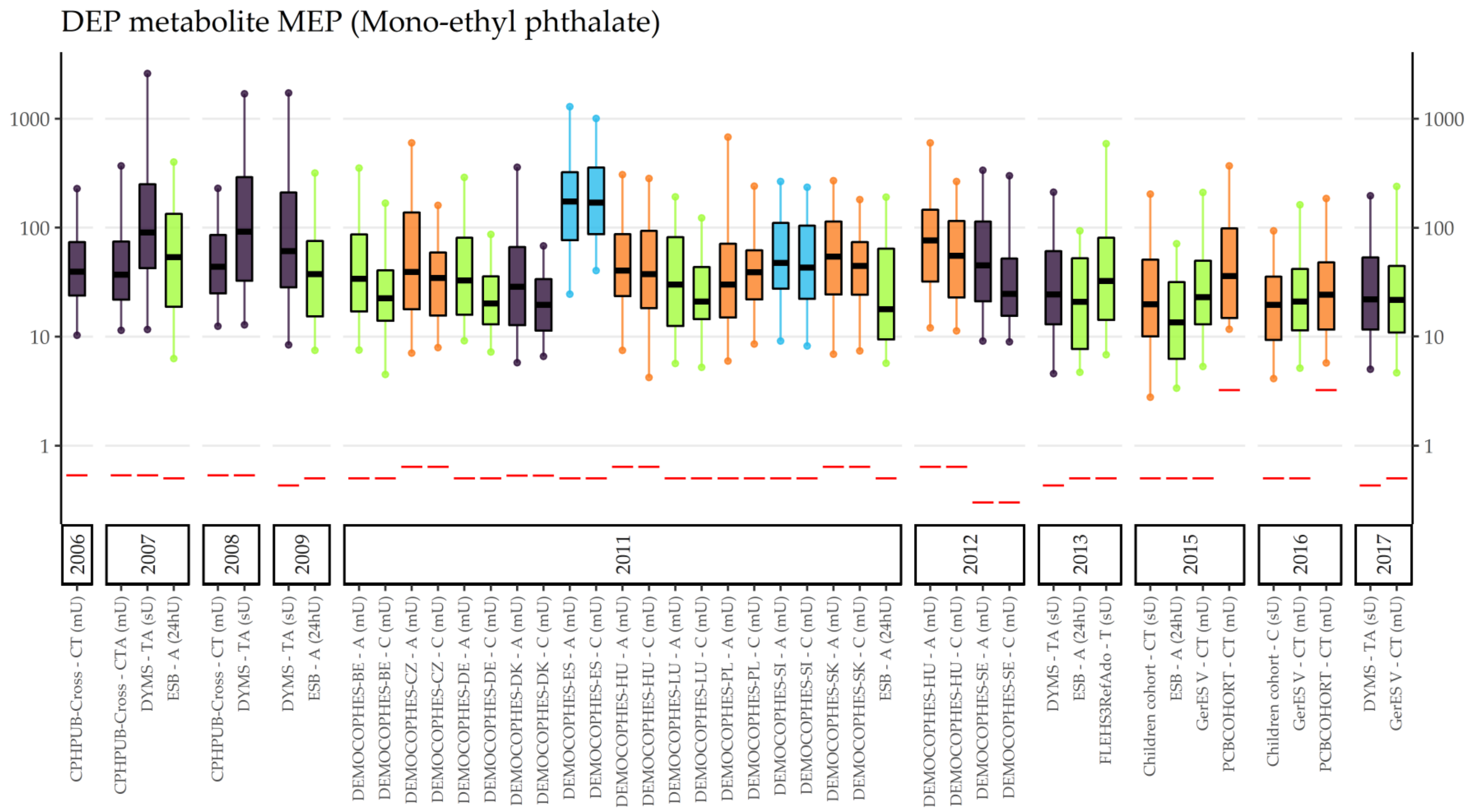

Figure S25. DEP metabolite MEP (Mono-ethyl phthalate) concentration in µg/L stratified by sampling year in boxplots.

The box is based on P25, P50, and P75, and the whiskers are based on P5 and P95. Within sampling years plots are sorted by country. Colors of the boxplots refer to the data collection's region (North = violet, South = blue, West = green, East = orange, Israel = dark red). Red lines denote LOQ/LOD for the respective study. Since percentiles are only available and displayed when they are above LOQ/LOD the lower side of boxplots might be truncated. The age categories are shown in capital letters: children of the age groups 3-5 and 6-11 (C), adolescents between 12 and 19 years (T) and adults from the age groups 20-39, 40-59 and 60 years and older (A). The sampling method is indicated in brackets: first morning urine void (mU), spot urine (sU) and 24-hours urine (24hU).

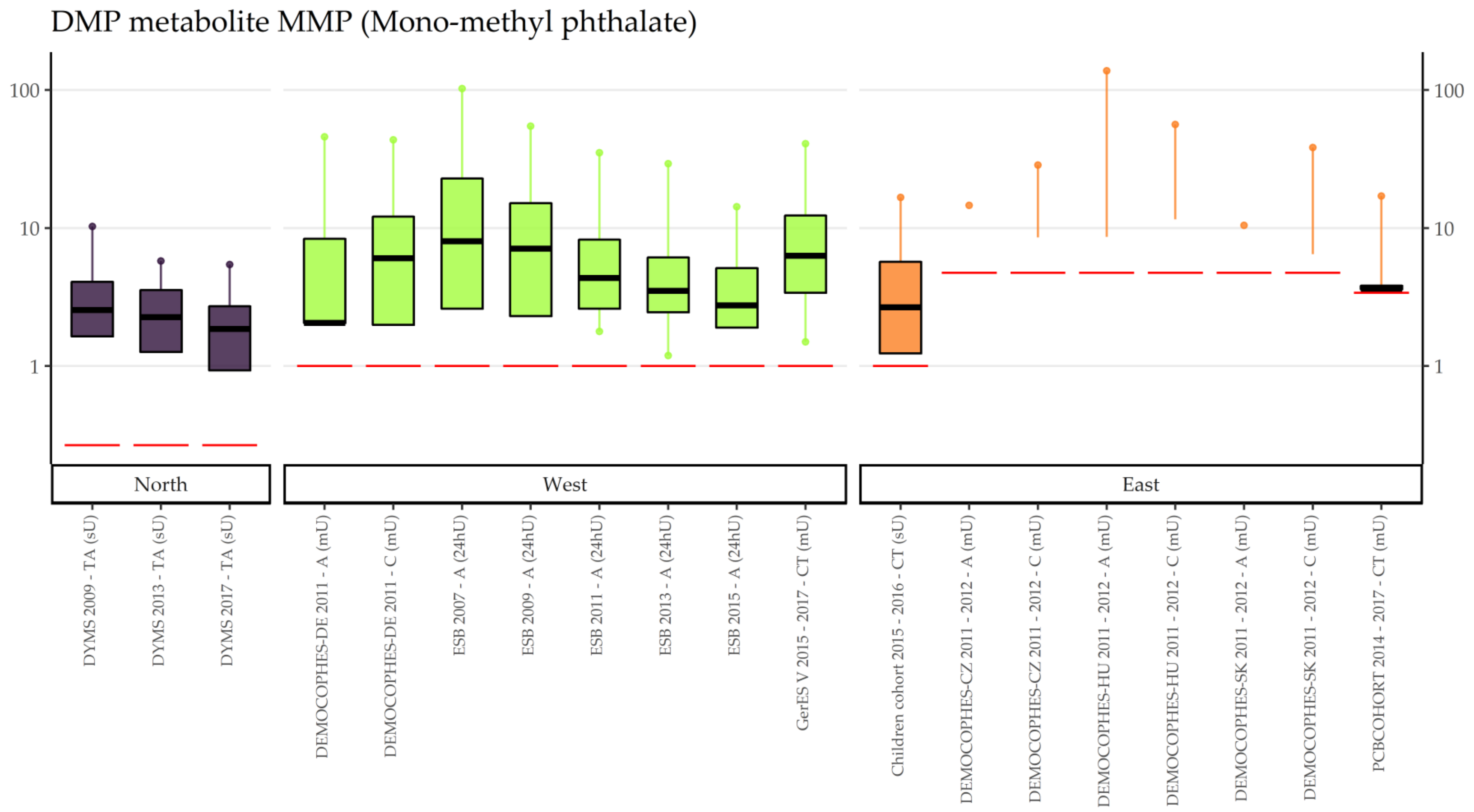

Figure S26. DMP metabolite MMP (Mono-methyl phthalate) concentration in µg/L stratified by region in boxplots.

The box is based on P25, P50, and P75, and the whiskers are based on P5 and P95. Within regions plots are sorted by country and year of sampling. Colors of the boxplots refer to the data collection's region (North = violet, South = blue, West = green, East = orange, Israel = dark red). Red lines denote LOQ/LOD for the respective study. Since percentiles are only available and displayed when they are above LOQ/LOD the lower side of boxplots might be truncated. The age categories are shown in capital letters: children of the age groups 3-5 and 6-11 (C), adolescents between 12 and 19 years (T) and adults from the age groups 20-39, 40-59 and 60 years and older (A). The sampling method is indicated in brackets: first morning urine void (mU), spot urine (sU) and 24-hours urine (24hU).

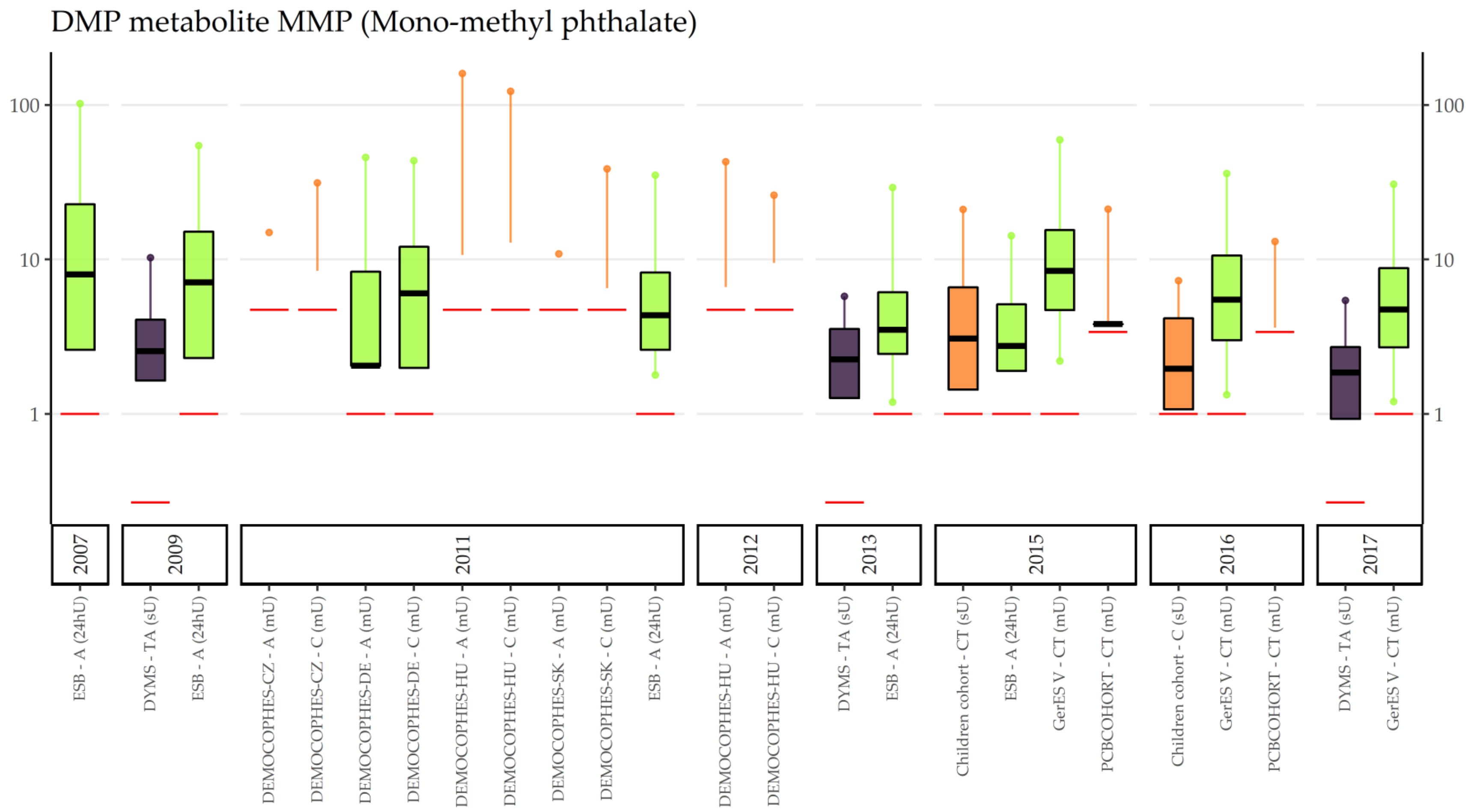

Figure S27. DMP metabolite MMP (Mono-methyl phthalate) concentration in µg/L stratified by sampling year in boxplots.

The box is based on P25, P50, and P75, and the whiskers are based on P5 and P95. Within sampling years plots are sorted by country. Colors of the boxplots refer to the data collection's region (North = violet, South = blue, West = green, East = orange, Israel = dark red). Red lines denote LOQ/LOD for the respective study. Since percentiles are only available and displayed when they are above LOQ/LOD the lower side of boxplots might be truncated. The age categories are shown in capital letters: children of the age groups 3-5 and 6-11 (C), adolescents between 12 and 19 years (T) and adults from the age groups 20-39, 40-59 and 60 years and older (A). The sampling method is indicated in brackets: first morning urine void (mU), spot urine (sU) and 24-hours urine (24hU).

DMP metabolite MMP (Mono-methyl phthalate)

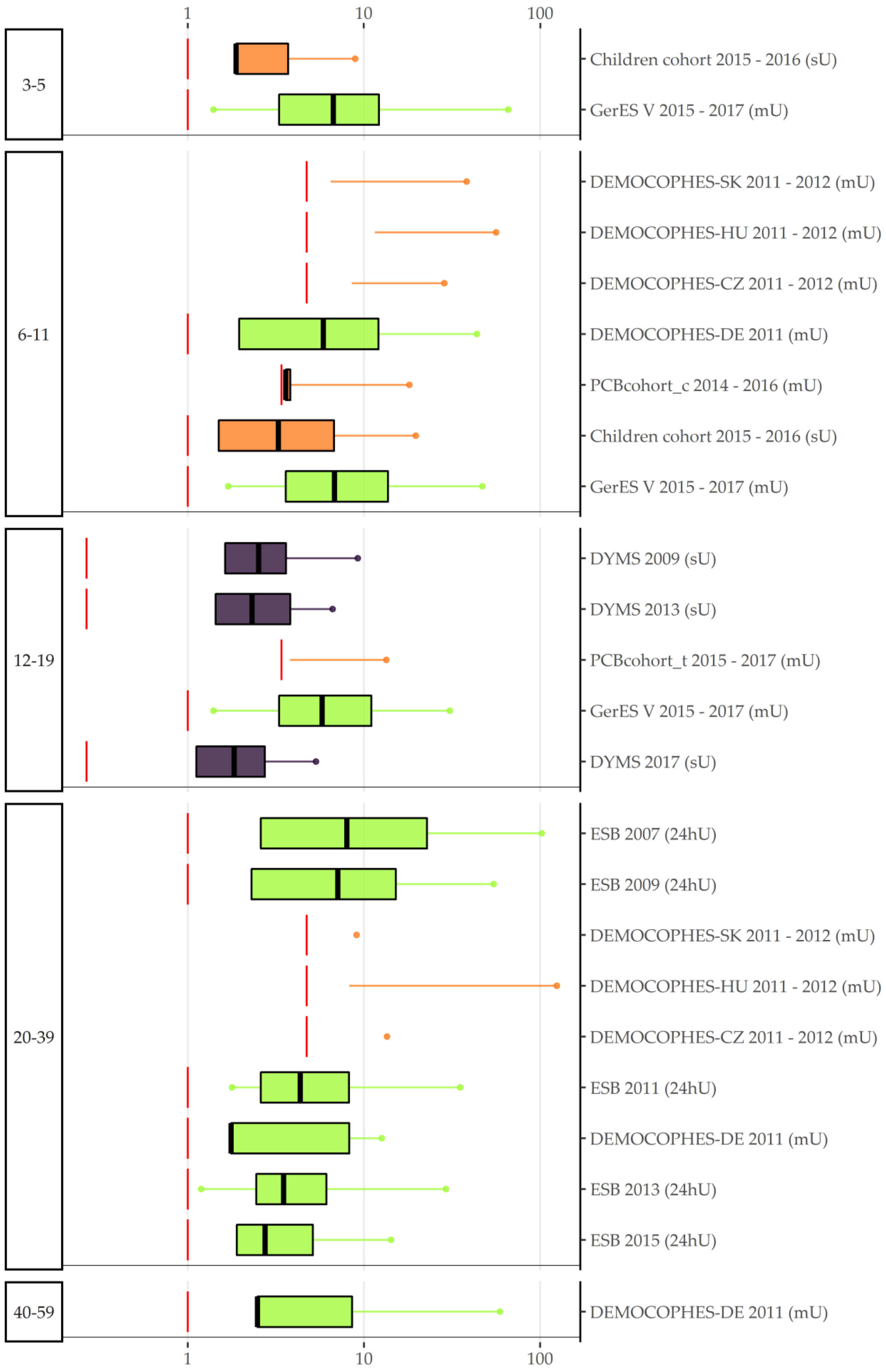

Figure S28. DMP metabolite MMP (Mono-methyl phthalate) concentration in µg/L stratified by age groups in boxplots.

The box is based on P25, P50, and P75, and the whiskers are based on P5 and P95. Within age groups plots are chronologically sorted by year of sampling. Colors of the boxplots refer to the data collection's region (North = violet, South = blue, West = green, East = orange, Israel = dark red). Red lines denote LOQ/LOD for the respective study. Since percentiles are only available and displayed when they are above LOQ/LOD the lower side of boxplots might be truncated. The sampling method is indicated in brackets: first morning urine void (mU), spot urine (sU) and 24-hours urine (24hU).

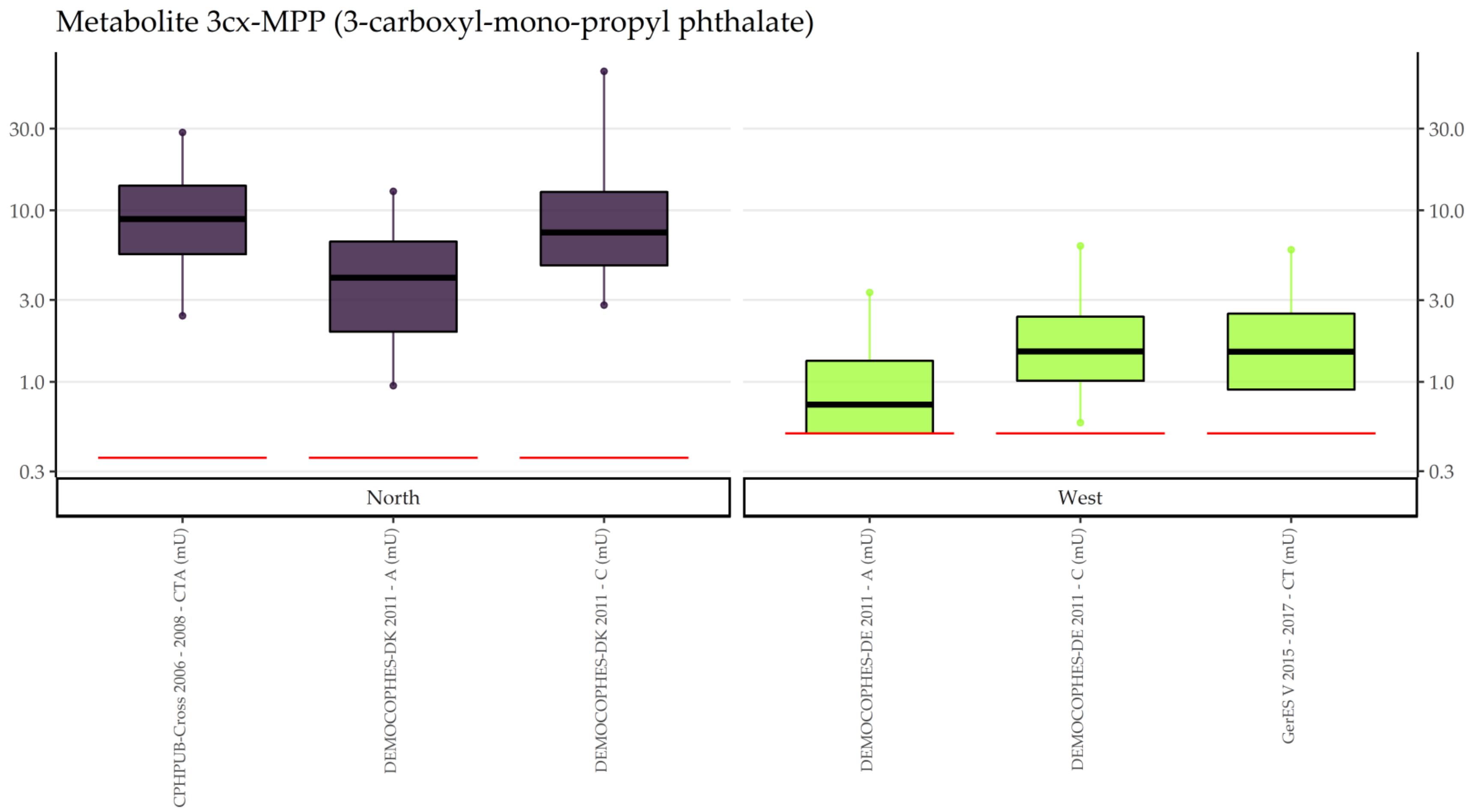

Figure S29. Metabolite 3cx-MPP (3-carboxyl-mono-propyl phthalate) concentration in µg/L stratified by region in boxplots.

The box is based on P25, P50, and P75, and the whiskers are based on P5 and P95. Within regions plots are sorted by country and year of sampling. Colors of the boxplots refer to the data collection's region (North = violet, South = blue, West = green, East = orange, Israel = dark red). Red lines denote LOQ/LOD for the respective study. Since percentiles are only available and displayed when they are above LOQ/LOD the lower side of boxplots might be truncated. The age categories are shown in capital letters: children of the age groups 3-5 and 6-11 (C), adolescents between 12 and 19 years (T) and adults from the age groups 20-39, 40-59 and 60 years and older (A). The sampling method is indicated in brackets: first morning urine void (mU), spot urine (sU) and 24-hours urine (24hU).

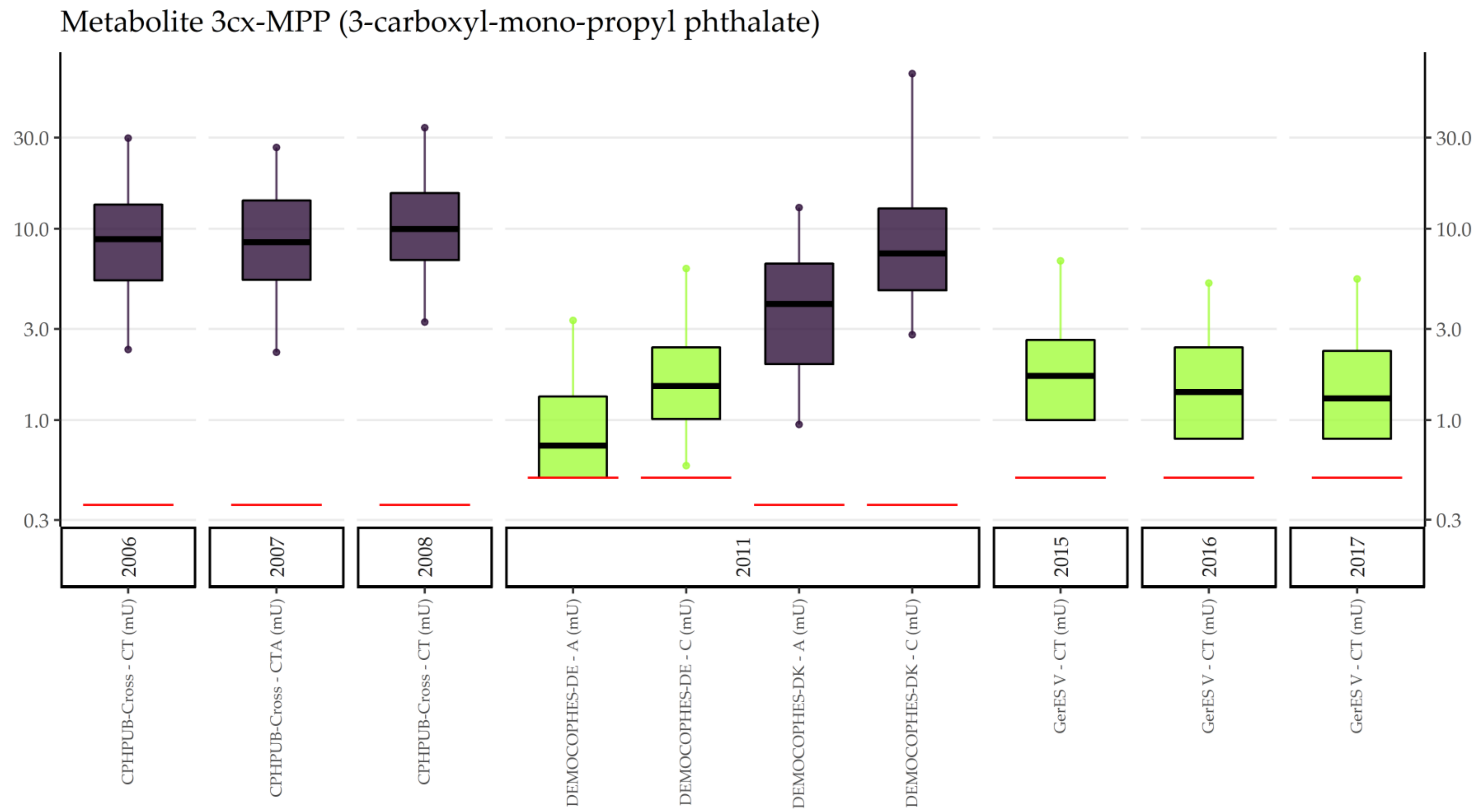

Figure S30. Metabolite 3cx-MPP (3-carboxyl-mono-propyl phthalate) concentration in µg/L stratified by sampling year in boxplots.

The box is based on P25, P50, and P75, and the whiskers are based on P5 and P95. Within sampling years plots are sorted by country. Colors of the boxplots refer to the data collection's region (North = violet, South = blue, West = green, East = orange, Israel = dark red). Red lines denote LOQ/LOD for the respective study. Since percentiles are only available and displayed when they are above LOQ/LOD the lower side of boxplots might be truncated. The age categories are shown in capital letters: children of the age groups 3-5 and 6-11 (C), adolescents between 12 and 19 years (T) and adults from the age groups 20-39, 40-59 and 60 years and older (A). The sampling method is indicated in brackets: first morning urine void (mU), spot urine (sU) and 24-hours urine (24hU).

Metabolite 3cx-MPP (3-carboxyl-mono-propyl phthalate)

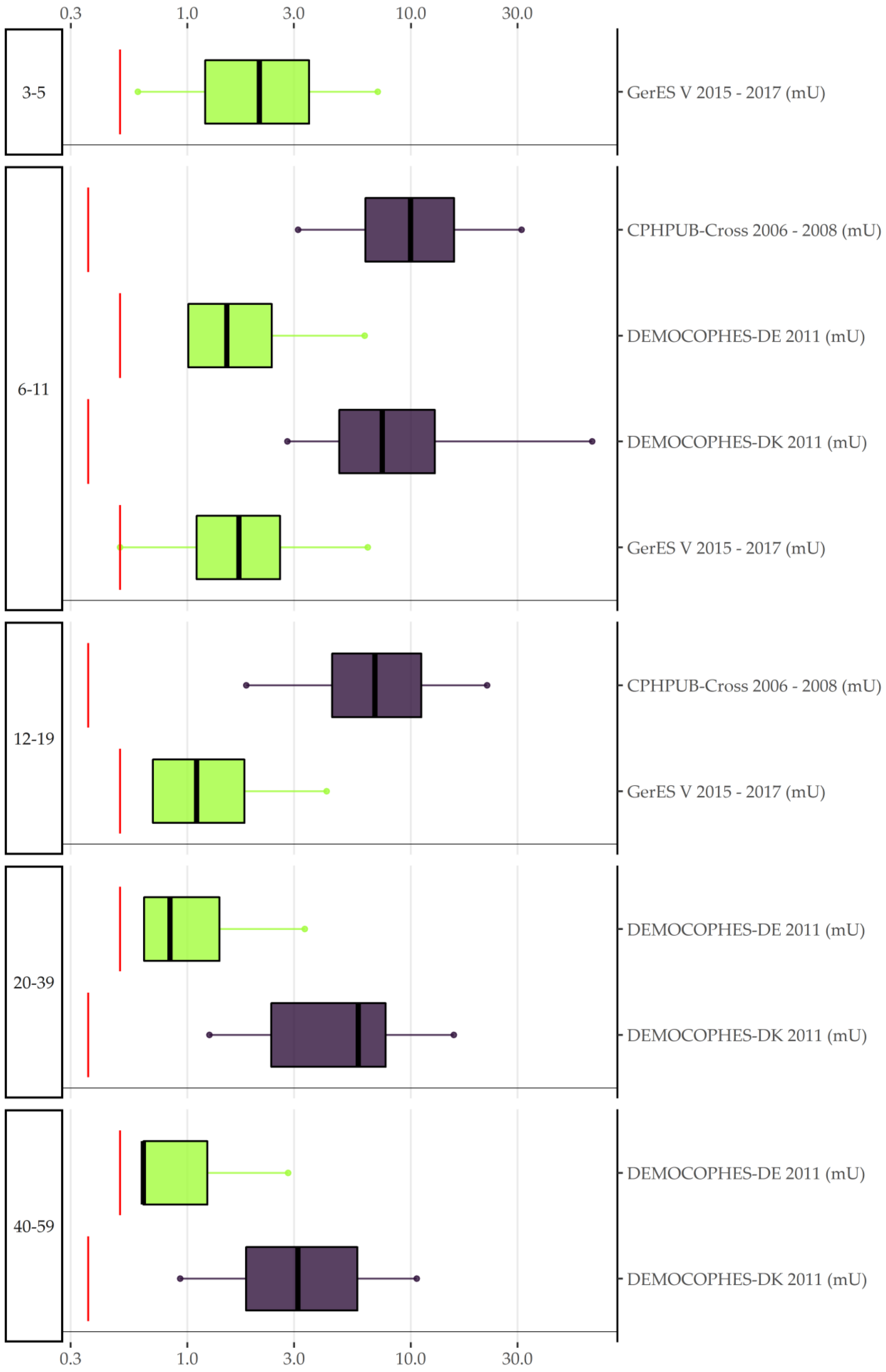

Figure S31. Metabolite 3cx-MPP (3-carboxyl-mono-propyl phthalate) concentration in µg/L stratified by age groups in boxplots.

The box is based on P25, P50, and P75, and the whiskers are based on P5 and P95. Within age groups plots are chronologically sorted by year of sampling. Colors of the boxplots refer to the data collection’s region (North = violet, South = blue, West = green, East = orange, Israel = dark red). Red lines denote LOQ/LOD for the respective study. Since percentiles are only available and displayed when they are above LOQ/LOD the lower side of boxplots might be truncated. The sampling method is indicated in brackets: first morning urine void (mU), spot urine (sU) and 24-hours urine (24hU).

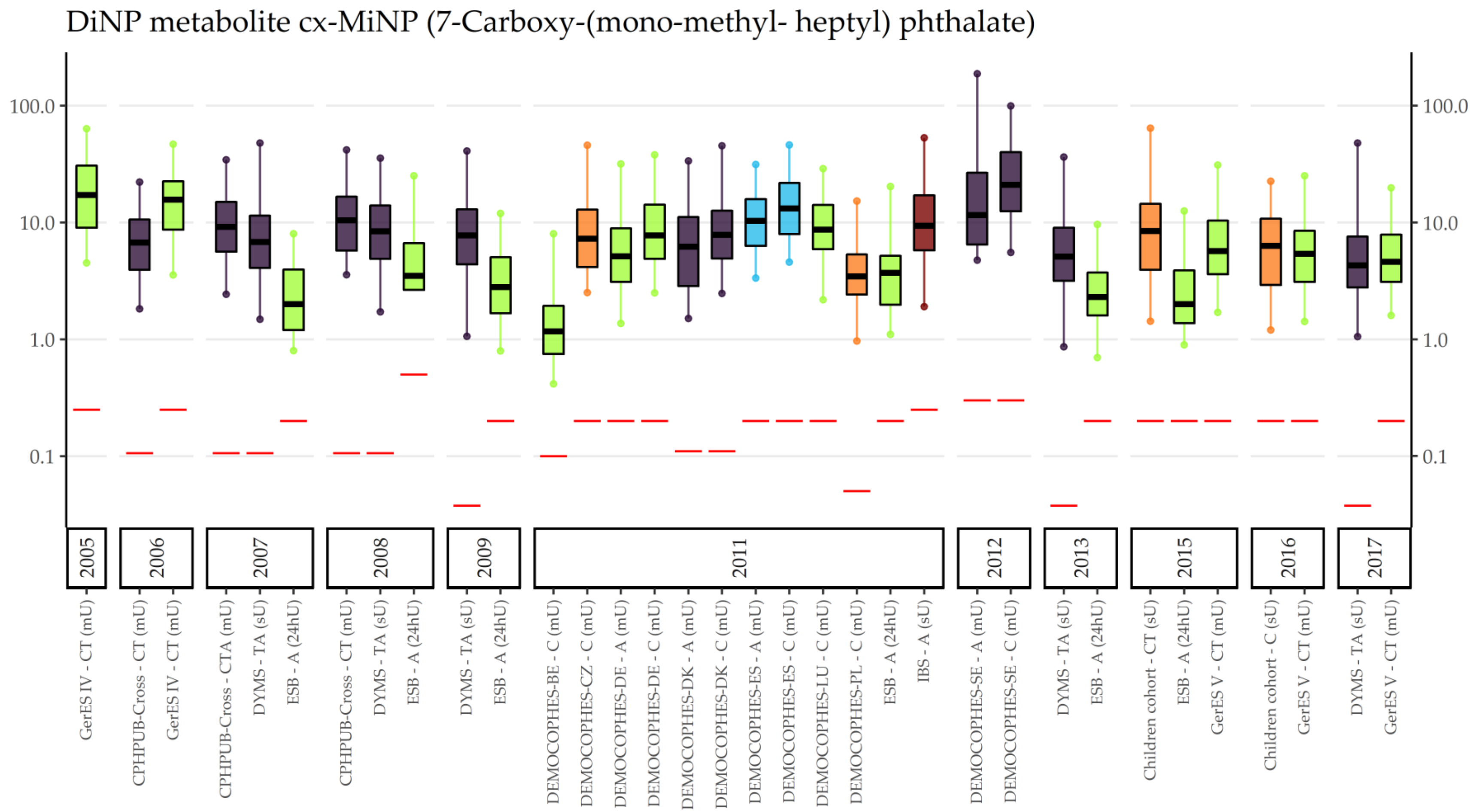

Figure S32. DiNP metabolite cx-MiNP (7-Carboxy-(mono-methyl- heptyl) phthalate) concentration in µg/L stratified by sampling year in boxplots.

The box is based on P25, P50, and P75, and the whiskers are based on P5 and P95. Within sampling years plots are sorted by country. Colors of the boxplots refer to the data collection's region (North = violet, South = blue, West = green, East = orange, Israel = dark red). Red lines denote LOQ/LOD for the respective study. Since percentiles are only available and displayed when they are above LOQ/LOD the lower side of boxplots might be truncated. The age categories are shown in capital letters: children of the age groups 3-5 and 6-11 (C), adolescents between 12 and 19 years (T) and adults from the age groups 20-39, 40-59 and 60 years and older (A). The sampling method is indicated in brackets: first morning urine void (mU), spot urine (sU) and 24-hours urine (24hU).

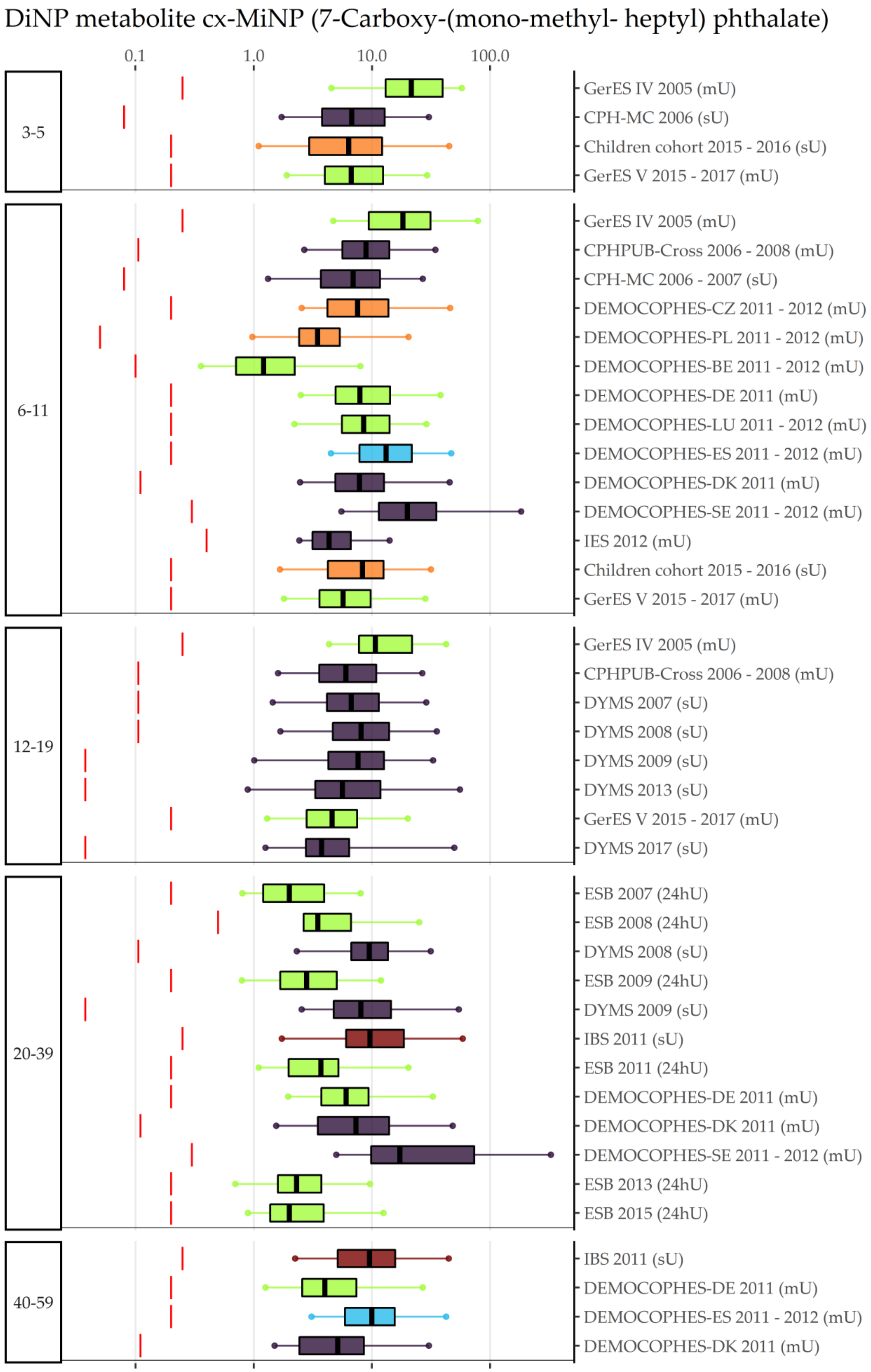

Figure S33. DiNP metabolite cx-MiNP (7-Carboxy-(mono-methyl- heptyl) phthalate) concentration in µg/L stratified by age groups in boxplots.

The box is based on P25, P50, and P75, and the whiskers are based on P5 and P95. Within age groups plots are chronologically sorted by year of sampling. Colors of the boxplots refer to the data collection's region (North = violet, South = blue, West = green, East = orange, Israel = dark red). Red lines denote LOQ/LOD for the respective study. Since percentiles are only available and displayed when they are above LOQ/LOD the lower side of boxplots might be truncated. The sampling method is indicated in brackets: first morning urine void (mU), spot urine (sU) and 24-hours urine (24hU).

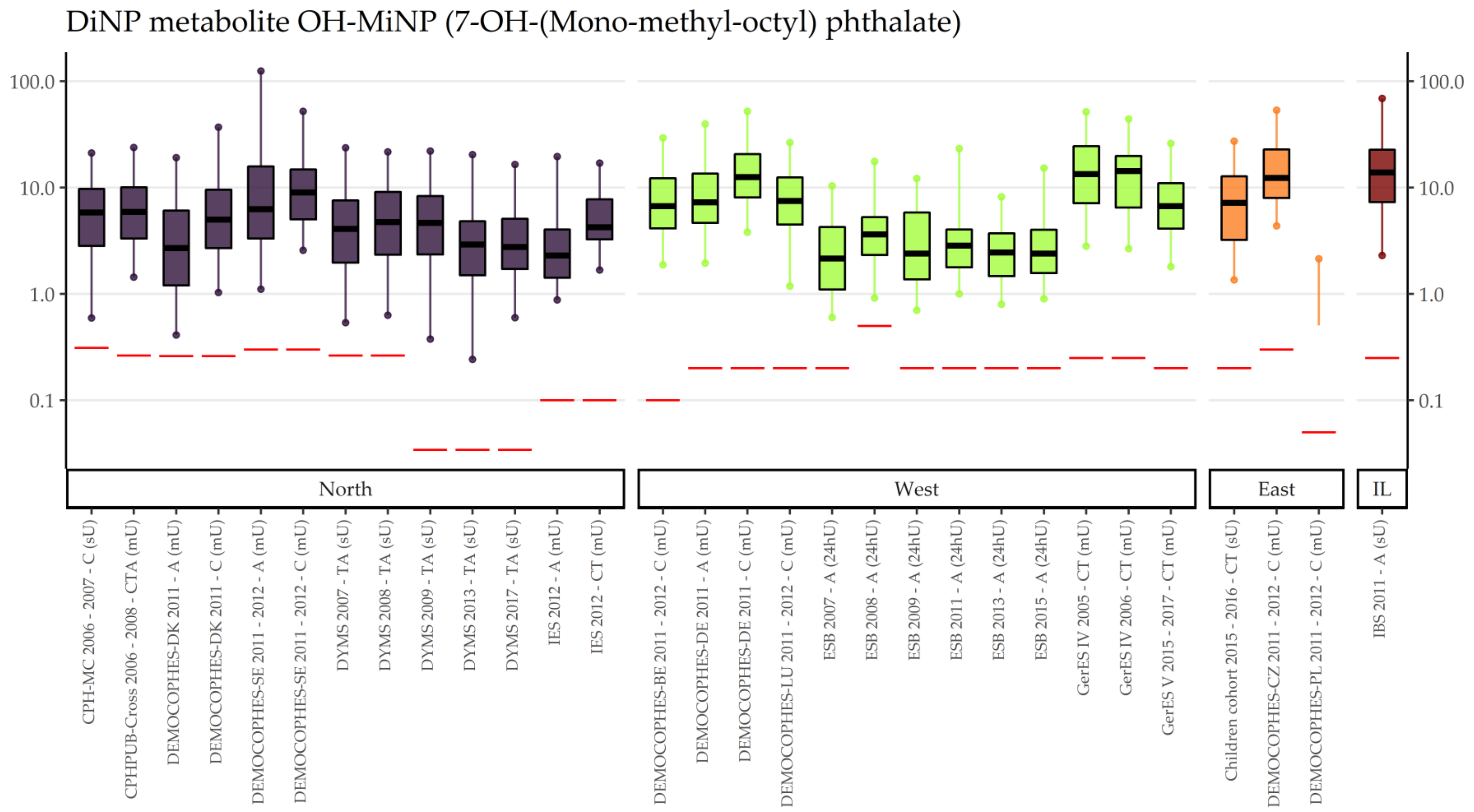

Figure S34. DiNP metabolite OH-MiNP (7-OH-(Mono-methyl-octyl) phthalate) concentration in µg/L stratified by region in boxplots.

The box is based on P25, P50, and P75, and the whiskers are based on P5 and P95. Within regions plots are sorted by country and year of sampling. Colors of the boxplots refer to the data collection's region (North = violet, South = blue, West = green, East = orange, Israel = dark red). Red lines denote LOQ/LOD for the respective study. Since percentiles are only available and displayed when they are above LOQ/LOD the lower side of boxplots might be truncated. The age categories are shown in capital letters: children of the age groups 3-5 and 6-11 (C), adolescents between 12 and 19 years (T) and adults from the age groups 20-39, 40-59 and 60 years and older (A). The sampling method is indicated in brackets: first morning urine void (mU), spot urine (sU) and 24-hours urine (24hU).

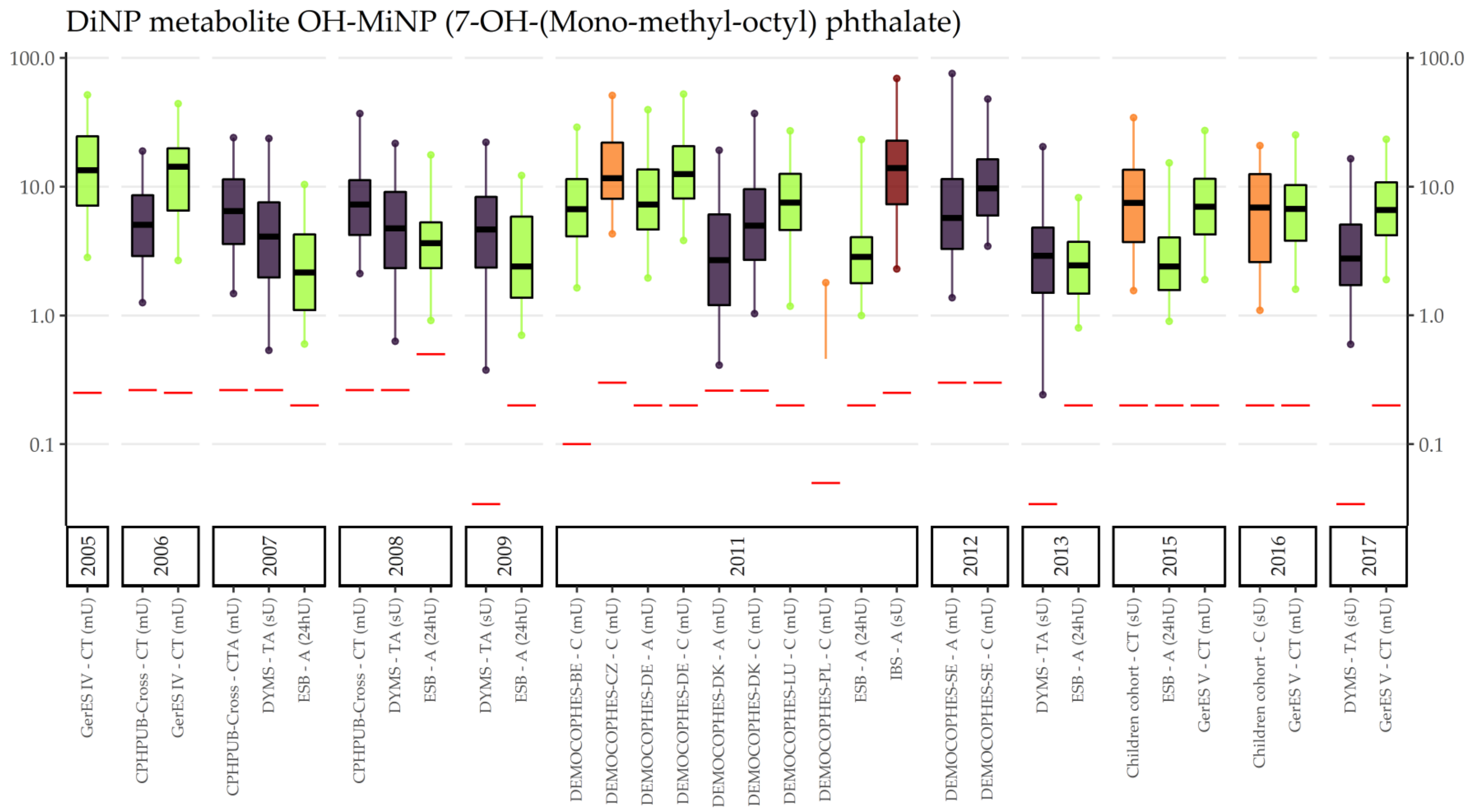

Figure S35. DiNP metabolite OH-MiNP (7-OH-(Mono-methyl-octyl) phthalate) concentration in µg/L stratified by sampling year in boxplots.

The box is based on P25, P50, and P75, and the whiskers are based on P5 and P95. Within sampling years plots are sorted by country. Colors of the boxplots refer to the data collection's region (North = violet, South = blue, West = green, East = orange, Israel = dark red). Red lines denote LOQ/LOD for the respective study. Since percentiles are only available and displayed when they are above LOQ/LOD the lower side of boxplots might be truncated. The age categories are shown in capital letters: children of the age groups 3-5 and 6-11 (C), adolescents between 12 and 19 years (T) and adults from the age groups 20-39, 40-59 and 60 years and older (A). The sampling method is indicated in brackets: first morning urine void (mU), spot urine (sU) and 24-hours urine (24hU).

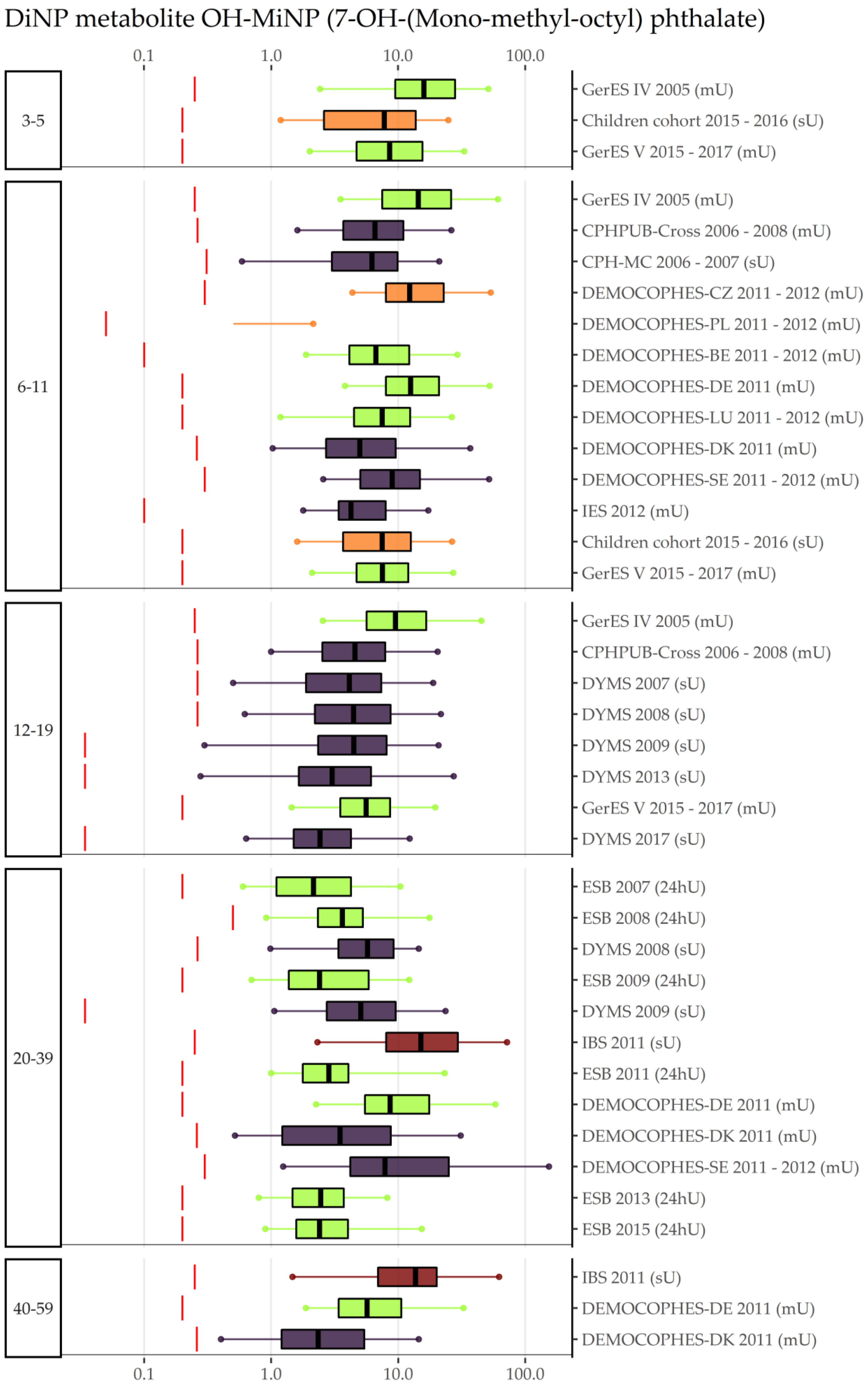

Figure S36. DiNP metabolite OH-MiNP (7-OH-(Mono-methyl-octyl) phthalate) concentration in µg/L stratified by age groups in boxplots.

The box is based on P25, P50, and P75, and the whiskers are based on P5 and P95. Within age groups plots are chronologically sorted by year of sampling. Colors of the boxplots refer to the data collection's region (North = violet, South = blue, West = green, East = orange, Israel = dark red). Red lines denote LOQ/LOD for the respective study. Since percentiles are only available and displayed when they are above LOQ/LOD the lower side of boxplots might be truncated. The sampling method is indicated in brackets: first morning urine void (mU), spot urine (sU) and 24-hours urine (24hU).

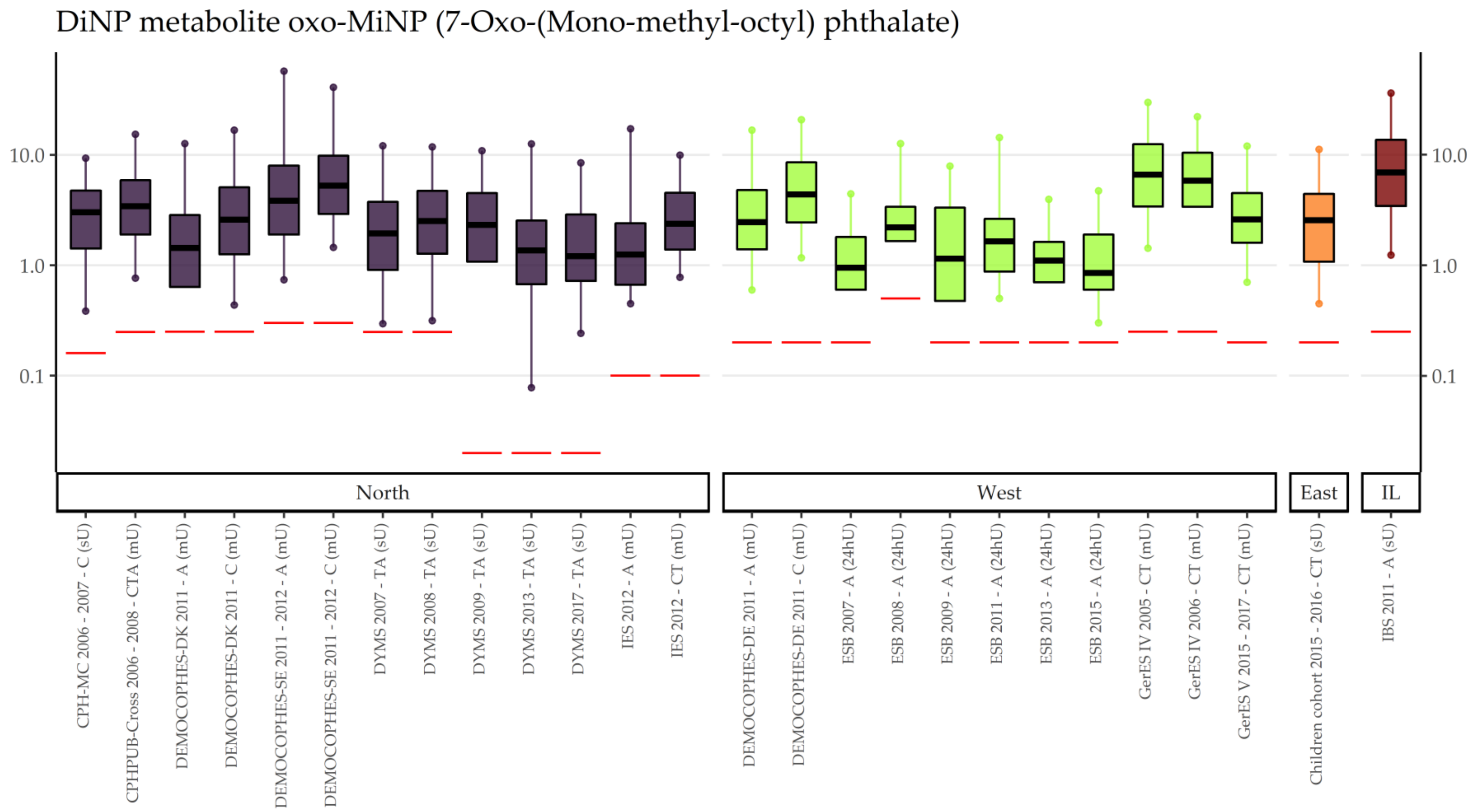

Figure S37. DiNP metabolite oxo-MiNP (7-Oxo-(Mono-methyl-octyl) phthalate) concentration in µg/L stratified by region in boxplots.

The box is based on P25, P50, and P75, and the whiskers are based on P5 and P95. Within regions plots are sorted by country and year of sampling. Colors of the boxplots refer to the data collection's region (North = violet, South = blue, West = green, East = orange, Israel = dark red). Red lines denote LOQ/LOD for the respective study. Since percentiles are only available and displayed when they are above LOQ/LOD the lower side of boxplots might be truncated. The age categories are shown in capital letters: children of the age groups 3-5 and 6-11 (C), adolescents between 12 and 19 years (T) and adults from the age groups 20-39, 40-59 and 60 years and older (A). The sampling method is indicated in brackets: first morning urine void (mU), spot urine (sU) and 24-hours urine (24hU).

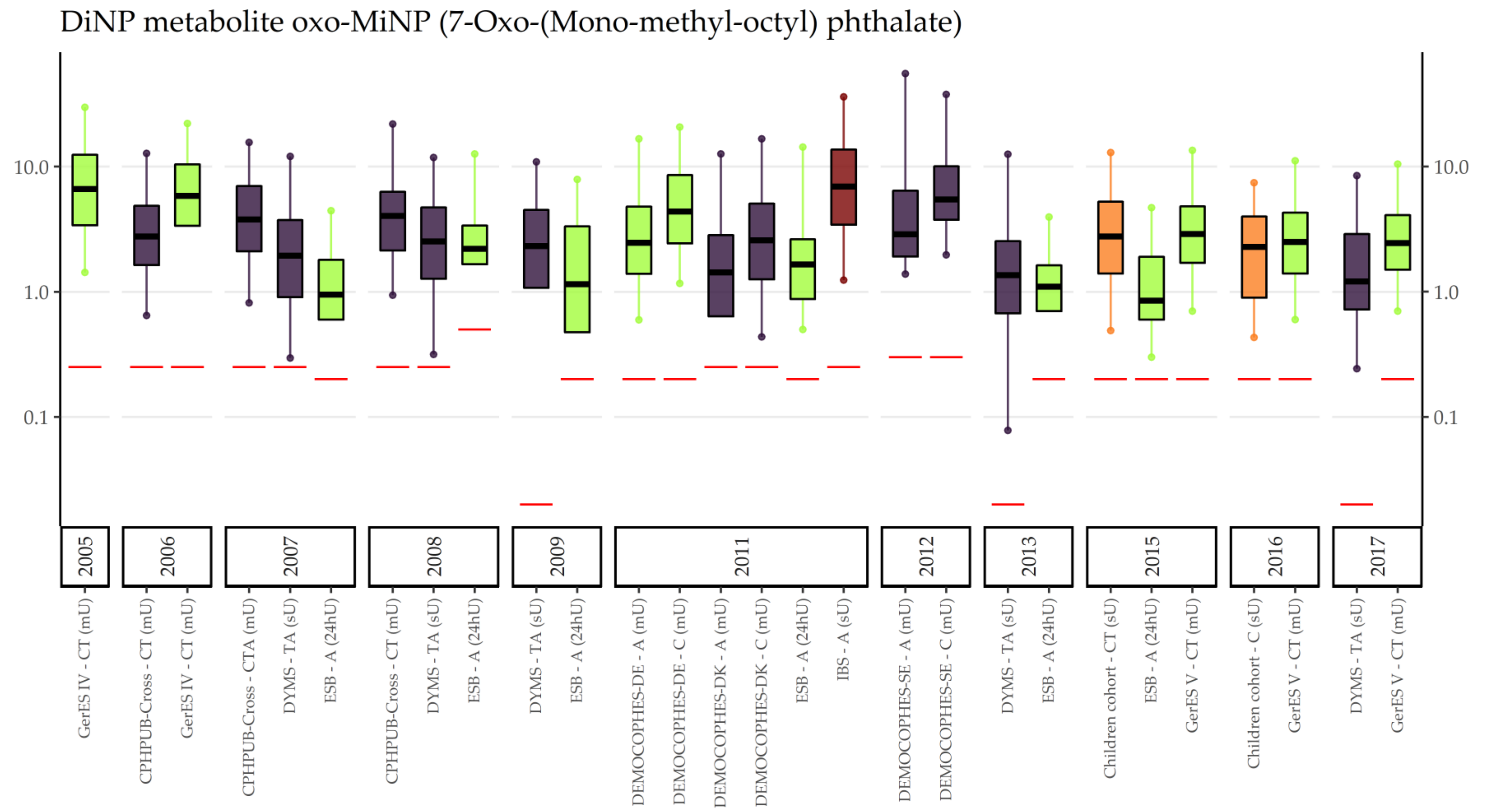

Figure S38. DiNP metabolite oxo-MiNP (7-Oxo-(Mono-methyl-octyl) phthalate) concentration in µg/L stratified by sampling year in boxplots.

The box is based on P25, P50, and P75, and the whiskers are based on P5 and P95. Within sampling years plots are sorted by country. Colors of the boxplots refer to the data collection's region (North = violet, South = blue, West = green, East = orange, Israel = dark red). Red lines denote LOQ/LOD for the respective study. Since percentiles are only available and displayed when they are above LOQ/LOD the lower side of boxplots might be truncated. The age categories are shown in capital letters: children of the age groups 3-5 and 6-11 (C), adolescents between 12 and 19 years (T) and adults from the age groups 20-39, 40-59 and 60 years and older (A). The sampling method is indicated in brackets: first morning urine void (mU), spot urine (sU) and 24-hours urine (24hU).

DiNP metabolite oxo-MiNP (7-Oxo-(Mono-methyl-octyl) phthalate)

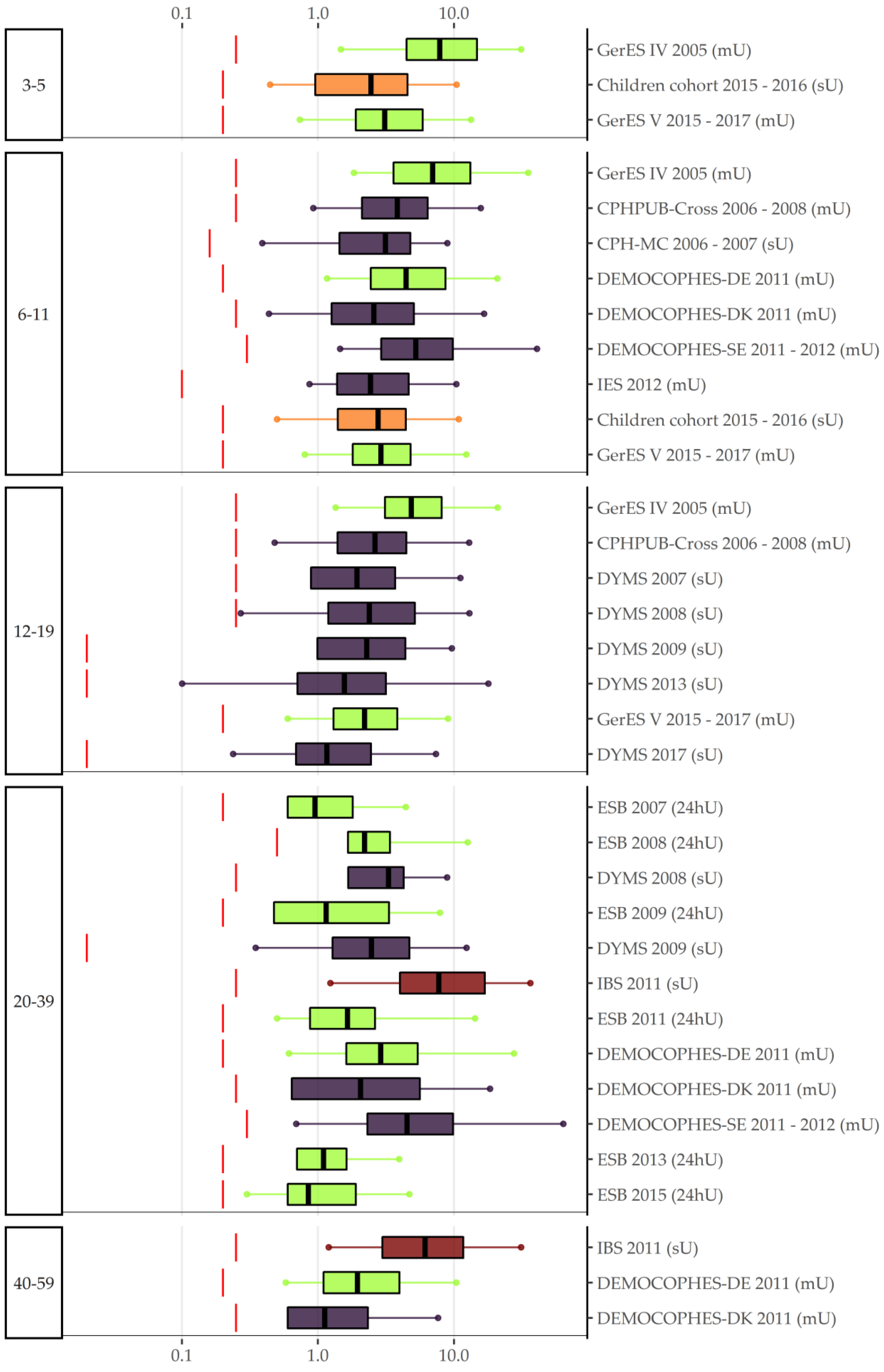

Figure S39. DiNP metabolite oxo-MiNP (7-Oxo-(Mono-methyl-octyl) phthalate) concentration in µg/L stratified by age groups in boxplots.

The box is based on P25, P50, and P75, and the whiskers are based on P5 and P95. Within age groups plots are chronologically sorted by year of sampling. Colors of the boxplots refer to the data collection's region (North = violet, South = blue, West = green, East = orange, Israel = dark red). Red lines denote LOQ/LOD for the respective study. Since percentiles are only available and displayed when they are above LOQ/LOD the lower side of boxplots might be truncated. The sampling method is indicated in brackets: first morning urine void (mU), spot urine (sU) and 24-hours urine (24hU).

DiDP metabolite cx-MiDP (Mono(2,7-methyl-7- carboxy-heptyl) phthalate)

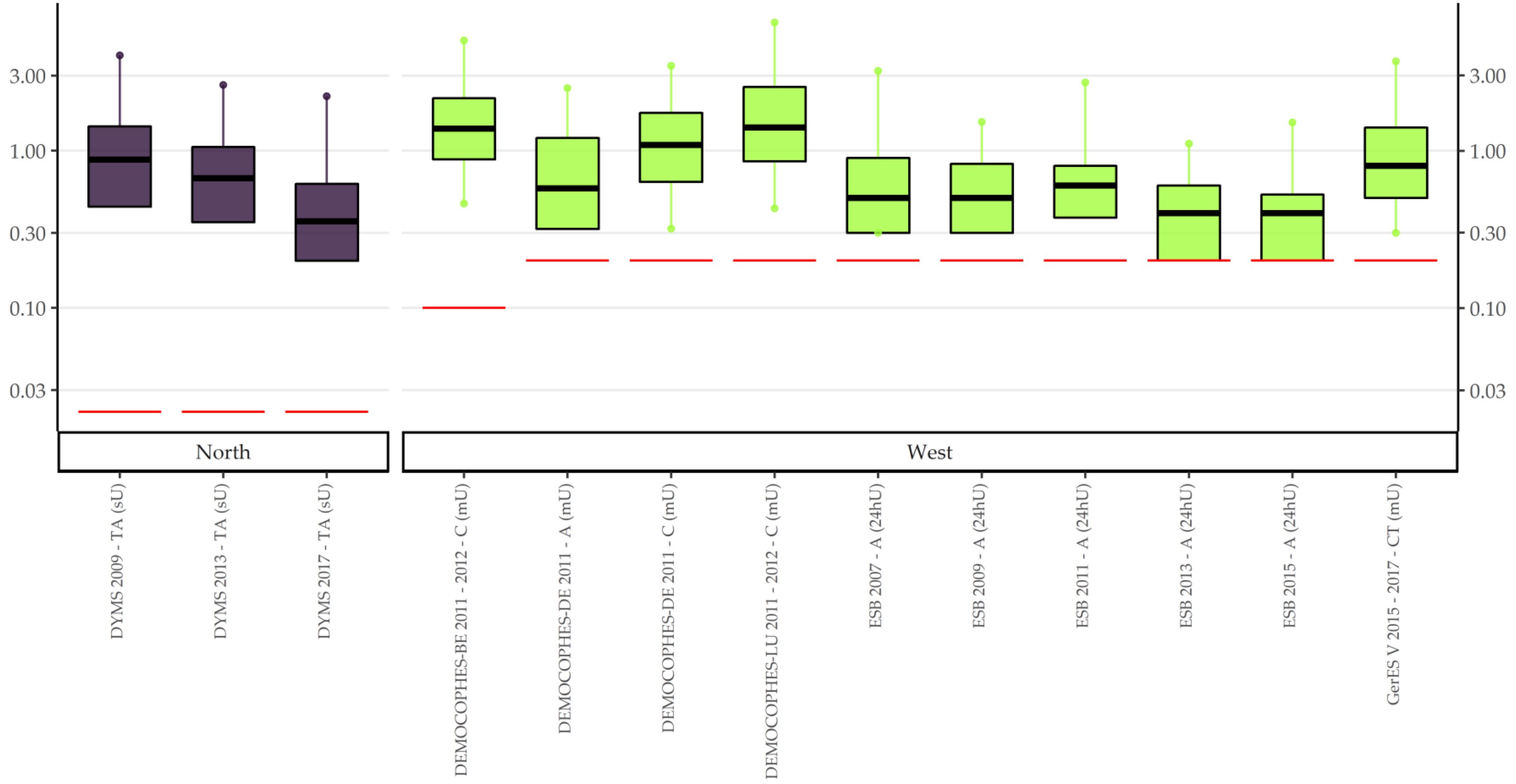

Figure S40. DiDP metabolite cx-MiDP (Mono(2,7-methyl-7- carboxy-heptyl) phthalate) concentration in µg/L stratified by region in boxplots.

The box is based on P25, P50, and P75, and the whiskers are based on P5 and P95. Within regions plots are sorted by country and year of sampling. Colors of the boxplots refer to the data collection's region (North = violet, South = blue, West = green, East = orange, Israel = dark red). Red lines denote LOQ/LOD for the respective study. Since percentiles are only available and displayed when they are above LOQ/LOD the lower side of boxplots might be truncated. The age categories are shown in capital letters: children of the age groups 3-5 and 6-11 (C), adolescents between 12 and 19 years (T) and adults from the age groups 20-39, 40-59 and 60 years and older (A). The sampling method is indicated in brackets: first morning urine void (mU), spot urine (sU) and 24-hours urine (24hU).

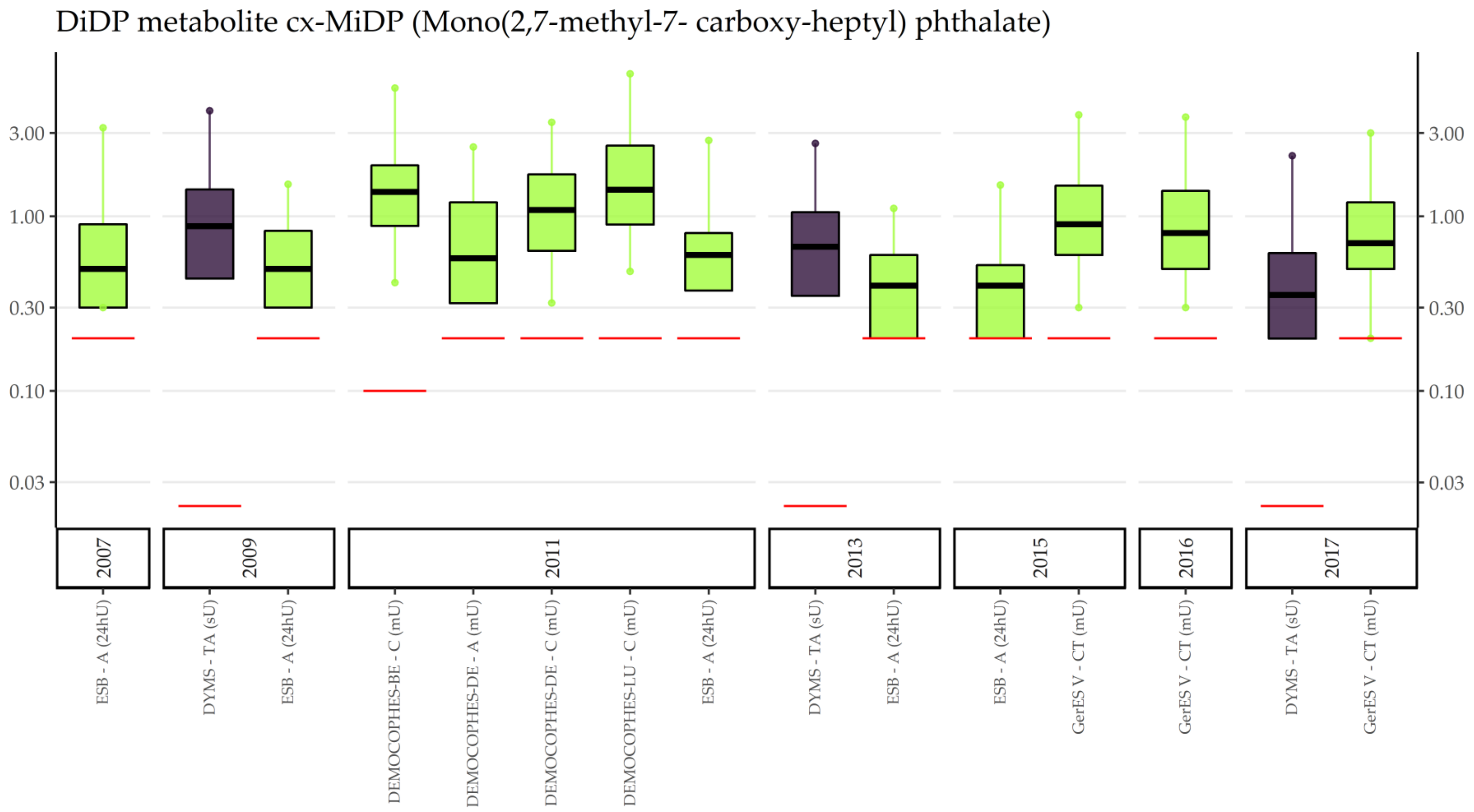

Figure S41. DiDP metabolite cx-MiDP (Mono(2,7-methyl-7- carboxy-heptyl) phthalate) concentration in µg/L stratified by sampling year in boxplots.

The box is based on P25, P50, and P75, and the whiskers are based on P5 and P95. Within sampling years plots are sorted by country. Colors of the boxplots refer to the data collection's region (North = violet, South = blue, West = green, East = orange, Israel = dark red). Red lines denote LOQ/LOD for the respective study. Since percentiles are only available and displayed when they are above LOQ/LOD the lower side of boxplots might be truncated. The age categories are shown in capital letters: children of the age groups 3-5 and 6-11 (C), adolescents between 12 and 19 years (T) and adults from the age groups 20-39, 40-59 and 60 years and older (A). The sampling method is indicated in brackets: first morning urine void (mU), spot urine (sU) and 24-hours urine (24hU).

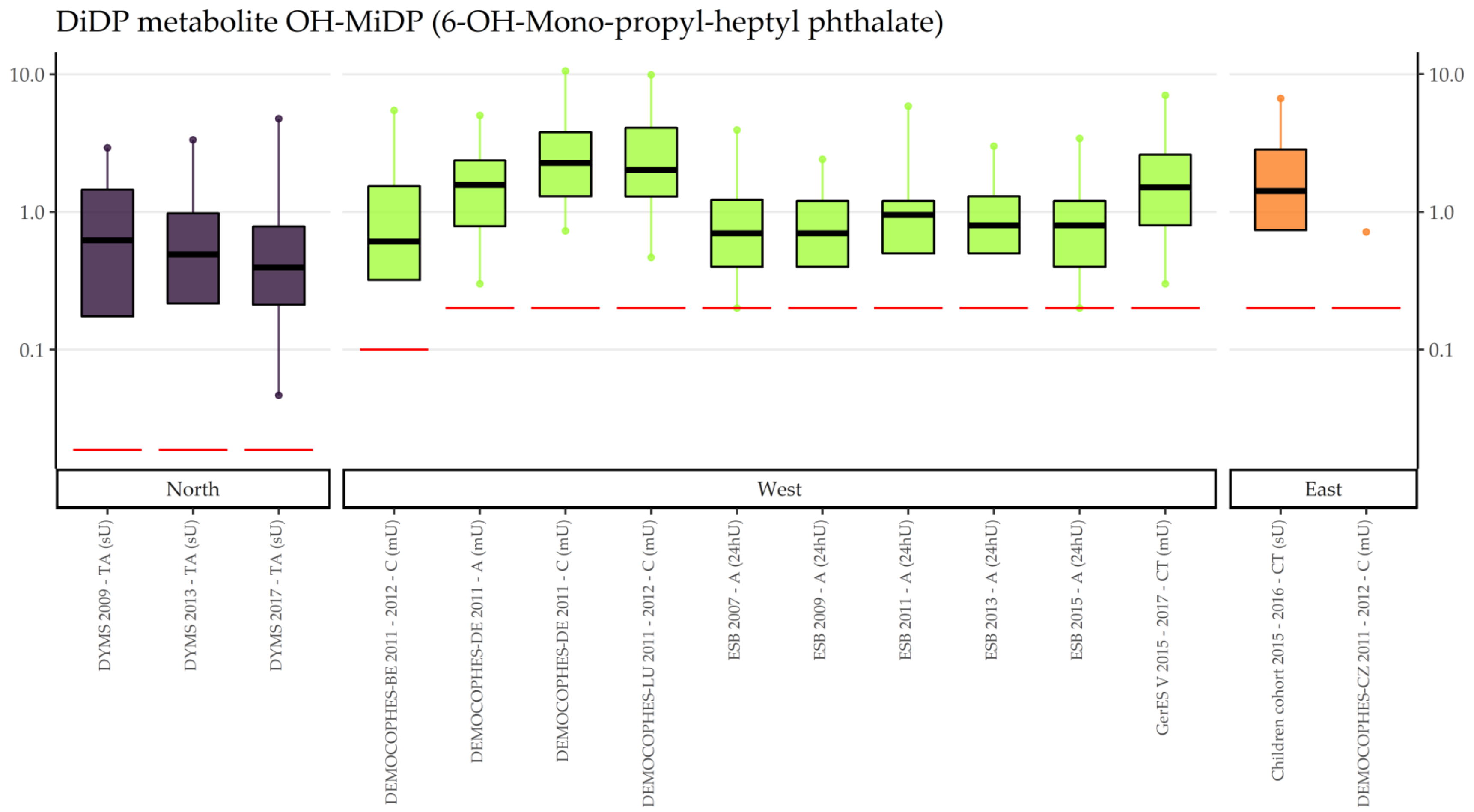

Figure S42. DiDP metabolite OH-MiDP (6-OH-Mono-propyl-heptyl phthalate) concentration in µg/L stratified by region in boxplots.

The box is based on P25, P50, and P75, and the whiskers are based on P5 and P95. Within regions plots are sorted by country and year of sampling. Colors of the boxplots refer to the data collection's region (North = violet, South = blue, West = green, East = orange, Israel = dark red). Red lines denote LOQ/LOD for the respective study. Since percentiles are only available and displayed when they are above LOQ/LOD the lower side of boxplots might be truncated. The age categories are shown in capital letters: children of the age groups 3-5 and 6-11 (C), adolescents between 12 and 19 years (T) and adults from the age groups 20-39, 40-59 and 60 years and older (A). The sampling method is indicated in brackets: first morning urine void (mU), spot urine (sU) and 24-hours urine (24hU).

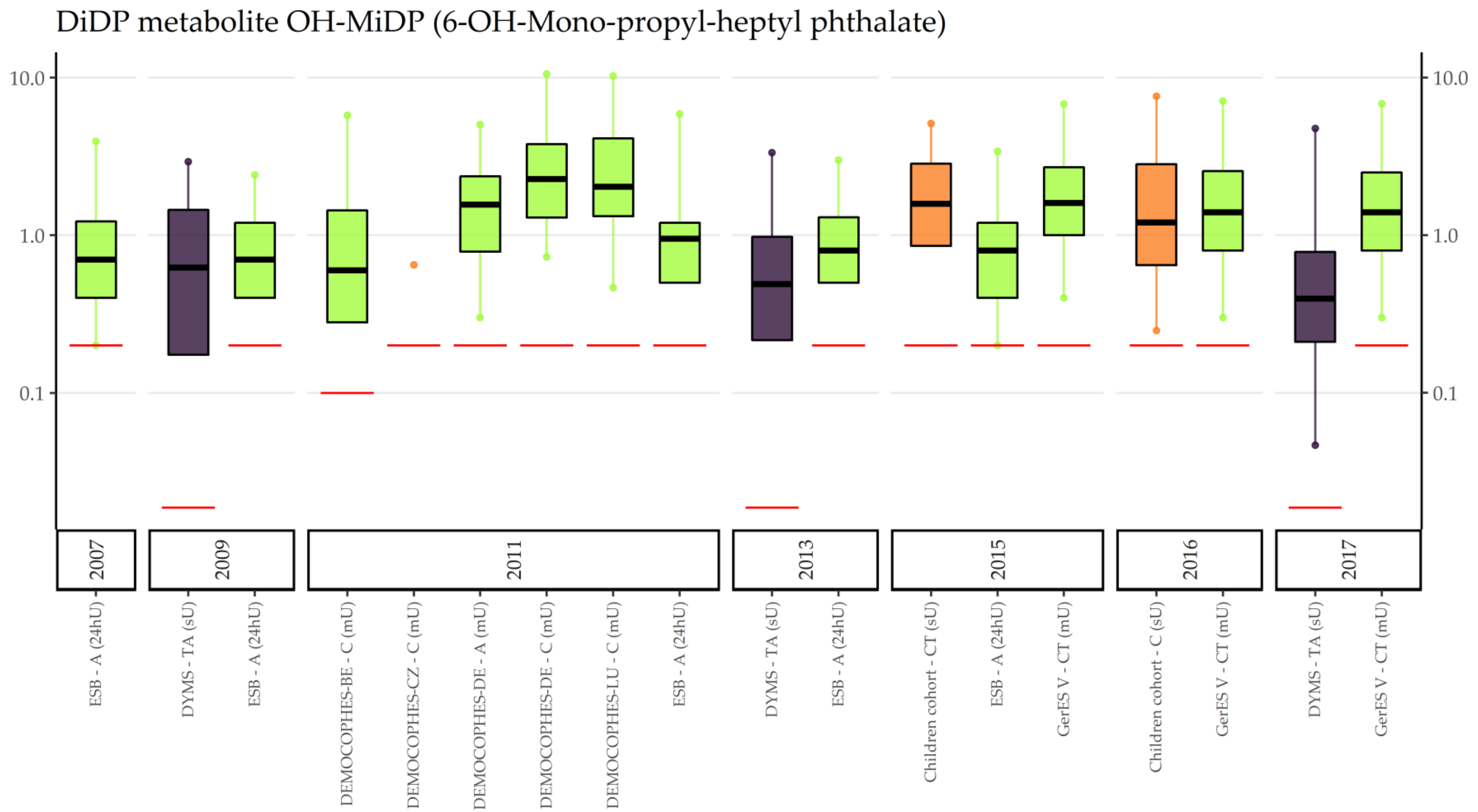

Figure S43. DiDP metabolite OH-MiDP (6-OH-Mono-propyl-heptyl phthalate) concentration in µg/L stratified by sampling year in boxplots.

The box is based on P25, P50, and P75, and the whiskers are based on P5 and P95. Within sampling years plots are sorted by country. Colors of the boxplots refer to the data collection's region (North = violet, South = blue, West = green, East = orange, Israel = dark red). Red lines denote LOQ/LOD for the respective study. Since percentiles are only available and displayed when they are above LOQ/LOD the lower side of boxplots might be truncated. The age categories are shown in capital letters: children of the age groups 3-5 and 6-11 (C), adolescents between 12 and 19 years (T) and adults from the age groups 20-39, 40-59 and 60 years and older (A). The sampling method is indicated in brackets: first morning urine void (mU), spot urine (sU) and 24-hours urine (24hU).

DiDP metabolite OH-MiDP (6-OH-Mono-propyl-heptyl phthalate)

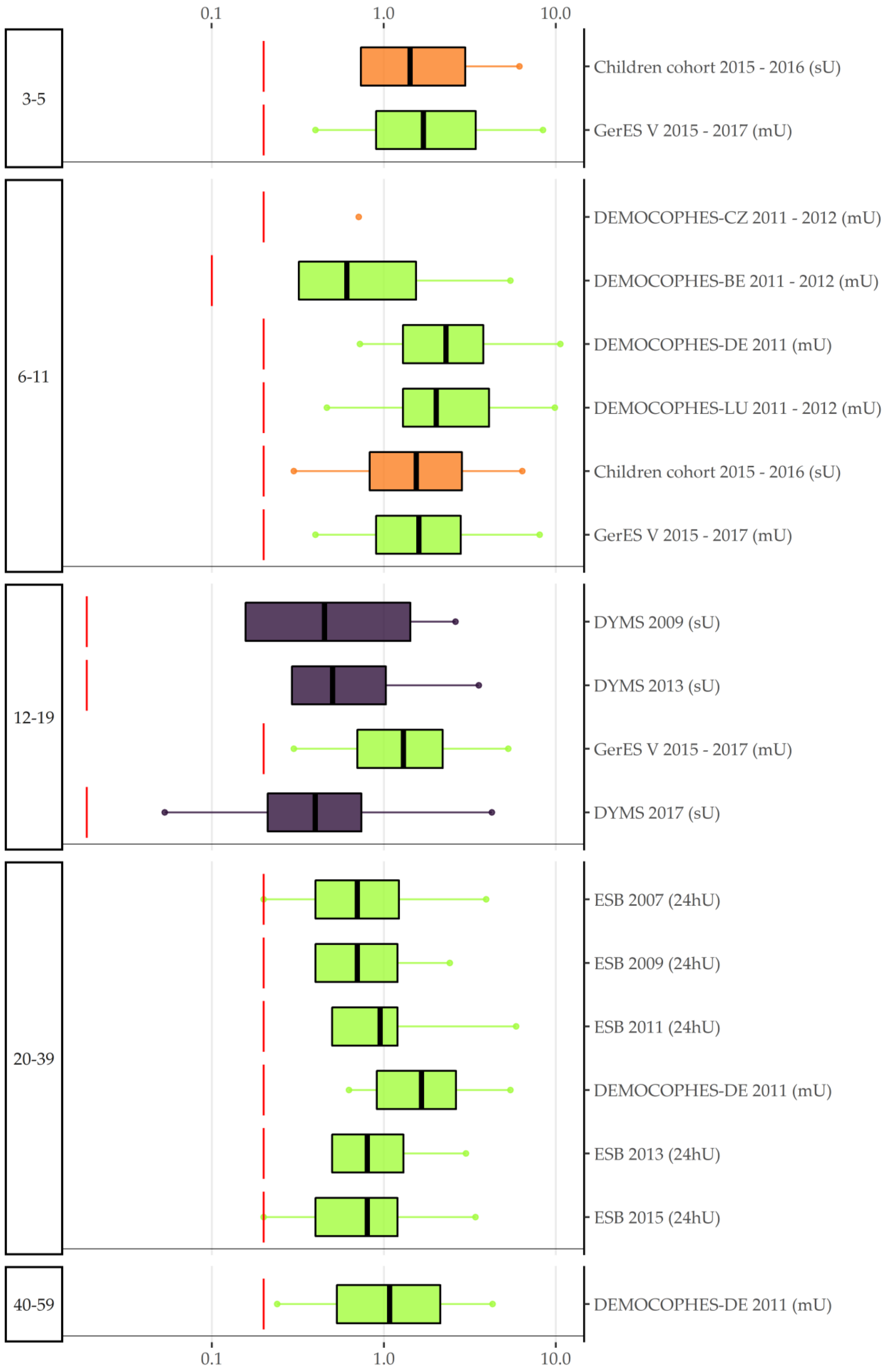

Figure S44. DiDP metabolite OH-MiDP (6-OH-Mono-propyl-heptyl phthalate) concentration in µg/L stratified by age groups in boxplots.

The box is based on P25, P50, and P75, and the whiskers are based on P5 and P95. Within age groups plots are chronologically sorted by year of sampling. Colors of the boxplots refer to the data collection's region (North = violet, South = blue, West = green, East = orange, Israel = dark red). Red lines denote LOQ/LOD for the respective study. Since percentiles are only available and displayed when they are above LOQ/LOD the lower side of boxplots might be truncated. The sampling method is indicated in brackets: first morning urine void (mU), spot urine (sU) and 24-hours urine (24hU).

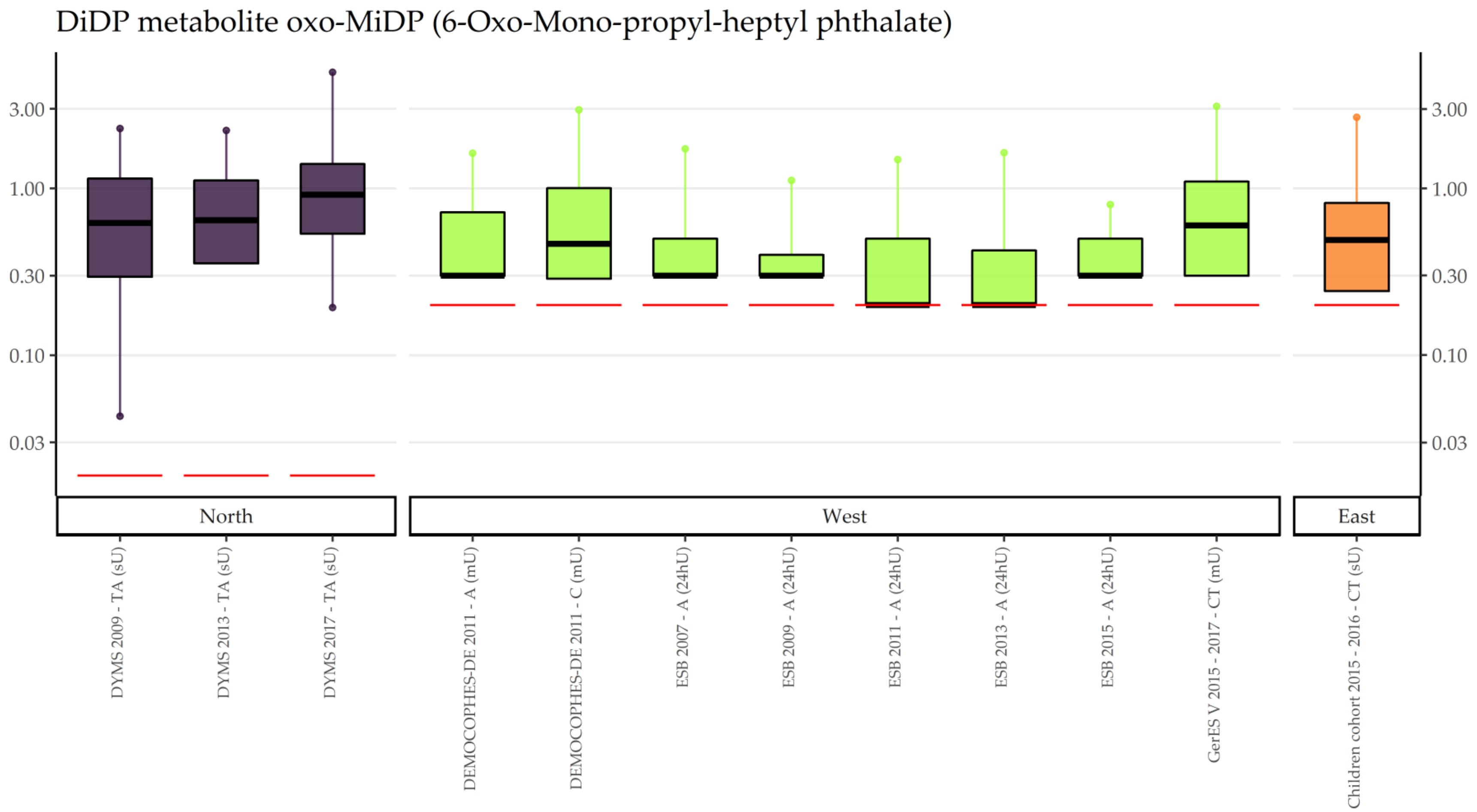

Figure S45. DiDP metabolite oxo-MiDP (6-Oxo-Mono-propyl-heptyl phthalate) concentration in µg/L stratified by region in boxplots.

The box is based on P25, P50, and P75, and the whiskers are based on P5 and P95. Within regions plots are sorted by country and year of sampling. Colors of the boxplots refer to the data collection's region (North = violet, South = blue, West = green, East = orange, Israel = dark red). Red lines denote LOQ/LOD for the respective study. Since percentiles are only available and displayed when they are above LOQ/LOD the lower side of boxplots might be truncated. The age categories are shown in capital letters: children of the age groups 3-5 and 6-11 (C), adolescents between 12 and 19 years (T) and adults from the age groups 20-39, 40-59 and 60 years and older (A). The sampling method is indicated in brackets: first morning urine void (mU), spot urine (sU) and 24-hours urine (24hU).

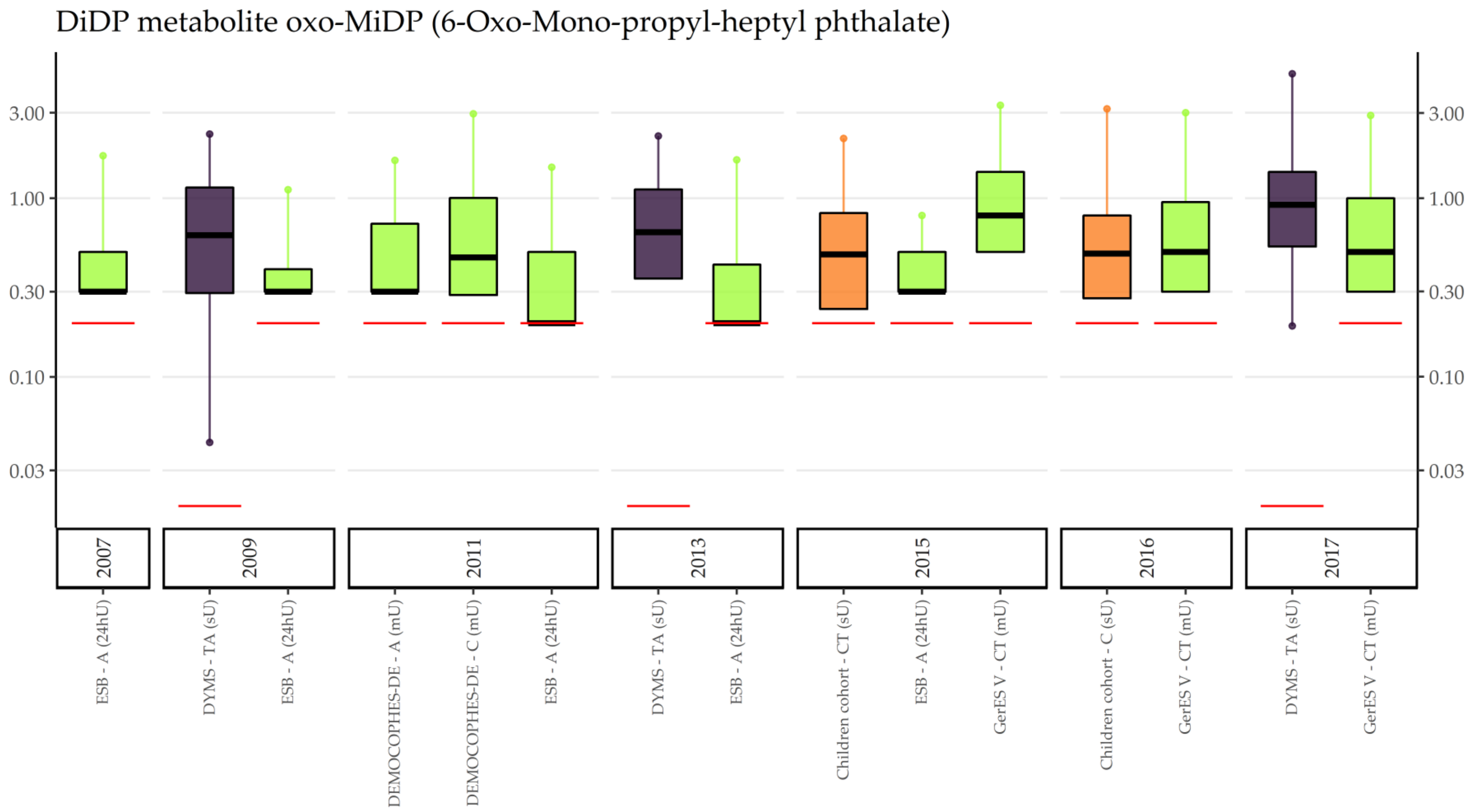

Figure S46. DiDP metabolite oxo-MiDP (6-Oxo-Mono-propyl-heptyl phthalate) concentration in µg/L stratified by sampling year in boxplots.

The box is based on P25, P50, and P75, and the whiskers are based on P5 and P95. Within sampling years plots are sorted by country. Colors of the boxplots refer to the data collection's region (North = violet, South = blue, West = green, East = orange, Israel = dark red). Red lines denote LOQ/LOD for the respective study. Since percentiles are only available and displayed when they are above LOQ/LOD the lower side of boxplots might be truncated. The age categories are shown in capital letters: children of the age groups 3-5 and 6-11 (C), adolescents between 12 and 19 years (T) and adults from the age groups 20-39, 40-59 and 60 years and older (A). The sampling method is indicated in brackets: first morning urine void (mU), spot urine (sU) and 24-hours urine (24hU).

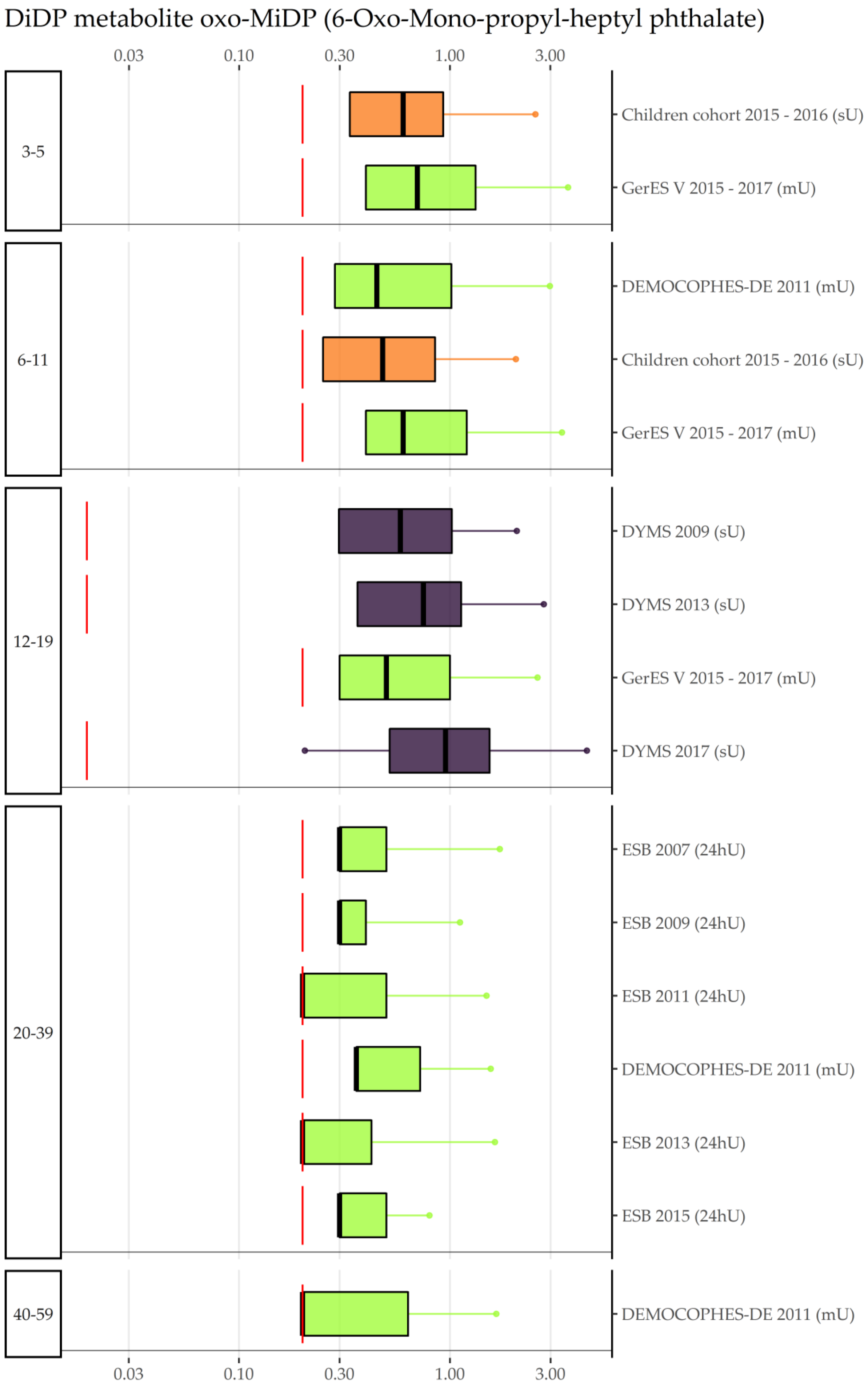

Figure S47. DiDP metabolite oxo-MiDP (6-Oxo-Mono-propyl-heptyl phthalate) concentration in µg/L stratified by age groups in boxplots.

The box is based on P25, P50, and P75, and the whiskers are based on P5 and P95. Within age groups plots are chronologically sorted by year of sampling. Colors of the boxplots refer to the data collection's region (North = violet, South = blue, West = green, East = orange, Israel = dark red). Red lines denote LOQ/LOD for the respective study. Since percentiles are only available and displayed when they are above LOQ/LOD the lower side of boxplots might be truncated. The sampling method is indicated in brackets: first morning urine void (mU), spot urine (sU) and 24-hours urine (24hU).

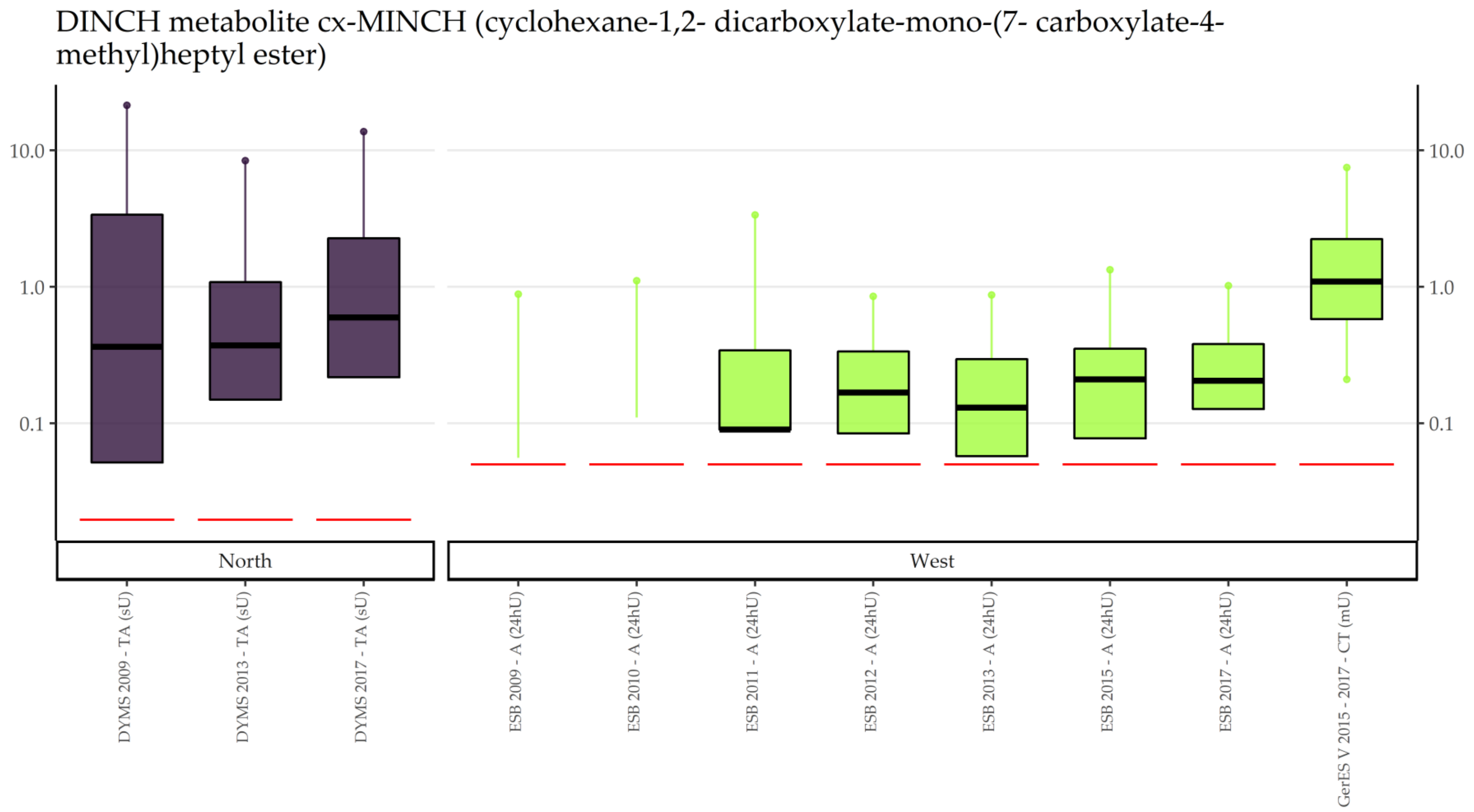

Figure S48. DINCH metabolite cx-MINCH (cyclohexane-1,2- dicarboxylate-mono-(7- carboxylate-4- methyl)heptyl ester) concentration in µg/L stratified by region in boxplots.

The box is based on P25, P50, and P75, and the whiskers are based on P5 and P95. Within regions plots are sorted by country and year of sampling. Colors of the boxplots refer to the data collection's region (North = violet, South = blue, West = green, East = orange, Israel = dark red). Red lines denote LOQ/LOD for the respective study. Since percentiles are only available and displayed when they are above LOQ/LOD the lower side of boxplots might be truncated. The age categories are shown in capital letters: children of the age groups 3-5 and 6-11 (C), adolescents between 12 and 19 years (T) and adults from the age groups 20-39, 40-59 and 60 years and older (A). The sampling method is indicated in brackets: first morning urine void (mU), spot urine (sU) and 24-hours urine (24hU).

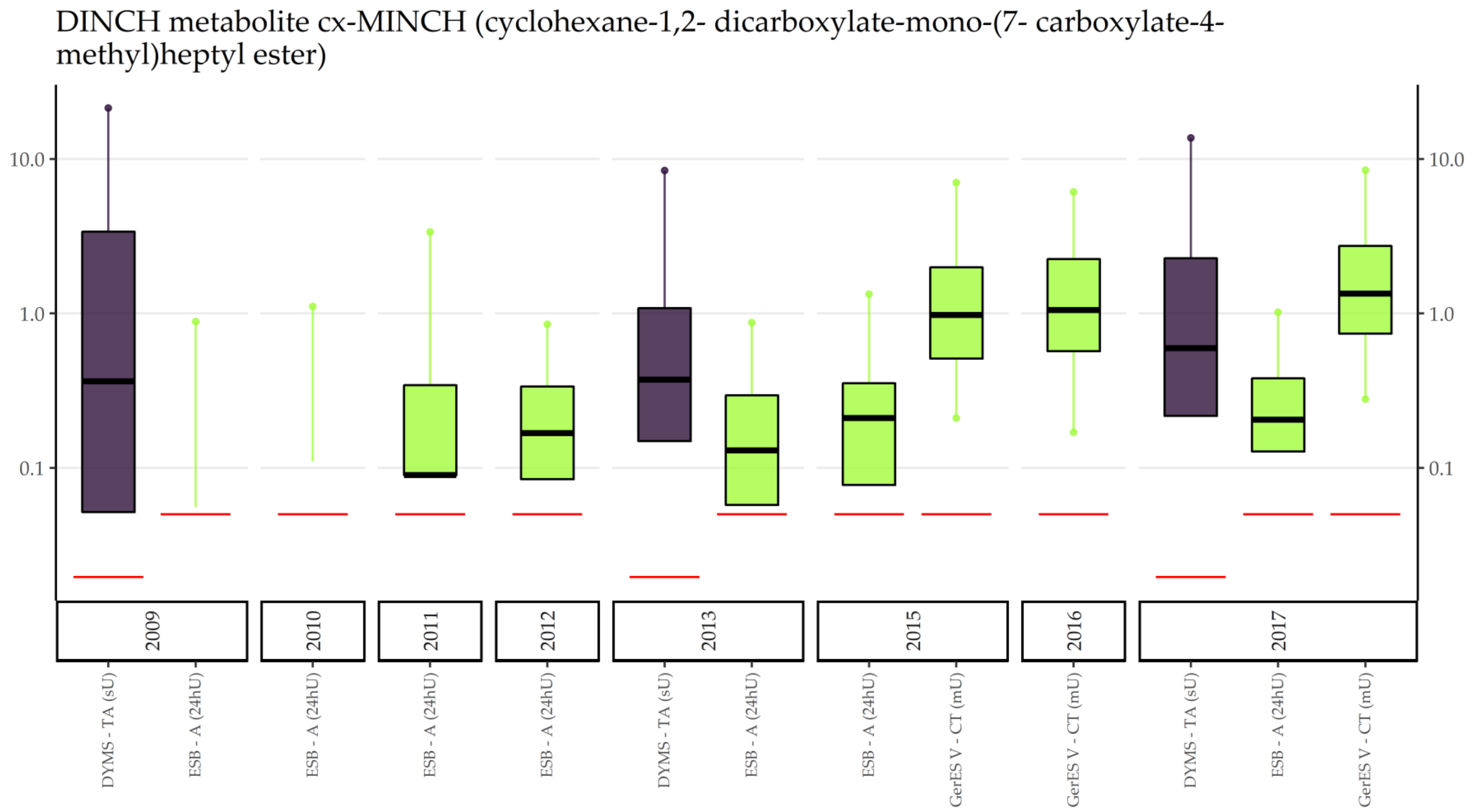

Figure S49. DINCH metabolite cx-MINCH (cyclohexane-1,2- dicarboxylate-mono-(7- carboxylate-4- methyl)heptyl ester) concentration in µg/L stratified by sampling year in boxplots.

The box is based on P25, P50, and P75, and the whiskers are based on P5 and P95. Within sampling years plots are sorted by country. Colors of the boxplots refer to the data collection's region (North = violet, South = blue, West = green, East = orange, Israel = dark red). Red lines denote LOQ/LOD for the respective study. Since percentiles are only available and displayed when they are above LOQ/LOD the lower side of boxplots might be truncated. The age categories are shown in capital letters: children of the age groups 3-5 and 6-11 (C), adolescents between 12 and 19 years (T) and adults from the age groups 20-39, 40-59 and 60 years and older (A). The sampling method is indicated in brackets: first morning urine void (mU), spot urine (sU) and 24-hours urine (24hU).

DINCH metabolite cx-MINCH (cyclohexane-1,2- dicarboxylate-mono-(7-carboxylate-4- methyl)heptyl ester)

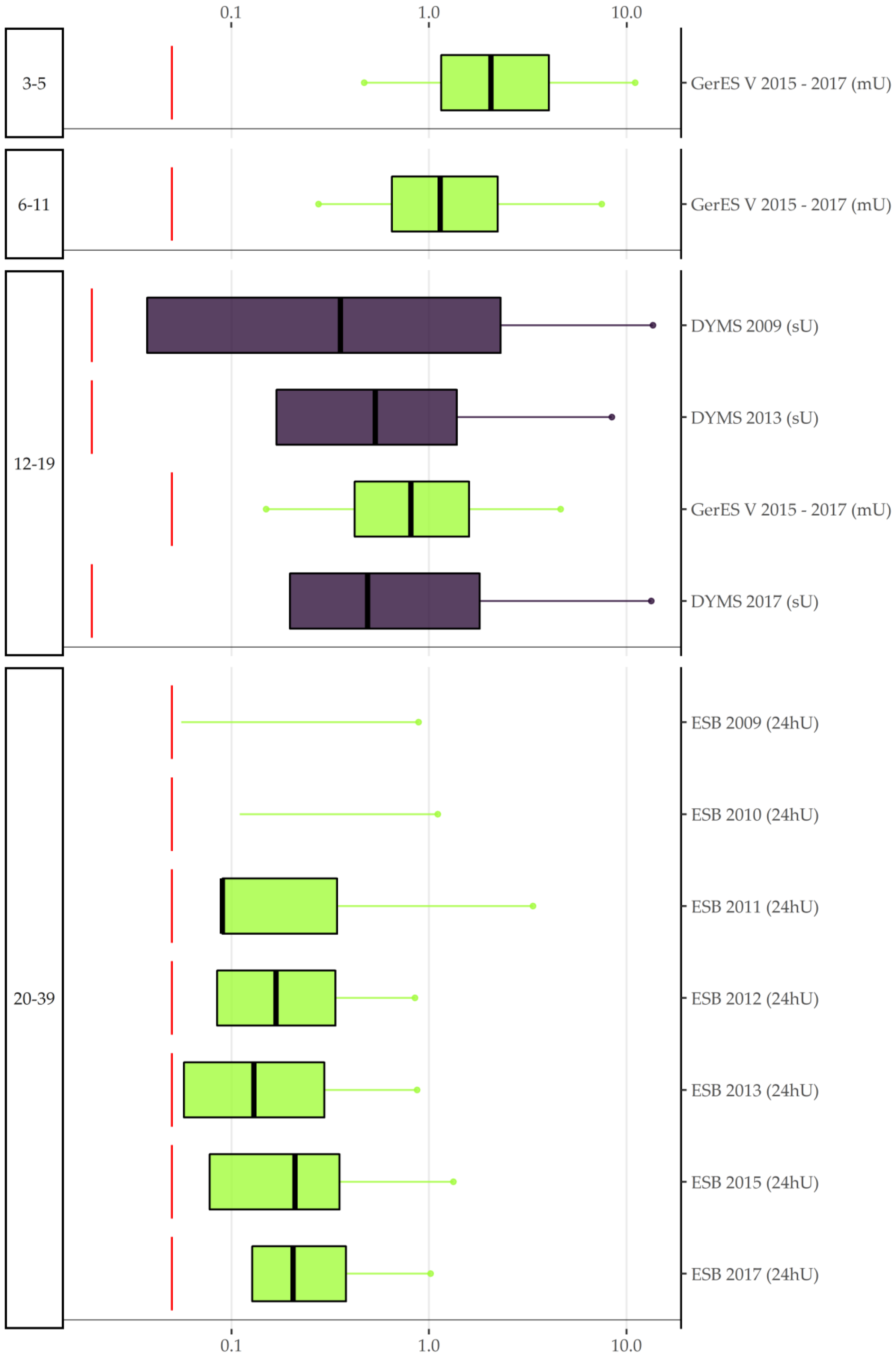

Figure S50. DINCH metabolite cx-MINCH (cyclohexane-1,2- dicarboxylate-mono-(7-carboxylate-4- methyl)heptyl ester) concentration in µg/L stratified by age groups in boxplots.

The box is based on P25, P50, and P75, and the whiskers are based on P5 and P95. Within age groups plots are chronologically sorted by year of sampling. Colors of the boxplots refer to the data collection's region (North = violet, South = blue, West = green, East = orange, Israel = dark red). Red lines denote LOQ/LOD for the respective study. Since percentiles are only available and displayed when they are above LOQ/LOD the lower side of boxplots might be truncated. The sampling method is indicated in brackets: first morning urine void (mU), spot urine (sU) and 24-hours urine (24hU).

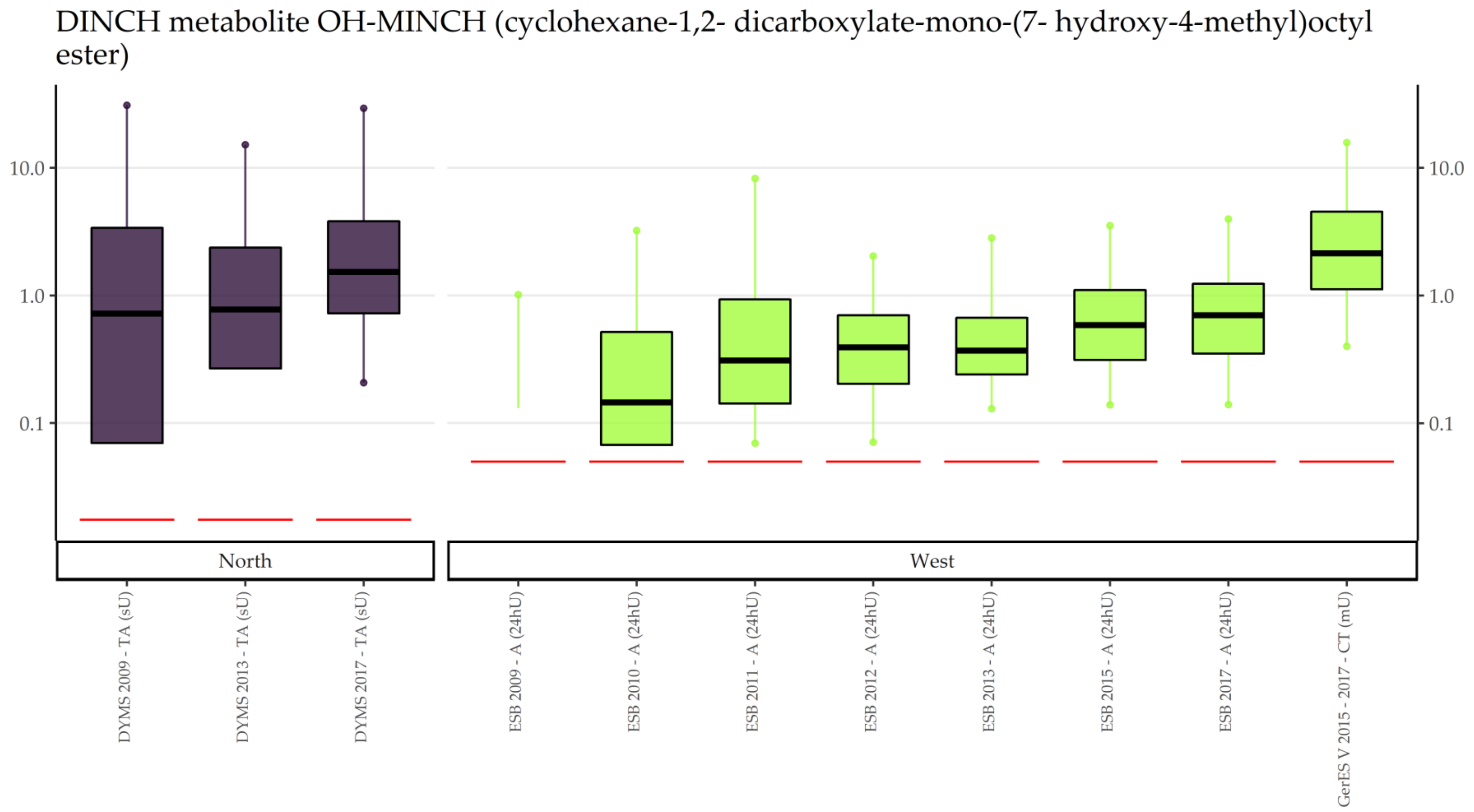

Figure S51. DINCH metabolite OH-MINCH (cyclohexane-1,2- dicarboxylate-mono-(7- hydroxy-4-methyl)octyl ester) concentration in µg/L stratified by region in boxplots.

The box is based on P25, P50, and P75, and the whiskers are based on P5 and P95. Within regions plots are sorted by country and year of sampling. Colors of the boxplots refer to the data collection's region (North = violet, South = blue, West = green, East = orange, Israel = dark red). Red lines denote LOQ/LOD for the respective study. Since percentiles are only available and displayed when they are above LOQ/LOD the lower side of boxplots might be truncated. The age categories are shown in capital letters: children of the age groups 3-5 and 6-11 (C), adolescents between 12 and 19 years (T) and adults from the age groups 20-39, 40-59 and 60 years and older (A). The sampling method is indicated in brackets: first morning urine void (mU), spot urine (sU) and 24-hours urine (24hU).

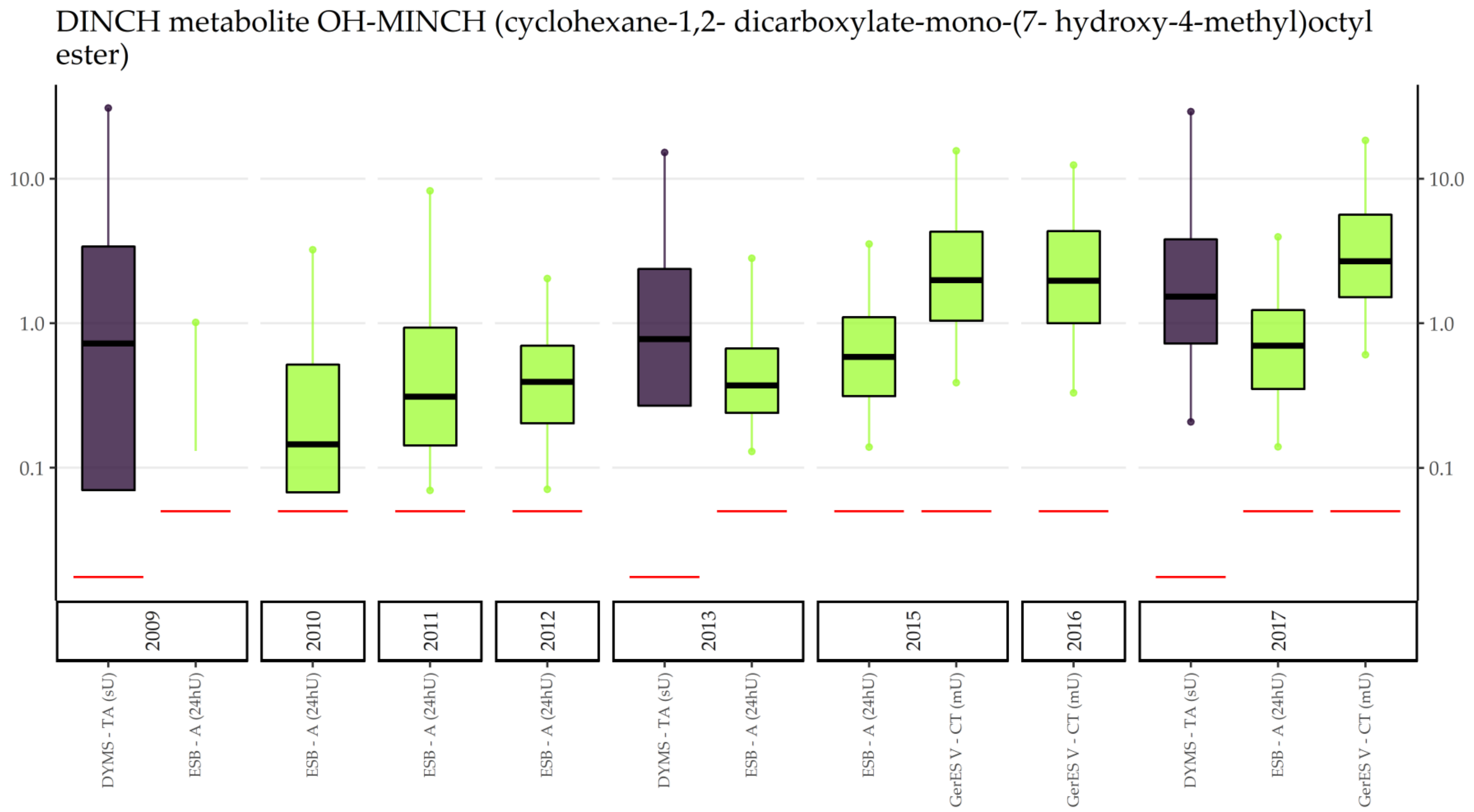

Figure S52. DINCH metabolite OH-MINCH (cyclohexane-1,2- dicarboxylate-mono-(7- hydroxy-4-methyl)octyl ester) concentration in µg/L stratified by sampling year in boxplots.

The box is based on P25, P50, and P75, and the whiskers are based on P5 and P95. Within sampling years plots are sorted by country. Colors of the boxplots refer to the data collection's region (North = violet, South = blue, West = green, East = orange, Israel = dark red). Red lines denote LOQ/LOD for the respective study. Since percentiles are only available and displayed when they are above LOQ/LOD the lower side of boxplots might be truncated. The age categories are shown in capital letters: children of the age groups 3-5 and 6-11 (C), adolescents between 12 and 19 years (T) and adults from the age groups 20-39, 40-59 and 60 years and older (A). The sampling method is indicated in brackets: first morning urine void (mU), spot urine (sU) and 24-hours urine (24hU).

DINCH metabolite OH-MINCH (cyclohexane-1,2- dicarboxylate-mono-(7-hydroxy-4-methyl)octyl ester)

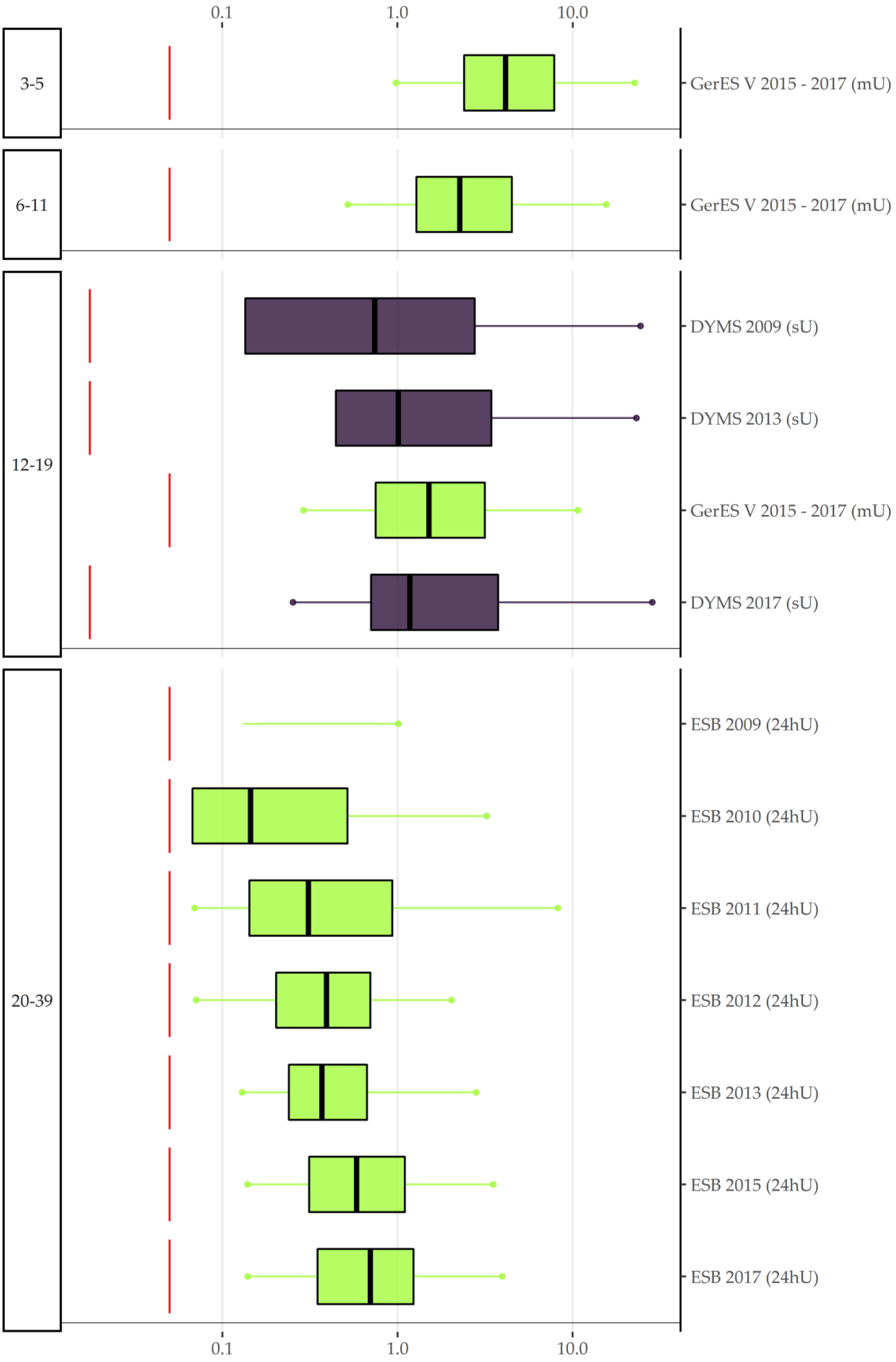

Figure S53. DINCH metabolite OH-MINCH (cyclohexane-1,2- dicarboxylate-mono-(7- hydroxy-4-methyl)octyl ester) concentration in µg/L stratified by age groups in boxplots.

The box is based on P25, P50, and P75, and the whiskers are based on P5 and P95. Within age groups plots are chronologically sorted by year of sampling. Colors of the boxplots refer to the data collection's region (North = violet, South = blue, West = green, East = orange, Israel = dark red). Red lines denote LOQ/LOD for the respective study. Since percentiles are only available and displayed when they are above LOQ/LOD the lower side of boxplots might be truncated. The sampling method is indicated in brackets: first morning urine void (mU), spot urine (sU) and 24-hours urine (24hU).

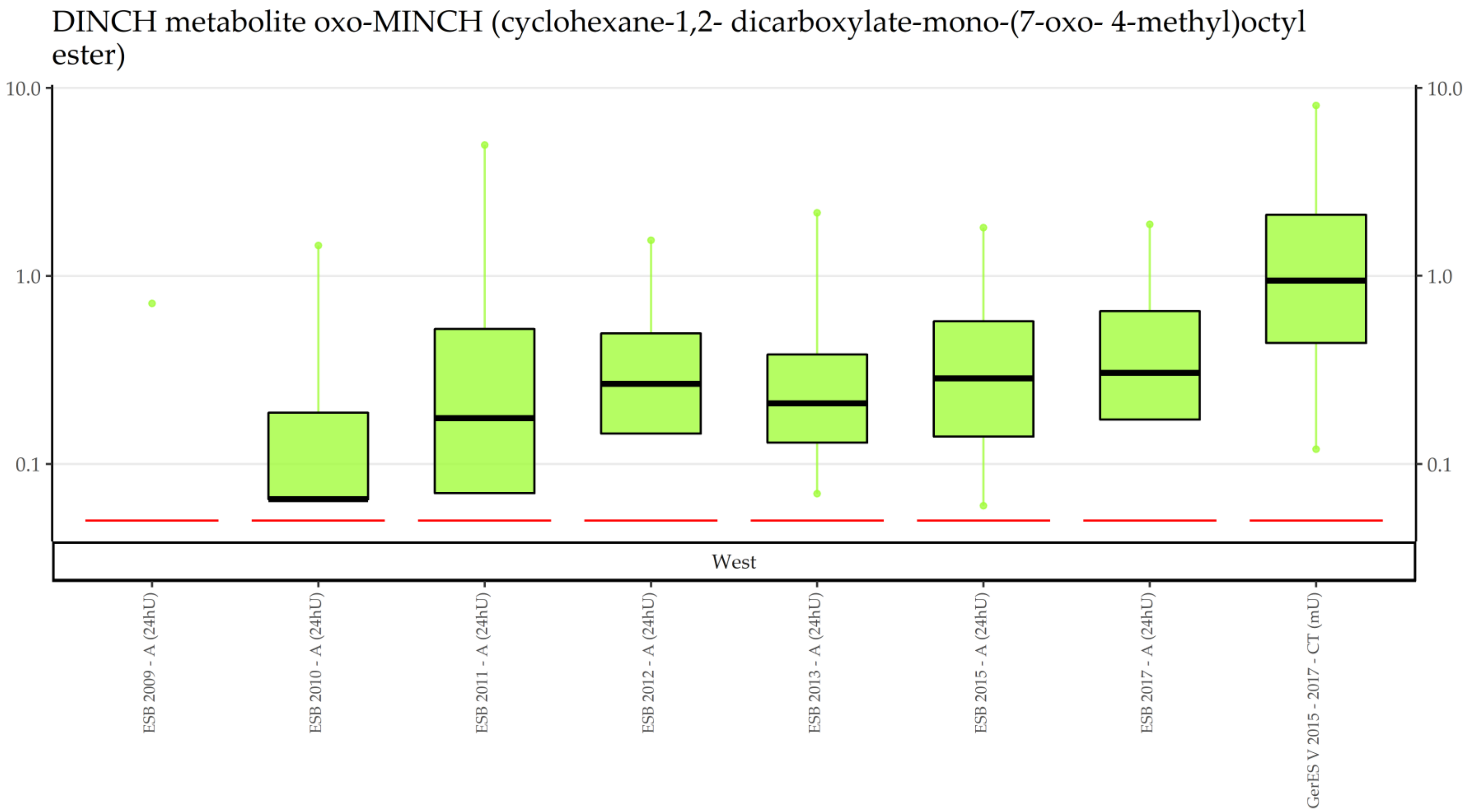

Figure S54. DINCH metabolite oxo-MINCH (cyclohexane-1,2- dicarboxylate-mono-(7-oxo- 4-methyl)octyl ester) concentration in µg/L stratified by region in boxplots.

The box is based on P25, P50, and P75, and the whiskers are based on P5 and P95. Within regions plots are sorted by country and year of sampling. Colors of the boxplots refer to the data collection's region (North = violet, South = blue, West = green, East = orange, Israel = dark red). Red lines denote LOQ/LOD for the respective study. Since percentiles are only available and displayed when they are above LOQ/LOD the lower side of boxplots might be truncated. The age categories are shown in capital letters: children of the age groups 3-5 and 6-11 (C), adolescents between 12 and 19 years (T) and adults from the age groups 20-39, 40-59 and 60 years and older (A). The sampling method is indicated in brackets: first morning urine void (mU), spot urine (sU) and 24-hours urine (24hU).

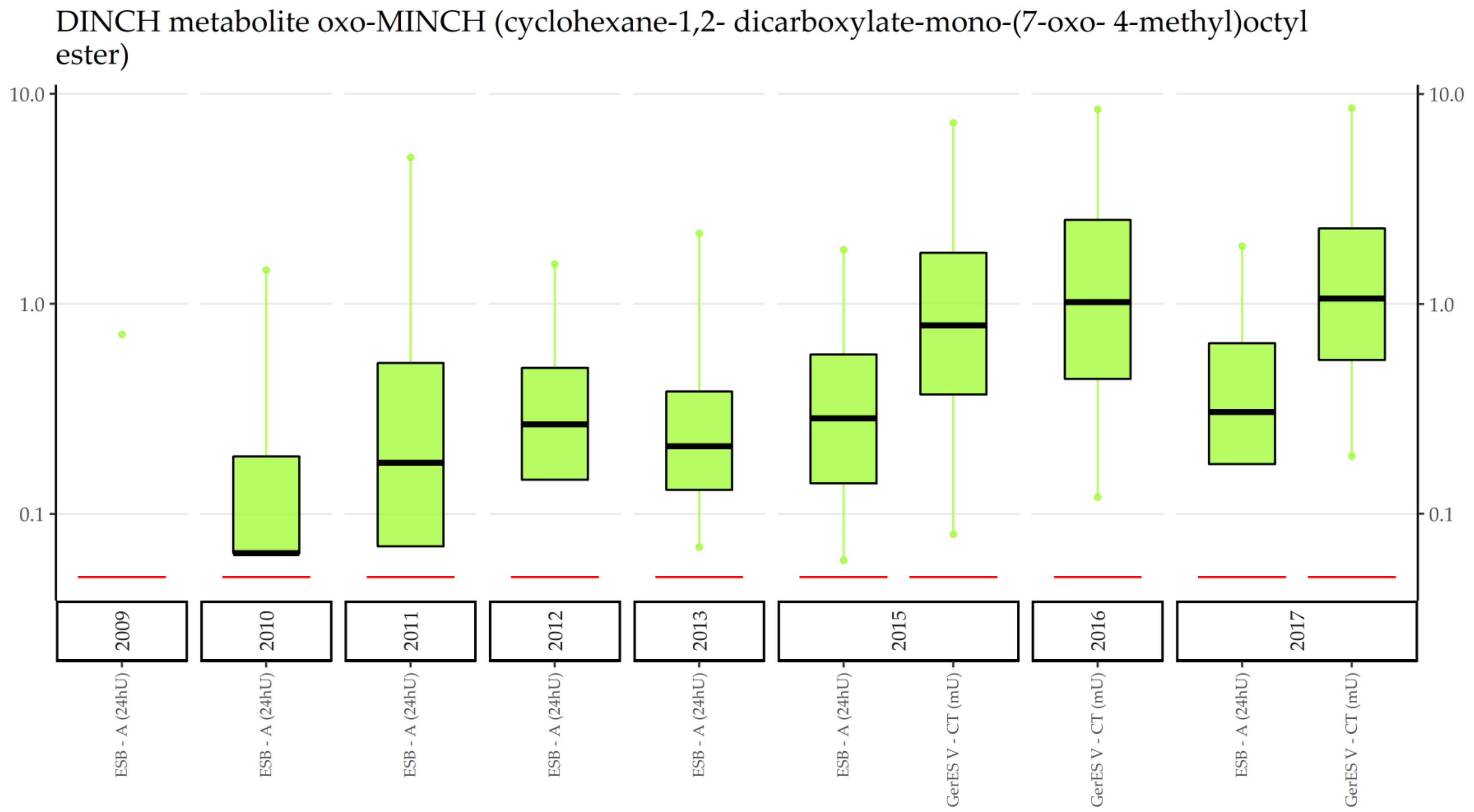

Figure S55. DINCH metabolite oxo-MINCH (cyclohexane-1,2- dicarboxylate-mono-(7-oxo- 4-methyl)octyl ester) concentration in µg/L stratified by sampling year in boxplots.

The box is based on P25, P50, and P75, and the whiskers are based on P5 and P95. Within sampling years plots are sorted by country. Colors of the boxplots refer to the data collection's region (North = violet, South = blue, West = green, East = orange, Israel = dark red). Red lines denote LOQ/LOD for the respective study. Since percentiles are only available and displayed when they are above LOQ/LOD the lower side of boxplots might be truncated. The age categories are shown in capital letters: children of the age groups 3-5 and 6-11 (C), adolescents between 12 and 19 years (T) and adults from the age groups 20-39, 40-59 and 60 years and older (A). The sampling method is indicated in brackets: first morning urine void (mU), spot urine (sU) and 24-hours urine (24hU).

DINCH metabolite oxo-MINCH (cyclohexane-1,2-dicarboxylate-mono-(7-oxo- 4-methyl)octyl ester)

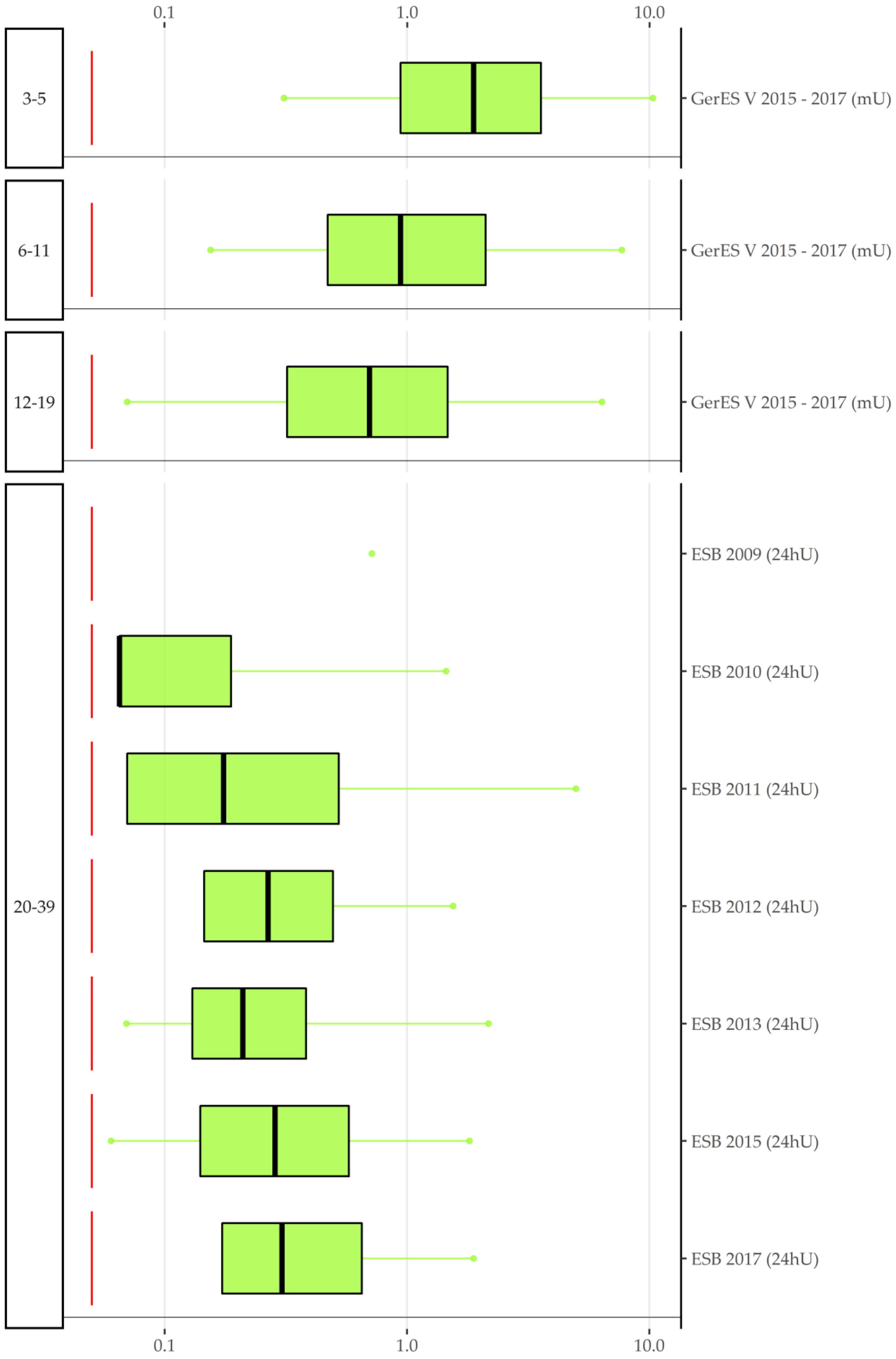

Figure S56. DINCH metabolite oxo-MINCH (cyclohexane-1,2- dicarboxylate-mono-(7-oxo- 4-methyl)octyl ester) concentration in µg/L stratified by age groups in boxplots.

The box is based on P25, P50, and P75, and the whiskers are based on P5 and P95. Within age groups plots are chronologically sorted by year of sampling. Colors of the boxplots refer to the data collection's region (North = violet, South = blue, West = green, East = orange, Israel = dark red). Red lines denote LOQ/LOD for the respective study. Since percentiles are only available and displayed when they are above LOQ/LOD the lower side of boxplots might be truncated. The sampling method is indicated in brackets: first morning urine void (mU), spot urine (sU) and 24-hours urine (24hU).
